# New view on the organization and evolution of Palaeognathae mitogenomes poses the question on the ancestral gene rearrangement in Aves

**DOI:** 10.1186/s12864-020-07284-5

**Published:** 2020-12-07

**Authors:** Adam Dawid Urantówka, Aleksandra Kroczak, Paweł Mackiewicz

**Affiliations:** 1 Department of Genetics, Wroclaw University of Environmental and Life Sciences, 7 Kozuchowska Street, 51-631 Wroclaw, Poland; 2 Department of Bioinformatics and Genomics, Faculty of Biotechnology, University of Wrocław, 14a Fryderyka Joliot-Curie Street, 50-383 Wrocław, Poland

**Keywords:** Ancestral state, Aves, Duplication, Mitochondrial genome, Mitogenome, Neognathae, Palaeognathae, Phylogeny, Rearrangement

## Abstract

**Background:**

Bird mitogenomes differ from other vertebrates in gene rearrangement. The most common avian gene order, identified first in *Gallus gallus*, is considered ancestral for all Aves. However, other rearrangements including a duplicated control region and neighboring genes have been reported in many representatives of avian orders. The repeated regions can be easily overlooked due to inappropriate DNA amplification or genome sequencing. This raises a question about the actual prevalence of mitogenomic duplications and the validity of the current view on the avian mitogenome evolution. In this context, Palaeognathae is especially interesting because is sister to all other living birds, i.e. Neognathae. So far, a unique duplicated region has been found in one palaeognath mitogenome, that of *Eudromia elegans*.

**Results:**

Therefore, we applied an appropriate PCR strategy to look for omitted duplications in other palaeognaths. The analyses revealed the duplicated control regions with adjacent genes in *Crypturellus, Rhea* and *Struthio* as well as *ND6* pseudogene in three moas. The copies are very similar and were subjected to concerted evolution. Mapping the presence and absence of duplication onto the Palaeognathae phylogeny indicates that the duplication was an ancestral state for this avian group. This feature was inherited by early diverged lineages and lost two times in others. Comparison of incongruent phylogenetic trees based on mitochondrial and nuclear sequences showed that two variants of mitogenomes could exist in the evolution of palaeognaths. Data collected for other avian mitogenomes revealed that the last common ancestor of all birds and early diverging lineages of Neoaves could also possess the mitogenomic duplication.

**Conclusions:**

The duplicated control regions with adjacent genes are more common in avian mitochondrial genomes than it was previously thought. These two regions could increase effectiveness of replication and transcription as well as the number of replicating mitogenomes per organelle. In consequence, energy production by mitochondria may be also more efficient. However, further physiological and molecular analyses are necessary to assess the potential selective advantages of the mitogenome duplications.

**Supplementary Information:**

The online version contains supplementary material available at 10.1186/s12864-020-07284-5.

## Background

**Fig. 1:**
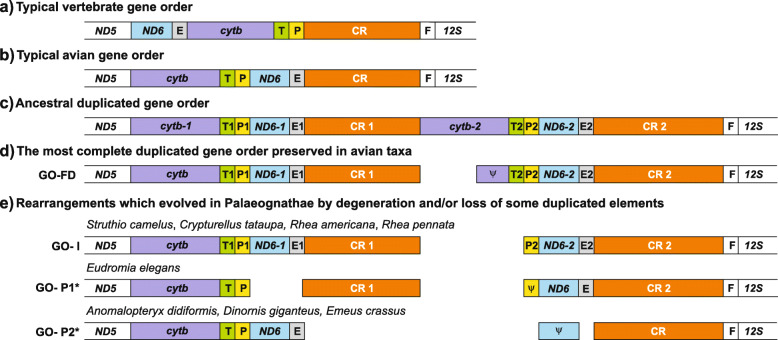
The comparison of various mitochondrial gene orders between *ND5* and *12S rRNA*: a typical vertebrate gene order (**a**), a typical avian gene order (**b**), an ancestral duplicated gene order assuming the tandem duplication of segment from *cytb* to CR (**c**), the most fully duplicated avian gene order, which was found in representatives of Bucerotiformes, Gruiformes, Procellariiformes, Psittaciformes and Suliformes (**d**), rearrangements that evolved by degeneration and/or loss of some duplicated elements in Palaeognathae and some Passeriformes: *Notiomystis cincta* and *Turdus philomelos* (**e**). *ND5* – gene for NADH dehydrogenase subunit 5; *cytb* – gene for cytochrome b; T – gene for tRNA-Thr; P – gene for tRNA-Pro; *ND6* – gene for NADH dehydrogenase subunit 6; E – gene for tRNA-Glu; CR – control region; F – gene for tRNA-Phe; 12S – gene for 12S rRNA. Pseudogenes are marked by ψ and colored correspondingly to their functional gene copy. Gene orders reannotated in this study are marked with an asterisk

Animal mitochondrial genomes are characterized by compact organization and almost invariable gene content, so any changes in them are especially interesting because they can be associated with major transitions in animal evolution [1, 2]. The first fully sequenced avian mitogenome from chicken *Gallus gallus* [3] turned out to contain single versions of 37 genes and one control region (CR) as in most other vertebrates, but organized in a different order (Fig. 1). This rearrangement is believed to have derived from the typical vertebrate gene order by a single tandem duplication of the fragment located between *ND5* and *tRNA-Phe* genes followed by random losses of one copy of duplicated items. Due to the prevalence of the *Gallus gallus* gene order in other birds, this rearrangement is generally believed to be an ancestral state for all Aves. In consequence, it is called common, standard or typical.

However, the growing number of avian mitochondrial genomes sequenced in recent years has revealed that other gene orders may also be present in a frequency higher than it was previously thought. To date, several distinct variations of mitochondrial rearrangements have been reported in a lot of representatives of many avian orders: Accipitriformes [4, 5], Bucerotiformes [6], Charadriiformes [7], Coraciiformes [8], Cuculiformes [9–11], Falconiformes [4], Gruiformes [12], Passeriformes [13, 14], Pelecaniformes [4, 15, 16], Phoenicopteriformes [17, 18], Piciformes [4, 19], Procellariiformes [20, 21], Psittaciformes [22, 23], Strigiformes [24], Suliformes [15, 20, 25] and Tinamiformes [26]. All these rearrangements include an additional region between *ND5* and *tRNA-Phe* genes, which seems to be particularly susceptible to duplication.

The most fully duplicated region (GO-FD; Fig. 1) was found in mitogenomes of all representatives of Gruidae [12] and Suliformes [15, 20, 25, 27], the majority of Pelecaniformes [4, 16] and Procellariiformes [4, 21, 28], as well as some Bucerotiformes and Psittaciformes species [6, 23]. All other avian gene orders containing the duplicated elements result from subsequent degenerations of GO-FD due to pseudogenization or loss of selected genes and/or the control region [22, 23].

It has been commonly assumed that the mitogenomic duplications are derived states and occurred independently in many Psittaciformes and Passeriformes lineages [13, 22, 29–31]. However, an independent origin of identical gene orders in different avian lineages seems unlikely because of the great number of possible arrangements [32–35]. More probable seems that the last common ancestor of many avian groups had a duplicated region. This feature was shown for Psittaciformes [23] and could be true for Accipitriformes [4, 5, 36–38], Falconiformes [4, 39], Gruidae [12] and Pelecaniformes [4, 15, 16], because all or almost all members of these groups contain mitogenomes with the duplicated regions. What is more, Mackiewicz, et al. [14] showed that even the last common ancestor of a larger monophyletic group of Aves including Psittaciformes, Passeriformes and Falconiformes could have had a duplication of the control region with adjacent genes in the mitochondrial genome.

The lack of duplication in some fully sequenced mitogenomes may be false and result from omission of identical repeats due to an inappropriate PCR strategy, insufficient sequencing methods or incorrect genome assembly. This problem was already addressed by Gibb, et al. [4], who found the fully duplicated gene order in *Thalassarche melanophris* mitogenome, which had been previously annotated without the duplication [40]. Similarly, two other mitogenomes of *Notiomystis cincta* and *Turdus philomelos* showed a novel duplicated gene order after a re-analysis [41], although previously the single version had been reported [42]. All re-amplified and re-sequenced crane mitogenomes also revealed the existence of duplication [12], which had not been found earlier [43]. Omitted duplications were also found within the mitochondrial genomes of Strigopoidea and Cacatuoidea, demonstrating that the ancestral parrot contained duplication in its mitogenome [23].

The growing number of formerly unidentified duplications implies that many avian mitogenomes published so far without duplication may, in fact, have it. Therefore, a diligent search for potential duplications is crucial in understanding the evolution of the avian mitogenome. Palaeognathae are particularly important to this subject because all comprehensive avian phylogenies have placed them as the sister group to the rest of birds, called Neognathae [44–48]. Palaeognaths comprise 25 genera and 82 species [49, 50], which are currently grouped into three extinct and five extant orders: the flighted Lithornithiformes known from Paleocene and Eocene of North America and Europe, and possibly from the Late Cretaceous; the flighted tinamous (Tinamiformes) from South and Central America; the flightless ratites containing the recently extinct New Zealand moas (Dinornithiformes) and Madagascan elephant birds (Aepyornithiformes) as well as the extant African ostrich (Struthioniformes), South American rheas (Rheiformes), Australian emu and Australasian cassowaries (Casuariiformes), and New Zealand kiwi (Apterygiformes). Phylogenetic relationships between these groups have been controversial. Molecular analyses have revealed that the ratites are paraphyletic and suggested that flightlessness evolved several times among ratites independently [51–60].

So far, a duplicated region (*cytb*/*tRNA-Thr*/*tRNA-Pro*/CR1/*ND6*/*tRNA-Glu*/CR2) has been found only in one representative of palaeognaths, namely *Eudromia elegans* [26]. This rearrangement has not been identified in any other avian species. Other Palaeognathae mitogenomes have a typical single avian gene order or were published as incomplete, especially in the part adjacent to the control region [26]. However, it cannot be ruled out that an inadequate PCR strategy was unable to amplify identical repeats or even prevented the completion of the mitogenome sequencing and assembly due to the presence of repeats [61]. Therefore, we applied another PCR strategy that allows the amplification of the fragment between two control regions including a potentially omitted duplication in representatives of *Struthio*, *Rhea*, *Casuarius*, *Dromaius* and *Crypturellus*. The new data help to elucidate the evolution of the Palaeognathae mitogenome in terms of duplication events, and also have implications for mitogenomic evolution in Aves as a whole.

## Results and discussion

### Duplicated gene order identified in mitogenomes of analyzed Palaeognathae taxa

**Fig. 2:**
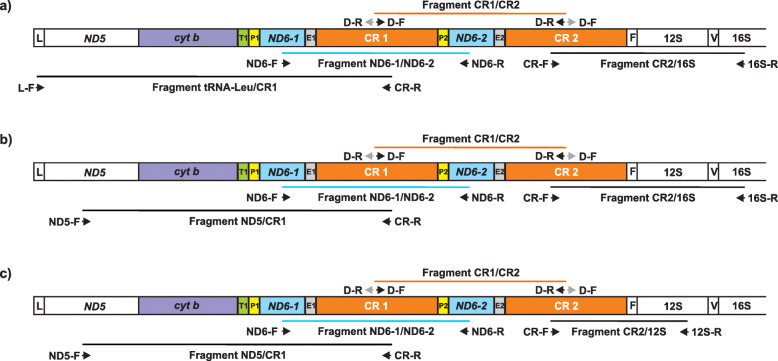
Strategy used in this study for identification of gene orders within duplicated regions in palaeognaths: *Struthio camelus* (**a**), *Rhea americana* and *Rhea pennata* (**b**) and *Crypturellus tataupa* (**c**) mitogenomes. L – gene for tRNA-Leu, *ND5* – gene for NADH dehydrogenase subunit 5, *cytb* – gene for cytochrome b, T – gene for tRNA-Thr, P – gene for tRNA-Pro, *ND6* – gene for NADH dehydrogenase subunit 6, E – gene for tRNA-Glu, CR – control region, F – gene for tRNA-Phe, 12S – gene for 12S rRNA, V – gene for tRNA-Val, 16S – gene for 16S rRNA. L-F, ND5-F, CR-R, ND6-F, ND6-R, D-F, D-R, CR-F, 12S-R, 16S-R: primers that were used for amplification of four overlapping mitogenomic fragments

Using an appropriate PCR strategy (Fig. 2), the diagnostic fragments ranges from the first (CR1) and the second control regions (CR2) were obtained for *Struthio camelus* (Fig. S1a in Additional file 1), *Rhea pennata* (Fig. S1b in Additional file 1), *Rhea americana* (Fig. S1c in Additional file 1) and *Crypturellus tataupa* (Fig. S1d in Additional file 1). Only two out of 16 or 48 reactions failed in the taxa for which species-specific primers were designed based on the previously published sequences of complete mitogenomes (*Struthio camelus* and *Rhea* species) (Table S1 in Additional file 2). In the case of *Crypturellus tataupa*, amplicons were obtained only for six out of 12 tested reactions. This was caused by the fact that primers dedicated for this species were designed on the sequence of more distant mitogenome from *Eudromia elegans* [26]. Similar to the published *Crypturellus tataupa* genomic sequence [62], the control region and adjacent genes were missing. Sequencing and annotation of the produced amplicons revealed the presence of *tRNA-Pro*/*ND6*/*tRNA-Glu* fragments between two control regions for *Struthio camelus*, *Rhea pennata*, *Rhea americana* and *Crypturellus tataupa* (Fig. 1). The duplicated fragment obtained for *Struthio camelus* differed only in one nucleotide from the homologous region in the previously published mitogenome (Fig. S2a in Additional file 1). These fragments in rheas showed 100% identity with corresponding homologous regions (Fig. S2b and Fig. S2c in Additional file 1).

Although the high identity strongly indicates a mitochondrial origin of the amplified CR1/CR2 fragments, additional diagnostic reactions were designed to exclude a possibility of nuclear mitochondrial DNA inserts (NUMTs) amplification. Based on the obtained sequences of *ND6* genes, appropriate primers were created to amplify *ND6–1*/*ND6–2* regions. Sequencing of the amplified PCR products revealed the *ND6*/*tRNA-Glu*/CR/*tRNA-Pro*/*ND6* gene order for all analyzed species. The corresponding CR/*tRNA-Pro*/*ND6* regions overlapped the appropriate CR1/CR2 diagnostic fragments and showed 100% identity. Additional PCR reactions (see Methods and Fig. 2) were run to complete the missing parts of CRs and to reveal the order of genes preceding the first control region. Finally, the complete mitogenomic fragments containing the duplicated regions were obtained by assembling four overlapping fragments (Fig. 2). Their length was: 8554 bp for *Struthio camelus*, 8254 for *Rhea Americana*, 8360 bp for *Rhea paennata* and 7044 bp for *Crypturellus tataupa* (Table 1; Fig. S3 in Additional file 1). In all cases the same gene order was found (GO-I; Fig. 1e, Table 1, Fig. S3 in Additional file 1), which was previously annotated only for two Passeriformes species, *Notiomystis cincta* and *Turdus philomelos* [41]. This gene rearrangement differs from the most complete known avian duplication (GO-FD; Fig. 1d) in the lack of the second copies of *cytb* and *tRNA-Thr* genes, expected between CR1 and *tRNA-Pro2* gene. The presence of identical copies of *tRNA-Glu* gene (Fig. S2a-d in Additional file 1) enabled us to position precisely the 5′ ends of both control regions. The 3′ ends of CR2s precede *tRNA-Phe* genes as in all other gene orders including two potentially functional control regions. The number of nucleotides between the *tRNA-Glu* copies and appropriate poly-C sequences located at the 5′ ends of CRs vary from 2 bp (*Rhea americana*, *Rhea pennata* and *Crypturellus tataupa*) to 26 bp for *Struthio camelus* (Table S2 in Additional file 2). The CR2 in *Rhea pennata* and *Crypturellus tataupa* is longer than CR1, which obey the rule observed in 13 crane species [12]. The tandem duplications found in the mitogenomes of *Struthio camelus*, *Rhea americana*, *Rhea pennata* and *Crypturellus tataupa* make them longer compared with their previous genomic versions assuming the typical avian gene order.

**Table 1:** Avian species analyzed in this study in terms of duplicated regions as well as gene orders found within their mitogenomic fragments, which were amplified and sequenced. The sequences are presented in Fig. S3 and S10

Order	Species	Sample type	Source^**1**^	Accession number	Length (bp)	Fragment^**2**^
Casuariiformes	*Casuarius casuarius*	Blood	ZOO WAW	–−	–−	–−
Casuariiformes	*Dromaius novaehollandiae*	Blood	ZOO WAW	–−	–−	–−
Rheiformes	*Rhea americana*	Blood	ZOO KAT	MK696563	8254	*ND5*/*cytb*/T/P1/*ND6–1*/E1/CR1/P2/*ND6–2*/E2/CR2/F/12S/V/16S
Rheiformes	*Rhea pennata*	Blood	ZOO WAW	MK696564	8306	*ND5*/*cytb*/T/P1/*ND6–1*/E1/CR1/P2/*ND6–2*/E2/CR2/F/12S/V/16S
Struthioniformes	*Struthio camelus*	Blood	ZOO WRO	MH264503	8554	L/*ND5*/*cytb*/T/P1/*ND6–1*/E1/CR1/P2/*ND6–2*/E2/CR2/F/12S/V/16S
Tinamiformes	*Crypturellus tataupa*	Blood	ZOO WAW	MK696562	7044	*ND5*/*cytb*/T/P1/*ND6–1*/E1/CR1/P2/*ND6–2*/E2/CR2/F/12S
Galliformes	*Chrysolophus pictus*	Blood	DPB UPWR	MW151829	1881	CR1/F/ Ψ12S/Ψ*ND6*/E/CR2
Caprimulgiformes	*Apus apus*	Blood	ORZ K	MW151827	2003	CR1/Ψ*cytb*/T/P/*ND6*/E/CR2
Cathartiformes	*Cathartes aura*	Blood	ZOO GDA	MN629891	7969	*ND5*/*cytb*/T1/P1/*ND6–1*/E1/CR1/Ψ*cytb*/T2/P2/*ND6–2*/E2/CR2/F/12S
Charadriiformes	*Alca torda*	Muscle	DVEZ UG	MK263222	2251	CR1/Ψ*cytb*/T/P/*ND6*/E/CR2
Charadriiformes	*Uria aalge*	Muscle	DVEZ UG	MK263188	2261	CR1/Ψ*cytb*/T/P/*ND6*/E/CR2
Ciconiiformes	*Ciconia nigra*	Blood	ZOO WAW	MH264509	3058	CR1/Ψ*cytb*/T/P/*ND6*/E/CR2
Eurypygiformes	*Eurypyga helias*	Blood	ZOO WAW	MW208859	7473	*cytb*/T/P/*ND6*/E/CR1/ … 3’rCR2/F/12S
Eurypygiformes	*Rhynochetos jubatus*	Feathers	BAP	–−	–−	–−
Gaviiformes	*Gavia arctica*	Muscle	DVEZ UG	MK263210	6598	*cytb*/T1/P1/*ND6–1*/E1/CR1/Ψ*cytb*/T2/P2/*ND6–2*/E2/CR2/F/12S
Gaviiformes	*Gavia stellata*	Muscle	DVEZ UG	MK263209	7539	*cytb*/T1/P1/*ND6–1*/E1/CR1/Ψ*cytb*/T2/P2/*ND6–2*/E2/CR2/F/12S
Musophagiformes	*Corythaixoides personatus*	Blood	Poland, captive	MW082596	2002	CR1/Ψ*cytb*/T/P/*ND6*/E/CR2
Pelecaniformes	*Scopus umbretta*	Blood	ZOO WRO	MW151828	1632	CR1/P/*ND6*/E/CR2
Podicipediformes	*Podiceps cristatus*	Muscle	DVEZ UG	MN629890	5171	*cytb*/T1/P1/*ND6–1*/E1/CR1/Ψ*cytb*/T2/P2/*ND6–2*/E2/CR2
Podicipediformes	*Podiceps grisegena*	Muscle	DVEZ UG	MK263194	4061	*ND6–1*/E1/CR1/Ψ*cytb*/T2/P2/*ND6–2*/E2/CR2
Sphenisciformes	*Spheniscus demersus*	Blood	ZOO WRO	MH264510	3032	CR1/Ψ*cytb*/T/P/*ND6*/E/CR2
Trogoniformes	*Trogon collaris*	Feathers	WBF	–−	–−	from *cytb* to 12S - not sequenced; from CR1 to CR2 - not sequenced

^1^*ZOO GDA* Zoological Garden in Gdańsk; *DPB UPWR* Department of Poultry Breeding at Wrocław University of Environmental and Life Sciences; *ORZ K* Animal Rehabilitation Center in Kątna; *DVEZ UG* Department of Vertebrate Ecology and Zoology at University of Gdańsk; *BAP* Berry Avicultural Park in Italy; *WBF* World of Birds Foundation in the Netherlands

^2^*L* gene for tRNA-Leu, *ND5* Gene for NADH dehydrogenase subunit 5, *cytb* Gene for cytochrome b, *T* Gene for tRNA-Thr, *P* Gene for tRNA-Pro, *ND6* Gene for NADH dehydrogenase subunit 6, *E* Gene for tRNA-Glu, *CR* Control region, *rCR* Remnant control region, *F* Gene for tRNA-Phe, *12S* Gene for 12S rRNA, *V* Gene for tRNA-Val, *16S* Gene for 16S rRNA

### Probable presence of mitochondrial CR1/CR2 fragments in *Casuarius casuarius* and *Dromaius novaehollandiae* nuclear genomes

In the case of two other Palaeognathae species, *Casuarius casuarius* and *Dromaius novaehollandiae*, an attempt to amplify the CR1/CR2 fragment was also made. Similar to other taxa, species-specific D-F and D-R primers (Fig. 2; Table S1 in Additional file 2) were designed using the sequences of previously published complete mitogenomes (AF338713.2 and AF338711.1). In contrast to the results obtained for the other Palaeognathae species, most PCR reactions failed to amplify the expected fragments. In *Dromaius novaehollandiae*, amplicons were obtained only for 3 out of 25 tested reactions (Fig. S4a in Additional file 1, Table S1 in Additional file 2). Analogously, PCR products were obtained only for 4 out of 56 reactions for *Casuarius casuarius* (Fig. S4b in Additional file 1, Table S1 in Additional file 2). Moreover, single DNA fragments were not produced for any of these seven reactions, although different annealing temperatures were applied (Fig. S4 in Additional file 1). Taking into account the heterogeneity of the obtained DNA fragments as well as the fact that most of the tested reactions failed, we can conclude that the PCR products presented in Fig. S4 in Additional file 1 were not amplified on the mitochondrial genome template. The D-F and D-R primers as well as the applied PCRs are highly specific and diagnostic for the presence of CR duplication in parrots [23], cranes [12] as well as black-browed albatross, ivory-billed aracari and osprey [4]. Therefore, the seven positive amplicons most likely represent mitochondrial DNA fragments located in the nuclear genomes, i.e. NUMTs. It means that *Casuarius casuarius* and *Dromaius novaehollandiae* or their ancestors had mitogenomes comprising two control regions, which were transferred into the nucleus during evolution.

### Reannotation of *Eudromia elegans* mitochondrial gene order

The GO-I gene order (Fig. 1) found in this study for four Palaeognathae taxa differs from that in the published mitogenomic sequence of *Eudromia elegans* [26]. This rearrangement appears to be a degenerated form of GO-I as it lacks the first copy of *ND6* and *tRNA-Glu* genes as well as the second copy of *tRNA-Pro* gene. This fact prompted us to search for a potential *tRNA-Pro* pseudogene hidden within the last 122 nucleotides of the first control region of *Eudromia elegans* mitogenome. In fact, the comparison of CR1 sequence with the potentially functional *tRNA-Pro* sequence of this species revealed a significant similarity (E-value = 1.2·10^− 6^; 81% identity without gaps and 64% including gaps) between these sequences along the 84-bp alignment (Fig. S5a in Additional file 1), which suggests the presence of the *tRNA-Pro* pseudogene in the *Eudromia* mitogenome in the position between 16,272 bp and 16,349 bp. After reannotation of this pseudogene, the length of CR1 reduced to 1352 bp. The newly annotated *Eudromia* gene order was defined as GO-P1 in Fig. 1e.

### Reannotation of mitochondrial gene order in the mitogenomes of Anomalopteryx didiformis, Emeus crassus and Dinornis giganteus

Our analysis of 5′ spacers, i.e. fragments of control regions located between the *tRNA-Glu* gene and poly-C motif, revealed that they are much longer in annotated *Anomalopteryx didiformis*, *Emeus crassus* and *Dinornis giganteus* mitogenomes than in other Palaeognathae species. These spacers of the most Palaeognathae taxa are from 2 bp to 33 bp in length (Table S2 in Additional file 2), but in *Anomalopteryx didiformis*, *Emeus crassus* and *Dinornis giganteus*, they are longer, i.e. 133 bp, 157 bp and 150 bp, respectively. Additionally, all three fragments contain a purine-rich insertion (Fig. S5b in Additional file 1) analogous to that in parrot *ND6* pseudogenes (Fig. S5c in Additional file 1) [23]. In the Psittaciformes mitogenomes (*Probosciger aterrimus goliath*, *Eolophus roseicapilla* and *Cacatua moluccensis*), this insertion is preceded by a fragment (with 433–450 bp) almost identical with the first *ND6* copy followed by a highly degenerated region. This similar sequence pattern prompted us to search for potential *ND6* pseudogenes within the 5′ spacers of CRs in *Anomalopteryx didiformis*, *Emeus crassus* and *Dinornis giganteus*. The comparison of 5′ CR sequences with appropriate *ND6* genes of these species revealed a significant similarity between the aligned sequences (Table S3 in Additional file 2). Those from *Anomalopteryx didiformis* were identical in 71% with E-value = 0.13 (Fig. S5d in Additional file 1) and from *Emeus crassus* in 73% with E-value = 0.0015 (Fig. S5e in Additional file 1). The alignment of *Dinornis giganteus* sequences was much more significant with E-value = 5.8·10^− 106^ and the sequences showed 83% identity (Fig. S5f in Additional file 1). The obtained identity and E values are in the range of those obtained for *ND6* pseudogenes and their functional copies annotated in other avian species, i.e. 65–96% and 0–0.23, respectively (Table S3 in Additional file 2).

Assuming the presence of *ND6* pseudogenes in *Anomalopteryx didiformis*, *Dinornis giganteus* and *Emeus crassus* mitogenomes, the length of their CR is reduced to 1347 bp, 1360 bp and 1346 bp, respectively. The CR sequences show 71–81% identity at 5′ spacers on the length 165 bp (Fig. S5b in Additional file 1). The new avian gene order present in these reannotated mitogenomes is indicated as GO-P2 in Fig. 1e.

### Comparison of the duplicated regions of Palaeognathae mitogenomes

The GO-I gene order found in four Palaeognathae species (Fig. 1, Table 2) is characterized generally by a high similarity between paralogous sequences, i.e. copies found within the same mitogenome. The second copies of *tRNA-Pro*, *ND6* and *tRNA-Glu* are identical with the first ones in the case of *Struthio camelus*, *Rhea americana* and *Rhea pennata* (Table 3). The second copy of *tRNA-Glu* is also identical with the first one in *Crypturellus tataupa* mitogenome. However, the first copies of *tRNA-Pro* and *ND6* genes of this species differ from their paralogous sequences in three nucleotides (Table 3). Two control regions of analyzed species show a slightly greater variation in identity, from 94.4% (*Rhea pennata*) to 97.8% (*Crypturellus tataupa*). The difference is mainly located at their 3′ ends, except for *Rhea* taxa, whose control regions differ also at their 5′ ends (Fig. S2 in Additional file 1). The high similarity of duplicated regions indicates that they evolved in concert, which homogenized their sequences as found in many other avian groups [4, 6, 14, 23, 25, 28, 30, 63–70].

**Table 2:** Avian mitochondrial genomes analyzed in this study. GO-I, GO-P1 and GO-P2 indicate gene orders with the duplicated region. GO-TA means the typical avian gene order without duplication

Order	Species	Accession	Length [bp]	Gene Order
Struthioniformes	*Struthio camelus*	AF338715.1	16,595	GO-I
Rheiformes	*Rhea americana*	AF090339.1	16,704	GO-I
Rheiformes	*Rhea pennata*	AF338709.2	16,749	GO-I
Casuariiformes	*Casuarius casuarius*	AF338713.2	16,756	GO-TA
Casuariiformes	*Casuarius bennetti*	AY016011.1*	12,348	?
Casuariiformes	*Dromaius novaehollandiae*	AF338711.1	16,711	GO-TA
Aepyornithiformes	*Aepyornis sp.*	KY412176.1	16,688	GO-TA
Aepyornithiformes	*Aepyornis hildebrandti*	KJ749824.1*	15,547	?
Aepyornithiformes	*Mullerornis agilis*	KJ749825.1*	15,731	?
Apterygiformes	*Apteryx mantelli*	KU695537.1	16,694	GO-TA
Apterygiformes	*Apteryx owenii*	GU071052.1	17,020	GO-TA
Apterygiformes	*Apteryx haastii*	AF338708.2	16,980	GO-TA
Tinamiformes	*Crypturellus tataupa*	AY016012.1*	12,205	GO-I
Tinamiformes	*Eudromia elegans*	AF338710.2	18,305	GO-P1
Tinamiformes	*Tinamus guttatus*	KR149454.1	16,750	GO-TA
Tinamiformes	*Tinamus major*	AF338707.3	16,701	GO-TA
Dinornithiformes	*Anomalopteryx didiformis*	AF338714.1*	16,716	?
Dinornithiformes	*Anomalopteryx didiformis*	MK778441.1	17,043	GO-P2
Dinornithiformes	*Emeus crassus*	AF338712.1*	16,662	?
Dinornithiformes	*Emeus crassus*	AY016015.1	17,061	GO-P2
Dinornithiformes	*Dinornis giganteus*	AY016013.1	17,070	GO-P2

*indicates incomplete mitogenomes

?means an unknown gene order

**Table 3:** Comparison of two copies of selected genes as well as control regions in mitogenomes from five Palaeognathae taxa

Species	Copy	Length (bp)				Percent of residues identical between two copies and number of aligned residues (in parentheses)			
		***tRNA-Pro***	***ND6***	***tRNA-Glu***	CR	***tRNA-Pro***	***ND6***	***tRNA-Glu***	CR
*Struthio camelus*	1st	70	522	68	1035	100 (70)	100 (522)	100 (68)	96.9 (1023)
	2nd	70	522	68	1036				
*Rhea americana*	1st	70	525	69	1118	100 (70)	100 (525)	100 (69)	94.4 (1076)
	2nd	70	525	69	1118				
*Rhea pennata*	1st	70	525	69	1103	100 (70)	100 (525)	100 (69)	94.1 (1076)
	2nd	70	525	69	1183				
*Crypturellus tataupa*	1st	70	522	69	1059	95.7 (70)	99.4 (522)	100 (69)	97.8 (1059)
	2nd	70	522	69	1196				
*Eudromia elegans*	1st	73	–	–	1352	80.6 (84)	–	–	98.2 (1252)
	2nd	78 (ψ)	522	70	1350				

In contrast to GO-I gene order, the newly defined rearrangement GO-P1 in *Eudromia elegans* is characterized by single versions of *ND6* and *tRNA-Glu* gene (Fig. 1). Moreover, the second copy of *tRNA-Pro* is a pseudogene, which has substantially diverged from its full version (Fig. S5a in Additional file 1). Therefore, it seems that the GO-P1 rearrangement is a degenerated form of GO-I, in which two genes were removed and one gene was pseudogenized. Surprisingly, despite the high degree of degeneration in comparison with other analyzed Palaeognathae species, two control regions of *Eudromia elegans* maintain the highest sequence identity (Table 3), although the alignment of these regions clearly shows the presence of several deletions/insertions (Fig. S6 in Additional file 1).

The comparison of paralogous control regions in Palaeognathae revealed that CR2s are much longer only in two species, i.e. *Rhea pennata* and *Crypturellus tataupa* (Table 3). Such a difference in the length of CRs seems to be a rule in most avian mitogenomes with a duplicated region [23]. Interestingly, CRs in *Rhea americana* are identical in length, while those in *Struthio camelus* and *Eudromia elegans* differ only in one and two nucleotides, respectively (Table 3).

### Phylogenetic relationships within Palaeognathae based on mitogenomes

**Fig. 3:**
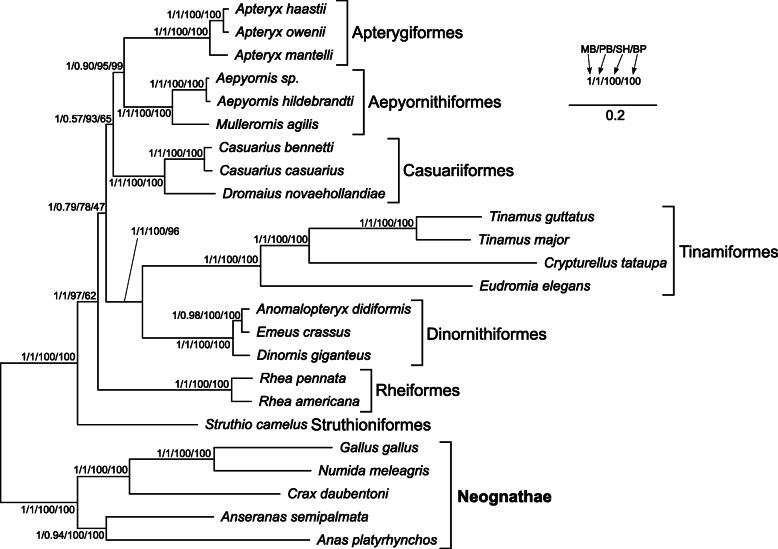
The phylogram obtained in MrBayes based on nucleotide sequences of mitochondrial genes. The values at nodes, in the following order MB/PB/SH/BP, indicate: posterior probabilities found in MrBayes (MB) and PhyloBayes (PB) as well as SH-aLRT (SH) and non-parametric bootstrap (BP) percentages calculated in IQ-TREE

Three phylogenetic methods applied for the mitogenomic sequences resulted in a consistent topology (Fig. 3). The earliest diverging lineage of Palaeognathae was *Struthio camelus* (representing Struthioniformes) and next, Rheiformes (Rheidae) diverged. Dinornithiformes (Dinornithidae + Emeidae) is grouped with Tinamiformes (Tinamidae), whereas Casuariiformes (Dromaiidae + Casuariidae) is sister to Aepyornithiformes (Aepyornithidae) + Apterygiformes (Apterygidae). Almost all nodes are very well supported. The least significant are two nodes: one clustering Casuariiformes, Aepyornithiformes and Apterygiformes, and the other encompassing the palaeognath lineages separated after the divergence of *Struthio* and *Rhea*. Nevertheless, these two nodes obtained the highest posterior probability in MrBayes analysis, i.e. 1.0 and support in the Shimodara-Hasegawa-like approximate likelihood ratio test (SH-aLRT) equal to 93 and 78, respectively.

**Fig. 4:**
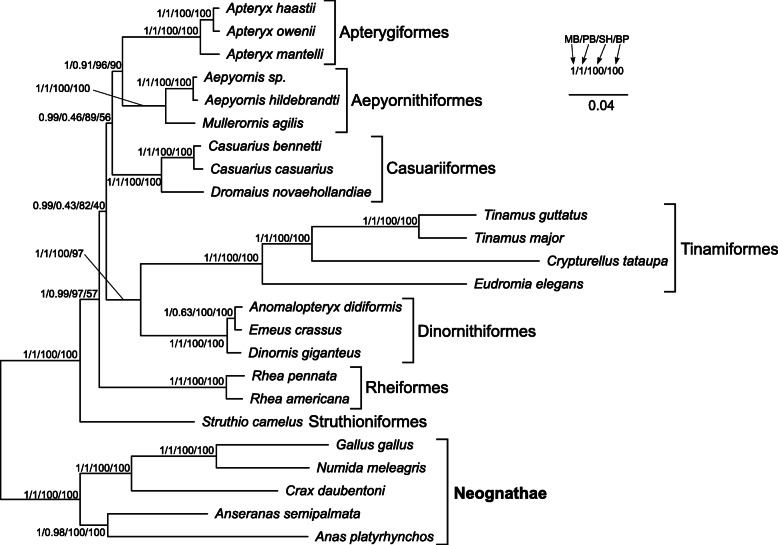
The phylogram obtained in MrBayes based on RY-recoded sequences of mitochondrial genes. See Fig. 3 for further explanations

In order to eliminate a potential artefact related with the compositional heterogeneity in the third codon positions of protein-coding genes, we created phylogenetic trees based on the RY-coding alignment (Fig. 4). The tree topology produced by the three methods was the same as that for the uncoded alignment. The posterior probability of the two controversial nodes was still very high in MrBayes tree, i.e. 0.99 and the SH-aLRT support was 89 and 82, respectively.

**Fig. 5:**
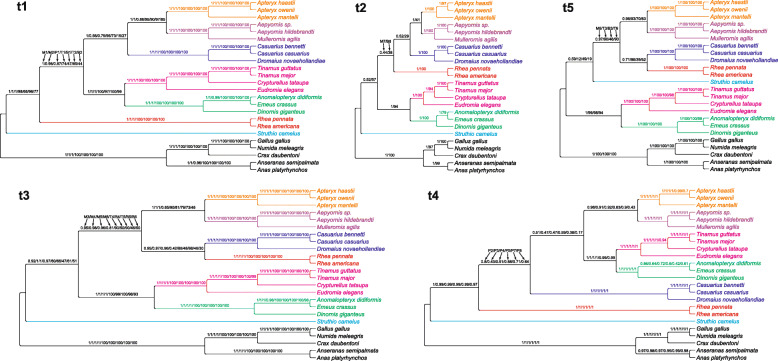
The most frequent tree topologies obtained in the phylogenetic analyses of mitochondrial gene alignments. Partitions characterized by the highest substitution rate were sequentially excluded from the alignment. The values at nodes indicate support values received for various partitions in different approaches. The approaches’ names were marked with the letter: MrBayes with M, PhyloBayes with P, SH-aLRT in IQ-TREE with T and non-parametric bootstrap in IQ-TREE with B. The digits after these letters indicate the number of the highest rate partitions removed from the analysis

Moreover, we performed phylogenetic analyses based on ten alignments, from which we sequentially excluded partitions characterized by the highest substitution rate (Table S4 in Additional file 2). The calculations produced in total 16 topologies, out of which five are worthy of mention because they were obtained by many independent approaches (Fig. 5). The topology t1 was identical with that based on the alignments including all sites and demonstrated rheas as sister to all other non-ostrich palaeognaths. Such a tree was produced by MrBayes, PhyloBayes and IQ-TREE using the alignment without sites characterized by the highest substitution rate, as well as by MrBayes and IQ-TREE using the alignment after removing sites with two highest rate categories. The posterior probabilities for the clade including palaeognaths other than ostrich and rheas were very high in MrBayes, i.e. 1 and 0.98, respectively, or moderate, i.e. 0.87 in PhyloBayes. In the topology t2, the *Rhea* clade was grouped with Casuariiformes + Apterygiformes. However, the support of this grouping was very weak and occurred only in MrBayes tree and IQ-TREE consensus bootstrap tree based on the alignments without seven and eight highest rate categories, respectively. A greater Bayesian support (0.95–0.97) was obtained by the node encompassing rheas with Casuariiformes in the topology t3 based on the alignments after removing three, four and five highest rate categories. This topology was also produced in MrBayes using the alignment without eight highest rate categories and in IQ-TREE for the alignments without four, five and six highest rate categories. However, the node support was generally weak. The topology t4 was produced only by PhyloBayes for the alignments without two, three, four, five, seven and eight highest rate categories. As in the topology t1, the *Rhea* clade was also sister to all other palaeognaths excluding *Struthio*, but Casuariiformes were clustered with the rest non-ostrich palaeognaths, not directly with Aepyornithiformes and Apterygiformes. The posterior probability values of the clade including palaeognaths sister to rheas did not exceed 0.8. The topology t5 differed from the others because *Struthio camelus* was placed within other Palaeognathae and the external position was occupied by Dinornithiformes + Tinamiformes, whereas *Rhea* was grouped with Casuariiformes. This topology was obtained for the alignments without three (in IQ-TREE) and six highest rate categories (in MrBayes and IQ-TREE). Nevertheless, the controversial nodes were poorly supported.

Removing the sites with the highest substitution rate eliminated the alignment positions that were saturated with substitutions, but the number of parsimony informative sites decreased, too (Fig. S7a). Therefore, the stochastic error could increase for the short alignments and the inferred phylogenetic relationships could be unreliable. After elimination of sites with two highest rate categories, the mean phylogenetic distance in the MrBayes tree decreased abruptly from 0.94 to 0.33 substitutions per site and the maximum distance in the tree dropped from 1.99 to 0.69 substitutions per site (Fig. S7a). The sharp decrease was also visible in the number of informative sites, which constituted 56% of those in the original alignment. However, the sisterhood of rheas to other non-ostrich palaeognaths was still present in the trees based on the purged alignments and the latter group was relatively highly supported (Fig. S7b). After removing sites with at least three highest rate categories, the alignment was deprived of more than half of informative sites and alternative topologies were favored, though with smaller support values (Fig. S7b).

Among the applied topology tests, the BIC approximation produced all Bayesian posterior probabilities for the alternative topologies much smaller than 0.05 indicating a strong rejection of the tested alternatives in favor of topology t1 (Table S5 in Additional file 2). Moreover, the topology t4 performed significantly worse than t1 in two bootstrap tests, whereas the bootstrap probabilities for the topology t2 were 0.063, i.e. very close to the 0.05 threshold. Other tests did not reject the alternative topologies. However, Bayes factor was greater than 9 indicating an overwhelming support for the topology t1 because the commonly assumed threshold for such interpretation is 5 [71].

### Comparison of Palaeognathae tree topologies

All the phylogenetic analyses imply that the relationships presented in the topology t1 describe the most probable evolutionary history between the mitochondrial genomes of palaeognaths. Such relationships, but not always on the full taxa set, were also obtained in other studies based on mitochondrial genes [55, 56], selected nuclear genes [48, 54, 57], the joined set of nuclear and mitochondrial genes [46, 52, 58] as well as the concatenated alignments of many nuclear markers [45, 59, 60]. However, the application of a coalescent species tree approach on these markers and the analysis of retroelement distribution indicated a closer relationship between rheas and the clade of Casuariiformes + Apterygiformes [45, 59, 60]. This phylogeny was also generated for selected nuclear genes [53] and in a supertree approach [47]. These relationships are presented in the topology t2 but are, however, insignificant for the mitochondrial gene set. An alternative, poorly supported topology, in which *Rhea* clustered with Tinamiformes or Dinornithiformes + Tinamiformes, was also received for some nuclear genes [54, 57].

Thus, two topologies, t1 and t2, aspire to be the real species tree but it is not easy to evaluate which one is true. Although t1 was found in many studies based on concatenated alignments of many markers, it has been criticized as a true species tree in favor of t2, which was obtained in coalescent-based approaches also using huge data sets [59, 60]. The selection of one out these two topologies is complicated by the fact that the topology t2 is supported by 229 loci and is only the 7th in the ranking of the most common gene tree topologies, whereas the topology t1 is the 2nd, supported by 280 loci [59]. The largest number of markers, i.e. 357, indicated another topology, in which Rheiformes was grouped with the clade of Dinornithiformes + Tinamiformes. This discrepancy was explained by the existence of an empirical anomaly zone resulting from incomplete lineage sorting (ILS) across short internal branches leading to the last common ancestor of Rheiformes, Apterygiformes and Casuariiformes [59, 60].

Although coalescent species trees account for ILS, simulations showed that species tree methods based on the gene tree summation may not provide significantly better resilts than concatenation alignment methods, which can perform even better [72]. Prum, et al. [45], who applied extensive avian taxon sampling and loci with slow substitution rates, found no single locus that would be able to fully resolve the tree topology. They concluded that this lack of phylogenetic information can challenge the accuracy of a coalescent-based summary approach relative to concatenation. The multispecies coalescent models work under some conditions [73]. For example, they assume that incongruent gene trees are independently generated from a coalescence process occurring along the species tree and there is no selection on the studied genetic markers. Because Cloutier, et al. [60] and Sackton, et al. [59] analyzed conserved and ultraconserved noncoding nuclear elements, we cannot exclude that they are involved in essential regulatory functions and then are subjected to selection [74–76], which could influence the model assumptions.

Generally, nuclear markers are more prone to ILS and hidden gene paralogy [77–84] than mitochondrial genes, which are present in a haploid genome and maternally inherited [85]. Thus, the time needed to completely sort the ancestral polymorphism of mitochondrial DNA is on average four times smaller than for nuclear genes [86]. Introgression of mtDNA is another reason for the discrepancy between the gene and species trees. However, this process concerning maternally inherited mtDNA is restricted between heterogametic avian species because female hybrids are characterized by reduced viability [87–93]. In agreement with that, a survey of causes of mtDNA gene tree paraphyly in birds found that 8% of studied species had paraphyly attributable to incorrect taxonomy, and only 3% because of ILS or hybridization, 2% on account of ILS and 1% due to introgressive hybridization [94]. Moreover, the mitochondrial genes are located on one molecule and are inherited together [82, 95], so they should bear a consistent phylogenetic signal. Accordingly, analyses based on complete mitogenomes have provided well-resolved phylogenies of various avian groups [9, 14, 40–42, 96–99]. Nevertheless, it is not inconceivable that the ILS effect can influence mtDNA in rapidly radiating taxa, in which on-going speciation occurs before genetic sorting [100].

### Distribution of mitogenomic rearrangements in phylogenetic trees of Palaeognathae

**Fig. 6:**
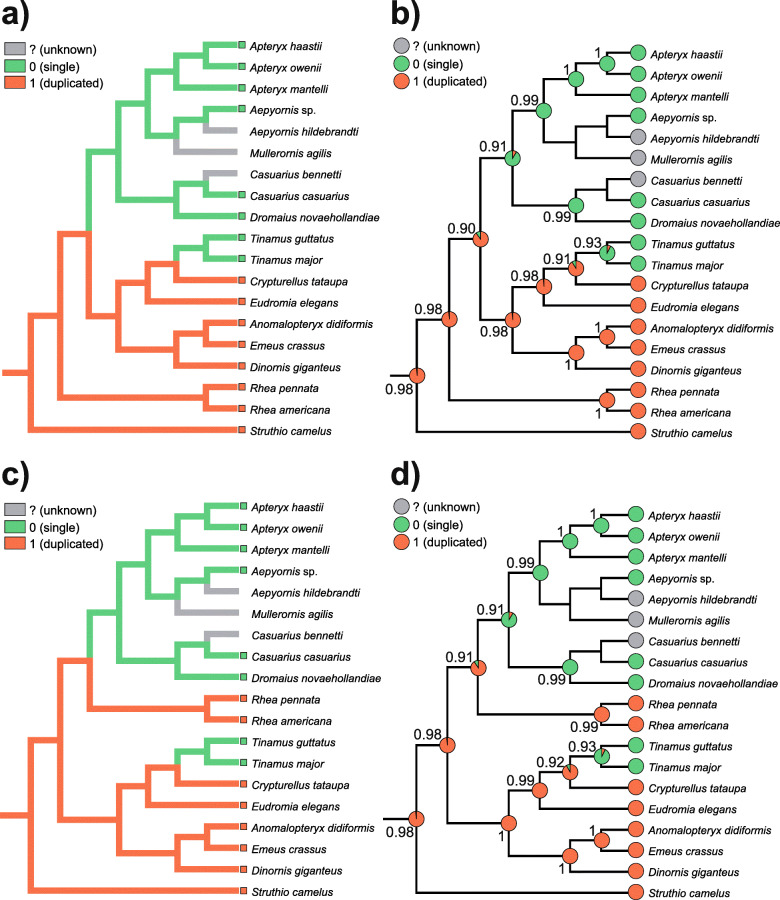
Reconstruction of ancestral states and mapping of mitogenomic duplications onto the Palaeognathae trees. Two methods, maximum parsimony (**a**, **c**) and maximum likelihood (**b**, **d**) were applied for two tree topologies: t1 based on sequences of mitochondrial genes (**a**, **b**) and t2 supported by nuclear markers in coalescent-based approaches (**c**, **d**). The area of colors at nodes in b and d corresponds to the probability of the given state, single or duplicated region. The probability value for a more likely state was also given at these nodes. Mk1 model was applied for ML approach

We considered both t1 and t2 topologies to analyze the presence and absence of the mitogenomic duplication in the phylogenetic context. Using these relationships, we mapped the mitogenomic features onto these topologies and inferred ancestral states for the individual lineages using maximum parsimony (MP) and maximum likelihood (ML) methods (Fig. 6).

Two methods applied to the topology t1 clearly indicated that the last common ancestor of palaeognaths contained a duplicated region in its mitogenome (Fig. 6 a and b). This state was inherited by the ostrich and rhea lineages. The ML method provided the probability *P* > 0.982 of this state for the last common ancestors of all palaeognaths and non-ostrich palaeognaths. The last common ancestor of the remaining groups, i.e. Dinornithiformes, Tinamiformes, Casuariiformes, Aepyornithiformes and Apterygiformes, could also contain a duplication with *P* = 0.899. However, the last common ancestor of Casuariiformes, Aepyornithiformes and Apterygiformes lost this duplication (*P* = 0.914). In turn, the last common ancestor of Dinornithiformes and Tinamiformes still had the duplicated region (*P* = 0.983), which was probably lost in *Tinamus*, whereas *Anomalopteryx*, *Dinornis* and *Emeus* maintained only a pseudogenized *ND6*.

According to the topology t2, the last common ancestors of all palaeognaths and non-ostrich palaeognaths also had the duplication in their mitogenomes with the probability of at least 0.982 (Fig. 6 c and d). The duplication was preserved in the last common ancestor of Dinornithiformes and Tinamiformes (*P* = 0.996) as well as the last common ancestor of Rheiformes, Casuariiformes, Aepyornithiformes and Apterygiformes (*P* = 0.906). Among four latter orders, only Rheiformes maintained the duplication, whereas the last common ancestor of the other orders had a mitogenome without the duplicated region (P = 0.914).

One could argue that the traces of *ND6* pseudogene in *Anomalopteryx*, *Dinornis* and *Emeus* are equivocal. Therefore, we conducted analyses in which we assumed that mitogenomes of these genera had already lost the duplication (Fig. S8 in Additional file 1). However, the general conclusion that the last common ancestor of all Palaeognathae had a mitogenome with a duplicated region did not change. The probability of this state was 0.916 and 0.829 for topology t1 and t2, respectively. The last common ancestor of non-ostrich palaeognaths also had this feature with *P* = 0.889 and 0.697 for these topologies, respectively.

**Fig. 7:**
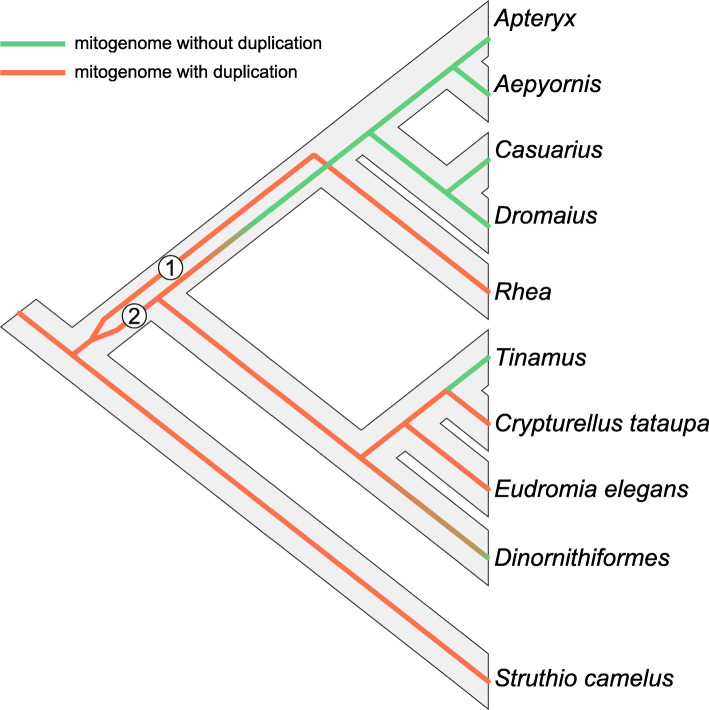
Superposition of the mitogenome phylogeny (thin colored lines) onto the potential species tree of Palaeognathae (grey thick branches). Two mitochondrial lineages were labelled as 1 and 2. Lineages with and without duplication were indicated in different colors. The tonal transition from orange to green indicates gradual disappearing of the duplication

The above-mentioned scenarios of palaeognath mitogenome evolution assume that the mitochondrial tree represents the true species tree. However, as discussed in the previous section, the topology t1 is highly supported by mitochondrial data, whereas t2 is backed up by nuclear markers in coalescent-based approaches and is regarded as the true species tree by some authors [59, 60]. Assuming that the t1 presents the real mitogenomic history, we superimposed the mitogenome phylogeny onto the potential species tree (Fig. 7). In order to reconcile the alternative positions of rheas in these topologies, we should assume that there existed heteroplasmy, i.e. at least two types of mitochondrial genomes, in the last common ancestor of non-ostrich palaeognaths. Both mitogenomes probably initially contained a duplication, as indicated by the inferred ancestral states. The lineages of these mitogenomes are marked in Fig. 7 as 1 and 2. Dinornithiformes and Tinamiformes inherited only mitogenome 2, whose duplicated regions had begun to fade and likely disappeared in *Tinamus*. However, the common ancestor of Rheiformes, Casuariiformes, Aepyornithiformes and Apterygiformes preserved two mitogenomes, but genome 2 lost the duplicated region during the course of evolution. Then, the mitochondrial lineages were segregated: mitogenome 1 with the duplication was left in rheas, whereas mitogenome 2 without the duplication was passed to Casuariiformes, Aepyornithiformes and Apterygiformes.

The assumption about the presence of heteroplasmy in some period of Palaeognathae evolution seems probable because such genomic diversity has also been reported in various avian groups: Accipitriformes [5], Bucerotiformes [6], Charadriiformes [7, 101, 102], Ciconiiformes [103], Columbiformes [104], Gruiformes [105], Passeriformes [106, 107], Pelecaniformes [16, 108], Piciformes [4], Procellariiformes [28], Psittaciformes [63], Sphenisciformes [109], Strigiformes [24] and Suliformes [25]. The period in which the ancestor carried two mitogenomes was likely very short. According to the results of molecular dating performed by Kimball, et al. [47], the time elapsed since the divergence of the clade Dinornithiformes+Tinamiformes and that including Rheiformes, Casuariiformes, Aepyornithiformes and Apterygiformes to the separation of Rheiformes from the three latter orders was only 3.6 million years.

The assumption of heteroplasmy is one of possible explanations. Alternatively, we can assume an introgression of mitochondrial DNA with a duplication to rheas from an extinct lineage that diverged after Struthioniformes lineage and before the clade of Dinornithiformes + Tinamiformes. However, this seems unlikely because the birds involved in this introgression must have had a long evolutionary history after the separation of their lineages, at least 9 up to 3.6 million years according to Kimball, et al. [47]. Thus, they would probably represent a separate species, which would protect them from hybridization. We would also have to assume that both the donor and the acceptor of mtDNA, i.e. rheas were still evolving in the same region, e.g. South America.

### Role of duplication in the evolution of palaeognaths

It was reported that two duplicated control regions can lead to a more effective initiation of replication or transcription and a greater number of replicating mitogenomes per organelle, which may increase energy production by mitochondria [65, 66, 110–112]. In support of this, it was found that parrots keeping two copies of control region in their mitogenomes show morphological features related to more energy-consuming active flight [23]. Following this finding, we can infer that the last common ancestor of palaeognaths was volant because mapping of mitogenomic features onto the phylogenetic trees showed that the ancestor most likely contained the duplicated regions (Fig. 6). This finding corresponds well to recently proposed scenarios for the evolutionary history of Palaeognathae, which may have originated in the Late Cretaceous in the Northern Hemisphere [58]. Around the Cretaceous-Paleogene boundary, they may have migrated to the Southern Hemisphere, where they dispersed widely and diversified. They extended their distribution by long-distance overseas dispersal to the Gondwana-derived landmasses, such as New Zealand and Madagascar [58]. These long-distance dispersals must have been accompanied by very good adaptations to active flight. However, modern palaeognath species are flightless or at most poor flyers, i.e. tinamous. Therefore, the maintenance of two control regions in some palaeognaths may be the legacy of their ancestors. Alternatively, these regions can still provide benefits even for flightless species during long-distance walking and running, which require a lot of energy.

### Implications for mitogenome evolution in all Aves

The finding that a palaeognath ancestor contained a mitogenomic duplication challenges the common assumption that this feature evolved independently in individual lineages of Neognathae, i.e. the sister group of Palaeognathae [13, 22, 29–31]. Increasing amount of data indicates that mitogenomes with a duplicated region are present in all or a vast majority of representatives of diverse Neognathae lineages, i.e. Accipitriformes [4, 5, 36–38], Falconiformes [4, 39, 68], Gruiformes [12], Pelecaniformes [4, 15, 16], Psittaciformes [23] and Suliformes [25]. It suggests that ancestors of these groups could have also possessed a mitogenomic duplication. The presence of this state was recently inferred for the last common ancestor of three closely related orders, Falconiformes, Passeriformes and Psittaciformes [14]. Therefore, it is interesting to consider if this feature was present much earlier in the evolution of birds or even in the last common ancestor of all known Aves.

In order to obtain data for this analysis, we surveyed as well as annotated and reannotated all avian mitogenomic sequences available in GenBank to identify cases of duplication (Table S6 in Additional file 2; Fig. S9 in Additional file 1). Moreover, we conducted appropriate PCR reactions checking the presence of duplicated fragments in representatives of selected bird orders, which were poorly represented in the database (Table 1). Most of PCR products were sequenced to determine the arrangement of genes located between two control regions (Table S9 in Additional file 2). The thorough analysis revealed many duplicated regions that were previously omitted.

#### Searching for duplicated regions in avian mitogenomes

**Fig. 8:**
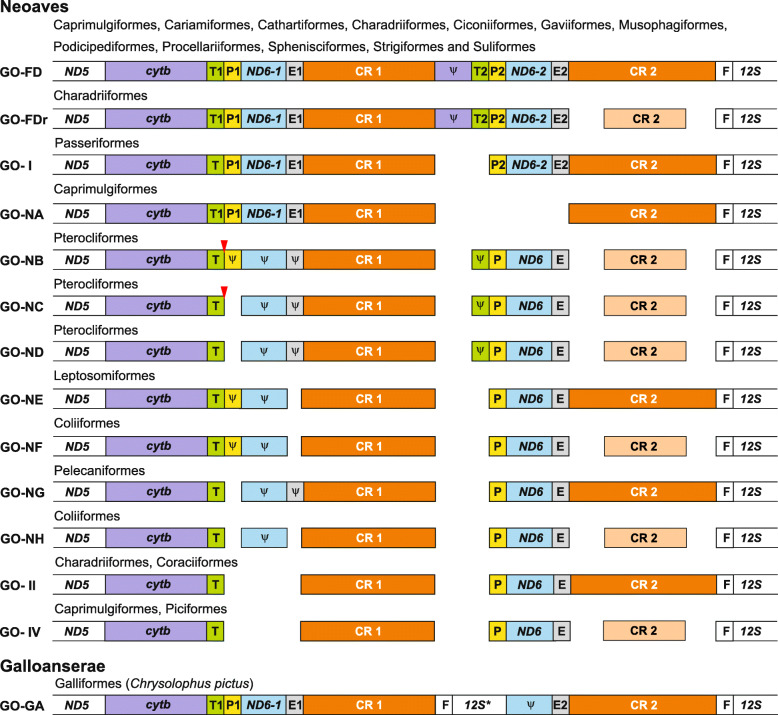
Mitochondrial gene orders between *ND5* and *12S rRNA* in Neognathae analyzed in this study. The red triangle indicates the position of microsatellite insertion. The asterisk at *12S rRNA* gene means that this version is shortened by 198 nucleotides in comparison to the full version in GenBank (NC_014576.1). See Fig. 1 for further explanations

So far, only two representatives of the order Chardriiformes have been shown to have duplication in mitochondrial genomes: *Calidris pugnax* (GQ255993.1) and *Turnix velox* (MK453380.1). However, our annotation and reannotation of another mitogenomes from *Alca torda* (CM018102.1), *Sterna hirundo* (CM020500.1) and *Uria aalge* (MN356418.1) revealed the presence of the most fully duplicated avian region (GO-FD) or its variant (GO-FDr), in which the second control region is remnant (Fig. 8 and Fig. S9 in Additional file 1). Therefore, we analyzed the mitogenomes of two of these species as well as 18 additional Charadriiformes annotated without duplication to identify a potentially unrecognized duplication in their mitogenomes. Using diagnostic PCR reactions amplifying a fragment between two control regions, we found GO-FD gene order in the mitogenomes of *Alca torda* and *Uria aalge* (Table 1 and Fig. S10 in Additional file 1) as well as additional 15 other species (paper in preparation). We also received previously omitted sequences of the GO-FD rearrangement in the mitogenomes of five additional avian orders: Cathartiformes (*Cathartes aura*), Ciconiiformes (*Ciconia nigra*), Gaviiformes (*Gavia arctica*, *Gavia stellata*), Podicipediformes (*Podiceps cristatus*) and Sphenisciformes (*Spheniscus demersus*) (Table 1 and Fig. S10 in Additional file 1). Moreover, our PCR experiments demonstrated the GO-FD gene order in the mitogenomes of *Apus apus* (Apodiformes), *Corythaixoides personatus* (Musophagiformes) and *Podiceps grisegena* (Podicipediformes) (Table 1 and Fig. S10 in Additional file 1). Furthermore, *ab initio* annotation of *Calypte anna* (Apodiformes) and *Puffinus lherminieri* (Procellariiformes) mitogenomes deposited in GenBank database revealed the presence of GO-FD (Fig. S9 in Additional file 1). The same gene order has been identified in the mitogenomes of *Morus serrator* (Suliformes) as well as *Ketupa blackistoni* and *Ketupa flavipes* (Strigiformes) after reannotation of their duplicated gene rearrangements (Fig. S9 in Additional file 1).

We also obtained interesting results for sole two species from Eurypygiformes, *Rhynochetos jubatus* and *Eurypyga helias*. For the former, two types of amplifying reactions showed no duplication, because the length of obtained amplicons corresponded to those predicted based on the reference sequence (MN356362.1), representing the typical avian gene order (Fig. S11a and b in Additional file 1). However, three out of nine reactions of the CR1/CR2 fragment gave an amplicon with 4000 bp accompanied by shorter ones (Fig. S11c in Additional file 1). These results suggest that these products represent NUMTs and there is no duplication in the *Rhynochetos jubatus* mitogenome, but it could be present in the past as indicated by the duplicated fragment transferred to the nuclear genome. For *Eurypyga helias*, we firstly amplified *cytb*/*12S rRNA* region, which produced a fragment 4000-bp longer than expected (Fig. S11d in Additional file 1). Thus, we checked fragments *cytb*/CR and CR/*12S rRNA* (Fig. S11e in Additional file 1). The latter occurred ca. 3600 bp longer than that in the reference genome, which strongly suggests the presence of duplication. Moreover, based on the length of amplicons *cytb*/CR, CR/*12S rRNA*, *cytb*/*12S rRNA* and obtained sequences, we found that the length of the region located between *tRNA-Glu* and *tRNA-Phe* genes is about 5100 bp. It includes a full CR terminated with repeated motifs, which are followed by an undetermined part and ended with another microsatellite region (Fig. S10 in Additional file 1). The great length of this fragment indicates that it most likely does not contain only one control region, because this fragment is 1704 bp longer than the longest single CR found in *Asio flammeus* (KP889214.1), so there is enough space for duplicates. Moreover, the length of this fragment corresponds well to those proved to contain two CRs in 46 representatives of avian orders closely related to Eurypygiformes, i.e. Charadriiformes, Gaviformes, Gaviiformes, Pelecaniformes, Phoenicopteriformes, Procellariiformes, Suliformes and Suliuformes [45, 47, 113]. The portions with two CRs are in the range of 2563–5575 bp with the average 3749 bp, which is even smaller than the length of the analyzed fragment in *Eurypyga helias*. This fragment should contain an additional but remnant CR and likely some pseudogenes, because no amplicon was obtained in diagnostic CR1/CR2 reactions (Table S9 in Additional file 2) and no read was get for *tRNA-Pro*, *ND6* and *tRNA-Glu* genes in sequencing of CR/*12S rRNA* amplicon.

A potential duplication was also noticed after the amplification of fragment *cytb*/*12S rRNA* for *Trogon collaris*, a representative of Trogoniformes (Fig. S11f in Additional file 1). The amplified product was ca. 3500 bp longer than expected. According to this, six out nine PCRs designed for CR1/CR2 showed several fragments with the length from ca. 3300 to 4300 bp indicating the presence of two CRs and their heteroplasmy (Fig. S11g in Additional file 1).

The data available in GenBank (in the day of 9.10.2020) and obtained in this study indicate that mitogenomes with sequenced duplications are distributed in 32 out of 43 avian orders (Table S6 in Additional file 2). Among 1261 species with known mitogenomes, 324 have a duplication and 713 do not show this feature, whereas in 46, both versions are reported. Among the species without duplication, 45 have partial mitogenomes too. Similarly, the mitogenomes of 187 species are too incomplete to classify them into one of two categories, with or without duplication. The number of mitogenomes with duplication is likely underestimated because of difficulties with the amplification and sequencing of repeated regions [4]. Accordingly, reanalysis of 13 crane mitogenomes, previously annotated without the duplication, showed that all of them contain the duplicated region [12]. Similarly, 15 mitogenomes of parrots from Cacatuidae and Nestoridae also revealed this character after using appropriate PCR and sequencing methods [23]. The obtained results indicate that underestimation of the mitogenomes with the duplication ranges from 85 to 100% in the case of Charadriiformes, Gruidae and Psittaciformes. It should be emphasized that the omission of GO-FD gene order can be common in the amplification and sequencing of avian mitogenomes using standard procedures due to the presence of two nearly identical copies.

#### Gene rearrangements in studied avian mitogenomes

The performed analyses revealed new gene orders in avian mitogenomes (Fig. 8). Fully duplicated gene rearrangement (GO-FD) was found in representatives of 12 avian orders Fig. S9 and S10). Other gene rearrangements are characterized by various degeneration levels of duplicated elements and were found in single or at most two avian groups, but some general tendencies can be noticed. The second copy of cytochrome b is the element that disappeared as the first in the evolution and shows pseudogene features already in the fully duplicated versions, GO-FD and GO-FDr. The second gene for tRNA-Thr fades also quite fast and is present intact only in the fully duplicated gene orders, whereas in GO-NB, GO-NC and GO-ND it is a pseudogene. CR2 also tends to degenerate because its remnant version is present in seven gene rearrangements. However, in the case of *tRNA-Pro*, *ND6* and *tRNA-Glu*, the first copies were subjected to decay, whereas the second ones remained. Both copies of these genes are present only in the fully duplicated versions and GO-I. The same tendencies in the deterioration of duplicated elements were observed in other avian mitogenomes of Accipitriformes [4, 114], Charadriiformes [115], Coraciiformes [116], Cuculiformes [10, 117], Falconiformes [4, 14, 29], Passeriformes [13, 14, 29, 41, 102, 118, 119], Piciformes [4, 97], Psittaciformes [23] and Strigiformes [24].

An exception is the gene order GO-NA in Caprimulgiformes, in which the first versions of *tRNA-Pro*, *ND6* and *tRNA-Glu* have been preserved, whereas the second ones were lost (Fig. 8). The same rearrangement was only reported in one representative of Procellariiformes [20], Passeriformes [120] and Psittaciformes [23]. The bias in the loss and preservation of duplicated elements can result from a specific selection for structural organization or a special mechanism of duplication around the control region.

Especially interesting is a gene order found in *Chrysolophus pictus* from Galliformes because it does not resemble any rearrangement reported in Aves. Between two control regions, there are: the *tRNA-Phe* gene, the 770-nt beginning of *12S rRNA* gene, the 66-nt end of *ND6* gene and the *tRNA-Glu* gene (Fig. 8 and S10). All these sequences are identical with those annotated in the GenBank record (NC_014576.1). Only the sequenced CR portions show 0.3% and 0.4% difference with the corresponding fragments. This gene order could have arisen by an insertion of duplicated genes *tRNA-Phe* and *12S rRNA* between CR1 and *ND6–2*.

#### Reconstruction of ancestral states in terms of mitogenomic duplications in Aves phylogeny

**Fig. 9:**
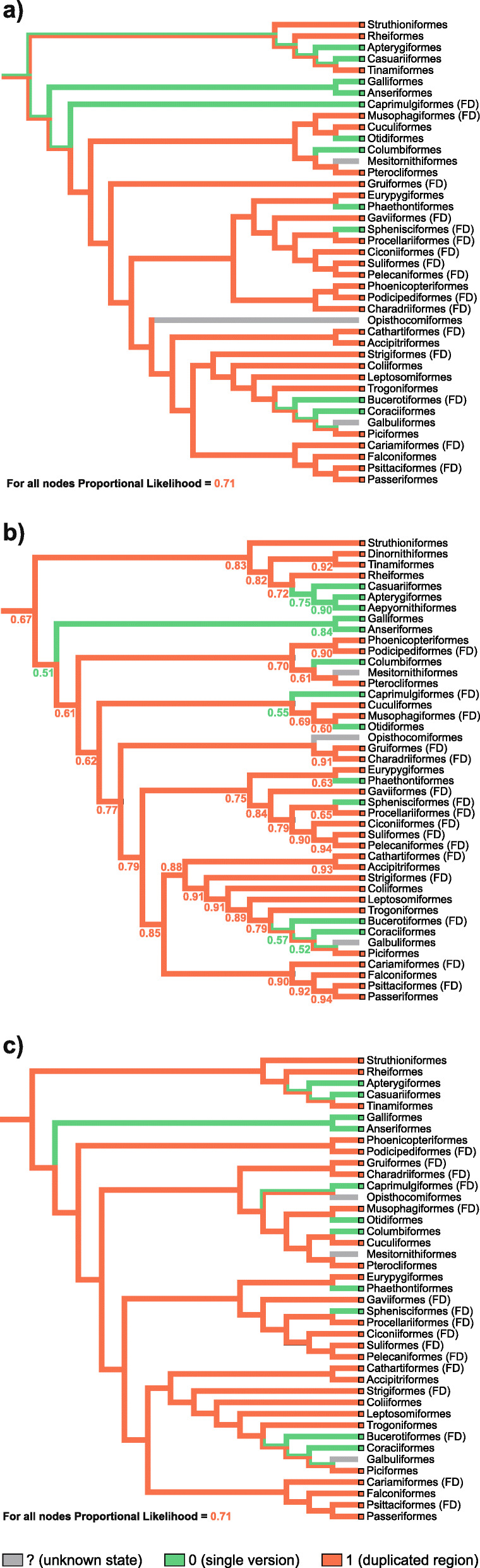
Reconstruction of ancestral states and mapping of mitogenomic duplications in Aves onto the three tree topologies. The colored thick lines of the trees correspond to maximum parsimony reconstruction, whereas values at nodes mean the probability (proportional likelihood) of a more likely ancestral state provided by maximum likelihood reconstruction. The tree obtained by Prum, et al. [45] is in **a**, by Kimball, et al. [47] in **b**, whereas by Kuhl, et al. [113] in c. The duplication state was assumed for a given avian order if duplication was found in at least 33% of its species or was inferred as an ancestral state in this study for Tinamiformes as well as in other studies for Passeriformes and Psittaciformes [14, 23]. FD indicates the presence of the most fully duplicated avian region. AsymmMk model was applied for ML approach

The gathered sequence data (Table S6 in Additional file 2) and results of PCR experiments were used to reconstruct the evolution of mitogenomic duplications in all birds. Using maximum parsimony (MP) and maximum likelihood (ML) methods, we mapped the data onto the phylogenetic trees of Aves obtained by Prum, et al. [45], Kimball, et al. [47] and Kuhl, et al. [113], so six approaches were applied in total. The reconstruction presented in Fig. 9 assumes the duplication state for a given avian order if at least 33% of its species show this feature or this state was already inferred as an ancestral state, i.e. for Tinamiformes (in this study, see Fig. 6), Passeriformes [14] and Psittaciformes [23]. This assumption is reasonable because it corresponds to only 41% underestimation of mitogenomes with the duplication, which is much smaller than that above-mentioned, 85–100%. This assumption implicates that the total number of species with such mitogenomes in the avian orders already containing at least one mitogenome of this type exceeds the number of species without the mitogenomic duplication. It would strongly suggest that the last common ancestor of these orders also contained the mitogenomic duplication in the past. As a result, there are such 27 avian orders out of 41 (Fig. 9 a and c) or 28 out of 43 (Fig. 9b).

For the tree topology obtained by Kimball, et al. [47], ML method produced 0.67 probability that the last common ancestor of all Aves contained a mitogenome with the duplication (Fig. 9b). The probability of this feature was still above 0.5 for the last common ancestors of Palaeognathae (0.83), Neoaves (0.61) and later diverged clades (≥0.62). In the case of Neognathae ancestor, the situation is ambiguous because the probability of its states is close to 0.5. Nevertheless, this method gave 0.71 probability of the duplication for all nodes in the tree topologies by Prum, et al. [45] and Kuhl, et al. [113] (Fig. 9 a and c). MP methods applied for the trees by Kimball, et al. [47] and Kuhl, et al. [113] also indicated the that the last common ancestor of all Aves had a mitogenome with duplication, which was also inherited by ancestors of deeply diverged lineages, Palaeognathae, Neognathae, Neoaves and its descendants (Fig. 9 b and c). However, for the tree by Prum, et al. [45], this method unresolved the states for the ancestors of these lineages and all Aves (Fig. 9a). This difference in the reconstruction is mainly caused by the placement of Caprimulgiformes, for which only five species with duplication and 20 without it were reported. Then, we assumed that the ancestor of this order did not contain a duplicated region in its mitogenome. This lineage diverged as the first among Neoaves in Prum, et al. [45] but later in the other trees [47, 113].

Concluding, more approaches indicate that the last common ancestor of all Aves and main bird groups contained a mitogenome with a duplication and no method favored the state without the duplication (Fig. 9). This mitogenomic feature was passed to the ancestors of many modern orders. Depending on the tree topology, the duplication was lost at least 8 or 9 times independently. Among the groups with the lost duplication is Galloanserae (including Galliformes and Anseriformes), for which the duplication was reported for one species in this study. If the duplicated regions are found in more representatives of avian orders, the presented conclusions would be only reinforced by the reconstruction of the ancestral states. If we assume the duplication state for all orders for which a duplication was reported in at least one species, the results of the six approaches will consistently show the ancestry of mitogenomic duplication (Fig. S12).

This view about the ancestry of the mitogenomic duplication can be further supported by the distribution of the most fully duplicated avian region (GO-FD). It includes the repetition of *cytb*/*tRNA-Thr*/*tRNA-Pro*/*ND6*/*tRNA-Glu*/CR in which only the cytochrome b gene is pseudogenized (Fig. 1). This rearrangement type occurs in 68 mitogenomes distributed among 17 bird orders (Fig. 9, Table S7 in Additional file 2). The length and complexity of this duplication suggests that it is unlikely that it occurred independently many times (see Fig. 9) because it would require the same recombination pattern and replication errors [12, 16, 25, 67]. Thus, it seems more probable that this state was inherited from shared ancestors by the avian lineages that contain this rearrangement type. In other bird groups, the duplicated regions were subjected to degenerations and loss of selected elements.

Whether the duplicated region is only a neutral trait or can provide a real selective advantage is an interesting question. Previous studies have suggested that bird species having mitogenomes with the duplicated control region can be characterized by longer life-span [23, 121] as well as a greater metabolic rate and energy production [14, 23]. However, further understanding the potential selective advantages of mitogenome duplications will require direct study at physiological and molecular levels.

## Conclusions

The obtained results indicate that duplicated control regions with adjacent genes are more common in Palaeognathae mitochondrial genomes than it was previously thought. What is more, this feature was most likely present in the last common ancestor of this avian group. Once the duplication occurred, it was preserved during the evolution of Struthioniformes, Rheiformes and some Tinamiformes. The duplicated regions were subjected to concerted evolution, which resulted in homogenization of some parts of duplicated regions and degeneration of others. Reconciliation of the mitogenome-based phylogenetic tree with a probable species tree based on nuclear markers suggests that the evolution of mitogenomes in Palaeognaths could have involved different mitogenomic variants in one cell, i.e. heteroplasmy, in a short period of time.

The distribution of mitogenomes with duplications across the avian phylogenetic tree implies that the last common ancestors of not only Palaeognathae but also major Neognathae groups and even all known Aves could have had a mitogenomic duplication, which was then inherited by many modern lineages. The presence of duplicated regions in mitogenomes can be a neutral feature associated with the mechanism of replication and recombination or can give a selective profits, e.g. more effective energy production by mitochondria. However, it needs further studies in many avian representatives.

## Methods

### Samples and DNA extraction

Blood, muscle and feather samples from avian species were obtained thanks to courtesy of European zoological gardens, universities, foundations, aviculture parks, animal rehabilitation centers and private breeding facilities (Table 1). They were taken as dry blood spots on a fiber filter dedicated to laboratory analyses and were preserved at − 20 °C in Eppendorf tubes sealed with parafilm to avoid damping. Total DNA was extracted with Sherlock AX Kit (A&A Biotechnology) according to the manufacturer’s protocol.

### PCR strategy for tandem duplication survey

To verify the presence or absence of tandem duplication within the mitochondrial genomes of Palaeognathae and other avian representatives, we used the strategy proposed by Gibb, et al. [4] and successfully applied to parrot mitogenomes [23]. Because two control regions (CRs) are common in the majority of avian duplicated rearrangements [22] and the paralogous CRs are usually nearly identical [12, 63], we designed appropriate primers D-F and D-R that were to anneal to the central parts of CRs (Table S1 in Additional file 2, Fig. 2). This property makes the PCR strategy diagnostic because the expected amplicons occur only when two control regions are present in the genome. Due to the high variability of the control region sequences from the analyzed taxa, it was impossible to use universal primers, which forced us to design primers specific to each genus or even species (Table S1 and S9 in Additional file 2). Based on the selected primers, we ran 12–48 different reactions for Palaeognathae representatives, 35 for *Chrysolophus pictus* (Galloanserae) and 4–25 for Neoaves representatives (Table S1 and S9 in Additional file 2).

### PCR strategy for amplification of the mitogenomic fragments containing the whole duplicated regions

The diagnostic fragment designed for tandem duplication survey comprises incomplete control regions, i.e. the second part of CR1 and the first part of CR2, as well as genes located between the two CRs. Therefore, in the case of taxa for which such a fragment was obtained, appropriate PCR reactions were performed to complete the missing parts of CRs and to reveal the order of genes preceding the CR1. The obtained partial CR1 sequences were used to design species specific CR-R primers for amplification of *tRNA-Leu*/CR1 fragment (for *Struthio camelus*) or *ND5*/CR1 fragments (for *Rhea americana*, *Rhea pennata*, *Crypturellus tataupa*) (Fig. 2). Similarly, the partial CR2 sequences were used to design species specific CR-F primers for amplification of CR2/16S fragments (for *Struthio camelus*, *Rhea americana*, *Rhea pennata*) or CR2/12S fragment (for *Crypturellus tataupa*) (Fig. 2). Appropriate L-F, ND5-F, 12S-R and 16S-R primers were designed based on reference mitogenomic sequences of the analyzed or related taxa deposited in GenBank (AF338715.1, AF090339.1, AF338709.2, AF338710.2). Suitable elongation times were applied to avoid amplification of *tRNA-Leu*/CR2, *ND5*/CR2, CR1/12S and CR1/16S fragments, which would contain two copies of some genes and/or control regions. Additional diagnostic *ND6–1*/*ND6–2* fragments were amplified to confirm that the CR1/CR2 sequences were not errors of the PCR reactions or copies present in the nuclear genome, i.e. nuclear mitochondrial DNA inserts (NUMTs). Finally, the whole duplicated mitogenomic regions of *Struthio camelus*, *Rhea americana*, *Rhea pennata* and *Crypturellus tataupa* were amplified in four overlapping fragments (Fig. 2). Both amplicons containing only one control region fragment (CR1 or CR2) were 3 kb to 5 kb in length, which excludes a possibility of NUMTs amplification, whose average size is usually below 1 kb [122]. Despite the length of two diagnostic fragments depends on elements (genes and/or control region) located between CRs or *ND6* genes, their length was longer than 1 kb, i.e. 2–3 kb on average. Uncropped and unprocessed agarose gels were presented in Fig. S13 in Additional file 1.

Similar amplification strategies based on fragments *ND5*/CR1, *cytb*/CR1, *tRNA-Pro1*/*tRNA-Pro2*, *ND6–1*/*tRNA-Pro2*, CR2/*12S rRNA* (Table S9 in Additional file 2) were used in the case of selected Neoaves representatives to verify the presence of GO-FD gene order.

### DNA amplification and sequencing

The PCR amplifications were performed in 25 μl reaction mixture containing 50 ng of the DNA template, 1 U DreamTaq Green DNA Polymerase (Thermo Fisher Scientific), 2.5 μl of 10 x buffer, 0.6 μl of 10 mM dNTPs, and 0.6 μl of each primer (10 μM). In the case of diagnostic fragments (CR1/CR2, *ND6–1*/*ND6–2*, *tRNA-Pro1*/*tRNA-Pro2*, *ND6–1*/*tRNA-Pro2*), following program was used: 94 °C for 5 min; 94 °C for 30 s, 56–62 °C for 30 s, 72 °C for 120 s repeated 35 times; and 72 °C for 5 min. In the case of all other fragments the reaction conditions were as follows: 94 °C for 5 min; 94 °C for 30 s, 56–62 °C for 30 s, 72 °C for 180 s repeated 35 times; and 72 °C for 5 min. For each amplified fragment, the appropriate amount of the PCR reaction mixtures was cleaned with the use of Clean-up Kit (A&A Biotechnology) to obtain the final volume of 100 μl with the concentration of at least 50 ng/μl. The two DNA strands of the cleaned PCR products were sequenced using the Primer Walking method (Wyzer Biosciences Inc., Cambridge, MA). Overlaps between three or four fragments amplified for each species were sufficient to assemble the whole mitogenomic regions including duplicated elements with the use of appropriate avian reference mitogenomes containing GO-FD (Table S7 in Additional file 2) or GO-I (*Notiomystis cincta* and *Turdus philomelos*) gene orders.

### Sequence analyses

The annotation of genes was performed with the help of MITOS [123]. MEGA software [124] with MUSCLE aligner [125] were used to inspect and align sequences. Default parameters were assumed in the sequence alignments. JalView [126] was applied to visualize the alignments. Control regions were searched for potential pseudogenes using the optimal global:local algorithm (glsearch) form FASTA package version 36.3.8 g [127]. We assumed one million of shuffles, match/mismatch scores 5/− 4 and gap cost opening/extension − 10/− 1.

### Phylogenetic analyses

Phylogenetic relationships between Palaeognathae were inferred based on all available 19 complete or almost complete mitochondrial genomes (Table 2). Five representatives of Neognathae were used as an outgroup: *Anas platyrhynchos* (NC_009684.1), *Anseranas semipalmata* (NC_005933.1), *Crax daubentoni* (NC_024617.1), *Gallus gallus* (NC_001323.1) and *Numida meleagris* (NC_034374.1). The sequence records were downloaded from the GenBank database. The analyses were based on nucleotide sequences of 13 protein coding genes, genes for 12S and 16S rRNAs, as well as 22 tRNAs. Sequences of the control region (CR) were excluded due to their high variation. The sequences were aligned in MAFFT using a slow and accurate algorithm L-INS-i with 1000 cycles of iterative refinement [128]. The resulted alignments were edited manually in JalView [126] and sites suitable for phylogenetic study were selected in GBlocks [129]. The concatenated alignment of mitochondrial genes consisted of 15,351 bp.

We applied three phylogenetic approaches in the phylogenetic analyses: the maximum likelihood method in IQ-TREE [130], as well as Bayesian analyses in MrBayes [131] and PhyloBayes [132]. We considered 63 potential partitions, i.e. three codon positions for each individual protein coding gene and separate partitions for each of RNA genes to test the necessity of using separate substitution models.

The ModelFinder program associated with IQ-TREE [133, 134], proposed 12 partitions with individual substitution models (Table S8 in Additional file 2). In the tree search, we used the more thorough and slower NNI search. In IQ-TREE, we applied Shimodara-Hasegawa-like approximate likelihood ratio test (SH-aLRT) assuming 10,000 replicates and non-parametric bootstrap with 1000 replicates.

In MrBayes, we assumed 11 substitution models for the appropriate partitions as proposed by PartitionFinder [135]. However, we implemented mixed models rather than fixed ones to specify appropriate substitution models across the large parameter space [136], but the assumptions on the heterogeneity rate across sites were adopted from PartitionFinder results (Table S4 in Additional file 2). Two independent runs each using 8 Markov chains were applied and trees were sampled every 100 generations for 20,000,000 generations. In the final analysis, we selected trees from the last 12,866,000 generations that reached the stationary phase and convergence, i.e. when the standard deviation of split frequencies (SD) stabilized and was below 0.00004.

In PhyloBayes, we applied the CAT-GTR + Γ5 model with the number of components, weights and profiles of the model inferred from the data. Two independent Markov chains were run for 100,000 generations with one tree sampled for each generation. The last 50,000 trees from each chain were collected to compute posterior consensus trees after reaching convergence, when the largest discrepancy observed across all bipartitions (maxdiff) was 0.0055.

To reduce a potential compositional heterogeneity in sequences related with AT or GC bias, we recoded respective nucleotides for purines (R) and pyrimidines (Y) in the third codon positions of protein-coding genes. In MrBayes and PhyloBayes, we adopted the assumptions as described above, whereas in IQ-TREE, we applied 11 partitions with individual substitution models proposed by ModelFinder (Table S10 in Additional file 2). The posterior consensus was calculated for trees from the last 5,845,000 generations in MrBayes for SD < 0.00013 and 90,000 generations in PhyloBayes for maxdiff = 0.007.

Besides the study based on the full alignment, we also performed phylogenetic analyses using ten alignments with sequentially removed partitions characterized by the highest substitution rate. The partition-specific rate was taken from MrBayes estimations. The settings for this study were the same as above. In the case of IQ-TREE analyses, the best substitution models were calculated for each set every time.

The best tree produced in IQ-Tree was compared in Consel [137] with alternative topologies using available tests. We applied 1000,000 replicates and site-wise log-likelihoods for the compared trees calculated in IQ-Tree under the best fitted substitution models. Moreover, we analyzed competitive topologies using Bayes Factor in MrBayes based on the stepping-stone method estimating the mean marginal likelihood using 50 steps of the sampling algorithm and other parameters as described above.

The data about the presence and absence of duplication in the palaeognath mitogenomes were mapped on the phylogenetic tree using Mesquite [138]. The lack of data about the duplication was coded as missing data. We applied maximum parsimony and maximum likelihood reconstruction methods. Depending on the AIC criterion, we used the better fit model for the data: either the Mk1 model (Markov k-state 1 parameter model) or the AsymmMk model (asymmetrical Markov k-state 2 parameter model).

## Supplementary Information

**Additional file 1: Fig. S1**. Representative cropped agarose gels of CR1/CR2 amplicons obtained for *Struthio camelus* (a), *Rhea pennata* (b), *Rhea americana* (c) and *Crypturellus tataupa* (d). The numbering of amplicons separated in the agarose gel corresponds to the reaction numbered in Table S1 in Additional file 2. The numbers of PCR fragments, which were finally sequenced, are underlined. Lane M - GeneRuler 1 kb DNA Ladder (Thermo Scientific). Original uncropped and unprocessed gels are presented in Fig. S13a-d. **Fig. S2**. Sequence comparison of two copies of *tRNA-Pro*, *ND6from*, *tRNA-Glu* genes and control region, found in this study as duplicated, with appropriate sequences of mitogenomes previously deposited in GenBank and showing a typical avian gene order. The alignments are shown for *Struthio camelus* (a), *Rhea americana* (b), *Rhea pennata* (c) and *Crypturellus tataupa* (d). Dots indicate residues identical in the obtained copies with those in the single sequence previously published. Genes for tRNA-Pro are marked in yellow coloring, NADH dehydrogenase subunit 6 in cyan coloring, tRNA-Glu in grey shading, and control regions in red fonts. In the case of *Crypturellus tataupa* species only two obtained copies are compared to each other because the previously published mitogenome of these taxa is incomplete and it does not contain any of the analyzed genes or the control region. The sequences were aligned with MUSCLE [125] in MEGA [124]. **Fig. S3**. Sequences of mitogenomic fragments amplified and sequenced in this study for *Struthio camelus* (MH264503), *Rhea americana* (MK696563), *Rhea pennata* (MK696564) and *Crypturellus tataupa* (MK696562). Genes for tRNA-Leu are marked in bold, NADH dehydrogenase subunit 5 in magenta coloring, cytochrome b in red coloring, tRNA-Thr in green coloring, tRNA-Pro in yellow coloring, NADH dehydrogenase subunit 6 in cyan coloring, tRNA-Glu in grey shading, 12S rRNA in blue fonts, tRNA-Val in brown fonts, 16S rRNA in green fonts, tRNA-Phe are in bold and underlined, and control regions in red fonts. **Fig. S4**. Cropped agarose gels of potential NUMTs amplified for *Dromaius novaehollandiae* (a) and *Casuarius casuarius* (b) with the use of primers designed for detection of CR1/CR2 fragments. The numbering of lanes corresponds to the reaction numbered in Table S1 in Additional file 2. Lane M - GeneRuler 1 kb DNA Ladder (Thermo Scientific). Original uncropped and unprocessed gels are presented in Fig. S13e and f. **Fig. S5**. Comparison of selected sequence associated with reannotation of Palaeognathae mitogenomes. *Eudromia elegans* gene for tRNA-Pro with the last 200 nucleotides of the control region (a); *Emeus crassus*, *Dinornis giganteus* and *Anomalopteryx didiformis* control region 5′ spacers (b); *Probosciger aterrinus* (MH133970.1), *Eolophus roseicapilla* (MH133971.1) and *Cacatua moluccensis* (MH133972.1) *ND6* pseudogenes (c); *Anomalopteryx didiformis ND6* gene with control region 5′ spacer (d); *Emeus crassus ND6* gene with control region 5′ spacer (e); *Dinornis giganteus ND6* gene with control region 5′ spacer (f). Sequences rich in G and A residues are marked in a red box. The sequences in b and c were aligned with MUSCLE [125] in MEGA [124], whereas those in a, d, e and f using the optimal global:local algorithm (glsearch) form FASTA package version 36.3.8 g [127]. **Fig. S6**. Sequence comparison of two *Eudromia elegans* control regions. Dots indicate residues identical in the compared copies. Dots indicate residues identical in the compared sequences. Sequence repeats present in both control regions are marked in red and blue boxes. The sequences were aligned with MUSCLE [125] in MEGA [124]. **Fig. S7**. Influence of removing partitions with the highest substitution rate on alignment and tree parameters: the number of parsimony informative sites and mean distance (a) as well as mean support values (b). The mean phylogenetic distance was obtained from MrBayes tree. The mean support values were calculated from posterior probabilities received in MrBayes and PhyloBayes as well as SH-aLRT and non-parametric bootstrap percentages obtained in IQ-TREE. The posterior probabilities were scaled to 100%. **Fig. S8**. Maximum parsimony (a, c) and maximum likelihood (b, d) reconstruction of ancestral states and mapping of mitogenomic duplications onto the Palaeognathae tree topology t1 based on sequences of mitochondrial genes (a, b) and the Palaeognathae tree topology t2 supported by nuclear markers in coalescent-based approaches (c, d). In contrast to Fig. 6, this approach assumes that *Anomalopteryx didiformis*, *Emeus crassus* and *Dinornis giganteus* already lost mitogenomic duplication. The area of colors at nodes in b and d corresponds to the probability of the given state, single or duplicated region. Two-colored branches correspond to the equal probability of two states, single or duplicated region. The probability value for a more likely state was also given at these nodes. Mk1 model was applied for ML approach. **Fig. S9**. Annotated and reannotated duplicated gene orders in mitogenomes of Neoaves and Galloanserae deposited in GenBank for: *Alca torda* (CM018102.1), *Antrostomus carolinensis* (MN356120.1), *Calypte anna* (CM016612.1), *Cariama cristata* (CM020379.1), *Catharus ustulatus* (CM020378.1), *Colius striatus* (MN356125.1), *Ketupa blackistoni* (LC099104), *Ketupa flavipes* (LC099100.1), *Leptosomus discolour* (MN356135.1), *Merops nubicus* (CM020464.1), *Melanereps aurifrons* (CM022134.1), *Morus serrator* (GU071056.1), *Nyctibius grandis* (CM023771.1), *Pterocles burchelli* (MN356340.1), *Pterocles gutturalis* (CM020177.1, MN356147.1), *Puffinus lherminieri* (MH206162.1), *Rynchops niger* (MN356248.1), *Sterna hirundo* (CM020500.1), *Thalassarche chlororhynchos* (MN356342.1), *Uria aalge* (MN356418.1) and *Urocolius indicus* (MN356373.1). Genes for NADH dehydrogenase subunit 5 are marked in magenta coloring, cytochrome b in red coloring, tRNA-Thr in green coloring, tRNA-Pro in yellow coloring, NADH dehydrogenase subunit 6 in cyan coloring, tRNA-Glu in grey shading, 12S rRNA in blue fonts, tRNA-Phe in bold fonts, and control regions in red fonts. Pseudogenes are in italics and underlined. Microsatellites present within *Pterocles gutturalis* mitogenomes between *tRNA-Thr* gene and *tRNA-Pro* pseudogene or *tRNA-Thr* gene and *ND6* pseudogene are marked in brown. **Fig. S10**. Sequences of mitogenomic fragments in Neoaves and Galloanserae mitogenomes amplified and sequenced for: *Alca torda* (MK263222), *Apus apus* (MW151827), *Cathartes aura* (MN629891), *Chrysolophus pictus* (MW151829), *Ciconia nigra* (MH264509), *Corythaixoides personatus* (MW082596), *Gavia arctica* (MK263210), *Gavia stellata* (MK263209), *Eurypyga helias* (MW208859), *Podiceps cristatus* (MN629890), *Podiceps grisegena* (MK263194), *Scopus umbretta* (MW151828), *Spheniscus demersus* (MH264510) and *Uria aalge* (MK263188). Genes for NADH dehydrogenase subunit 5 are marked in magenta coloring, cytochrome b in red coloring, tRNA-Thr in green coloring, tRNA-Pro in yellow coloring, NADH dehydrogenase subunit 6 in cyan coloring, tRNA-Glu in grey shading, 12S rRNA in blue fonts, tRNA-Phe in bold fonts, and control regions in red fonts. Pseudogenes are in italics and underlined. **Fig. S11**. Representative cropped agarose gels of the following amplicons: *cytb*/12S rRNA obtained *for Rhynochetos jubatus* (a), *Eurypyga helias* (d) and *Trogon collaris* (f); CR1/CR2 obtained for *Rhynochetos jubatus* (c) and *Trogon collaris* (g); CR/12S rRNA obtained for *Rhynochetos jubatus* (b) and *Eurypyga helias* (e); *cytb*/CR obtained for *Eurypyga helias* (e). The numbering of amplicons separated in the agarose gel corresponds to the reaction numbered in Table S9. Lane M - GeneRuler 1 kb DNA Ladder (Thermo Scientific). **Fig. S12**. Reconstruction of ancestral states and mapping of mitogenomic duplications onto the Aves three tree topologies under a more liberal assumption for assignment of duplication states. The colored thick lines of the trees correspond to maximum parsimony reconstruction, whereas values at nodes mean the probability of a more likely ancestral state (proportional likelihood) provided by maximum likelihood reconstruction. The trees obtained by Prum, et al. [45] are in a, by Kimball, et al. [47] in b, whereas by Kuhl, et al. [113] in c. The duplication state was assumed for a given order if a duplication was reported in at least one species from this order. FD indicates the presence of the most fully duplicated avian region. In ML approach, AsymmMk model was applied for a and, c whereas Mk1 for b. **Fig. S13**. Original uncropped and unprocessed agarose gels of: CR1/CR2 amplicons obtained for *Struthio camelus* (a), which were also shown in the Fig. S1a; CR1/CR2 amplicons obtained for *Rhea pennata* (b) which were also shown in the Fig. S1b; CR1/CR2 amplicons obtained for *Rhea americana* (7 lanes to the left of DNA Ladder) (c), which were also shown in the Fig. S1c; CR1/CR2 amplicons obtained for *Crypturellus tataupa* (6 lanes to the right of the DNA Ladder) (d), which were also shown in the Fig. S1d; potential NUMTs amplified for *Dromaius novaehollandiae* (e), which were also shown in the Fig. S4a; potential NUMTs amplified for *Casuarius casuarius* (f), which were also shown in the Fig. S4b; *cytb*/*12S rRNA* amplicons obtained for *Rhynochetos jubatus* (g), which were shown in the Fig. S11a; CR/*12S rRNA* amplicons obtained for *Rhynochetos jubatus* (h), which were shown in the Fig. S11b; CR1/CR2 amplicons (potential NUMTs) obtained for *Rhynochetos jubatus* (i), which were shown in the Fig. S11c; *cytb*/*12S rRNA* amplicon obtained for *Eurypyga helias* (j), which was shown in the Fig. S11d; *cytb*/CR and CR/*12S rRNA* amplicons obtained for *Eurypyga helias* (k), which were shown in the Fig. S11e; *cytb*/*12S rRNA* amplicon obtained for *Trogon collaris* (the first lane prior the first DNA Ladder) (l), which was shown in the Fig. S11f; CR1/CR2 amplicons obtained for *Trogon collaris* (m), which were shown in the Fig. S11g.

**Additional file 2: Table S1**. Characteristics of primers and PCR reactions for amplification of CR1/CR2 fragments. Sequences of primers, their internal laboratory numbering and naming as well as positions according to reference mitogenomes are included. Reactions that failed are marked with an asterisk. Reactions whose products were finally sequenced are marked in grey shading. **Table S2**. Characteristics of control regions in Palaeognathae species based on mitochondrial fragments obtained in this study and previously published mitogenomes. **Table S3**. Characteristics of alignments between *ND6* copies for various avian taxa. **Table S4**. Substitution models, partitions and their relative rate in MrBayes analysis. **Table S5**. Results of tests comparing various palaeognath tree topologies. The topology t1 corresponds to the best tree found for the full alignment. The topologies are shown in Fig. 5. The table includes: *p*-value from an approximately unbiased test (AU), the bootstrap probability calculated from the multiscale bootstrap (NP), the bootstrap probability calculated in the usual manner (BP), Bayesian posterior probability calculated by the BIC approximation (PP), p-value of the Shimodaira-Hasegawa (SH) and the weighted Shimodaira-Hasegawa tests (WSH), Bayes factor (BF) expressed in natural logarithm units as differences between marginal likelihoods of the given and the topology (t1). **Table S6**. Number of known mitogenomic sequences and species in terms of duplication for individual avian orders. **Table S7**. Avian species in which GO-FD gene order was identified in their mitogenomes. Previous annotations assuming single, i.e. without duplication, version were also included. Incomplete mitogenomes are marked with an asterisk. **Table S8**. Substitution models and partitions used in IQ-TREE analysis. **Table S9**. Characteristics of primers and PCR reactions for amplification of mitochondrial fragments from Neoaves and Galloanserae representatives. Sequences of primers, their internal laboratory numbering and naming as well as positions according to reference mitogenomes are included. Reactions that failed are marked with an asterisk. Reactions whose products were finally sequenced are marked in grey shading. **Table S10**. Substitution models and partitions used in IQ-TREE analysis for RY-recoded sequences.

## Data Availability

Newly generated sequences during the current study are available in the GenBank repository under the accession numbers: MH264503, MH264509, MH264510, MK263188, MK263194, MK263209, MK263210, MK263222, MK696562–4, MN629890, MN629891, MW082596, MW151827–9, MW208859. All other data generated or analyzed during this study are included in this published article.

## Abbreviations

CR: Control region
CR1: The first copy of control region
CR2: The second copy of control region
GO: Gene order
GO-FD: Fully duplicated gene order
GO-P: Palaeognathae gene order
ILS: Incomplete lineage sorting
ML: Maximum likelihood
MP: Maximum parsimony
NUMTs: Nuclear mitochondrial DNA inserts
SH-aLRT: Shimodara-Hasegawa-like approximate likelihood ratio test

## References

[1] Lavrov DV (2007). Key transitions in animal evolution: a mitochondrial DNA perspective. Integr Comp Biol.

[2] Lavrov DV, Desalle R, Schierwater B (2011). Key Transitions in Animal Evolution: a Mitochondrial DNA Perspective. Key Transitions in Animal Evolution.

[3] Desjardins P, Morais R (1990). Sequence and gene organization of the chicken mitochondrial genome. A novel gene order in higher vertebrates. J Mol Biol.

[4] Gibb GC, Kardailsky O, Kimball RT, Braun EL, Penny D (2007). Mitochondrial genomes and avian phylogeny: complex characters and resolvability without explosive radiations. Mol Biol Evol.

[5] Roques S, Godoy JA, Negro JJ, Hiraldo F (2004). Organization and variation of the mitochondrial control region in two vulture species, Gypaetus barbatus and Neophron percnopterus. J Hered.

[6] Sammler S, Bleidorn C, Tiedemann R (2011). Full mitochondrial genome sequences of two endemic Philippine hornbill species (Aves: Bucerotidae) provide evidence for pervasive mitochondrial DNA recombination. BMC Genomics.

[7] Verkuil YI, Piersma T, Baker AJ (2010). A novel mitochondrial gene order in shorebirds (Scolopacidae, Charadriiformes). Mol Phylogenet Evol.

[8] Huang R, Zhou YY, Yao YF, Zhao B, Zhang YL, Xu HL (2016). Complete mitochondrial genome and phylogenetic relationship analysis of Garrulax affinis (Passeriformes, Timaliidae). Mitochondrial DNA A.

[9] Pacheco MA, Battistuzzi FU, Lentino M, Aguilar RF, Kumar S, Escalante AA (2011). Evolution of modern birds revealed by Mitogenomics: timing the radiation and origin of Major orders. Mol Biol Evol.

[10] Pratt RC, Gibb GC, Morgan-Richards M, Phillips MJ, Hendy MD, Penny D (2009). Toward resolving deep neoaves phylogeny: data, signal enhancement, and priors. Mol Biol Evol.

[11] Wang N, Liang B, Huo J, Liang W (2016). Complete mitochondrial genome and the phylogenetic position of the lesser cuckoo, Cuculus poliocephalus (ayes: Cuculiformes). Mitochondrial DNA A.

[12] Akiyama T, Nishida C, Momose K, Onuma M, Takami K, Masuda R (2017). Gene duplication and concerted evolution of mitochondrial DNA in crane species. Mol Phylogenet Evol.

[13] Caparroz R, Rocha AV, Cabanne GS, Tubaro P, Aleixo A, Lemmon EM, Lemmon AR. Mitogenomes of two neotropical bird species and the multiple independent origin of mitochondrial gene orders in Passeriformes. Mol Biol Rep. 2018;45(3):279–85. 10.1007/s11033-018-4160-5 29455315

[14] Mackiewicz P, Urantowka AD, Kroczak A, Mackiewicz D (2019). Resolving phylogenetic relationships within Passeriformes based on mitochondrial genes and inferring the evolution of their Mitogenomes in terms of duplications. Genome Biol Evol.

[15] Rodrigues P, A'lvarez P, Verdugo C (2017). Complete mitochondrial genome of the Neotropic cormorant (Phalacrocorax brasilianus). Mitochondrial DNA A.

[16] Zhou X, Lin Q, Fang W, Chen X (2014). The complete mitochondrial genomes of sixteen ardeid birds revealing the evolutionary process of the gene rearrangements. BMC Genomics.

[17] Luo X, Kang XB, Zhang DY (2016). Complete mitochondrial genome of the American flamingo, Phoenicopterus ruber (Phoenicopteriformes, Phoenicopteridae). Mitochondrial DNA A.

[18] Morgan-Richards M, Trewick SA, Bartosch-Haerlid A, Kardailsky O, Phillips MJ, McLenachan PA, Penny D (2008). Bird evolution: testing the Metaves clade with six new mitochondrial genomes. BMC Evol Biol.

[19] Fuchs J, Pons JM, Pasquet E, Bonillo C (2016). Complete mitochondrial genomes of the white-browed piculet (Sasia ochracea, Picidae) and pale-billed woodpecker (Campephilus guatemalensis, Picidae). Mitochondrial DNA A.

[20] Gibb GC, Kennedy M, Penny D (2013). Beyond phylogeny: pelecaniform and ciconiiform birds, and long-term niche stability. Mol Phylogenet Evol.

[21] Lounsberry ZT, Brown SK, Collins PW, Henry RW, Newsome SD, Sacks BN (2015). Next-generation sequencing workflow for assembly of nonmodel mitogenomes exemplified with North Pacific albatrosses (Phoebastria spp.). Mol Ecol Resour.

[22] Eberhard JR, Wright TF (2016). Rearrangement and evolution of mitochondrial genomes in parrots. Mol Phylogenet Evol.

[23] Urantowka AD, Kroczak A, Silva T, Padron RZ, Gallardo NF, Blanch J, Blanch B, Mackiewicz P (2018). New insight into parrots' mitogenomes indicates that their ancestor contained a duplicated region. Mol Biol Evol.

[24] Hanna ZR, Henderson JB, Sellas AB, Fuchs J, Bowie RCK, Dumbacher JP (2017). Complete mitochondrial genome sequences of the northern spotted owl (Strix occidentalis caurina) and the barred owl (Strix varia; Aves: Strigiformes: Strigidae) confirm the presence of a duplicated control region. PeerJ.

[25] Morris-Pocock JA, Taylor SA, Birt TP, Friesen VL (2010). Concerted evolution of duplicated mitochondrial control regions in three related seabird species. BMC Evol Biol.

[26] Haddrath O, Baker AJ (2001). Complete mitochondrial DNA genome sequences of extinct birds: ratite phylogenetics and the vicariance biogeography hypothesis. Proc Biol Scie Royal Soc.

[27] Zhang LT, Zhang M, He SY (2017). The complete mitochondrial genome of great cormorant, Phalacrocorax carbo (Phalacrocorax, Phalacrocoracidae). Mitochondrial DNA A.

[28] Abbott CL, Double MC, Trueman JW, Robinson A, Cockburn A (2005). An unusual source of apparent mitochondrial heteroplasmy: duplicate mitochondrial control regions in Thalassarche albatrosses. Mol Ecol.

[29] Mindell DP, Sorenson MD, Dimcheff DE (1998). Multiple independent origins of mitochondrial gene order in birds. Proc Natl Acad Sci U S A.

[30] Schirtzinger EE, Tavares ES, Gonzales LA, Eberhard JR, Miyaki CY, Sanchez JJ, Hernandez A, Mueller H, Graves GR, Fleischer RC (2012). Multiple independent origins of mitochondrial control region duplications in the order Psittaciformes. Mol Phylogenet Evol.

[31] Alstrom P, Ericson PG, Olsson U, Sundberg P (2006). Phylogeny and classification of the avian superfamily Sylvioidea. Mol Phylogenet Evol.

[32] Boore JL (2006). The use of genome-level characters for phylogenetic reconstruction. Trends Ecol Evol.

[33] Boore JL, Brown WM (1998). Big trees from little genomes: mitochondrial gene order as a phylogenetic tool. Curr Opin Genet Dev.

[34] Boore JL, Fuerstenberg SI (2008). Beyond linear sequence comparisons: the use of genome-level characters for phylogenetic reconstruction. Philos T R Soc B.

[35] Rokas A, Holland PW (2000). Rare genomic changes as a tool for phylogenetics. Trends Ecol Evol.

[36] Qin XM, Guan QX, Shi JP, Hou LX, Qin PS (2013). Complete mitochondrial genome of the Spilornis cheela (Falconiformes, Accipitridae): comparison of S. cheela and Spizaetus alboniger. Mitochondrial DNA.

[37] Jiang L, Chen J, Wang P, Ren QQ, Yuan J, Qian CJ, Hua XH, Guo ZC, Zhang L, Yang JK (2015). The mitochondrial genomes of Aquila fasciata and Buteo lagopus (Aves, Accipitriformes): sequence, structure and phylogenetic analyses (vol 10, e0136297, 2015). PLoS One.

[38] Liu G, Li C, Du Y, Liu X (2017). The complete mitochondrial genome of Japanese sparrowhawk (Accipiter gularis) and the phylogenetic relationships among some predatory birds. Biochem Syst Ecol.

[39] Sveinsdottir M, Guomundsdottir L, Magnusson KP (2017). Complete mitochondrial genome of the gyrfalcon Falco rusticolus (Aves, Falconiformes, Falconidae). Mitochondrial DNA A.

[40] Slack KE, Delsuc F, McLenachan PA, Arnason U, Penny D (2007). Resolving the root of the avian mitogenomic tree by breaking up long branches. Mol Phylogenet Evol.

[41] Gibb GC, England R, Hartig G, McLenachan PA, Smith BLT, McComish BJ, Cooper A, Penny D (2015). New Zealand passerines help clarify the diversification of Major songbird lineages during the Oligocene. Genome Biol Evol.

[42] Barker FK (2014). Mitogenomic data resolve basal relationships among passeriform and passeridan birds. Mol Phylogenet Evol.

[43] Krajewski C, Sipiorski JT, Anderson FE (2010). Complete mitochondrial genome sequences and the phylogeny of cranes (Gruiformes: Gruidae). Auk.

[44] Jarvis ED, Mirarab S, Aberer AJ, Li B, Houde P, Li C, Ho SY, Faircloth BC, Nabholz B, Howard JT (2014). Whole-genome analyses resolve early branches in the tree of life of modern birds. Science.

[45] Prum RO, Berv JS, Dornburg A, Field DJ, Townsend JP, Lemmon EM, Lemmon AR (2015). A comprehensive phylogeny of birds (Aves) using targeted next-generation DNA sequencing. Nature.

[46] Burleigh JG, Kimball RT, Braun EL (2015). Building the avian tree of life using a large-scale, sparse supermatrix. Mol Phylogenet Evol.

[47] Kimball RT, Oliveros CH, Wang N, White ND, Barker FK, Field DJ, Ksepka DT, Chesser RT, Moyle RG, Braun MJ (2019). A Phylogenomic Supertree of birds. Diversity.

[48] Hackett SJ, Kimball RT, Reddy S, Bowie RC, Braun EL, Braun MJ, Chojnowski JL, Cox WA, Han KL, Harshman J (2008). A phylogenomic study of birds reveals their evolutionary history. Science.

[49] Gill F, Donsker D: IOC World Bird List (v9.2). https://wwwworldbirdnamesorg/ 2019.

[50] Clements JF, Schulenberg TS, Iliff MJ, Roberson D, Fredericks TA, Sullivan BL, Wood CL: The eBird/Clements checklist of birds of the world: v2018. http://wwwbirdscornelledu/clementschecklist/download/ 2018.

[51] Baker AJ, Haddrath O, McPherson JD, Cloutier A (2014). Genomic support for a moa-tinamou clade and adaptive morphological convergence in flightless ratites. Mol Biol Evol.

[52] Grealy A, Phillips M, Miller G, Gilbert MTP, Rouillard JM, Lambert D, Bunce M, Haile J (2017). Eggshell palaeogenomics: Palaeognath evolutionary history revealed through ancient nuclear and mitochondrial DNA from Madagascan elephant bird (Aepyornis sp.) eggshell. Mol Phylogenet Evol.

[53] Haddrath O, Baker AJ (2012). Multiple nuclear genes and retroposons support vicariance and dispersal of the palaeognaths, and an early cretaceous origin of modern birds. Proc Biol Sci Royal Soc.

[54] Harshman J, Braun EL, Braun MJ, Huddleston CJ, Bowie RCK, Chojnowski JL, Hackett SJ, Han KL, Kimball RT, Marks BD (2008). Phylogenomic evidence for multiple losses of flight in ratite birds. Proc Natl Acad Sci U S A.

[55] Mitchell KJ, Llamas B, Soubrier J, Rawlence NJ, Worthy TH, Wood J, Lee MS, Cooper A (2014). Ancient DNA reveals elephant birds and kiwi are sister taxa and clarifies ratite bird evolution. Science.

[56] Phillips MJ, Gibb GC, Crimp EA, Penny D (2010). Tinamous and moa flock together: mitochondrial genome sequence analysis reveals independent losses of flight among ratites. Syst Biol.

[57] Smith JV, Braun EL, Kimball RT (2013). Ratite nonmonophyly: independent evidence from 40 novel loci. Syst Biol.

[58] Yonezawa T, Segawa T, Mori H, Campos PF, Hongoh Y, Endo H, Akiyoshi A, Kohno N, Nishida S, Wu J (2017). Phylogenomics and morphology of extinct Paleognaths reveal the origin and evolution of the ratites. Curr Biol.

[59] Sackton TB, Grayson P, Cloutier A, Hu Z, Liu JS, Wheeler NE, Gardner PP, Clarke JA, Baker AJ, Clamp M (2019). Convergent regulatory evolution and loss of flight in paleognathous birds. Science.

[60] Cloutier A, Sackton TB, Grayson P, Clamp M, Baker AJ, Edwards SV (2019). Whole-genome analyses resolve the phylogeny of flightless birds (Palaeognathae) in the presence of an empirical anomaly zone. Syst Biol.

[61] Harrison GL, McLenachan PA, Phillips MJ, Slack KE, Cooper A, Penny D (2004). Four new avian mitochondrial genomes help get to basic evolutionary questions in the late cretaceous. Mol Biol Evol.

[62] Cooper A, Lalueza-Fox C, Anderson S, Rambaut A, Austin J, Ward R (2001). Complete mitochondrial genome sequences of two extinct moas clarify ratite evolution. Nature.

[63] Eberhard JR, Wright TF, Bermingham E (2001). Duplication and concerted evolution of the mitochondrial control region in the parrot genus Amazona. Mol Biol Evol.

[64] Kumazawa Y, Ota H, Nishida M, Ozawa T (1998). The complete nucleotide sequence of a snake (Dinodon semicarinatus) mitochondrial genome with two identical control regions. Genetics.

[65] Kumazawa Y, Ota H, Nishida M, Ozawa T (1996). Gene rearrangements in snake mitochondrial genomes: highly concerted evolution of control-region-like sequences duplicated and inserted into a tRNA gene cluster. Mol Biol Evol.

[66] Arndt A, Smith MJ (1998). Mitochondrial gene rearrangement in the sea cucumber genus Cucumaria. Mol Biol Evol.

[67] Shao R, Barker SC, Mitani H, Aoki Y, Fukunaga M (2005). Evolution of duplicate control regions in the mitochondrial genomes of metazoa: a case study with Australasian Ixodes ticks. Mol Biol Evol.

[68] Cadahia L, Pinsker W, Negro JJ, Pavlicev M, Urios V, Haring E (2009). Repeated sequence homogenization between the control and pseudo-control regions in the mitochondrial genomes of the subfamily Aquilinae. J Exp Zool Part B.

[69] Eda M, Kuro-o M, Higuchi H, Hasegawa H, Koike H (2010). Mosaic gene conversion after a tandem duplication of mtDNA sequence in Diomedeidae (albatrosses). Genes Genet Syst.

[70] Kurabayashi A, Sumida M, Yonekawa H, Glaw F, Vences M, Hasegawa M (2008). Phylogeny, recombination, and mechanisms of stepwise mitochondrial genome reorganization in mantellid frogs from Madagascar. Mol Biol Evol.

[71] Kass RE, Raftery AE (1995). Bayes factors. J Am Stat Assoc.

[72] Tonini J, Moore A, Stern D, Shcheglovitova M, Orti G. Concatenation and species tree methods exhibit statistically indistinguishable accuracy under a range of simulated conditions. PLoS Curr. 2015;7:ecurrents.tol.34260cc27551a527b124ec5f6334b6be. 10.1371/currents.tol.34260cc27551a527b124ec5f6334b6be PMC4391732 25901289

[73] Liu L, Wu SY, Yu LL (2015). Coalescent methods for estimating species trees from phylogenomic data. J Syst Evol.

[74] Chiang CW, Liu CT, Lettre G, Lange LA, Jorgensen NW, Keating BJ, Vedantam S, Nock NL, Franceschini N, Reiner AP (2012). Ultraconserved elements in the human genome: association and transmission analyses of highly constrained single-nucleotide polymorphisms. Genetics.

[75] Katzman S, Kern AD, Bejerano G, Fewell G, Fulton L, Wilson RK, Salama SR, Haussler D (2007). Human genome ultraconserved elements are ultraselected. Science.

[76] Pennacchio LA, Ahituv N, Moses AM, Prabhakar S, Nobrega MA, Shoukry M, Minovitsky S, Dubchak I, Holt A, Lewis KD (2006). In vivo enhancer analysis of human conserved non-coding sequences. Nature.

[77] Kuraku S (2010). Palaeophylogenomics of the vertebrate ancestor-impact of hidden Paralogy on hagfish and lamprey gene phylogeny. Integr Comp Biol.

[78] Feiner N, Begemann G, Renz AJ, Meyer A, Kuraku S (2009). The origin of bmp16, a novel Bmp2/4 relative, retained in teleost fish genomes. BMC Evol Biol.

[79] Kuraku S (2013). Impact of asymmetric gene repertoire between cyclostomes and gnathostomes. Semin Cell Dev Biol.

[80] Gribaldo S, Philippe H (2002). Ancient phylogenetic relationships. Theor Popul Biol.

[81] Maddison WP (1997). Gene trees in species trees. Syst Biol.

[82] Moore WS (1995). Inferring phylogenies from mtDNA variation - mitochondrial-gene trees versus nuclear-gene trees. Evolution.

[83] Page RDM (2000). Extracting species trees from complex gene trees: reconciled trees and vertebrate phylogeny. Mol Phylogenet Evol.

[84] Martin AP, Burg TM (2002). Perils of paralogy: using HSP70 genes for inferring organismal phylogenies. Syst Biol.

[85] Hudson RR, Turelli M (2003). Stochasticity overrules the "three-times rule": genetic drift, genetic draft, and coalescence times for nuclear loci versus mitochondrial DNA. Evolution.

[86] Pamilo P, Nei M (1988). Relationships between gene trees and species trees. Mol Biol Evol.

[87] Brumfield RT, Jernigan RW, McDonald DB, Braun MJ (2001). Evolutionary implications of divergent clines in an avian (Manacus: Aves) hybrid zone. Evolution.

[88] Carling MD, Brumfield RT (2008). Haldane's rule in an avian system: using cline theory and divergence population genetics to test for differential introgression of mitochondrial, autosomal, and sex-linked loci across the Passerina bunting hybrid zone. Evolution.

[89] Rheindt FE, Edwards SV (2011). Genetic introgression: an integral but neglected component of speciation in birds. Auk.

[90] Saetre GP, Borge T, Lindell J, Moum T, Primmer CR, Sheldon BC, Haavie J, Johnsen A, Ellegren H (2001). Speciation, introgressive hybridization and nonlinear rate of molecular evolution in flycatchers. Mol Ecol.

[91] Saetre GP, Borge T, Lindroos K, Haavie J, Sheldon BC, Primmer C, Syvanen AC (2003). Sex chromosome evolution and speciation in Ficedula flycatchers. P Roy Soc B-Biol Sci.

[92] Tegelstrom H, Gelter HP (1990). Haldane rule and sex biased gene flow between 2 hybridizing flycatcher species (Ficedula-Albicollis and F-Hypoleuca, Aves, Muscicapidae). Evolution.

[93] Turelli M, Orr HA (1995). The dominance theory of Haldane's rule. Genetics.

[94] Mckay BD, Zink RM (2010). The causes of mitochondrial DNA gene tree paraphyly in birds. Mol Phylogenet Evol.

[95] Berlin S, Ellegren H (2001). Evolutionary genetics. Clonal inheritance of avian mitochondrial DNA. Nature.

[96] Urantowka AD, Kroczak A, Mackiewicz P (2017). The influence of molecular markers and methods on inferring the phylogenetic relationships between the representatives of the Arini (parrots, Psittaciformes), determined on the basis of their complete mitochondrial genomes. BMC Evol Biol.

[97] Tamashiro RA, White ND, Braun MJ, Faircloth BC, Braun EL, Kimball RT (2019). What are the roles of taxon sampling and model fit in tests of cyto-nuclear discordance using avian mitogenomic data?. Mol Phylogenet Evol.

[98] Powell AF, Barker FK, Lanyon SM (2013). Empirical evaluation of partitioning schemes for phylogenetic analyses of mitogenomic data: an avian case study. Mol Phylogenet Evol.

[99] Meikejohn KA, Danielson MJ, Faircloth BC, Glenn TC, Braun EL, Kimball RT (2014). Incongruence among different mitochondrial regions: a case study using complete mitogenomes. Mol Phylogenet Evol.

[100] Funk DJ, Omland KE (2003). Species-level paraphyly and polyphyly: frequency, causes, and consequences, with insights from animal mitochondrial DNA. Annu Rev Ecol Evol S.

[101] Moum T, Bakke I (2001). Mitochondrial control region structure and single site heteroplasmy in the razorbill (Alca torda; Aves). Curr Genet.

[102] He XL, Ding CQ, Han JL (2013). Lack of structural variation but extensive length polymorphisms and Heteroplasmic length variations in the mitochondrial DNA control region of highly inbred crested Ibis**,*****Nipponia nippon***. PloS one.

[103] Berg T, Moum T, Johansen S (1995). Variable numbers of simple tandem repeats make birds of the order Ciconiiformes Heteroplasmic in their mitochondrial genomes. Curr Genet.

[104] Ball RM, Avise JC (1992). Mitochondrial-DNA Phylogeographic differentiation among avian populations and the evolutionary significance of subspecies. Auk.

[105] Avise JC, Zink RM (1988). Molecular genetic-divergence between avian sibling species - King and clapper rails, long-billed and short-billed Dowitchers, boat-tailed and great-tailed grackles, and tufted and black-crested titmice. Auk.

[106] Mundy NI, Winchell CS, Woodruff DS (1996). Tandem repeats and heteroplasmy in the mitochondrial DNA control region of the loggerhead shrike (Lanius ludovicianus). J Hered.

[107] Qian CJ, Wang YX, Guo ZC, Yang JK, Kan XZ (2013). Complete mitochondrial genome of skylark, Alauda arvensis (Aves: Passeriformes): the first representative of the family Alaudidae with two extensive heteroplasmic control regions. Mitochondrial DNA.

[108] Cho HJ, Eda M, Nishida S, Yasukochi Y, Chong JR, Koike H (2009). Tandem duplication of mitochondrial DNA in the black-faced spoonbill, Platalea minor. Genes & genetic systems.

[109] Slack KE, Janke A, Penny D, Arnason U (2003). Two new avian mitochondrial genomes (penguin and goose) and a summary of bird and reptile mitogenomic features. Gene.

[110] Umeda S, Tang YY, Okamoto M, Hamasaki N, Schon EA, Kang DC (2001). Both heavy strand replication origins are active in partially duplicated human mitochondrial DNAs. Biochem Biophys Res Commun.

[111] Tang Y, Manfredi G, Hirano M, Schon EA (2000). Maintenance of human rearranged mitochondrial DNAs in long-term cultured transmitochondrial cell lines. Mol Biol Cell.

[112] Jiang ZJ, Castoe TA, Austin CC, Burbrink FT, Herron MD, McGuire JA, Parkinson CL, Pollock DD (2007). Comparative mitochondrial genomics of snakes: extraordinary substitution rate dynamics and functionality of the duplicate control region. BMC Evol Biol.

[113] Kuhl H, Frankl-Vilches C, Bakker A, Mayr G, Nikolaus G, Boerno ST, Klages S, Timmermann B, Gahr M. An unbiased molecular approach using 3'UTRs resolves the avian family-level tree of life. Mol Biol Evol. 2020. 10.1093/molbev/msaa191 PMC7783168 32781465

[114] Jiang L, Peng L, Tang M, You Z, Zhang M, West A, Ruan Q, Chen W, Merila J (2019). Complete mitochondrial genome sequence of the Himalayan griffon, Gyps himalayensis (Accipitriformes: Accipitridae): sequence, structure, and phylogenetic analyses. Ecol Evol.

[115] Grealy A, Bunce M, Holleley CE (2019). Avian mitochondrial genomes retrieved from museum eggshell. Mol Ecol Resour.

[116] Huang ZH, Tu FY, Ke DH (2017). Complete mitochondrial genome of blue-throated bee-eater Merops viridis (Coraciiformes: Meropidae) with its taxonomic consideration. Pak J Zool.

[117] Wang HW, Zhang HF, Ren L, Xu Y, Zeng YJ, Miao YL, Luo HY, Wang KH (2016). The whole mitochondrial genome of the lesser kestrel (Falco naumanni). Mitochondrial DNA A.

[118] Singh TR, Shneor O, Huchon D (2008). Bird mitochondrial gene order: insight from 3 warbler mitochondrial genomes. Mol Biol Evol.

[119] Cooke GM, King AG, Johnson RN, Boles WE, Major RE (2012). Rapid characterization of mitochondrial genome rearrangements in Australian songbirds using next-generation sequencing technology. J Hered.

[120] Shi RR, Chen K, Li SB (2017). A novel gene organization of the rock sparrow Petronia petronia (Aves: Passeriformes) revealed by complete mitochondrial genome. Mitochondrial DNA B.

[121] Skujina I, McMahon R, Lenis VPE, Gkoutos GV, Hegarty M (2016). Duplication of the mitochondrial control region is associated with increased longevity in birds. Aging-Us.

[122] Richly E, Leister D (2004). NUMTs in sequenced eukaryotic genomes. Mol Biol Evol.

[123] Bernt M, Braband A, Schierwater B, Stadler PF (2013). Genetic aspects of mitochondrial genome evolution. Mol Phylogenet Evol.

[124] Kumar S, Stecher G, Li M, Knyaz C, Tamura K (2018). MEGA X: molecular evolutionary genetics analysis across computing platforms. Mol Biol Evol.

[125] Edgar RC (2004). MUSCLE: multiple sequence alignment with high accuracy and high throughput. Nucleic Acids Res.

[126] Waterhouse AM, Procter JB, Martin DM, Clamp M, Barton GJ (2009). Jalview version 2--a multiple sequence alignment editor and analysis workbench. Bioinformatics.

[127] Pearson WR, Wood T, Zhang Z, Miller W (1997). Comparison of DNA sequences with protein sequences. Genomics.

[128] Katoh K, Standley DM (2013). MAFFT multiple sequence alignment software version 7: improvements in performance and usability. Mol Biol Evol.

[129] Talavera G, Castresana J (2007). Improvement of phylogenies after removing divergent and ambiguously aligned blocks from protein sequence alignments. Syst Biol.

[130] Nguyen LT, Schmidt HA, von Haeseler A, Minh BQ (2015). IQ-TREE: a fast and effective stochastic algorithm for estimating maximum-likelihood phylogenies. Mol Biol Evol.

[131] Ronquist F, Teslenko M, van der Mark P, Ayres DL, Darling A, Hohna S, Larget B, Liu L, Suchard MA, Huelsenbeck JP (2012). MrBayes 3.2: efficient Bayesian phylogenetic inference and model choice across a large model space. Syst Biol.

[132] Lartillot N, Philippe H (2004). A Bayesian mixture model for across-site heterogeneities in the amino-acid replacement process. Mol Biol Evol.

[133] Chernomor O, von Haeseler A, Minh BQ (2016). Terrace aware data structure for Phylogenomic inference from Supermatrices. Syst Biol.

[134] Kalyaanamoorthy S, Minh BQ, Wong TKF, von Haeseler A, Jermiin LS (2017). ModelFinder: fast model selection for accurate phylogenetic estimates. Nat Methods.

[135] Lanfear R, Calcott B, Ho SY, Guindon S (2012). Partitionfinder: combined selection of partitioning schemes and substitution models for phylogenetic analyses. Mol Biol Evol.

[136] Huelsenbeck JP, Larget B, Alfaro ME (2004). Bayesian phylogenetic model selection using reversible jump Markov chain Monte Carlo. Mol Biol Evol.

[137] Shimodaira H, Hasegawa M (2001). CONSEL: for assessing the confidence of phylogenetic tree selection. Bioinformatics.

[138] Maddison WP, Maddison DR: **Mesquite: a modular system for evolutionary analysis.** . In*.*, Version 3.31 edn; 2017.

