# Supplementary material for: New view on the organization and evolution of Palaeognathae mitogenomes poses the question on the ancestral gene rearrangement in Aves
Source: BMC Genomics. 2020 Dec 7;21:874. doi: 10.1186/s12864-020-07284-5 (PMC7720580; doi:10.1186/s12864-020-07284-5)

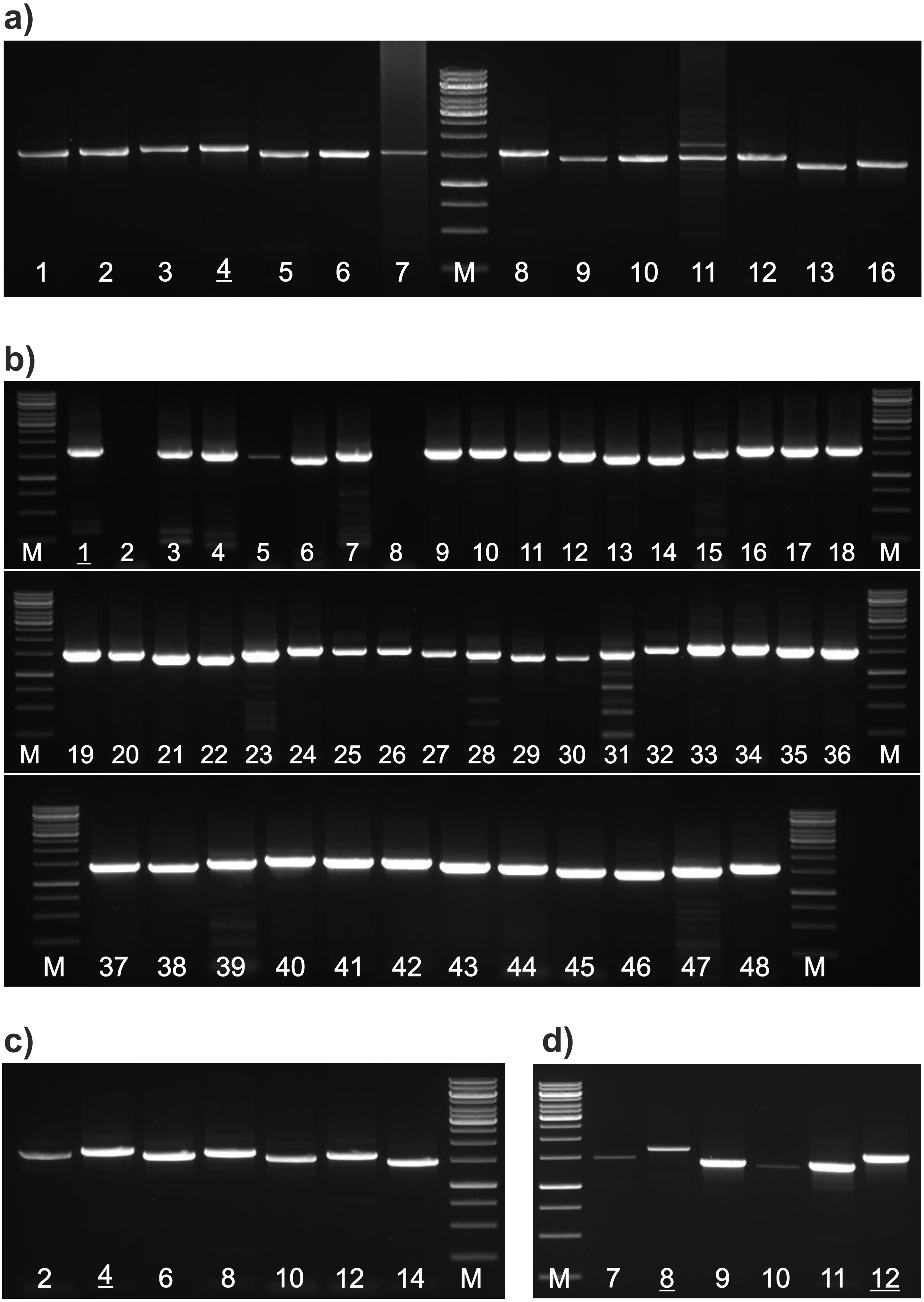

Figure S1. Representative cropped agarose gels of CR1/CR2 amplicons obtained for *Struthio camelus* (a), *Rhea pennata* (b), *Rhea americana* (c) and *Crypturellus tataupa* (d). The numbering of amplicons separated in the agarose gel corresponds to the reaction numbered in Table S1. The numbers of PCR fragments, which were finally sequenced, are underlined. Lane M - GeneRuler 1 kb DNA Ladder (Thermo Scientific). Original uncropped and unprocessed gels are presented in Fig. S13a-d.

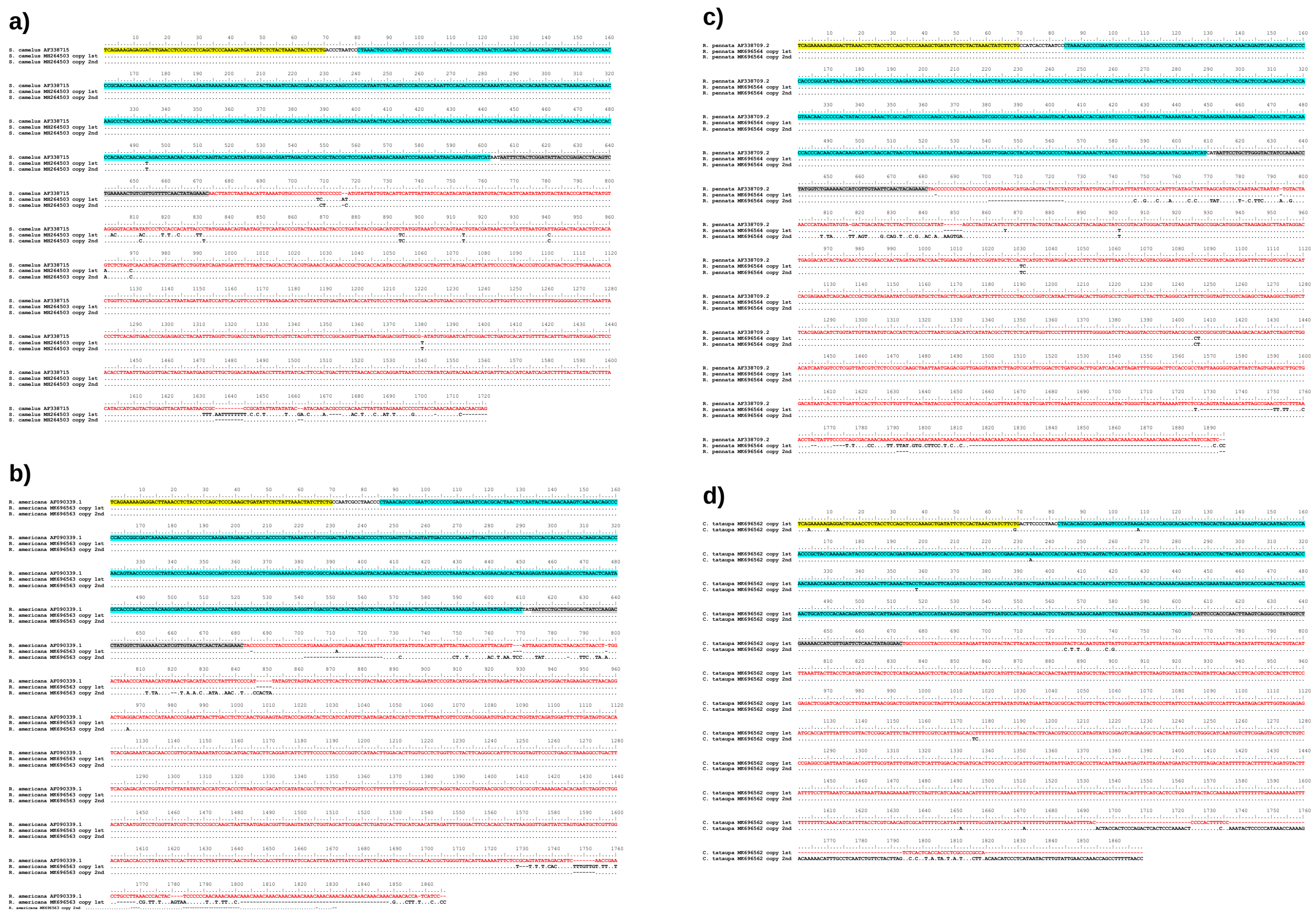

Figure S2. Sequence comparison of two copies of *tRNA-Pro*, *ND6*, *tRNA-Glu* genes and control region, found in this study as duplicated, with appropriate sequences of mitogenomes previously deposited in GenBank and showing a typical avian gene order. The alignments are shown for *Struthio camelus* (a), *Rhea americana* (b), *Rhea pennata* (c) and *Crypturellus tataupa* (d). Dots indicate residues identical in the obtained copies with those in the single sequence previously published. Genes for tRNA-Pro are marked in yellow coloring, NADH dehydrogenase subunit 6 in cyan coloring, tRNA-Glu in grey shading, and control regions in red fonts. In the case of *Crypturellus tataupa* only two obtained copies are compared to each other because the previously published mitogenome of these taxa is incomplete and it does not contain any of the analyzed genes or the control region. The sequences were aligned with MUSCLE [125] in MEGA [124].

***Struthio camelus*: tRNA-Leu/16S rRNA (MH264503)**

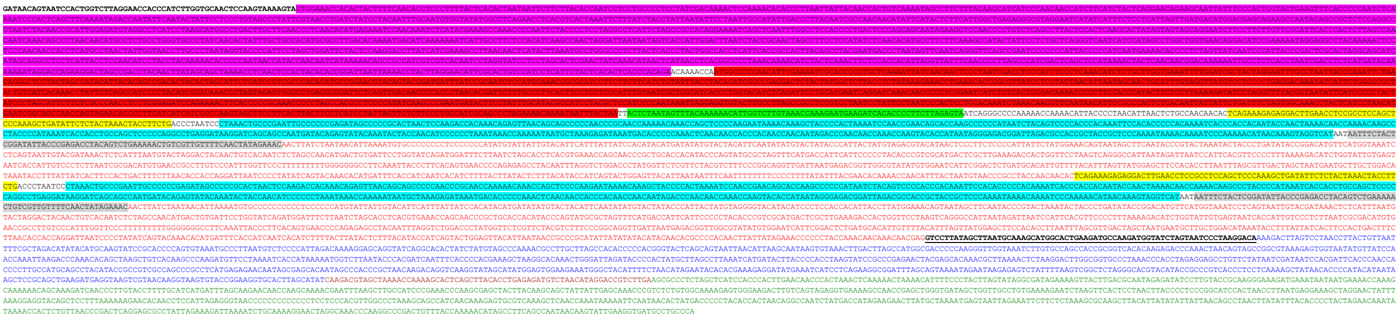

***Rhea americana*: ND5/16S rRNA (MK696563)**

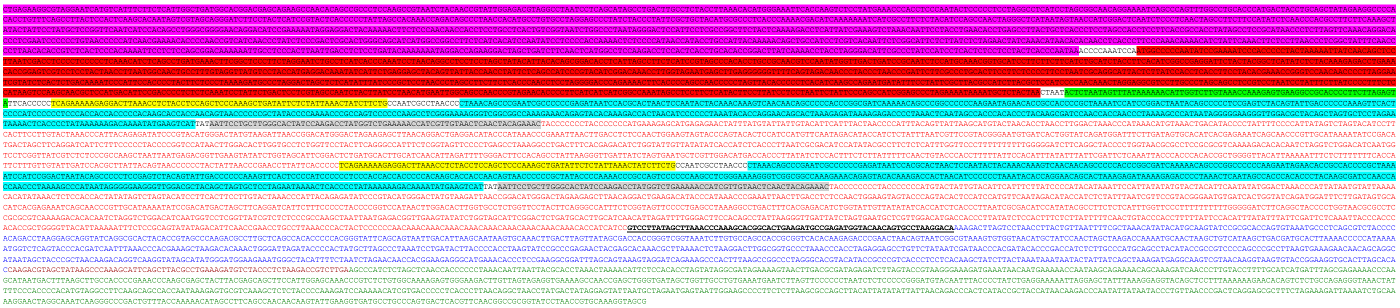

***Rhea pennata*: ND5/16S rRNA (MK696564)**

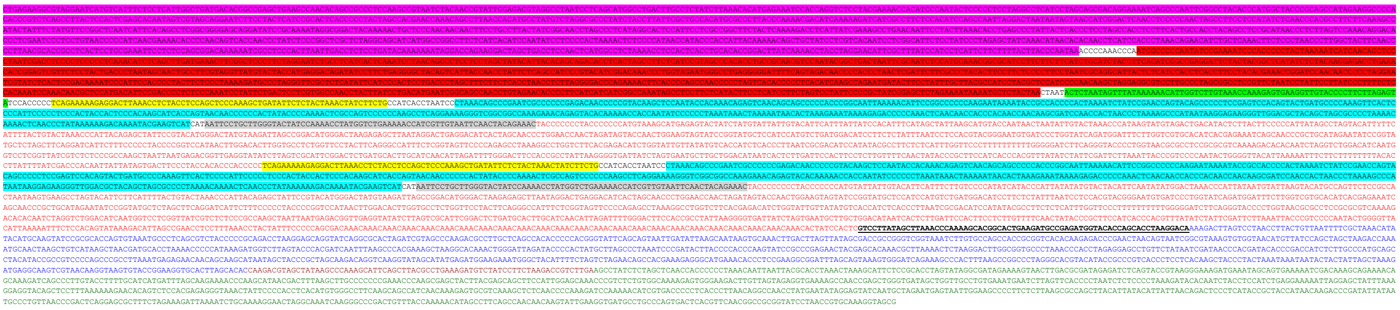

***Crypturellus tataupa*: ND5/12S rRNA (MK696562)**

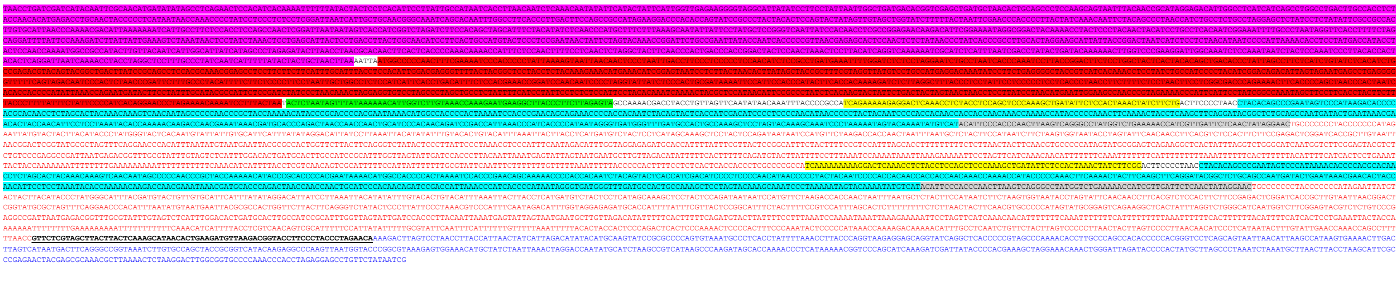

Figure S3. Sequences of mitogenomic fragments amplified and sequenced in this study for *Struthio camelus* (MH264503), *Rhea americana* (MK696563), *Rhea pennata* (MK696564) and *Crypturellus tataupa* (MK696562). **Genes for tRNA-Leu are marked in bold**, NADH dehydrogenase subunit 5 in magenta coloring, cytochrome b in red coloring, tRNA-Thr in green coloring, tRNA-Pro in yellow coloring, NADH dehydrogenase subunit 6 in cyan coloring, tRNA-Glu in grey shading, 12S rRNA in blue fonts, tRNA-Val in brown fonts, 16S rRNA in green fonts, **tRNA-Phe are in bold and underlined**, and control regions in red fonts.

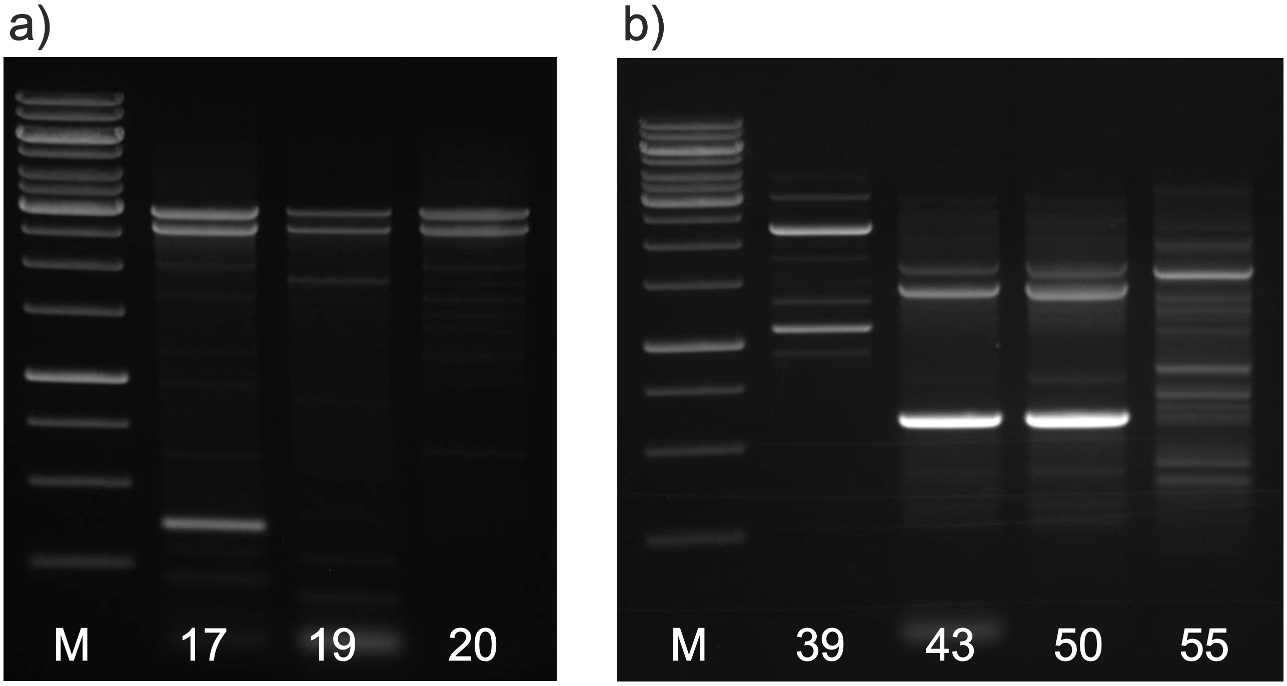

Figure S4. Cropped agarose gels of potential NUMTs amplified for *Dromaius novaehollandiae* (a) and *Casuarius casuarius* (b) with the use of primers designed for detection of CR1/CR2 fragments. The numbering of lanes corresponds to the reaction numbered in Table S1. Lane M - GeneRuler 1 kb DNA Ladder (Thermo Scientific). Original uncropped and unprocessed gels are presented in Fig. S13e and f.

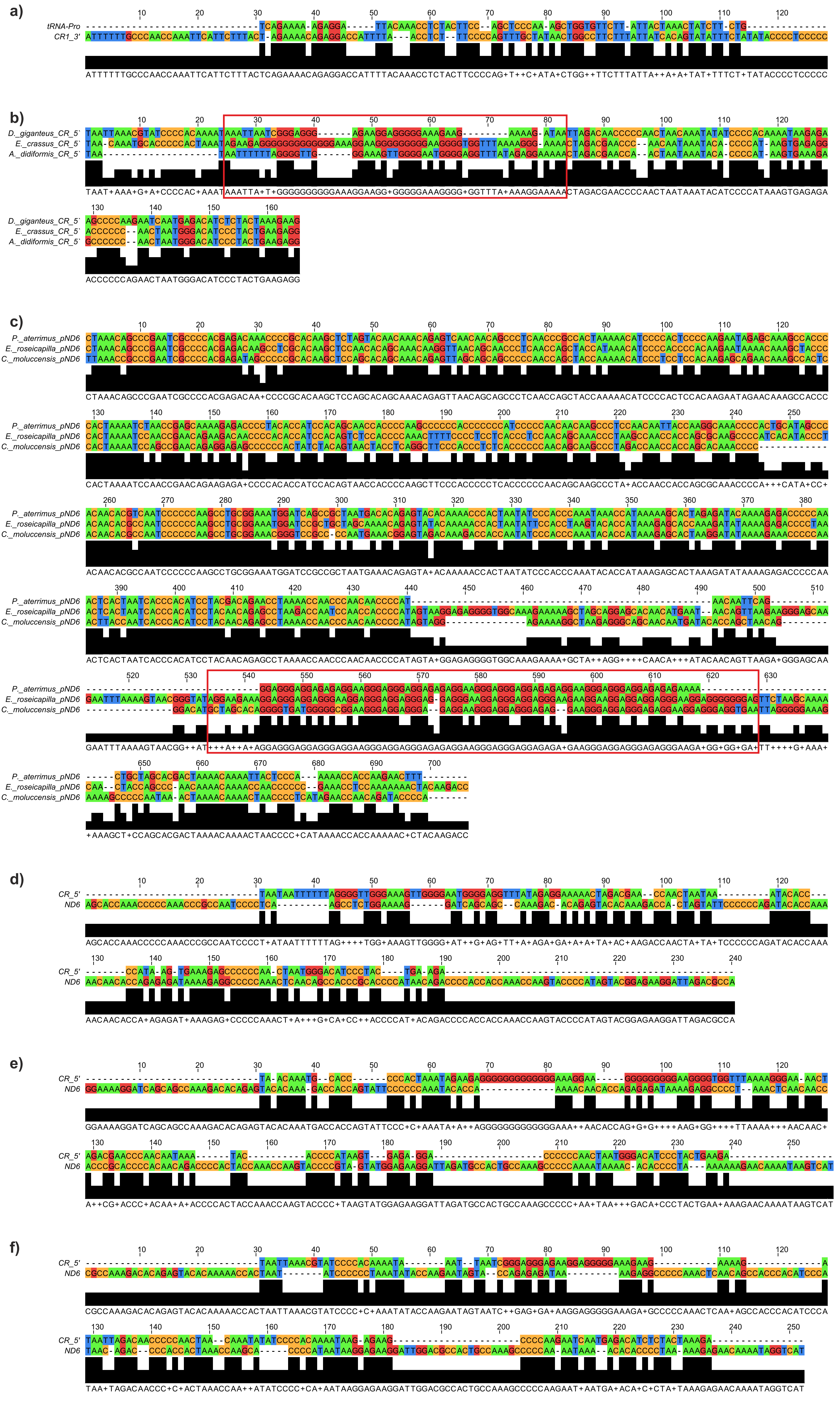

Figure S5. Comparison of selected sequence associated with reannotation of Palaeognathae mitogenomes. *Eudromia elegans* gene for tRNA-Pro with the last 200 nucleotides of the control region (a); *Emeus crassus*, *Dinornis giganteus* and *Anomalopteryx didiformis* control region 5' spacers (b); *Probosciger aterrinus* (MH133970.1), *Eolophus roseicapilla* (MH133971.1) and *Cacatua moluccensis* (MH133972.1) *ND6* pseudogenes (c); *Anomalopteryx didiformis ND6* gene with control region 5' spacer (d); *Emeus crassus ND6* gene with control region 5' spacer (e); *Dinornis giganteus ND6* gene with control region 5' spacer (f). Sequences rich in G and A residues are marked in a red box. The sequences in b and c were aligned with MUSCLE [125] in MEGA [124], whereas those in a, d, e and f using the optimal global:local algorithm (glsearch) form FASTA package version 36.3.8g[127].

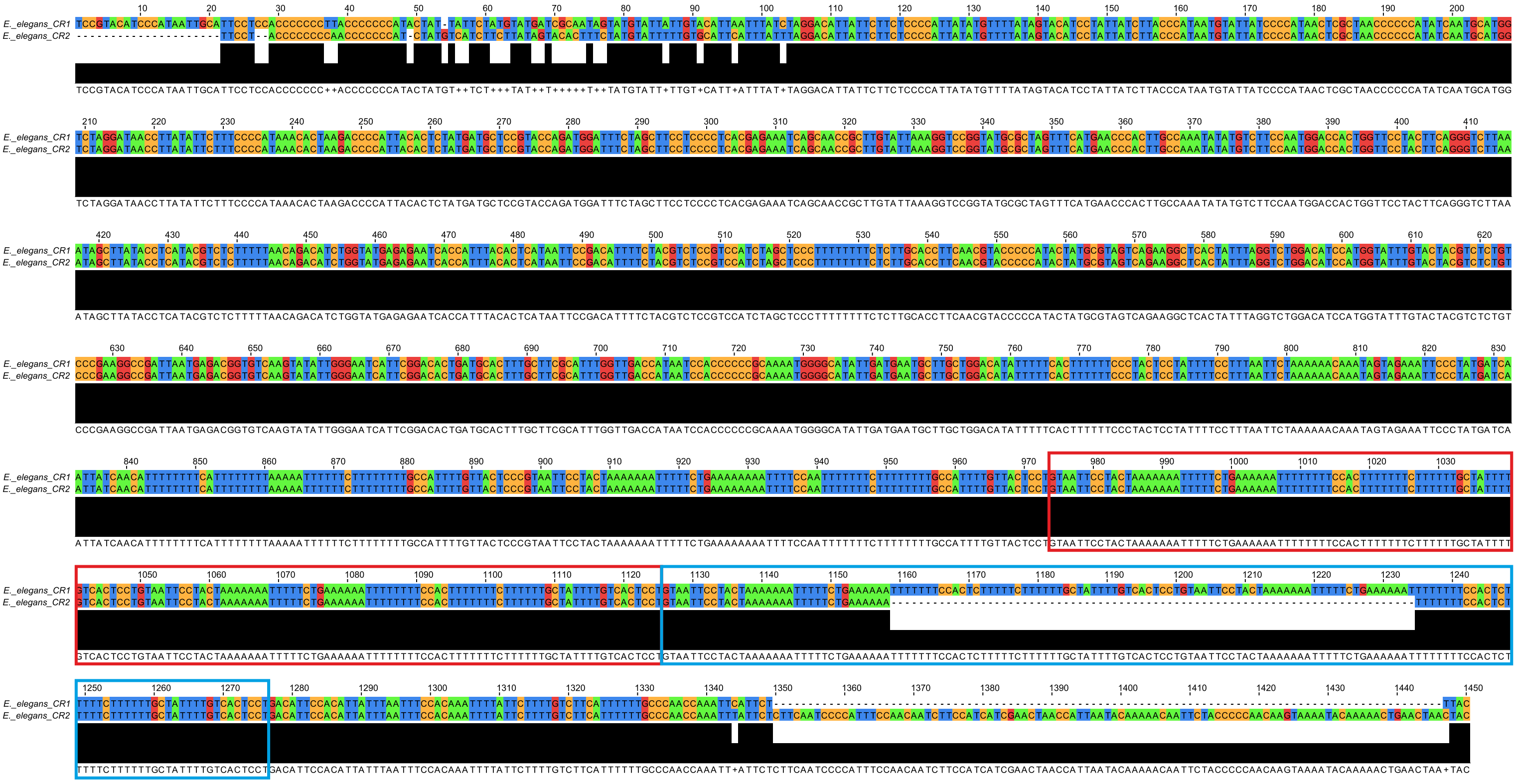

Figure S6. Sequence comparison of two *Eudromia elegans* control regions. Dots indicate residues identical in the compared copies. Dots indicate residues identical in the compared sequences. Sequence repeats present in both control regions are marked in red and blue boxes.The sequences were aligned with MUSCLE[125] in MEGA[124].

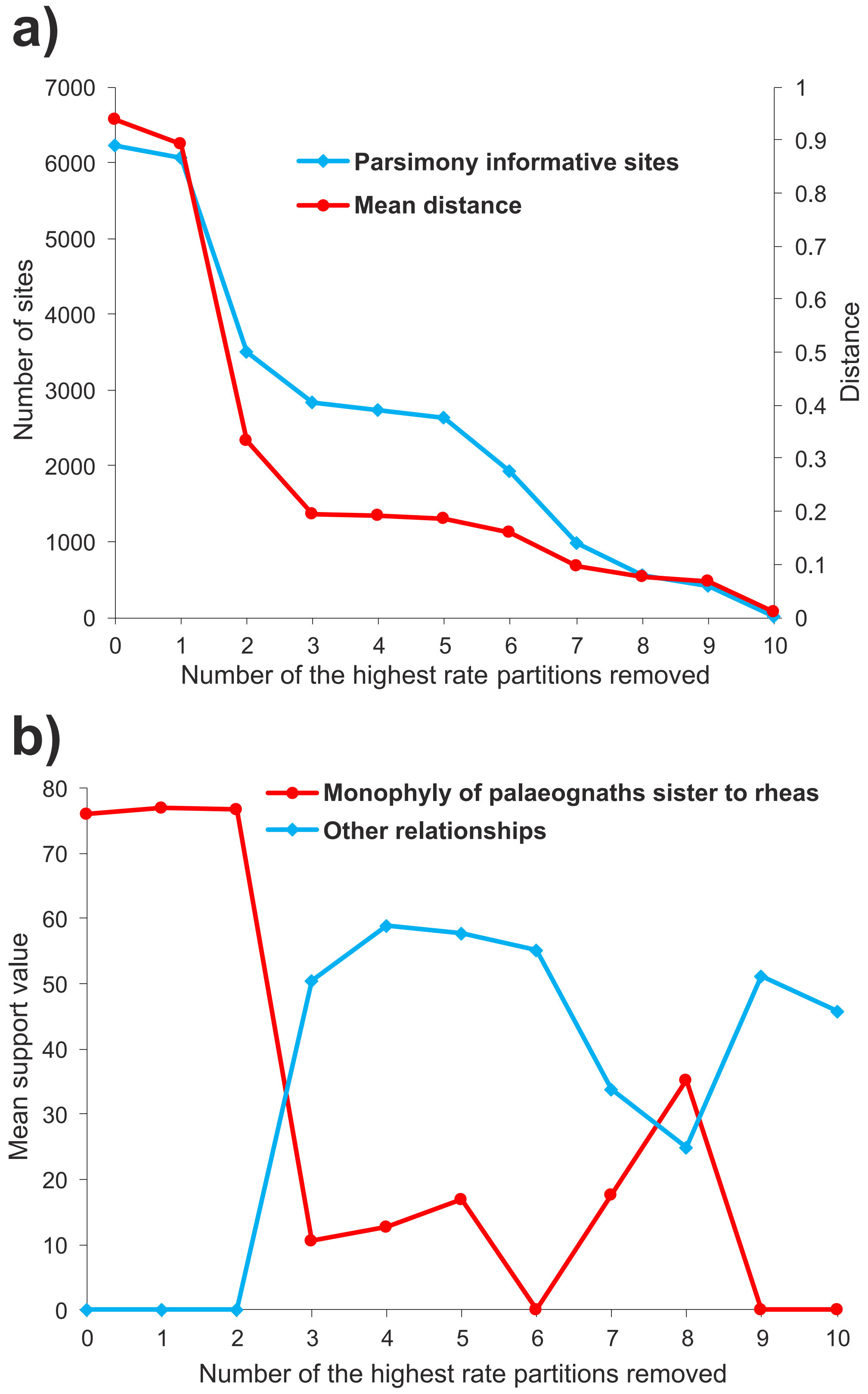

Figure S7. Influence of removing partitions with the highest substitution rate on alignment and tree parameters: the number of parsimony informative sites and mean distance (a) as well as mean support values (b). The mean phylogenetic distance was obtained from MrBayes tree. The mean support values were calculated from posterior probabilities received in MrBayes and PhyloBayes as well as SH-aLRT and non-parametric bootstrap percentages obtained in IQ-TREE. The posterior probabilities were scaled to 100%.

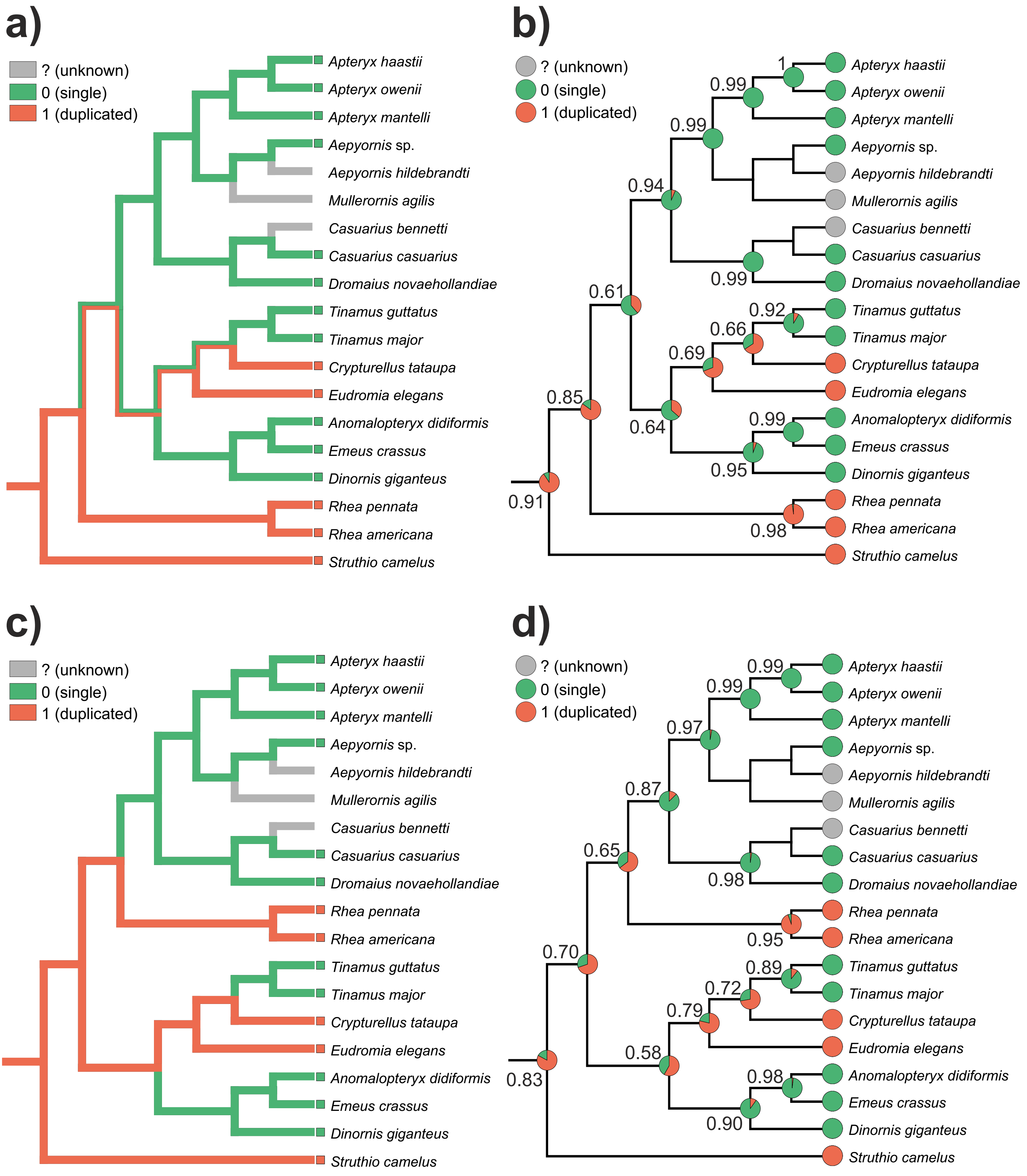

Figure S8. Maximum parsimony (a, c) and maximum likelihood (b, d) reconstruction of ancestral states and mapping of mitogenomic duplications onto the Palaeognathae tree topology t1 based on sequences of mitochondrial genes (a, b) and the Palaeognathae tree topology t2 supported by nuclear markers in coalescent-based approaches (c, d). In contrast to Fig. 6, this approach assumes that *Anomalopteryx didiformis*, *Emeus crassus* and *Dinornis giganteus* already lost mitogenomic duplication. The area of colors at nodes in b and d corresponds to the probability of the given state, single or duplicated region. Two-colored branches correspond to the equal probability of two states, single or duplicated region. The probability value for a more likely state was also given at these nodes. Mk1 model was applied for ML approach.

Figure S9. Annotated and reannotated duplicated gene orders in mitogenomes of Neoaves and Galloanserae deposited in GenBank for: *Alca torda* (CM018102.1), *Antrostomus carolinensis* (MN356120.1), *Calypte anna* (CM016612.1), *Cariama cristata* (CM020379.1), *Catharus ustulatus* (CM020378.1), *Colius striatus* (MN356125.1), *Ketupa blackistoni* (LC099104), *Ketupa flavipes* (LC099100.1), *Leptosomus discolour* (MN356135.1), *Merops nubicus* (CM020464.1), *Melanereps aurifrons* (CM022134.1), *Morus serrator* (GU071056.1), *Nyctibius grandis* (CM023771.1), *Pterocles burchelli* (MN356340.1), *Pterocles gutturalis* (CM020177.1, MN356147.1), *Puffinus lherminieri* (MH206162.1), *Rynchops niger* (MN356248.1), *Sterna hirundo* (CM020500.1), *Thalassarche chlororhynchos* (MN356342.1), *Uria aalge* (MN356418.1) and *Urocolius indicus* (MN356373.1). Genes for NADH dehydrogenase subunit 5 are marked in magenta coloring, cytochrome b in red coloring, tRNA-Thr in green coloring, tRNA-Pro in yellow coloring, NADH dehydrogenase subunit 6 in cyan coloring, tRNA-Glu in grey shading, 12S rRNA in blue fonts, **tRNA-Phe in bold fonts**, and control regions in red fonts. *Pseudogenes are in italics and underlined*. Microsatellites present within *Pterocles gutturalis* mitogenomes between *tRNA-Thr* gene and *tRNA-Pro* pseudogene or *tRNA-Thr* gene and *ND6* pseudogene are marked in brown.

Alca torda **(Charadriiformes/Alcidae) mitogenome from ND5 to 12S rRNA (CM018102.1 - annotated)**

GO-FD in Figure 8

[illegible]

Antrostomus carolinensis **(Caprimulgiformes/Caprimulgidae) mitogenome from ND5 to 12S rRNA (MN356120.1 - re-annotated)**

GO-IV in Figure 8

[illegible]

Calypte anna **(Caprimulgiformes/Trochilidae) mitogenome from ND5 to 12S rRNA (CM016612.1 - annotated)**

GO-FD in Figure 8

[illegible]

ATTTGCGCACTAACTCAAAACGACATTAAAAAAATCATTGCCTTCTCTACATCAAGCCAATTAGGACTAATAATAGTCACCATTGGATTAAACCTCCCCCAACTCGCCTTCCTACACATCTCAACCCATGCTTTCTTTAAGGCCATACTATTCCTATGCTCCGGTTCAATCATC
CACAGTCTCAACGGAGAACAGGACATCCGAAAAATAGGAGGATTGCAAAAAACACTTCCAACAACAACCTCCTGCCTAACCATCGGCAACCTCGCCCTAATAGGAACCCCATTCCTAGCTGGATTCTACTCAAAAGACTTAATCATCGAAAACCTAAACACCTCATACCTAAAC
TCCTGAGCCCTACTACTAACCCTACTGGCCACCTCATTCACTGCAACCTATAGCACTCGCATAGCCATCCTAGTCCAAACCAACTTCACACGAATCACAACACTCACACCCATGAACGAAAATGACACCACAATCACGTCCCCAATCCTACGCCTCGCCTTCGGCAGCATCCTA
GCTGGATTAATCATCACATCCTACCTAGTCCCCTGCAAAACCCCGCCAATAACCATACCAATAATTACAAAAACTGCAGCCCTAATCGTCACAATAATAGGAATCATTCTGGCCCTAGAACTATCAAACACAAACCTGACCTTAACCCAACCAAAACAAAACACATACCTAAAC
TTCTCCTCCTCGCTAGGATATTTCAACCCACTAACCCACCGGCTCCTATCAAAAATTCTACTAAACAACGGACAAAAAATCGCATCCCACTTAATCGACCTGTCTTGATACAAAAAAATAGGGCCCGAAGGCCTAGCCGACCTACAACTAGCAGCGTCCAAAACCTCTACCTCC
ATGCACTCAGGCTTAATCAAAACATACCTTGGCTCATTCGCCCTATCAATCCTAATCATTCTCCTATCAACATAAACCAAACACCTAATGGCCCCAAACCTACGCAAATCCCACCCCCTTCTCAAAATAGTTAACGACATGCTAATCGACCTTCCTGCCCCATCCAACATCTCA
ACCTGATGAAACTTCGGATCCCTCCTAGGCCTATGCTTAATAACACAAATCCTAACAGGCCTCTTGCTCGCCATACACTACACCGCAGACACTACCTTAGCTTTCACATCAGTCGCCCACACCTGCCGAAACGTTCAATACGGATGACTGATCCGAAACCTCCATGCAAATGGC
GCCTCATTCTTCTTCATCTGCATCTACCTACACATTGGACGAGGATTCTACTACGGATCCTACCTATACAAAGAAACCTGAAACACGGGAGTAATCCTACTACTCACCCTAATAGCTACAGCTTTCGTAGGCTATGTACTACCATGAGGGCAAATATCATTCTGAGGAGCCACA
GTAATTACCAACCTATTCTCAGCCATCCCCTATATCGGACAAACCTTGGTAGAATGAGCATGAGGTGGATTCTCCGTAGACAACCCAACACTGACCCGATTCTTTGCCCTTCACTTCCTTCTTCCTTTCATAATCGCAGGACTTACGTTCATTCACCTAACCTTCCTTCACGAA
ACTGGCTCCAACAACCCCCTAGGCATTACATCAGACTGCGACAAAATCCCGTTCCACCCCTACTTCTCCACAAAAGACATCCTAGGCTTCCTATTAATAATCACCCCCCTACTGACCCTAGCCATATTCTCCCCAAACCTCCTAGGAGATCCAGAAAACTTCACCCCAGCCAAC
CCGCTAGTCACACCCCCACACATCAAGCCAGAATGATACTTTTTATTTGCATACGCCATCCTACGATCAATCCCAAATAAACTAGGAGGCGTCCTAGCCTTAGCTGCCTCCGTCCTAGTCCTATTTTTAGCCCCTTCCTCCATAAATCAAAACAACGCACAATAACATTCCGA
CCTCTCTCACAACTTCTATTCTGAATCCTCGTAGCAAACCTTTTCATCCTAACCTGAGTTGGCAGCCAACCAGTAGAACACCCATTCATCATCATTGGCCAAATCGCATCCTTAACCTACTTCACAATCCTCCTAATCCTCTTCCCCCTCACAAGCGCCCTAGAAAACAAAATA
CTCAACTACTAATAATACTCTAATAGTTTATAAAAACATTGGCCTTGTAAGCCAAAGAATGAAGACTATACCCCTTCTTAGAGTTTCGCCCCAACTATCAGAAAAAAAGGACTCAAACCTCTATCTCCAACTCCCAAAGCTGGCATTTTCCATTAAACTATTTTCTGGCCCCCC
CCCGGCCGCTTTAAACCGCCCGAATGGCCCCACGGGATAAGCCCCGCACAAGCTCTAAAACAACAAACAATGTCAGCAACAACCCCCACCCCGCAACTAAAAACAATCCTGCCCCCCAAGAATAAAAAAGCGCCGCCCCGCCAAAATCCATACGAACAAAAAACATCTCCACTC
CATCAACAGTCCCCACCCCAAACTTCCAATCCATAGCAGGACCCCCCACTACTACCCCTAAAACTAGAACTAACACAAAACCAAATCCATATCCCATAACCCGCCAATCTCCCCAAGTCTCTGGAAATGGATCTGCAGCCAGAGACACTGAATAGACAAAGACCACCAACATCC
CCCCCAAATAAACCATAAATAACACCAACGACACAAAAGAAAGCCCAAGACTTATTAACCACCCACAGCCAACAATAGAAGACAGTACCAAACCTACAACTCCATAGTAAGGAGATGGATTAGACGCAACTGCCAACCCCCCTAATACGAAACACAACCCCAAAAACAACATAA
AATATATCATAATTTCTGCCTGGCCTCTATCCAAGCCCTATGGCTTGAAAAGCCATCGTTGTCTTCCTCAACTACAGAAACAACTTCGTATACCCCCCCTCCCCCCATATTATGGGGACTCTTCTAATTCATTGCTATGTACGCTTTGCATTAACTATTATTGTCCACATTCAA
TTAATTAATGTATATCCAAATAACTACTATAACAGTACATAACCCATTTATAATCATATAGTACATAAACCATGACAACTAAGATAGACATAAGATTGTCTTAAATTCATACCTACCCACGTAAATTTACTGTAATGACCTATCCAACAGTACAACTCTATCAATACTAAAACC
ATAAAGTCCTGAAATCAACTGAAGCAGTCTTTACCATACGAAGGGGCTCTCAAGTACATACGTTTCAGTGGTCACTCACCAGAATAGTGCGAACAACTCTTGAAGTACTTACAACCAAAGTACTAGGTTATCTATTAGTCGGTCTTCTCACGAGAAATCAGCAACGCACCGCAT
AAAATGTCCTAAGTCACTAGCTTCAAGTGCATTCTTCCCCCCTAAACCCTAGCACAACTCGCACTTTTGCGCTACTGGTTCCTATGTCAGGGCCATAACAAGATAATTCCCCAAAGAATTCTCTTCAAAAGGCATTTGGTTGGCGTCTCGTATTTCATTCTATTCTTGGCCGGG
TCACCTTCCTTTTTAGCGCGGTTGGTTCTTTTTTTTTTCTGGATAACTTCAACGGGCCCTCCGGTGGCTAACGCGGAGCATACAATCTCTTGACCTGAGCATCGGTGGCCCCCGGGCCTTTTCTCTCCTTCAAGGATTGCTTAATGATACGGTTTCATGTGTTATCGATCTCAT
TTTAGCACTGATGCACTTTGGATAGCATTTGGTTATGGTATTCTCACATACCTACTAAGCTATGTTGCTTTTTAGTTTATGGTCTCCGGACATATTTTACAACTTTTACACTTCCTCTAACTTTCTAACCAACACCACTGGAGTTCTAAGTTATAATTTTATCATCATTCATCA
TATTTTCATCGTGTATTTTATTCTTCAATATTAATCTATATTCAACACCGGGGTTACATTAAAAAGTTAACACCGTTTGTCATCGTGTTTATCATGTTAAATTTCACACACAAACACCATAAAATTACTAATGGAACCCCCCTAGAAAAATTCAAGACAACCAAAATCAAAACA
ACGCACAATAACATTCCGACCTCTCTCACAACTTCTATTCTGAATCCTCGTAGCAAACCTTTTCATCCTAACCTGAGTTGGCAGCCAACCAGTAGAACACCCATTCATCATCATTGGCCAAATCGCATCCTTAACCTACTTCACAATCCTCCTAATCCTCTTCCCCCTCACAAG
CGCCCTAGAAAACAAAATACTCAACTACTAATAATACTCTAATAGTTTATAAAAACATTGGCCTTGTAAGCCAAAGAATGAAGACTATACCCCTTCTTAGAGTTTCGCCCCAACTATCAGAAAAAAAGGACTCAAACCTCTATCTCCAACTCCCAAAGCTGGCATTTTCCATTA
AACTATTTTCTGGCCCCCCCGGCCGCTTTAAACCGCCCGAATGGCCCCACGGGATAAGCCCCGCACAAGCTCTAAAACAACAAACAATGTCAGCAACAACCCCCACCCCGCAACTAAAAACAATCCTGCCCCCCAAGAATAAAAAAGCGCCGCCCCGCCAAAATCCATACGAAC
AAAAAACATCTCCACTCCATCAACAGTCCCCACCCCAAACTTCCAATCCATAGCAGGACCCCCCACTACTACCCCTAAAACTAGAACTAACACAAAACCAAATCCATATCCCATAACCCGCCAATCTCCCCAAGTCTCTGGAAATGGATCTGCAGCCAGAGACACTGAATAGAC
AAAGACCACCAACATCCCCCCCAAATAAACCATAAATAACACCAACGACACAAAAGAAAGCCCAAGACTTATTAACCACCCACAGCCAACAATAGAAGACAGTACCAAACCTACAACTCCATAGTAAGGAGATGGATTAGACGCAACTGCCAACCCCCCTAATACGAAACACAA
CCCCAAAAACAACATAAAATATATCATAATTTCTGCCTGGCCTCTATCCAAGCCCTATGGCTTGAAAAGCCATCGTTGTCTTCCTCAACTACAGAAACAACTTCGTATACCCCCCCTCCCCCCATATTATGGGGACTCTTCTAATTCATTGCTATGTACGCTTTGCATTAACTA
TTATTGTCCACATTCAATTAATTAATGTATATCCAAATAACTACTATAACAGTACATAACCCATTTATAATCATATAGTACATAAACCATGACAACTAAGATAGACATAAGATTGTCTTAAATTCATACCTACCCACGTAAATTTACTGTAATGACCTATCCAACAGTACAACT
CTATCAATACTAAAACCATAAAGTCCTGAAATCAACTGAAGCAGTCTTTACCATACGAAGGGGCTCTCAAGTACATACGTTTCAGTGGTCACTCACCAGAATAGTGCGAACAACTCTTGAAGTACTTACAACCAAAGTACTAGGTTATCTATTAGTCGGTCTTCTCACGAGAAA
TCAGCAACGCACCGCATAAAATGTCCTAAGTCACTAGCTTCAAGTGCATTCTTCCCCCCTAAACCCTAGCACAACTCGCACTTTTGCGCTACTGGTTCCTATGTCAGGGCCATAACAAGATAATTCCCCAAAGAATTCTCTTCAAAAGGCATTTGGTTGGCGTCTCGTATTTCA
TTCTATTCTTGGCCGGGTCACCTTCCTTTTTAGCGCGGTTGGTTCTTTTTTTTTTCTGGATAACTTCAACGGGCCCTCCGGTGGCTAACGCGGAGCATACAATCTCTTGACCTGAGCATCGGTGGCCCCCGGGCCTTTTCTCTCCTTCAAGGATTGCTTAATGATACGGTTTCA
TGTGTTATCGATCTCATTTTAGCACTGATGCACTTTGGATAGCATTTGGTTATGGTATTCTCACATACCTACTAAGCTATGTTGCTTTTTAGTTTATGGTCTCCGGACATATTTTACAACTTTTACACTTCCTCTAACTTTCTAACCAACACCACTGGAGTTCTAAGTTATAAT
TTTATCATCATTCATCATATTTTCATCGTGTATTTTATTCTTCAATATTAATCTATATTCAACACCGGGGTTACATTAAAAAGTTAACACCGTTTGTCATCGTGTTTATCATGTTAAATTTCACACACAAACACCATAAAATTACTAATGAAACCCCCCTAGAAAAATTTAAGA
CACCACAAACCCATCCCAACAAACAAACAAACAAACAAACAAACAAACAAACAAACAAACAAACAAACAAACAAACAAACAAACAAACAAACAAACAAACAAACAAACAAACAAACAAACAAACAAACAAACAAACAAACAAACAAACAG
CCACCCCAAACT**GTCCATGTAGCTTAGCCAAAGCATAGCACTGAAGATGCTAAGACGACACTAATAAAATGTCCATGGACA**AAAGACTCAGTCCTAACCTTACCGTTGATTGTTACCAAACATATACATGCAAGTATCCGCGCCCCAGTGTAAATGCCCTCAGACACCTTACCA
AGACAAAAGGAGCAGGTATCAGGCACACCACGACTGTAGCCCAAAACACCTTGCTCAGCCACACCCCCACGGGTACTCAGCAGTAATTAACATTAAGCAATAAGTGTAAACTTGACTTAGTTATGGTAAACCTAAGGGCTGGTTAATCTTGTGCCAGCCACCGCGGTCATACAA
GAAACCCAAACCAACGGCAGCACGGCGTAAAGAGTGACATGACAATATCCTCCAAGCTAAGATCGAAACACAACTGAGCTGTCATAAGCACAAGATGTGCAGAAGAACCCCAAAAAGAAGGTCCTAGCTAACACGACTTAATTACTGTCACGAAAGCTAAGACACAAACTGGGA
TTAGATACCCCACTATGCTTAGCCCTAAATCTTGACACTTCCCCGACCAAAGTGTCCGCCTGAGAACTACGAGCACAAACGCTTAAAACTCTAAGGACTTGGCGGTGCCTCAAACCCACCTAGAGGAGCCTGTTCTATAATCGATAACCCACGCTACACCCGGCCGCTCTTTGC
ACATTCAGCCTACATACCGCCGTCGCCAGTCCACCTATCCTGAAAGAACAACAGTGAACGCAATAGCCTTAACCACGCTAAAAAGACAGGTCAAGGTATAGCCAATAGAGCGGAAGAAATGGGCTACATTTTCTAACTTAGAAAACTACGAAAGGGGACATGAAACCACCCCCA
GAAGGCGGATTTAGCAGTAAAGTTGGATCATAGTGCCATCTTTAAACCGGCCCCGGGGCACGTACATACCGCCCGTCACCCTCCTCGCAAGCTACTCACATGAGTAATTAACACTAACTCCTAGCCAAAGATGAGGTAAGTCGTAACAAGGTAAGTGTACCGGAAGGTGCACTT
AGCACAC

Cariama cristata (Cariamiformes/Cariamidae) mitogenome from ND5 to 12S rRNA (CM020379.1 - annotated)

GO-FD in Figure 8

ATGGAGACCACACTACTCCTCCACACCTCCATAACTCTCACGCTCATCACCATCCTTACCCCGATACTACTCCCACTACTATCAAAAACAATGCAAAGCTCCCCAACCATTGTCACACATGCAGTAAAAACTGCCTTCCTAATAAGCCTCGTACCCATAACACTCTTCATATAC
TCAGGCACAGAAAACATTATCACCTACTGAGAGTGAAAATTTATGATAAACTTTAAAATCCCCCTCAGCCTAAAAATAGACCAATACTCAATAATATTCTTCCCAATCGCACTGTTCGTAACCTGATCCATCCTTCAATTCGCCACATGGTACATAGCCTCAGACCCACACATC
ACAAAATTCTTCTCCTACCTCCTAGCATTCCTAATTGCCATACTAATTCTGACAACCGCTAACAACATATTTCTACTATTCATTGGCTGAGAAGGAGTAGGAATCATGTCCTTCCTATTAATCGGCTGATGACATGGTCGGGCAGAAGCCAACACAGCCGCCCTACAAGCCGTA
ATTTACAACCGAATCGGAGATGTAGGCCTTATCCTAAGCATAGCCTGACTTGCTTCCTCTACAAGCACATGAGACATCCATCAAGCCCCCACTCAACCTACAACACTCCCCCTCCTAGGGCTCATTCTAGCAGCTGCCGGAAAATCCGCCCAATTTGGGCTCCACCCATGACTA
CCCGCGGCCATAGAAGGCCCTACGCCAGTCTCAGCCCTACTCCACTCAAGCACTATAGTAGTTGCCGGAATTTTCCTACTCATCCGCACCCACCAAATATTTACCAATAACCAAATCGCCCTCACTTTATGTCTGTGCTTAGGGGCCTATCCACCCTATTTGCCGCCACATGC
GCCCTCACACAAAATGACATCAAAAAAATCATTGCTTTCTCCACATCAAGCCAACTAGGACTAATAATAGTCACCATTGGATTAAACCTCCCGCAACTAGCTTTCCTCCACATCTCAACACATGCCTTTTTCAAAGCCATACTATTCCTCTGCTCAGGATCAATTATCCACAGC
CTCAATGGAGAGCAAGACATTCGAAAAATGGGAGGCCTACAAAAAACGCTCCCAACAACCACCTCTTGCCTGACCATCGGAAACCTAGCCCTCATGGGAACCCCATTCTTAGCAGGATTTTATTCAAAAGACCTCATCATCGAAAACCTAAACACCTCTTACCTAAATACCTGA
GCACTACTCACAACCCTCCTAGCCACATCATTCACCGCAACCTACAGTCTACGCATAACCATACTAACCCAAGCAGGGACCATCCGCACACCAACAATTACCCCAATAAACGAAAACGACCCAACAATCCTCAATCCAATTACCCGCCTCGCCACAGGCAGTGTCATAGCAGGC
CTACTTATCACATCTTACATCCCCCCCTCAAAAACCCCACCAATAACCATACCCACAATCACAAAAATCGCAGCCATTGCCGTCACAGCCCTAGGCCTAGTTCTGGCCCTCGAACTATCAAGCATGGCACACTCACTAACCCAACCAAAACAAACCCCCCATCTAACCTTCTCC
TCCATACTAGGCTACTTTAACCACCTAGCACACCGCTCAAACACTACAAAACTATTAAACAGCGGACAAAAAATTGCCTCTCACTTAATCGACTTATCCTGATACAAAAAAATTGGCCCAGAAGGACTTGCCAGCCTACAGCTCATAGCAACTAAAACTTCAACCTCCCTCCAC
ACCGGACTAATTAAAACCTACCTAGGATCATTCGCCCTAACCACCCTCACCCTCCTGCTAATATACTAAACCACAAACCACGCACTAATGGCCCCCAACCTCCGAAAATCCCACCCCCTACTAAAAATAGTCAACAACTCCCTAATTGACCTCCCCACCCCCACCAAACATCTCC
GCCTGATGAAACTTCGGGTCCCTACTCGGCATCTGCCTAGTAGTACAAATCCTAACCGGCCTACTACTAGCCATACACTACACAGCAGATACAACATTAGCCTTCTCATCTGTCGCCCACACTTGCCGAAACGTACAACATGGCTGACTAATCCGTAACCTACACGCAAACGGA
GCATCCTTCTTCTTTATTTGTATTTACCTACATATCGGACGTGGATTCTACTATGGTTCGTACTTGTACAAAGAAACTTGAAACACAGGTATCATCCTCCTACTCACCCTCATAGCAACTGCCTTCGTGGGTTATGTCCTCCCATGAGGACAAATATCGTTTTGAGGGGCCACA
GTCATCACCAATCTATTTTCAGCCATCCCCTACATCGGCCAGACCCTCGTAGAATGAGCCTGAGGGGGCTTCTCAGTAGATAATCCCACACTAACTCGATTCTTTGCCCTACACTTCCTCCTCCCCTTCATAATCATAGGCCTCACTCTAATCCACCTCACCTTCCTCCACGAA
TCAGGCTCAAACAACCCCCTAGGTGTTACGTCAGACTGTGACAAAATCCCATTCCACCCATACTTCTCCACAAAAGACACCCTAGGATTCACATTCCTCCTTCCCCTTCTAACAACCCTAGCCCTATTCTCGCCAAACCTATTAGGAGACCCAGAAAACTTCACACCAGCAAAC
CCACTAACTACCCCCCCTCACATCAAGCCAGAATGATATTTCCTATTCGCATATGCCATCCTACGCTCAATCCCCAATAAACTAGGAGGAGTACTAGCCCTTGCCGCCTCAATCCTAATCCTGTTCTTAGTCCCCCTCCTTCACAAATCCAAACAACGATCCCTGACCTTCCGC
CCGCTATCCCAACTACTATTCTGAACCCTAGTCACCAACCTCTTTATCCTCACATGAGTAGGGGGCCAACCTGTAGAACACCCATTTATTATCATTGGTCAGCTAGCATCCCTCACCTACTTCACCACTCTCCTAATCCTCTTCCCCATCGTCGGAAGCCTAGAGAACAAACTA
CTCAACCTTTAAATACTCTAATAGTTTACAAAAACATTGGTCTTGTAAACCAAAGAACGGAGATCATGCCCCTCCTTAGAGTTTTCCTTCTTTTTCCCCTCAGAAAGAGGGGACTTAAACCCCCATCGCCAGCTCCCAAAGCTGGCATTTTTGCGCTAAACTACTCTCTGACC
TCCCCTCACCGCCCGAATCGCCCCCCGCGACAAACCCCGCACAACTTCCAGCACTACAAACAGAGCCAACAGCAACCCCCACCCCGCCACTAAAAACATCCCCACCCCATACGAATAAAGCACAGCCACCCCACTAAAATCTAAACGAACAAAAAATACACCCCCTCCATCGAC
AGTCACCACCCCAAACTTCCAACCCTCAACAAACCCCCCCACAATCACCCCCATAGCAAGCACTAAAACAAACCCTGCACCATACACCACAACCCGTCAATCCCCCCAAGCCTCCGGAAACGGGTCCGCCGCCAACGACACAGAATAAACAAAAACCACCAACATGCCCCCTAA
GTAGATCATAAACAGCACTAACGATACAAAAGAAACCCCCAGACTCAATAATCACCCACACCCCACAGCTGACCCTAATACCAAACCAACCACCCCATAGTACGGAGACGGATTAGAAGCAACTGCCAGCGCCCCAAGAACGAAACAAACCCCTAAAAATGATACAAAATAAGC
CATAGCACATTCTCGCTTGGCTTTTCTCCAAGGTCTGTGGTCTGAAAAACCACCGTTGACTAATCTCAACTACAAGAACACGCAAAATAAGTACATTCTTCTCCCCGCCCCCCCCCCCCCTTCCCCCCATTATGTATTATTGTACATTAACTTATCTACCCCATATACATAC
TATGCATGTACTATATACGCCACAATGTTTAACAACCATTCATCAACATTCCACATACTACATTCAAGGAACAATCATGCATGTACTATAGACATAATTACCTACCACGACAATAACTGCTCTCCCACACTCCACCCAAGGACTAGCTCAAAGCTGAATGGTCCAGGAAATGCT
ACAACAAACCTACTAAAACCATATCCCTGTTAACTTATACTCTACCAGCTTTCCTCGAATACGACAGTGCTTAGCTACAAACCCTGAATGGTAACCAGACATATCCTATAATCTCTATTAAGACGGTCACACCTTGCGACTTTCTCGTTGTATCGGTTTCAGGTATCAGGTTAT
TTATTGATCGAACTTCTCACGTGAAATCAGCAACCCGGTGTTAGTAAGATCCATCATGACTAGCTTCAGGCCCACTCTTTCCCCCTACACCCCTAGCACAACTTGCGCTTTTGCGCCTCTGGTTCCTCGGTCAGGGCCATCTCAACGTTTATCTCTATGATCTTGCTCTTCACC
GATACATCTGGTTGGCCATAACTCACCATTTAGTCCGTGATCGCGACATTTTCTCTCTTCTTCTCTATTGGTTCTCTTTTTTTTGGGGGGTCTTCAGGCAGCCCTTCAGAGTGGATAACGGTGAATACAATCTAAGACCTGGGCATCTCATGGTCGGCGGTCGGAACTTGGCC
CTCAGGGATCCACTAATGTCATGGTTTAACGGATATGCGGTCTCACCTTTACACTGATGCACTTTGTCTTCCATTCAGCTTTGGTGTGCCCACCCTCCCAGCTTGATGGTGCTATTTAGTGAATGCTTGCTGGACATACTTATATAAGTTCACCTTTCCTTTGAGTTGTCGCCC
TATTCTGCTGAACAAACTAGTAATTTCTAGCCAACTAGGCAAAACACCAAGCCACAAAACATCACATTACACCATACTGCACCATATACCCACCTTTATGTGTACTTTTCATATCTTCTCATATACCACCAACACTAAAGTTACAGTAACAAATCAACCATTCCACTTCTTTAC
TTCTCCATCCATTATCCCTCACACTCTCCCACAATGTTTTAAATAACATTCATTTTATTTTTATTTTTACTCTTATTTTCATTCTTATCTTCACCCTTATCTCTATCTATTCACCCTTATTCCGCTTAATTTCACTTACTTTTATTTTTATTTTTACTTTATATTTTTTTATT
TTTACTCTTATTTTCATTCTTATCTTCACCCTTATCTCTATCTATTCACCCCTTATTTCCGCTTAATTCACTTACTTTATTTTTATTTGAATTATTTTTTATTTTTACTCTTATTTCATTCTATCTTCACCTTATCTTATCTTTTTATTTTAATTTCACACACTTTTATTTTTA
TTTTTTACTTTTATTTTTTATTTTTACTTTTATTTTTTATTTTTACTCTTATTTTCATTCTTATCTTCACCCTTATCTCTATCTATTCACCCCTTATTTCCGCTTAATTTCACTTACTTTTATTTTTTATTTTTACTTTTATTTTTTATTTTTACTTTTATTTTTTATTTTTTACT
TTTATTTTTTATTTTTACTCTTATTTTCATTCTTACCTTCACCCTTATCTCTATCTATTCACCCCTTATTTCCGCTTAATTTCACTTACTTTTATTTTTTATTTTTACTTTTATTTTTTATTTTTACTCTTATTTTCATTCTTATCTTCACCCTTATCTCTATCTATTCACCCC
TTATTTCCGCTTAATTTCACTTACTTTTATTTTTTATTTTTACTTTTATTTTTTATTTTTACTTTTATTTTTTATTTTTACTTTTATTTTTTATTTTTACTCTTATTTTCATTCTTACCTTCACCCTTATCTCTATCTATTCACCCCTTATTTCCGCTTAATTTCACTTACTTT
TATTTTTTATTTTTACTTTTATTTTTTATTTTTACTCTTATTTTCATTCTTATCTTCACCCTTATCTCTATCTATTCACCCCTTATTTCCGCTTAATTTCACTTACTTTTATTTTTTATTTTTACTTTTATTTTTTATTTTTACTCTTATTTTCATTCTTACCTTCACCCTTAT
CTCTATCTATTCACCCCTTATTTCCGCTTAATTTCACTTACTTTTATTTTTTATTTTTACTTTTATTTTTTATTTTTACTCTTATTTTCATTCTTACCTTCACCCTTATCTCTATCTATTCACCCCTTATTTCCGCTTAATTTCACTTACTTTTATTTTTACTCTTATCTTTAC
TTCTATCTCATCCTCATTCCTACCAACTACTATTCTGAACCCTAGTCACCAACCTCTTTATCCTCACATGAGTAGGGGGCCAACCTGTAGAACACCCATTTATTATCATTGGTCAGCTAACATCCCTCACCTACTTCACCACTCTCCTAATCCTCTTCCCCATCGTCGGAAGCC
TAGAGAACAAACTACTCAACCTTTAAATACTCTAATAGTTTACAAAAACATTGGTCTTGTAAACCAAAGAACGGAGATCATGCCCCTCCTTAGAGTTTTCCTTCTTTTTCCCCTCAGAAAGAGGGGACTTAAACCCCCATCGCCAGCTCCCAAAGCTGGCATTTTTGCGCTAA
ACTACTCTCTGACCTCCCCTCACCGCCCGAATCGCCCCCCGCGACAAACCCCGCACAACTTCCAGCACTACAAACAGAGCCAACAGCAACCCCCACCCCGCCACTAAAAACATCCCCACCCCATACGAATAAAGCACAGCCACCCCACTAAAATCTAAACGAACAAAAAATACA
CCCCCTCCATCGACAGTCACCACCCCAAACTTCCAACCCTCAACAAACCCCCCCACAATCACCCCCATAGCAAGCACTAAAACAAACCCTGCACCATACACCACAACCCGTCAATCCCCCCAAGCCTCCGGAAACGGGTCCGCCGCCAACGACACAGAATAAACAAAAACCACC
AACATGCCCCCTAAGTAGATCATAAACAGCACTAACGATACAAAAGAAACCCCCAGACTCAATAATCACCCACACCCCACAGCTGACCCTAATACCAAACCAACCACCCCATAGTACGGAGACGGATTAGAAGCAACTGCCAGCGCCCCAAGAACGAAACAAACCCCTAAAAAT
GATACAAAATAAGCCATAGCACATTCTCGCTTGGCTTTTCTCCAAGGTCTGTGGTCTGAAAAACCACCGTTGACTAATCTCAACTACAAGAACACGCAAAATAAGTACATTCTTCTCCCCGCCCCCCCCCTTCCCCCCCATTATGTGTTGTTGTACATTAACCTACTTCCCCC
ATATACATATTATGTATGTACTATATATACTACAATGCCTAACAACCATTCATTCATATTCCACATACTACATTCAAGGAACAATCATGCATGTACTATAGACATAATTACCTACCACGACAATAACTGCTCTCCCACACTCCACCCAAGGACTAGCTCAAAGCTGAATGGTCC
AGGAAATGCTACAACAAACCTACTAAAACCATATCCCTGTTAACTTATACTCTACCAGCTTTCCTCGAATACGACAGTGCTTAGCTACAAACCCTGAATGGTAACCAGACATATCCTATAATCTCTATTAAGACGGTCACACCTTGCGACTTTCTCGTTGTATCGGTTTCAGGT
ATCAGGTTATTTATTGATCGAACTTCTCACGTGAAATCAGCAACCCGGTGTTAGTAAGATCCATCATGACTAGCTTCAGGCCCACTCTTTCCCCCTACACCCCTAGCACAACTTGCGCTTTTGCGCCTCTGGTTCCTCGGTCAGGGCCATCTCAACGTTTATCTCTATGATCTT
GCTCTTCACCGATACATCTGGTTGGCCATAACTCACCATTTAGTCCGTGATCGCGACATTTTCTCTCTTCTTCTCTATTGGTTCTCTTTTTTTTGGGGGGTCTTCAGGCAGCCCTTCAGAGTGGATAACGGTGAATACAATCTAAGACCTGGGCATCTCATGGTCGGCGGTCG
GAACTTGGCCCTCAGGGATCCACTAATGTCATGGTTTAACGGATATGCGGTCTCACCTTTACACTGATGCACTTTGTCTTCCATTCAGCTTTGGTGTGCCCACCCTCCCAGCTTGATGGTGCTATTTAGTGAATGCTTGCTGGACATACTTATATAAGTTCACCTTTCCTTTGA
GTTGTCGCCCTATTCTGCTGAACAAACTAGTAATTTCTAGCCAACTAGGCAAAACACCAAGCCACAAAACATCACATTACACCATACTGCACCATATACCCACCTTTATGTGTACTTTTCATATCTTCTCATATACCACCAACACTAAAGTTACAGTAACAAATCAACCATTC
CACTTCTTTACTTCTCCATCCATTATCCCTCACACTCTCCCACAATTTTTAAATAACATTCATTTTATTTTTATTTTTACTCTTATTTTCATTCTTATCTTCACCCTTATCTCTATCTATTCACCCCTTATTTCCGCTTAATTTCACTTACTTTTATTTTTTATTTTTACTTT
TATTTTTTATTTTTACTCTTATTTTCATTCTTATCTTCACCCTTATCTCTATCTATTCACCCCTTATTTCCGCTTAATTTCACTTACTTTTATTTTTTATTTTTACTTTTATTTTTTATTTTTACTCTTATTTTCATTCTTATCTTCACCCTTATCTCTATCTATTCACCCCTT
ATTTCCGCTTAATTTCACTTACTTTTATTTTTTATTTTTACTTTTATTTTTTATTTTTACTTTTATTTTTTATTTTTACTCTTATTTTCATTCTTATCTTCACCCTTATCTCTATCTATTCACCCCTTATTTCCGCTTAATTTCACTTACTTTTATTTTTTATTTTTACTTTA
TTTTTTATTTTTACTCTTATTTTCATTCTTATCTTCACCCTTATCTCTATCTATTCACCCCTTATTTCCGCTTAATTTCACTTACTTTTATTTTTTATTTTTACTTTTATTTTTTATTTTTACTTTTATTTTTTATTTTTACTCTTATTTTCATTCTTATCTTCACCCTTATCT
CTATCTATTCACCCCTTATTTCCGCTTAATTTCACTTACTTTTATTTTTTATTTTTACTTTTATTTTTTATTTTTACTCTTATTTTCATTCTTATCTTCACCCTTATCTCTATCTATTCACCCCTTATTTCCGCTTAATTTCACTTACTTTTATTTTTTATTTTTACTTTTATTT
TTTATTTTTACTCTTATTTTCATTCTTATCTTCACCCTTATCTCTATCTATTCACCCCTTATTTCCGCTTAATTTCACTTACTTTTATTTTTTATTTTTACTTTTATTTTTTATTTTTACTCTTATTTTCATTCTTACCTTCACCCTTATCTCTATCTATTCACCCCTTATTTC
CGCTTAATTTCACTTACTTTTATTTTTTATTTTTACTTTTATTTTTTATTTTTACTCTTATTTTCATTCTTACCTTCACCCTTATCTCTATCTATTCACCCCTTATTTCCGCTTAATTTCACTTACTTTTACTTTTACTCTCATCCTCATTTTTCACCCACCATTATCCCAAAC
TCCCACTTCCCCACAATTTAACAAACCAATTTAACAAACCAATTTAACAAACCAATTTAACAAACCAATTTAACAAACCAATTTAACAAACCAATTTAACAAACCAATTTAACAAACCAATTAACAAACCAATTAACAAACCAATTTAACAAACCAATTTAACAAACCAATTTA
ACAAACCAATTTAACAAACCAATTTAACAAACCAATTTAACAAACCAATTTAACAAACCAATTTAACAAACCAATTTAACAACCAATTTAACAAACCAATTTAACAAACCAATTTAACAAACCAATTTAACAAACCAATTTAACAAACCAATTTAACAAACCAATTTAACAAAC
CAATTTAACAAACCAATTTAACAAACCAATTTAACAAACCAATTTAACAAACCAATTTAACAAACCAATTTAACAAACCAATTTAACAAG**GTCCCCATAGCTTACAACAAAGCATAACACTGAAGATGTTAAGACGCTGCTAAATGCATCTGAGGAC**AAGAGACTTAGTCCTAACCTTGCCATTAAT
TCCCCGCTAAACATATACATGCAAGTATCCGCACGCCAGTGTAAATGCCCACAGCCGCCTTACCAAGGCAAAAGGAGCAGGCATCAGGCGCACTTACGACTGTAGCCCAAGACGCCTTGCTTAGCCACACCCCCACGGGAACTCAGCAGTAATTAACATTAAGCAATGAGTGTA
AACTTGACTTAGTTATAGCAACCCAAGGGCCGGTAAATCTTGTGCCAGCCACCGCGGTCACACAAGAGGCCCAAATTAATCGCTAAACGGCGTAAAGCGTGGCACTACCCTATCACAGCAACTAAGATCAAAATGCAATCGAGCTGTCATAAGCCTAAGCTGCACCAAAGACCG
CCTTAAAGATGATCTCAGCCCCTCTGATTGACTACGCCCACGAAAGCCAGGGCACAAACTGGGATTAGATACCCCACTATGCCTGGCCTTAAATCTAGATGCTCACCGTACCCAAGCATCCGCCCGAGAACTACGAGCACAAACGCTTGAAACTCTAAGGACTTGGCGGTGCCC
CAAACCCACCTAGAGGAGCCTGTCCTATAATCGATAACCCACGATACACCCGCCCGCTTCTTGCCAAAGCAGCCTATATACCGCCGTCACCAGTCCACCTCTGTGAGAGAATAATAGTGGGCACAATAGCCCCCGCGCTAACAAGACAGGTCAAGGTATAGCCTATGAAGCGGA
AGAGATGGGCTACATTCTCTAAAATAGAGAACTCACGAAAGCGGGTGTGAAATCACCCCCGGAAGGCGGATTTAGCAGTAAAGTGGAATAATAAAGCCCCCTTTAAGTTGGCCCTGGGGCACGTACATACCGCCCGTCACCCTCCTCATAAGCTTCAACCGCCCATAGCTAATA
CGCCCACTAGCCAAAGACGAGGTAAGTCGTAACAAGGTAAGTGTACCGGAAGGTGCACTTAGCACA

Catharus ustulatus (Passeriformes/Turdidae) mitogenome from ND5 to 12S rRNA (CM020378.1 - annotated)

GO-I in Figure 8

ATGGACCTATCATTAGTCCTAAACACATTTATACTACTTACCCTAGCAACCTTATCCACCCCTATCATCTTCCCTTTACTCTCAGACAACCTCAAAAACACCCCCACCATCATCACAAACACAGTTAAGACCGCCTTCATAACCAGCCTAGTCCCCATAACAATCCACCTACAC
TCAGGGACAGAAAGCCTAACATCCCTCTGAGAATGAAAATTCATCATAAACTTTAAAATCCCAATTAGCCTGAAGCTAGACCTATACTCCCTTACATTCTTCCCAATCGCCCTATTCGTCTCTTGATCCATCTTACAGTTCGCAACATGATACATAGCATCAGACCCTCACATC
ACAAAATTCTTCACCTACCTCCTACTCTTCCTAATCGCAATACTCATTCTAATCGTCGCTAATAACCTATTTGTCCTATTCATCGGCTGAGAAGGAGTAGGAATCATATCCTTCCTACTAATCAGCTGATGACACGGACGAGCAGAAGCCAACACCGCCGCCCTCCAAGCCGTG
CTGTTCAACCGAGTCGGGGACATTGGCCTTATCCTCTGCATAGCATGACTTGCATCCACCATAAACACCTGAGAAATCCAACAAGCCACCATACCCCACCAAACCCCAACCCTACCACTCCTAGGCCTCATCCTAGCCGCAACAGGCAAATCCGCCCAATTCGGCCTACACCCA
TGACTCCCAGCAGCCATAGAAGGCCCAACCCCCGTATCCGCCCTACTCCACTCCAGCACAATAGTGGTCGCCGGAATTTTCCTACTCATCCGAACCCACCCACTACTCAGCAACAACCAAACTGCCCTGACCCTATGCCTATGCCTCGGCGCCCTATCTACACTATTTGCTGCC
ACATGTGCCCTCACACAAAATGACATTAAAAAAATCATTGCCTTCTCCACTTCAAGTCAACTAGGCCTCATAATAGTTACAATCGGACTAAACCTCCCCCAACTAGCCTTCCTCCACATCTCAACCCACGCATTCTTCAAAGCTATACTATTCCTATGTTCCGGATCCATCATC
CACAGCCTAAATGGCGAACAAGACATTCGAAAAATGGGAGGCCTCCAAAAACTACTACCCACAACCACCTCATGTTTAACTATCGGCAACCTGGCCCTAATAGGAACACCTTTCCTAGCCGGCTTCTACTCAAAAGACCAAATCATTGAATGCCTCAACACATCCTACCTAAAC
TCCTGAGCCCTACTACTAACCCTCCTAGCTACATCCTTCACCGCAGTATACACAATCCGCATGACCATACTGGTACAAGCTGGCTTTGTACGAATCCCCCCTCTAGCCCCAATCAACGAAAACAACCCAGCAGTAATCTCCCCCATCACCCGCCTAGCACTAGGAAGCATCACA
GCCGGGTTCATCATCACCTCCTTTATCCTTCCAACAAAAACCCCACCCATAACCATACCCTTACACATCAAAATCACAGCCCTAATTGTCACAGCCCTAGGCATCATCCTAGCCCTAGAAATCTCAAAAATAACCCAAACCCTAATCCTTACAAAACAAGGCCCCTTCTCAAAC
TTCTCCACATCACTAGGATACTTTAACCCCCTAATCCACCGATTCAACGTAACAACCATCCTAGCCGGGAGCCAAAACATTGCCTCCCACCTAATCGACCTATCCTGATACAAAATATTAGGCCCAGAGGGCTTAGCCTCCCTACAAGCAACAGCAGCCAAAACTGCCACCACC

TTCCATTCCGGCCTAATCAAAGCCTACCTAGGGTCCTTTGCACTCTCCATCCTGATCATCCTCATATCCACATACAGAACCCCCAAATGGCCCTCAATCTTCGTAAAAACCACCCACTACTCAAAACCATCAACGATGCCCTAATCGACCTCCCCACACCATCAAACATTTCTA
CTTGATGAAATTTTGGATCTCTATTGGGCATCTGCCTTGTCACACAAATCGTCACCGGCCTGCTTCTAGCCACACACTACACAGCAGATACTTCCCTAGCCTTCAACTCAGTCGCCCACATGTGTCGAAATGTCCAATTCGGCTGATTAATCCGAAACCTACACGCAAACGGAG
CCTCATTCTTCTTCATCTGCATCTACCTCCACATCGGCCGAGGATTCTACTACGGCTCGTACCTAAACAAAGAAACCTGAAACATCGGAGTTATCCTCCTCCTAGCCCTTATAGCAACCGCCTTCGTAGGCTACGTACTTCCCTGAGGACAAATATCATTCTGAGGAGCTACAG
TAATTACCAACCTATTCTCAGCAATCCCCTATATTGGCCAAACACTAGTTGAATGAGCTTGAGGCGGGTTCTCAGTTGACAATCCCACGCTAACACGATTCTTCGCCCTCCACTTCCTCCTCCCATTCGTCATCGCAGGCCTAACACTAGTTCACCTCACCTTCCTACACGAAA
CAGGATCAAACAACCCATTAGGAATCCCTGCAGACTGCGATAAAATTCCCTTCCACCCATACTACTCCACAAAAGACATCCTAGGATTCGCACTAATACTTATCCTCCTCGCCTCCATAGCCCTATTCTCCCCTAACATACTAGGAGACCCAGAAAACTTCACCCCGGCCAATC
CCCTAGCTACACCCCCACACATCAAACCCGAATGATACTTCCTATTCGCATATGCCATCCTACGATCCATCCCAAACAAACTAGGAGGAGTCCTAGCCCTAGCCGCCTCCGTCTTAGTCCTATTTTTAGTCCCATTACTCCACAAATCCAAACAACGCTCAATAACCTTTCGGC
CCCTGTCCCAAATTCTCTTCTGAACCCTAGTTGCAAACCTCCTAATCCTCACTTGAGTAGGAAGCCAACCAGTCGAACATCCATTCATCATCATCGGCCAACTAGCTTCAATCTCCTACTTCACAATCATTCTAGTCCTATTCCCACTCGCAGCTGTACTAGAAAATAAAATGC
TAAAATTATAACTCTAATAGTTTATAAAAACATTGGTCTTGTAAGCCAAAGATTGAAGACTCAACCTCTTCTTAGAGTTTCCCCATAAACACTCAGAAAGAAAGGAATCAAACCTTCCTCACCAACTCCCAAAGCTGGTATTTTCAACTAAACTACTTTCTGACCCCTAAACAG
CCCGAATAGCCCCCCGAGATAACCCCCGCACAAGCTCCAGTACCACGAACAACGTCAACAACAACCCTCACCCACCAATTAAAAACAGCCCCGCCCCCCACGAATAGAACTCAGCCACCCCACTAAAATCCAACCGAACCGAAGACAAACCGCCAGAATCCACTGTACCCCACC
CACCAACAACTCAGGCCCCGCTCCCAAAACAACCCCCACCAGAACAACCAAACACATACCCAAACCATAACCAACAACCCCCCAATCTGCCCACGCCTCAGGATAAGGATCCGCCGCCAGCGAAACCGAATAAACAAACACCACCAACATACCTCCAAGATAAACCATCACCAG
CACCAAAGACACAAAAGAAACCCCCAAACTAACCAGTCAACCGCACCCAGCAACAGAAGCCAAAACCAACCCCACAACCCCATAATAAGGCGACGGGTTAGAAGCAACTGCCAACCCCCCCAAAACAAAGCATAACCCCAAAAATAAGACAAACTGCATCATAAATTCCCGCCC
GGGCTTTCTCCAGGATCTGCGGCCTGAAAAGCCGCTGTTATAGAAATTTAACTACAAGAACTCCTTACACCGCCGCCCCCCCTCGGCCCCCCCAGCATGTTTTCTCATGCTTTACAGGGTATGTACGTTCTGCATTAGGTATTTTTGCCCCATCAGACAGTACTATAATGTAGG
ATACTCCACGCGATACGGGTATGCTCTCCCAATTTAACTCAAACATGAACGCCCATGAGATAATGTTCGTGCCATCACCCTACTAGGGACACCTTTGTTTCAGGTACCATATAGCCCAAATGATCCTACCTCCAGCCCAGGCCGCCGGCGTCGCTTAAGTTCGACACTGCTCCT
TTGTACCGACAATCCATACAATATACGGATAACGTCACAGTACACCTTTGTAACCTCCAGGACAATTGAATTCGCCCACCTCCAAGGGTCCCTTCCCGTCCAACCACTTTCAGGAACTCCCAAGCCAGAGAGCCTGGTTATCTATTGATCGTGTTTCTCACGAGAACCGAGCTA
CCCCGTGTCAGTTATACCTTCGGTTATTGGCTTCAAGGACATAACATCCCCCTACACCCTAGCCCAACTTGCTCTTTTGCGCCACTGGTTCCTATTTCAGGGCCATAACTTGATCCATTCCTTCTCTCTTGCTCTTCACAGATACAAGCGGTCGGGATGAATACTCCTCATTCT
CTCTCGTAATCGCGGCATTCCGACCGTCTCTGCACTTTTTCCTTTCTGGGGGTCTCTTCAATAAACCCTTCAAGTGCGTAGCAGGAGTTATCTTCCTCTTGACATGTCCATCACATGTGCGCCTGACTATCGTCCCCCGGAAACCGCATAACTTGTCATGGTCTCATGGTATAA
CCTGTCGCATACCCGGACACTGATGCACTTTGACCCCATTCACGAGGGGGAGGCTATTTACCTCTTAAGTATGCAGATAATGCAATGGTCACCGGACATATTTAGATTGTTTCCCCTTTCTAGGAATTTCCATCTAAACCACCAAAAATCATCATTTTTTTTATTCGTTTATTT
TTATCATGACATTTTCATACAAAATTAGCTAAATATTTTAATACATCTCCCTACCTCTAACCAAAGCATTCGTTATCATAACATTTACAAACAAACTTCCTCTATTTTCCCCCTATTAAACAAACCAACAAACCCATACCATAACACCCACCCATCAACTAACAATCAACCAAA
CATTTTAATACATTCCCCTACCCCTAACCGATATCATTCACTGTCACAATATTTACAAAAGCTTGCCCCATTCCCCCCCCTCCACTCGGACAAAAACTTAACAGCGAGCAAACCCAAAAGAATAAGCCCCCACAAACACTCAGAAAGAAAGGAATCAAACCTTCCTCACCAACT
CCCAAAGCTGGTATTTTCAACTAAACTACTTTCTGACCCCTAAACAGCCCGAATAGCCCCCCGAGATAACCCCCGCACAAGCTCCAGTACCACGAACAACGTCAACAACAACCCTCACCCACCAATTAAAAACAGCCCCGCCCCCCACGAATAGAACTCAGCCACCCCACTAAA
ATCCAACCGAACCGAAGACAAACCACCAGAATCCACTGTACCCCCACCCACCAACAATTCAGGCCCCGCTCCCAAAACAACCCCCACCAGAACAACCAAACACATGCCCAAACCATAACCAACAACCCCCCAATCTGCCCACGCCTCAGGATAAGGATCCGCCGCCAGCGAAAC
CGAATAAACAAACAGCACCAGCATTCCTCCAAGATAAACCATCACCAACACCAAAGACACAAAAGAAACCCCTAAACTAACCAGTCAACCGCACCCAGCAACAGAAGCCAAAACCAACCCCACAACCCCATAATAAGGCGACGGATTAGAAGCAACTGCCAACCCCCCCAAAAC
AAAGCATAACCCCAAAAATAAGACAAACTGCATCATAAATTCCCGCCCGGGCTTTCTCCAGGATCTGCGGCCTGAAAAGCCGCTGTTATAGAAATTTAACTACAAGAACTCCTTACACCCCCCCCCAGTACGTCCCCCCCCCCAGCATGTTTTCTCATGCTTTACAGGGTATGT
ACGTTCTGCATTAGGTATTTTTGCCCCATCAGACAGTACTATAATGTAGGATACTCCACGCGATACGGGTATGCTCTCCCAATTTAACTCAAACATGAACGCCCATGAGATAATGTTCGTGCCATCACCCTACTAGGGACACCTTTGTTTCAGGTACCATATAGCCCAAATGAT
CCTACCTCCAGCCCAGGCCGCCGGCGTCGCTTAAGTTCGACACTGCTCCTTTGTACCGACAATCCATACAATATACGGATAACGTCACAGTACACCTTTGTAACCTCCAGGACAATTGAATTCGCCCACCTCCAAGGGTCCCTTCCCGTCCAACCACTTTCAGGAACTCCCAAG
CCAGAGAGCCTGGTTATCTATTGATCGTGTTTCTCACGAGAACCGAGCTACCCCGTGTCAGTTATACCTTCGGTTATTGGCTTCAAGGACATAACATCCCCCTACACCCTAGCCCAACTTGCTCTTTTGCGCCACTGGTTCCTATTTCAGGGCCATAACTTGATCCATTCCTTC
TCTCTTGCTCTTCACAGATACAAGCGGTCGGGATGAATACTCCTCATTCTCTCTCGTAATCGCGGCATTCCGACCGTCTCTGCACTTTTTCCTTTCTGGGGGTCTCTTCAATAAACCCTTCAAGTGCGTAGCAGGAGTTATCTTCCTCTTGACATGTCCATCACATGTGCGCCT
GACTATCGTCCCCCGGAAACCGCATAACTTGTCATGGTCTCATGGTATAACCTGTCGCATACCCGGACACTGATGCACTTTGACCCCATTCACGAGGGGGAGGCTATTTACCTCTTAAGTATGCAGATAATGCAATGGTCACCGGACATATTTAGATTGTTTCCCCTTTCTAGG
AATTTCCATCTAAACCACCAAAAATCATCATTTTTTTTATTCGTTTATTTTTATCATGACATTTTCATACAAAATTAGCTAAATATTTTAATACATCTCCCTACCTCTAACCAAAGCATTCGTTATCATAACATTTACAAACAAACTTCCTCTATTTTCCCCCTATTAAACAAA
CTAAAAATACAACAAAATCTTTACCTCCACACACACGTACACAAACAGTAATTCCCCTGACAAATCACCCCAACTAAAACCAAACAAAAACACAACACAT**GTCCCTGTAGCTTAATGAAAGCATGACACTGAAGATGTCAAGATGGCTGCCACACACACCCAAGGACA**AGAGAC
TTAGTCCCAACCTTACTGTTAGTTGTTGCTAGGATTATACATGCAAGTATCCGCGCCCCAGTGAGGACGCCCTGGACACCTTACCCAAGGTAGATAGGAGCAGACATCAGGCACACCACCAACCGTAGCCCAAGACGTCTGGCAATGCCACACCCCCACGGGTTATCAGCAGTA
GTTAACATTAAGCAATGAGTGCAAACTTGACTTAGCCATAGCAAATCAGAGTCGGTAAATCCTGTGCCAGCCACCGCGGTCATACAGGAGACTCAAATTAACTTTATAACGGCGTAAAGAGTGGTCGCATGCTATCCAAGTAGCTAAGATTAAAAAGCAACTGAGCTGTCATAA
GCCCAAGATGCTCATAAGGCCTCCGTCCTCAAAGAAGATCTTAGAACAACGATCAATTGAATCCACGAAAGCCAGGGCCCAAACTGGGATTAGATACCCCACTATGCCTGGCCCTAAATCTTGATGCTCTACATTACCTGAGCGTCCGCCCGAGAACTACGAGCACAAACGCTT
AAAACTCTAAGGACTTGGCGGTGCCCCAAACCCACCTAGAGGAGCCTGTTCTGTAATCGATAATCCACGATACTACCTGACCATTCCTTGCACGAATCAGCCTACATACCGCCGTCGCCAGCCCACCTTTCCTGACAGCCCAACAGTGAGCGCAATAGCCTAACCCGCTAGTAA
GACAGGTCAAGGTATAGCCTATGGAATGGAAGCAATGGGCTACATTTTCTAAGCTAGAACATACGGATAGGGATATGAAACTGTCCCTGGAAGGCGGATTTAGCAGTAAAGAGGGACAATGGAGCCCTCTTTAAGCCGGCTCTGGAGCACGTACATACCGCCCGTCACCCTCCT
CATAAGCGCCCCACACCCAATACCTAATAAGCCATTCAGCTAAAGATGAGGTAAGTCGTAACAAGGTAAGTGTACCGGAAGGTGCACTTAGGACAC

Colius striatus (Coliiformes/Coliidae) mitogenome from ND5 to 12S rRNA (MN356125.1 - re-annotated)

GO-NF in Figure 8

ATGGAACCAGCCCTCCTCATTAACACCCTCATAATTCTCACACTACTCGTCATCCTAACCCCCATTCTCCTACCTCTCCTATCAAAAACAATAAAAACCACCCCAACCCTAATTACACATACCACTAAAACAGCTTTCATCATTAGCCTAGCCCCTATGTCCCTATTTATATGG
ACAAACACAGAAAGCATCACATCCTACTGAGAATTAAAGTTCATTATAACATTCAAAATCCCCCTCAGCTTTAAATTCGACCAATATTCCATAATATTCTTCCCGATCGCTTTACTCGTCACGTGGTCAATCCTACAATTTGCAACATGATACATACACCTGGAACCCCACATT
ACAAAATTTTTCTCATACCTATCAATATTCCTAATCGCCATGCTAACCCTCACAGCAGCTAACAACATATTCCTCCTATTCATTGGCTGAGAAGGAGTAGGCATCATATCTTTCATGCTGATTGGGTGATGGTATGGCCGAGCAGATGCTAACACAGCTGCCCTCCAAGCCGTA
CTATACAACCGAATTGGCGATATCGGCTTAATTCTAACCATAGCATGACTCGCCCTGACCTCCAACACCTGAGAAATACAAACCTGCCCGACCCACATACAGACACTACCACTCCTAGGACTCATTCTAGCCGCCACGGGAAAATCAGCCCAATTTGGCCTCCACCCGTGACTA
CCTGCAGCCATGGAGGGCCCAACACCTGTCTCCGCCTTACTTCACTCCAGCACCATAGTAGTAGCCGGAATTTTCCTATTAATCCGCACCCATTACATATTAGAAACCAACCAACTTGCTCTCACCCTATGCCTATGCCTAGGAGCTCTCTCCACCCTATTCGCCGCCACCTGT
GCCCTCACACAAAATGACATCAAAAAAATTATCGCCTTCTCCACCTCCAGCCAACTAGGACTTATGATAGTTACCATTGGACTAAACCTCCCACAACTAGCCTTCCTTCATATCTCAACACACGCTTTCTTCAAAGCCATATTATTTTTATGCTCAGGATCAATCATCCACAAC
CTTAATGGAGAACAAGACATTCGAAAAATGGGCAACCTCCAAAAAATATTACCAACTACCACCTCCTGCTTTACCATCGGCAACCTAGCACTCATAGGCACCCCATTCCTAGCTGGATTTTACTCAAAAGATGTAATTATCGAAAACCTTAACACCTCCTACTTAAACACCTGA
GCACTACTACTCACACTCCTAGCCACGGCATTCACCGCAACTTACAGCATACGCATAACCTTAATAGTACAGACAGGGCAAACCCGAACTAACCCTGCAATTATAATAGACGAAAATAACCCTTCGATCACCCAACCCCTCACCCGCCTTACATTAGGCAGCATCATAGCAGGA
CTACTCATCACATCATACATCACTCCGACCAGCATCTCACCAATAACTATACCAACCCTTACCAAAACAGCTGCCATAACCACCACCATAATAGGACTCATCCTAGCCCTAGAACTCTCAACCCTAACACACAATTTCTATCACCCAAAACAAACCCCATATATAAATTTCTCC
TCCTCCCTAGGCTACTTCAACCCACTCACACATCGCCTTAACATCACCAAACTCCTAAACAACAGCCAAAAAATTGCAACCCACCTTGTAGATTTATCCTGATACAAAAAAATAGGCCCAGAAGGCCTCACCGACTTCCAATTAAAACTAACCAAAACATCAACAATCCTACAC
ACCGGACTAATCAAAACTTACTTAACATCATTTGCACTATCAACCCTAATCATCCTATCTACACAATAAATTAATGGCCCCCAACCTACGAAAATCCCACCCCTTACTAAAAATAATTAATAACTCCCTAATCGACCTACCCACACCCTCCAATATCTCTGTCTGATGGAATTT
CGGGTCACTCCTAGGCATCTGCCTCCTAACACAAATTACAACCGGCCTACTATTAGCCGCCCATTACACTGCAGATACTACACTAGCCTTCTCATCCGTAGCCCACACTTGCCGAAACGTTCAATACGGATGACTAATCCGAAACCTACACGCAAACGGAGCATCATTCTTTTT
CATTTGCATCTACTTCCACATCGGACGAGGATTCTACTATGGCTCCTACCTTTACAAAGAGACCTGAAACACAGGTGTCATCCTTCTCCTTACCCTAATAGCAACAGCCTTCGTAGGCTATGTTTTACCATGAGGACAGATATCCTTCTGAGGAGCCACCGTAATCACCAACCT
ATTCTCAGCTGTCCCATACATCGGCCAATCTCTAGTAGAATGAGCCTGAGGGGGGTTCTCAGTGGACAACCCAACCCTAACCCGATTCTTCACCCTCCACTTCCTCCTCCCCTTCGCAATCGCAGGAATTACACTAATCCACCTGACCTTCCTACACGAATCAGGCTCAAACAA
CCCCCTCGGCATCATCTCAAACTGCGACAAAATCCCCTTCCACCCGTACTTCTCCCTAAAAGATATCCTAGGGTTTGCATTCATATTCCTACCACTAACAACCCTAGCCCTATTCTCACCAAACTTGCTAGGAGACCCAGAAAACTTCACTCCAGCAAACCCCCTAGTAACTCC
CCCACATATTAAACCAGAGTGATATTTTCTATTTGCATACGCCATTCTACGCTCCATCCCAAATAAGCTGGGAGGAGTACTAGCCCTAGCCGCCTCAGTCCTAGTACTTTTCTTATGTCCTCTGCTACACAAATCCAAACAACGCACAATAACTTTCCGACCCCTGTCCCAACT
ACTCTTCTGAACCCTCACTGCAAACCTATTTATCCTCACATGAGTAGGGAGCCAACCAGTAGAACACCCATTCATCATCATCGGCCAACTAGCCTCAATCACATACTTCACAATTATTCTAATCCTATTCCCCATCACCCAAACCCTAGAGAACAAGATACTTAAATACTAAAA
CACTCTAATAGTTTATTAAAACATTGGTCTTGTAAGCCAAAGAGTGAAGACTTATGCCCCTTCTTAGAGTT***ATACCTCCCCACCTCAACCAAAGGTCCCTCCCCAACATTTAACAGTTTCGCGGAGAGGGGCCTTTATTGCCCCACCCCCCTTAACCTCAACCAAAGGTCCCTC***
CCCAACATTTAACAGTTTCGCGGAGAGGGGCCTTTATTGCCCCACCCCCCTTAACCTCAACCAAAGGTCCCTCCCCAACATTTAACAGTTTCGCGGAGAGGGNCCTTTATTGCCCCNCCCCCCTTAACCTCAACCAGTGACCCACCACAAAAAATTACCCCCCCCTACCCCCCC
ATCCACTTTTACAACTTATGTACTTCTGTGCATTCACTTTATAACCCCATTTCAATGCACCTCTCTAGGCGTATTTCTCAATGTTTGAACTGATCCCATAGCATGTCAACGGATACGAACTCGACACAAGCCATTTCTACCAATGTACTAAAAGAATCCATGGCACAAGGAATA
CAGACATAGATAACTTCGTGCTAAAACCATAAAACGCTGCTTTATACATCCCTAATTTCAAGCATACGGGAGTGCTTTACCCATCCCGCCAATCGTATCTAGGCAAAACCGCAGCATTTCTCGTAGTGCCGGTTTCTAAAGGACTGGGTTATTTATTAGTCGTGCACCTCACG
TGAAACCATCAACTCGACGTAGAAAATGTCCTACGTTACTAGCGTCAGGCCCATACTTTCCCCCTACACCCTCGCCCGACTTGCGCTTTTGCGCCTCTGGTTCCTATTTCAGGGCCATCTATAGGCGTTATCCCCACAACTTGTCATTCACAGATACATCTGCTGACCTATGTA
TCAACATTTGGCCTCTTTATCTTGTCACTGGTCGGTTCTCTACTTTTGGTTCTCTTTTTTTTGGGGTAACTTCACCGGACCCCCCCGGCGGTGCGAGATGAGCTTACTATTGCGTGACGTGGGCATCGGTGGACTTCGGCCGATTCCTCTCTTTCCTTCGGTTTTACTGAATGA
GACGGTGTCATGTGTCCGGGGAATCATTTTGACACTGATGCACTTTGTCATGCATTTCTCATGTCTATATTACATCCTTCAATTTATGAGGTTATTTAATTAATGCTCGTAGACATAAAAATTTTTATTTTTGCTTCCTCTAACTTTTCTGCTGATTGCCATCCCAGCTAGAGA
AATCTTCCTAGATACGACACTAAAAATTTTTAATTTTTATCATCATTTCAACACATGTTCAAATTTTTTATCACCGCCTAATTTGCCCTAAAAAAAAATTTCCATTTCATCAAAATTTTAAAAATTTTTCATATTATCTTCACGCAAATACCTTAGCAAATTTCCCCTAAAATTT
TTCACTTCTCCACTATTTGATCCTTACCAAAAAATTCACATACAACATAGGCAAAATCACCCCATTACACCCCCCCTCACACCCCACCCCAAAACTTATTCACCAAACACCAAAAAATCACCTACAATCCCCAACAAACTAGTACCTGATCAGAAAAAGAGGAATTAAACCCCT
ACCTCCAACTCCCAAAGCTGGTATTCTCTACTAAACTACTTTCTGCTTTATACCCCCTACACCGCCCGAATCGCCCCCCGAGATAACCCACGAACCATTTCCAATACCACAAACAGAGTTAACAACAGCCCCCACCCAGCCACTAGAAACAACCCTGCCCCTCATGAATAAAAC
ATAGCCACCCCACTAAAATCCAACCGAACAAAAAACTCACCACCACTATCTACAACACCTACCCCAAACTTCCAATCCCCACTAACCCCGCCAATCAAGAACNCAATTACAAGCACCAACACCAACCCCAAACCATACCCCACTACCCGTCAATCCCCCCAAGCCTCAGGAAAA
GGATCAGCCGCCAAGGCCACAGAATATACAAAAACCACCAGCATCCCCCCAAATAAACCATAAACAACACCAAGGAAACAAAAGACAACCCCAAACTCATTAACCACCCACATCCAACAACAGATGCTAACACCAACCCTACCACCCCATAATACGGAGACGGATTAGACGCA
ACAGCCAACCCCGCCAAAACCAAGCACACCCCCAAAAAAATCACAAAATATGTCATAATAATTTCTGCTTGGCCTCTCTCCAAAACCTGCGGCCTGAAAAGCCGCCGTTGTCTAATTCAACTACAGAAACCTTACCCCAACAAACTTTTAGACCTCTATCCTAATCACCCCAAC
CTCACCCACAAAAACCTACAAAACATCTATTTAACTAAATTTCTGCAAACCCACCTAAGAACTATCAAACAGACTAACCCACACCCACAATACTCCCCGATAAAACCCCCATCCCACAAAATCGCCTAACTATACAACTCCTACATGACTTCGCTACTAAACCACCTAACAAC
CCATACCATAAATTTGACTGTTCAAACGACCCTAACACCATACTTACCCTAAACCCCCCAAACAAAAAATTATACCCCCC**GTCTTTGTAGCTTACCACAAAGCATAACACTGAAGATGTTAAGACGATGCACCACCGCACCCAAAGACA**AAAGACTTAGTCCTAACCTTACAG
TTAGTTTTTACCAAGTATATACATGCAAGTATCTGCACTCCAGTGTAAATGCCCTAGTAACTTCACCAAAGACAAAGGAGCTGGTATCAGGCACACCCACCGTAGTAGCCCAAAACACCTTGCTTTGCCACACCCCCACGGGTACTCAGCAGTAATTGACATTAAGCAATAAGT
GTAAACTTGACTTAGCTATGGTAAACAACCAGGGTTGGTAAATCTTGTGCCAGCCACCGCGGTCATACAAGAAACCCAAATTAACTGTATACGGCGTAAAGAGTGGCACAACAATATTAAACTAACTAAGGCTAAAACATAACCAAGCCGTCATAAGCCAAAGTTACCCATAAA
CTCACCCTAAAAACGACCTTAGTATTCATGATAAACCTATTGCCACGAAAGCTATGACACAAACTGGGATTAGATACCCCACTATGCCTAGCCATAAATCCAGATCCTCCACCCACCCGAGTATCCGCCCGAGAACTACGAGCACAAACGCTTAAAACTCTAAGGACTTGGCGG
TACCCCAAACCCACCTAGAGGAGCCTGTTCTGTAATCGATAACCCACGATACACCTAACCACTTCTAGCCAAAACAGCCTATATACCGCCGTCGTCAGTTTGCCTTAATTGAGAGTGCAACAGCAAACACAATAACCACCACGCGCTAAAAAGACAGGTCAAGGTATAGCCCAT
GAAGTGGAAGAAATGGGCTACATTTTCTATAATAGAACATTCACGAAAAGGGGTATGAAATCTACCCCTAGAAGGCGGATTTAGCAGTAAAAAAAGACAATAAAACTTTTTTTAAGACGGCCCTGGGGTACGTACATACCGCCCGTCACCCTCCTCATATACTGCCCACCAATA
ATACTTAATACAACTAGCCAAAGATAAGAAGAGGTAAGTCGTAACAAGGTAAGTGTACTGGAAAGTGCACTTAGCATA

Fregetta grallaria (Procellariiformes/ Oceanitidae) mitogenome from ND5 to 12S rRNA (MN356296.1 - re-annotated)

GO-FD in Figure 8

ATGGAGCCCACACTACTCCTCAACACCTCCATACTCCTAACACTAACAACTATTCTTACCCCAACGCTACTACCCCTACTATCAAACAAATTCCAAAACTCCCCCACCTCCATTACAAAAGCTGTCAAAGCCGCCTTTCTAATCAGCCTAATCCCAATAACACTCTTCATACAC
TCAGGCATAGAAAGCATCATCTCCCACTGAGAGTGAAAATTCACTACAAACTTCAAAATCCCCCTCAGCCTCAAAATAGACCAATACTCTATAATATTCTTCCCCATTGCCCTATTTGTAACATGATCAATTCTTCAATTCGCAACATGGTACATAGCCTCAGAACCCTACATC
ACAAAATTCTTCTCTTACTTGCTAATATTTCTAATCGCCATACTAACACTAATTATCGCCAACAACATATTCCTACTATTTATTGGCTGAGAAGGAGTCGGAATCATGTCATTCCTGCTAATCGGCTGATGACAAGGACGGACAGAAGCCAACACAGCCGCCCTCCAAGCCGTA
CTCTACAATCGAATCGGCGACATCGGCCTAATTCTAAGTATAGCATGACTCGCCTCAACCACAAACACCTGAGAGATCCAACAAACCATCTCTCCCACCCAGACCCAAACACTCCCACTTTTAGGCCTCATCCTCGCAGCCACAGGAAAATCAGCCCAATTCGGCCTCCACCCC
TGACTACCAGCTGCCATAGAAGGCCCAACCCCAGTCTCCGCCTTACTCCACTCCAGCACCATGGTAGTAGCCGGAATCTTCCTACTCATCCGCACTCACCCCATACTCACCAACAACCAGACTGCCCTCACCCTGTGTCTATGCCTAGGGGCCCTATCAACACTATTTGCCGCC
ATCTGCGCCCTCACACAAAACGACATCAAAAAAATCATTGCCTTCTCCACATCTAGTCAACTAGGACTAATGATAGTCACCATCGGACTAAACCTCCCACAACTAACCTTCCTCCATATCTCAACCCACGCCTTCTTTAAAGCCATACTATTCCTCTGCTCAGGGTCCATTATC
CACAGCCTCAATGGAGAACAAGACATTCGAAAAATAGGAAGCCTACAAAAAACCCTTCCAACAACCACCTCATGTTTAACCATCGGCAACCTCGCATTAATAGGAACACCATTCCTAGCAGGATTTTACTCAAAAGACCTCATCATCGAAAACCTAAACACCTCACACCTAAAC
ACCTGAGCACTACTCCTAACTCTCCTAGCCACATCATTCACCGCAACCTACAGCCTTCGCATAACCCTGCTTGTCCAAACAGGATTCACACGCATTCCCACAGTCACTCCAATAAACGAAAACAACCCAACTATCACCAACCCAATCATCCGCCTTGCCTTAGGCAGCATCCTA
GCAGGCCTAATCATTACATCCTACATTCCTCCTACAAAAACCCCCCAATAACCATACCCACACTTACAAAGACCGCAGCCATCATTATCACAATCTTAGGTATCATCCTTGCCCTAGAACTCTCAAACATAACCCATACCCTGACCCAACCTAAACAAAACATTTACCTAAAC
TTCTCCACCACATTAGGATACTTCAATCCCCTAACTCACCGCTTCAGCTCCACAAGCCTACTAAACAACGGACAAAAACTTGCCCTACACCTAATCGACCTGTCCTGATACAAGAAAATTGGTCCCGAAGGACTCGCCGACCTTCAACTCATAGCAACCAAGACCTCAACCACC
CTCCACACCGGATTAATCAAAACATACCTAGGATCCTTCGCCCTATCGATCCTAATCATTCTCTCAACATACAGACCAAATTAATGGCCCCAAACCTCCGAAAATCTCATCCACTACTAAAAATAATCAACAGCTCCCTAATCGATCTACCCACCCCCCCAAACATCTCTGCCT
GATGAAACTTTGGTTCCCTCCTAGGCATCTGCCTAGCAACACAAATCCTCACCGGCCTACTACTAGCCATACACTACACCGCAGACACAACCCTAGCATTCTCATCCGTTGCCCACACATGCCGAAACGTACAGTACGGCTGACTAATCCGAAACCTCCATGCAAACGGAGCCT
CATTTTTCTTCATCTGCATCTACCTACACATCGGACGAGGACTCTACTACGGCTCGTACCTGTACAAAGAAACTTGAAACACAGGAATCCTTCTCCTACTCACCCTCATAGCAACTGCCTTTGTAGGCTATGTCCTGCCATGAGGACAAATATCGTTCTGAGGGGCCACAGTCA
TCACCAACTTATTCTCGGCTATCCCCTACATCGGCCAAACCATCGTAGAATGAGCCTGAGGGGGATTCTCAGTAGATAACCCCACACTAACCCGATTTTTTGCCTTGCACTTCCTCCTCCCCTTTACAATCGCAGGCCTTACAATAATTCACCTTACCTTCCTCCACGAATCAG
GCTCAAACAATCCCCTAGGTATCGTATCAAACTGTGACAAAATCCCATTCCACCCATATTTCTCCCTAAAAGACATCCTAGGATTCATGCTCATACTCCTCCTACTAACAACCCTAGCCCTCTTCTCCCCCAACCTACTAGGAGACCCAGAGAACTTCACCCCAGCAAATCCAC
TGGTTACACCCCCTCACATCAAACCAGAGTGATACTTCCTATTCGCATACGCCATCCTACGCTCAATCCCCAACAAACTAGGCGGAGTACTAGCCTTAACCGCCTCCGTACTCATCCTATTCCTAAGCCCCCTCCTCCACAAATCCAAGCAACGCACAATAGCTTTCCGTCCAC
TCTCCCAACTCCTATTTTGAATCCTAGTCGCCAACCTACTCATCCTAACATGAGTAGGCAGCCAGCCAGTAGAACACCCATTCATCATCATCGGTCAACTAGCCTCCCTCACCTACTTCACCATCCTCCTAATCCTACTCCCCCTCACCGGAGCCCTAGAAAACAAAATACTTA
ACTACTAAAAACACTCTAATAGTTTATTAAAACATTGGTCTTGTAAACCAAAGAATGAAGACTACACCTCTTCTTAGAGTTCCACTAACCATAAACCACAAACACACCTCAGAAAAAAAGGACTTAAACCTTCATCTCCAACTCCCAAAGCTGGCATTTTACATTAAACTATTC
TCTGACCACCCCTAAACAGCCCGAAGAGTCCCACGCGACAACCCTCGTACAAGCTCCAGTACAACAAACAAAGTCAACAATAACCCCCACCCTGCCACCAAAAACATTCCTACACCCCACGAATAAAANATGGCCACCCCACTAAAATCCAACCGAACAGAAAACATACCCCCA
CTATCAACAGTCCCCACATCCACCTTCCAACACTCAATGAATCCCCCAACAACACCCCCAATAACAAGCACAGAAACAAGCCCCACCCCATACCCTACAACATCCCAATCTCCCCAGGCCTCCGGAAAAGGATCGGCCGCCAAAGACACCGAGTACACAAAGACCACTAACATT
CCACCCAAATAGACCATAAACAGCACCAATGATACAAAAGAAATACCCAAACTCAACAATCACCCACACCCCACAACTGACCCTAATACCAAACCAACTACCCCATAATAAGGCGAAGGATTGGACGCAACTGCCAATCCTCCCAAAACAAAGCATAACCCCAAAAAAAGGACA
AAATAAGTCATAGCAGTTTCCGCCTGGCCCCTCCCCAGGACTTGTGGCCTGAAAAACCACCGTTGTAAATTTCAACTACAGAAACCCCCAAGACACTATCAATGGTACTTCGGACATTACTATATGGCTACCCCCCCTACCCCCCCAGGTACTGACATCAGCTATGTATATCGG
AATTACCTGGTCCATCGAGCATTACATGGTATGTGAGAGGTGCATGCAGTAGCATGTGCTAAATCCATGCCATGTAAATAATACATTATATGTATATGTGTCGCATACATTNNATGCATGTATANCNTNCATAACATGTTAACGGATATACCCTACATCACCACCCCTCTACTT
TCAGGAAATGGCAGAGCTTCAATGATCCTTGGACATAACACAAACACTCCTCGCGCTAAACCCATTCACCCTCTAGGTTATACTTGACAAGTCCCTAAGTATACGGCAGTGCTTAAGTACAAACTCTGTCACGGTAGCAGGACATAACCCTTCAATCACTTCTCGTAGGGCCGG
TATCTATCGGACTGGGTTATCTATTAATCGTTCTTCTCACGTGAAATCAGCAACCCGGTGTATGAAAGACTCTGCGTTACCAGCTTCAGGACCATTCATTCCCCCTACACCCCTAGCACTACTTGCACTTTTGCGCCTCTGGTTCCTATGTCAGGGCCATAACTTGGTTAATCC
TACGACCTTGCTCTTTACGAATACATCTGGTTGGCTATATGCGAGCTTCTCACCCGTGATCGCGACATCTGGATAGCCTTTCCTCTTTTGGTTCCCTTTTTTTTTTGGGGCGTCTTCAGGCAGCCCCTCCAGTGCGGAGCAGGTGATTCCAAGTTATAGACGTGAGCATCACATG
ACATGCGGCCTGATTTTGGTCCTCAGGTGTTACTGAATGAGACGGTTGAAGTATATGGGGAATCATCTTAACACTGATGCACTTTGCGTTGCATTTGGTTATGGTGTGTCCACAGACTATTTTATATGTTGCTATTTGATGAATGCTTGTGGGACATGATTTTTCACTTTTACA
CTTCCTCTAACTTTCTAAACAACACTAGGAACTTTCAACTAATTTTTCACTACAATTTTTTTTCATTTTTTTTCATTTTTTCATCACACTTTTTTTCACATACCATATCCGCTAAAAATCCATTAATAAATCAAAACCATTCCATCATCGTTTCATCATCGTTTCATCATCGTTT
CATCACCACCCACACCAAACAAATACTAGGAATATTTCTACTAAACTTTTACCATTTCTTTCCATCGTATTTTTATCACTCATTTTATGTGTGCAAATGCGTGTATATCGACATTAATCAGCGCTGGAGTTACATTAATAAATTTGATTTCCATTTCTTTTTCCCACACCACAT
GCAGCGATATCCCTACTAACCACCACCAACCACTAACAAATCTTTCCTCCACGAATCAGGCTCAAACAATCCCCTAGGTATCGTATCAAACTGTGACAAAATCCCATTCCACCCATATTTCTCCCTAAAAGACATCCTAGGATTCATGCTCATACTCCTCCTACTAACAACCCT
AGCCCTCTTCTCCCCCAACCTACTAGGAGACCCAGAGAACTTCACCCCAGCAAATCCACTGGTTACACCCCCTCACATCAAACCAGAGTGATACTTCCTATTCGCATACGCCATCCTACGCTCAATCCCCAACAAACTAGGCGGAGTACTAGCCTTAACCGCCTCCGTACTCAT
CCTATTCCTAAGCCCCCTCCTCCACAAATCCAAGCAACGCACAATAGCTTTCCGTCCACTCTCCCAACTCCTATTTTGAATCCTAGTCGCCAACCTACTCATCCTAACATGAGTAGGCAGCCAGCCAGTAGAACACCCATTCATCATCATCGGTCAACTAGCCTCCCTCACCTA
CTTCACCATCCTCCTAATCCTACTCCCCCTCACCGGAGCCCTAGAAAACAAAATACTTAACTACTAAAAACACTCTAATAGTTTATTAAAACATTGGTCTTGTAAACCAAAGAATGAAGACTACACCTCTTCTTAGAGTTCCACTAACCATAAACCACAAACACACCTCAGAAA
AAAAGGACTTAAACCTTCATCTCCAACTCCCAAAGCTGGCATTTTACATTAAACTATTCTCTGACCACCCCTAAACAGCCCGAAGAGTCCCACGCGACAACCCTCGTACAAGCTCCAGTACAACAAACAAAGTCAACAATAACCCCCACCCTGCCACCAAAAACATTCCTACAC
CCCACGAATAAAANATGGCCACCCCACTAAAATCCAACCGAACAGAAAACATACCCCCACTATCAACAGTCCCCACATCCACCTTCCAACACTCAATGAATCCCCCAACAACACCCCCAATAACAAGCACAGAAACAAGCCCCACCCCATACCCTACAACATCCCAATCTCCCC
AGGCCTCCGGAAAAGGATCGGCCGCCAAAGACACCGAGTACACAAAGACCACTAACATTCCACCCAAATAGACCATAAACAGCACCAATGATACAAAAGAAATACCCAAACTCAACAATCACCCACACCCCACAACTGACCCTAATACCAAACCAACTACCCCATAATAAGGCG
AAGGATTGGACGCAACTGCCAATCCTCCCAAAACAAAGCATAACCCCAAAAAAAGGACAAAATAAGTCATAGCAGTTTCCGCCTGGCCCCTCCCCAGGACTTGTGGCCTGAAAAACCACCGTTGTAAATTTCAACTACAGAAACCCCCAAGACACTATCAATGGTACTTCGGAC
ATTACTATATGGCTACCCCCCCTACCCCCCCAGGTACTGACATCAGCTATGTATATCGGAATTACCTGGTCCATCGAGCATTACATGGTATGTGAGAGGTGCATGCAGTAGCATGTGCTAAATCCATGCCATGTAAATAATACATTATATGTATATGTGTCGCATACATTNNAT
GCATGTATANCNTNCATAACATGTTAACGGATATACCCTACATCACCACCCCTCTACTTTCAGGAAATGGCAGAGCTTCAATGATCCTTGGACATAACACAAACACTCCTCGCGCTAAACCCATTCACCCTCTAGGTTATACTTGACAAGTCCCTAAGTATACGGCAGTGCTTA
AGTACAAACTCTGTCACGGTAGCAGGACATAACCCTTCAATCACTTCTCGTAGGGCCGGTATCTATCGGACTGGGTTATCTATTAATCGTTCTTCTCACGTGAAATCAGCAACCCGGTGTATGAAAGACTCTGCGTTACCAGCTTCAGGACCATTCATTCCCCCTACACCCCTA
GCACTACTTGCACTTTTGCGCCTCTGGTTCCTATGTCAGGGCCATAACTTGGTTAATCCTACGACCTTGCTCTTTACGAATACATCTGGTTGGCTATATGCGAGCTTCTCACCCGTGATCGCGACATCTGGATAGCCTTTCCTCTTTTGGTTCCCTTTTTTTTTTGGGGCGTCTT
CAGGCAGCCCCTCCAGTGCGGAGCAGGTGATTCCAAGTTATAGACGTGAGCATCACATGACATGCGGCCTGATTTTGGTCCTCAGGTGTTACTGAATGAGACGGTTGAAGTATATGGGGAATCATCTTAACACTGATGCACTTTGCGTTGCATTTGGTTATGGTGTGTCCACAG

[illegible]

Ketupa blackistoni (Strigiformes/Strigidae) mitogenome from ND5 to 12S rRNA (LC099104 - re-annotated)

GO-FD in Figure 8

[illegible]

Ketupa flavipes (Strigiformes/Strigidae) mitogenome from ND5 to 12S rRNA (LC099100.1 - re-annotated)

GO-FD in Figure 8

[illegible]

Leptosomus discolor (Leptosomiformes/Leptosomidae) mitogenome from CYTB to CR2 (MN356135.1 - re-annotated)

GO-NE in Figure 8

[illegible]

Merops nubicus (Coraciiformes/Meropidae) mitogenome from ND5 to 12S rRNA (CM020464.1 - annotated)

GO-II in Figure 8

[illegible]

Melanerpes aurifrons (Piciformes/Picidae) mitogenome from ND5 to 12S rRNA (CM022134.1 - annotated)

GO-IV in Figure 8

[illegible]

Morus serrator (Suliformes/Sulidae) mitogenome from ND5 to 12S rRNA (GU071056.1 - re-annotated)

GO-FD in Figure 8

ATGGAAACTACACTACTCCTGAATACCTCCATACTTCTTACACTTACAATTATCACCACACCAATTCTACTCCCACTTCTATCAAAAAAATTACAAAACTCCCCAGCCACCATCACTCGTACTGTTAAAATTGCCTTCATAATTAGCCTAGTACCCATAATACTCTTCATATAC
TCAGGCATAGAAAGCATTACATCCTGCTGAGAATGAAAATTCATCGCAAACTTCAAAATCCCGCTCAACCTCAAAATCGACCAATACTCCACAATATTCTTCCCTATCGCTCTGTTTGTAACATGATCCATCCTTCAATTTGCAACATGATACATAAACGCAGAGCCACACATT
ACAAAATTCTTTTTGTACCTCCTAACATTCCTAATTGCCATACTCACACTAACCATCGCCAACAATATATTCCTACTATTCATTGGCTGAGAAGGAGTTGGAATCATGTCATTCTTACTAATTGGCTGATGACAAGGACGAGCAGAAGCCAACACAGCAGCCCTCCAAGCTATC
CTATACAACCGAATCGGAGACATTGGCCTTATCTTAAGCATAGCATGACTTGCCTCAACCCTAAACACCTGAGAACTACAACAACCCCTTTCCTACACCCAAACCCCCACAATCCCCCTACTAGGCCTTATCCTCGCTGCAGCAGGAAAATCCGCCCAATTCGGCCTTCACCCC
TGACTTCCCGCTGCTATAGAGGGCCCAACCCCTGTCTCTGCATTACTTCACTCCAGCACTATAGTTGTAGCTGGAATTTTTCTACTCATTCGCACTCACCCCCTCCTTGCCAACAACCCAACTGCCCTCTCCACCTGCCTATGTCTTGGAGCCCTATCCACATTATTCGCTGCT
ACATGCGCACTAACACAAAACGACATCAAAAAAATCATTGCCTTCTCCACATCTAGTCAACTCGGCCTAATAATAGTTACAATCGGACTAAATCTACCACAACTAGCCTTCCTCCACATCTCTACCCACGCATTCTTCAAAGCTATACTGTTTCTATGCTCGGGCTCAATCATC
CACAACCTCAATGGAGAACAAGATATCCGAAAAATAGGGGGCCTACAAAAAACACTCCCAACAACTACATCCTGCTTAACTATCGGCAACATAGCCCTCATAGGAACTCCATTCCTAGCAGGATTCTATTCAAAAGACCTCATTATTGAAAACCTAAACACATCTTACCTTAAT
ACATGAGCACTACTTCTAACACTCCTGGCCACATCATTCACTGCAACCTACAGCCTGCGTATAACCCTTTTAGTCCAAACAGGATTCACCCGCACACCAACAATCACTTCAATAAATGAAAACAACCCAATAATTACCAATCCAATCACCCGCCTAGCCCTAGGTAGCATTACA
GCCGGCCTACTTATCACATCTTATATTACACCAACTAAAACACCTCCAATAACCATGCCTACAAGCATAAAAACTGCTGCCATTATCATTACAGCCTTAGGTATTATCCTAGCCCTAGAACTTTCAAATATAACCCACACCCTAACCCAGCCAAAACAAAACACATACCTAAAC
TTCTCCTCTACGCTAGGATACTTCAACCTCCTAACACACCGTACTAGCTCCACCAACCTCCTACACAGCGGACAAAAAATCGCCTCACATTTAATTGACTCATCCTGATACAAAAAAATAGGACCCGAAGGACTTGCCAACTTACAACAGATAGCAGCCAAAACCTCAACCACC
CTTCATACCGGACTAATCAAAACCTACCTAGGATCCTTCGCCCTATCTATTCTCATTATCCTTATATCGACCCAATAACCCTAACCTAATGGCCCCAAATATTCGAAAATCTCACCCCCTATTAAAAATAGTCAACAACACCCTAATTGACCTCCCCACCCCTCCAAACATCTC
TGCTTGATGAAACTTTGGCTCACTCTTAGGTATCTGCCTTATAACACAAATCGTAACCGGACTTCTCCTAGCCACCCACTACACAGCAGACACAACCCTAGCATTCTCATCAGTCGCCCACACCTGCCGAAATGTTCAATACGGCTGATTAATCCGTAACCTCCATGCAAACGG
AGCATCCTTCTTCTTCATCTGTATCTACCTACACATCGGACGAGGACTCTACTATGGCTCCTACTTGTACAAAGAAACCTGAAACACTGGAGTTATCCTCCTACTTACTCTCATAGCAACAGCCTTCGTAGGATACGTCCTTCCATGAGGACAGATATCATTCTGAGGAGCCAC
AGTCATTACCAACCTATTCTCAGCCATCCCATACATTGGCCAAACCCTTGTAGAATGGGCCTGAGGCGGATTCTCTGTAGACAATCCAACATTAACCCGATTCTTTGCCTTACACTTCCTACTCCCATTCATCATTGCAGGGCTTGCCCTAATCCATCTCACCTTCCTCCACGA
ATCCGGCTCAAACAATCCACTAGGTATCTCATCAGACTGTGACAAAATCCCATTTCACCCCTACTTCTCCATTAAAGATATGCTAGGATTCATCCTACTACTTCCCTTACTAACAACCCTAGCCCTATTCTCCCCCAACCTACTCGGAGACCCAGAAAACTTCACCCCAGCAAA
TCCACTAGTTACCCCTCCCCATATCAAGCCAGAATGATACTTCTTATTTGCATACGCCATTCTACGCTCAATCCCCAACAAACTAGGAGGAGTATTAGCCCTAGCTGCCTCCGTATTAATTCTCTTCCTCAGCCCCCTCCTCCACAAATCCAAACAACGCACAATAACTTTTCG
ACCACTTTCCCAACTCCTATTCTGAACTCTAGTTGCTAACCTCCTTGTCCTAACCTGAGTCGGCAGCCAACCCGTAGAACATCCATTCATTATCATCGGCCAATTAGCCTCCATCGCCTACTTCACTATCCTCCTCATTCTCTTCCCACTCATTGGAGCCCTAGAAAACAAAAT
ACTAAACTACTAAAGTACTCTAATAGTTTATAAAAACATTGGTCTTGTAAACCAAAGAATGAAGATTACACCCCTTCTTAGAGTTTCAACATCTACCATCAGAGAAAAAGGACTTAAACCTCTACCGCCAGCTCCCAAAGCTGATATTCTACATTAAACTATCCTCTGATCTCC
CCCCTAAACCGCCCGAATCGCCCCCCGCGATAACCCACGTACAAGCTCCAACACAACAAACAAAGTTAACAGCAACCCTCACCCAGCCACTACAAACAACCCCGCCCCATTCGAATAAAATATTGCTACACCCCCAAAATCCAACCGAACCAAAAACATCCCCCCATTATCCAC
AGTTACTACACCCAACTTCCATCCCTCTATCAACCCTCCAACAGTAACCCCAACCAAGAGCACCAAAATAAATCCTATACCATACCCCACAACCCGTCAATCATCCCAAGCTTCAGGAAACGGATCAGCAGCTAAAGCCACAGAATATACAAAAACAACCAACATACCCCCTAA
ATATACCATAAACAGCACCAATGCCACAAAAGACACCCCTAAACTTAACAACCACCCACATCCCACAACAGATGCCACTACCAAACCAATTACCCCATAATAAGGAGACGGATTAGAAGCGACTGCCAACGCACCCAAAATAAAACCCACCCCCAAAAAAACTACAAAATAAGT
CATCACAGTTCCTGCTTGGCCTCTCTCCAAGCTATGCGACCTGAAAAGCCGCCGTTGTAAACCTCAACCACAGGAACCCAAAGAGAATTTCTCTTTTTTCCCCCCCCCTCCCCCCCCGCATACAATCTACTACTTTTCTAAGTACTTTTTTATTTTTATGTCCCCCTATGCATT
CATTCATAATCCACACCCTACATTAACTTTTTCCTAGTTTGATAACTCAATTTATGGATTAAACACATAGAAATACCATCGGATAATACCAAACTTTACCCTCTAACTAACCAAACCATTATAAATGAAATGTCCTGCCAACGGCCACTCCTTTGACCTCGAACCCAAACCAT
CCTAGAATAAATAATCTGTACCTTAATATCTTTAATGATACGGCAGTGCTGCAGCATACTAACCCCTGAATGGTTACTCGACATACCCTCTCAACAATTCTCGAAGTACCGGTTTCTGAAGTATTAGGTTATCTATTGATCGAGCTTCTCACGTGAAATCAGCAACGCGCCGCA
TAGAAGATCCTACGTTACTAGCTTCAGGACCATTCTTTCCCCATCACCCCTAGCACTTCTTGCACTTTTGCGCCTCTGGTTCCTCGGTCAGGGCCATAACTTGATTAACTCCTATGTTTCTCACCTTTCACAGAGTCATCTGGTTGGCTATTGATCATCATTGTCTCTCTTAA
TCGCGTCACCTAAACTTTTCCTCTTTTGGTTCTCTTTTTTTTTGGGCGTCTTCACAGGTGACTTCTCCCAGTGCAGAGCGGAGAACTTACAATCTAGGTCTGAACATACACTGGTATGCGTCCGGGCCTCGCCGTCAAGAGTTGATTAATGAGACGGTTGACGTATATGGGGAA
TCATTTTTACACTGATGCACTTTGTTTTCCATTTGGTTATGGATTATTTACCCACTGTTTACTATGTTGTTATTTATTGAATGCTTCTAGGACATGATTTTTCACTTTTTCACTTCCTCTAATTTTTTAACCAACACTAGTAGACTTTCAAGTAATTTTTATCATTCACTTACG
ATAAATTTTTTTCAATTTATTTGTATTTTTATCATTCTTTTTTACATGTTATTAGTACTGGAGTTCCATTAATAAATTAATCAATCTTTCTTATCATACTTTTCCACTTTACCATACCTTTTCACTTTAGTGTCACCCTTACTATTTCCTCCACGAATCCGGCTCAAACAAT
CCACTAGGTATCTCATCAGACTGTGACAAAATCCCATTTCACCCCTACTTCTCCATTAAAGATATGCTAGGATTCATCCTACTACTTCCCTTACTAACAACCCTAGCCCTATTCTCCCCCAACCTACTCGGAGACCCAGAAAACTTCACCCCAGCAAATCCACTAGTTACCCCT
CCCCATATCAAGCCAGAATGATACTTCTTATTTGCATACGCCATTCTACGCTCAATCCCCAACAAACTAGGAGGAGTATTAGCCCTAGCTGCCTCCGTATTAATTCTCTTCCTCAGCCCCCTCCTCCACAAATCCAAACAACGCACAATAACTTTTCGACCACTTTCCCAACTC
CTATTCTGAACTCTAGTTGCTAACCTCCTTGTCCTAACCTGAGTCGGCAGCCAACCCGTAGAACATCCATTCATTATCATCGGCCAATTAGCCTCCATCGCCTACTTCACTATCCTCCTCATTCTCTTCCCACTCATTGGAGCCCTAGAAAACAAAATACTAAACTACTAAAGT
ACTCTAATAGTTTATAAAAACATTGGTCTTGTAAACCAAAGAATGAAGATTACACCCCTTCTTAGAGTTTCAACATCTACCATCAGAGAAAAAGGACTTAAACCTCTACCGCCAGCTCCCAAAGCTGATATTCTACATTAAACTATCCTCTGATCTCCCCCCTAAACCGCCCGA
ATCGCCCCCCGCGATAACCCACGTACAAGCTCCAACACAACAAACAAAGTTAACAGCAACCCTCACCCAGCCACTACAAACAACCCCGCCCCATTCGAATAAAATATTGCTACACCCCCAAAATCCAACCGAACCAAAAACATCCCCCCATTATCCACAGTTACTACACCCAAC
TTCCATCCCTCTATCAACCCTCCAACAGTAACCCCAACCAAGAGCACCAAAATAAATCCTATACCATACCCCACAACCCGTCAATCATCCCAAGCTTCAGGAAACGGATCAGCAGCTAAAGCCACAGAATATACAAAAACAACCAACATACCCCCTAAATATACCATAAACAGC
ACCAATGCCACAAAAGACACCCCTAAACTTAACAACCACCCACATCCCACAACAGATGCCACTACCAAACCAATTACCCCATAATAAGGAGACGGATTAGAAGCGACTGCCAACGCACCCAAAATAAAACCCACCCCCAAAAAAACTACAAAATAAGTCATCACAGTTCCTGCT
TGGCCTCTCTCCAAGCTATGCGACCTGAAAAGCCGCCGTTGTAAACCTCAACCACAGGAACCCAAAGAGAATTTCTCTTTTTTCCCCCCCCCTCCCCCCCCGCATACAATCTACTACTTTTCTAAGTACTTTTTTATTTTTATGTCCCTTGGGCATTCAACTATAACCCATAT
CATACATTAACTTTTTTCCTAGTTTGATAACTCAATTTATGGATTAAACACATAGAAATACCATCGGATAATACCAAACTTTACCCTCTAACTAACCAAACCATTATAAATGAAATGTTCTACCAACAGTCACTCCTTTGACCTCGAACCCAAACCATCCTAGAATAAATAACC
TGTACCTTAATATCTTTAATGATACGGCAGTGCTGCAGCATACTAACCCCTAAATGGTTACTCGACATACCCTCTCAACAATTCTCGAAGTACCGGTTTCTGAAGTATTAGGTTATCTATTGATCGAGCTTCTCACGTGAAATCAGCAACGCGCCGCATAGAAGATCCTACGTT
ACTAGCTTCAGGACCATTCTTTCCCCCTACACCCCTAGCACTTCTTGCACTTTTGCGCCTCTGGTTCCTCGGTCAGGGCCATAACTTGATTAACTCCTATGTTTCTCACCTTTCACAGAGTCATCTGGTTGGCTATTGATCATCATTGTCTCTCTTAATCGCGTCACCTAAACT
TCTCCTCTTTTGGTTCTCTTTTTTTTTGGGCGTCTTCACAGGTGACTTCTCCCAGTGCAGAGCGGAGAACTTACAATCTAGGTCTGAACATACACTGGTATGCGTCCGGGCCTCGCCGTCAAGAGTTGATTAATGAGACGGTTGACGTATATGGGGAATCATTTTTACACTGAT
GCACTTTGTTTTCCATTTGGTTATGGATTATTTACCCACTGTTTACTATGTTGTTATTTATTGAATGCTTCTAGGACATGATTTTTCACTTTTTCACTTCCTCTAATTTTTTAACCAACACTAGTAGACTTTCAAGTAATTTTTATCATTCACTTACGATAAATTTTTTTTCAA
TTTTATTTGTATTTTTATCATTCTTTTTTACATGTTATTAGTACTGGAGTTCCATTAATAAATTAACACCACTCGAACGAACAAACTCTTACATATTTTCCATATATATTCACACAATCATAACCCAAAATTACTAAAGAAACCCCCCTACAAACAAACAAACAAACAAACAAA
CAAACAAACAAACAAACAAACAAACAAACAAACAAACAAACAAACAAACAAACAAACAAACAAACAAACAAACAAACAAACAAACAAACAAACAAACAAACAAACAAACAAACAAACAAACAAACAAACAAACAAACCA**GTCCCTGTAGCTTACAACAAAGCACGGCACTGAAGATGC**
CAAGACGGCTGCTACATGCACCCAGAGACAAAAGACTTAGTCCTAACCTTACCGTTAATTCTTGCTAAACATATACATGCAAGTATCTGCGCCCCAGTGTAAATGCCCTCAGCGCCCTAATTAGGGTAAGAGGAGCAGGTATCAGGCACACCCACGACAGTAGCCCAAGACGCC
TTGCTAGGCCACACCCCCACGGGTACTCAGCAGTAATTAACATTAAGCAATAAGTGTAAACTTGACTTAGTTATAGCAACATTCAGGGCTGGTAAATCTTGTGCCAGCCACCGCGGTCACACAAGAAGCCCAAATTAACCCGGATATCGGCGTAAAGAGTGGTATTGTATTATC
CTTCTAACTAGAGTTGAAATACAACTGAGCTGTCATAAGCCTAAGATGTACTTAAAATTCCCCCTAAAAACGACCCTAGTACTTATGATCAATTAAACACCACTAAAGCTAGGGCACAAACTGGGATTAGATACCCCACTATGCCTAGCCTTAAATCTTGGCACTTACCATACT
AAAGTACCCGCCTGAGAACTACGAGCACAAACGCTTAAAACTCTAAGGACTTGGCGGTGCTCTAAACCCACCTAGAGGAGCCTGTTCTATAATCGATAATCCACGATGCACCCGACCACCCCTTGCCAGTACAGCCTACATACCGCCGTCGTCAGCTCACCTCCCTTGAGAGTG
TAGTAGTGAGCACAATAGCCATAAATTCCGCTAGTAAGACAGGTCAAGGTATAGCCCATGGGGTGGGAGAAATGGGCTACATTTTCTAAGATAGAAAAGTTCACGAAAGGGGGTGTGCAAGCATCCCTAGAAGGCGGATTTAGTAGTAAAGCGGGACCATAGAGCCCCCTTTAA
ACCGGCCCTGGAGCACGTACATACCGCCCGTCACCCTCATCACGAGCTATAAACTCTTATTACTAATACTCCATACCAGCTAAAGATGAGGTAAGTCGTAACAAGGTAAGTGTACCGGAAGGTGCACTTAGCATAT

Nyctibius grandis (Caprimulgiformes/Nyctibiidae) mitogenome from ND5 to 12S rRNA (CM023771.1 - annotated)

GO-NA in Figure 8

ATGGAGACCACACTGCTTATCACCTCCATACTCCTCACACTAACAGTCATCCTCTCTCCAATACTCCTTCCACTGTTATCAGAAACATTCAAAAACTCCCCAACTACCATCACCCATGCTATCAAAACCGCATTCTTCTGCAGCCTAGTACCAATAACACTATTCATTCACTCG
AACACAAATGTCATCGTCTCTTACTGAGAATGAAAACTTGCTCCAAATTTCAAAATCCCACTCAGCTTTAAAATCGACCAATACTCCATAATATTCTTCCCTATCGCCCTGTTCGTAACATGATCCATCCTCCAATTTGCAACGTGATACATAGCCACCGAACCCTATACCACA
AAATTCTTCTCTTACCTCCTGATATTCTTAATTGCCATACTAACATTAATCATTGCTAATAATATGTTCCTACTATTCATCGGCTGAGAAGGAGTTGGAATCATGTCATTCCTACTAATTGGTTGATGACAAGGACGGGCAGAAGCCAACACCGCTGCCCTTCAAGCAGTACTC
TATAACCGAATTGGCGACATCGGCCTCATCCTAAGCATAGCATGACTTGCCTTAACCTTGAACACCTGAGAACTGCAACAAGCCTCCTCCCCCACACAAATTCATACACTCCCCCTTCTAGGTCTTATTCTAGCAGCCATAGGAAAATCCGCACAATTTGGCCTCCACCCCTGA
CTACCAGCCGCCATGGAAGGTCCAACTCCCGTCTCTGCCCTACTTCACTCAAGCACCATAGTAGTAGCCGGAATTTTCCTACTAATCCGCACTCACCATATACTCGCCAGCAACCAGACCGCCCTCACCTTGTGCTTATGCCTAGGGGCCTTATCCACACTGTTCGCTGCCACC
TGTGCTCTCACACAAAATGACATCAAGAAAATCATTGCCTTCTCTACATCCAGCCAACTAGGCCTAATAATAGTTACCATTGGACTAAACCTACCACAACTAACATTCCTACACATCTCAACACACGCCTTCTTTAAAGCTATGCTATTTCTCTGTTCAGGGTCAATCATCCAC
AACCTAAGTGGCGAACAAGACATTCGAAAAATAGGGAACCTCCAAAAAATACTTCCAACAACCACCTCTTGCCTAACCATTGGCAACCTCGCCCTAATAGGAACACCATTCCTAGCAGGATTTTACTCAAAAGACCTCATCATCGAACATCTGAACACCTCCTACCTAAACACC
TGAGCACTACTCCTGACACTCCTAGCCACATCCTTCACCGCAACCTATACATTACGAATAACTCTACTAGTCCAAACAGGGTTCACCCGTACACCTGCAACAACCTCAACAAATGAAAGTAACCCAACAATCACCAACCCCATTATCCGCCTAGCCTTAGGCAGCATTCTAGCC
GGTCTACTCATCACATCATACATCACTCCCTCAAAAACACCCCCAATAACCATACCAACCATCACAAAAACTGCCGCCATTATCATCACAATCCTAGGTATCATTATGGCTCTAGAACTCTCAACCCTAACCCACACAATAACCTCGCCAAAACAAAACATCTACCTAAACTTC
TCATCTACCTTAGGATATTTCAACCCCTTAACCCACCGCTTCTTCCCTACCAAATTACTAAGCAATGGACAAAAAATCGCCTCACACCTAATCGATCTCTCATGGTATAAAAAAATAGGCCCCGAAGGCCTTGCCGACCTACAAACAATAGTAGCCAAAACCTCAACCACCATT
CACTCAGGATTAATCAAAACCTATCTGGGAACGTTCGCCCTATCTATCCTTACCATTCTATCGACTCAGATCTAAATCCAAATAATGGCCCCCAACATCCGAAAATCTCACCCCGTATTGAAAATAATCAACAATTCCCTAATTGACCTGCCCACCCCATCAAACATTTCTGCC
TGATGAAACTTCGGGTCCCTCCTAGGCATCTGTTTAACCACTCAAATCCTCACTGGCCTTCTACTAGCCATACACTACACTGCCGATACAACCCTGGCCTTTTCATCAGTTGCCCACACATGTCGAGACGTACAGTATGGCTGATTAATTCGTAACCTACATGCAAACGGAGCC
TCTTTCTTCTTCATTTGCATCTACCTACACATCGGACGAGGACTCTACTATGGCTCATTCCTGTATAAAGAAACCTGAAATACCGGAGTCATCCTCCTACTAACCCTAATAGCCACAGCCTTCGTAGGCTACGTCCTGCCATGAGGACAGATATCCTTCTGAGGTGCCACAGTT
ATCACCAACCTATTCTCTGCTATTCCTTATATTGGTCAAACCCTAGTAGAATGGGCCTGAGGCGGATTCTCCGTAGACAACCCTACACTCACCCGATTTTTCGCCCTACACTTCCTCCTCCCCTTCATGATTGCAGGCCTTACTGTAATTCACCTTACTTTCCTCCACGAATCC
GGCTCGAACAACCCACTAGGTATCACGTCAGACTGTGATAAAATCCCATTCCACCCCTACTTCTCCCTAAAAGACATCCTAGGCTTCATAATTATACTCGCCCTACTAACAACCTTAGCCCTATTCTCCCCCAACTTACTAGGTGACCCAGAAAACTTTACCCCAGCAAATCCC
CTAGTCACACCCCCACATATCAAGCCAGAGTGATATTTCCTATTCGCATACGCCATCCTACGCTCAATCCCCAACAAACTGGGAGGAGTACTAGCCCTAGCTGCCTCCGTACTAATCCTATTCCTGATCCCCATCCTCCACAAGTCAAAACAACGCACCATGACATTCCGCCCA
CTCTCTCAACTCCTATTCTGAGCCTTAGCCGCCAACCTTCTCATCTTAACCTGAGTGGGCAGCCAACCAGTCGAACAACCGTTCATCATCATTGGTCAACTAGCCTCAACCACCTACTTCACCATCCTCCTAATCCTACTACCCACCATTGGAGCCCTAGAAAACAAAATACTC
AACTGCTAATCACTCTAATAGTTTATAAAAACATTGGTCTTGTAAGCCAAAGAATGAAGATTATACCCCTTCTTAGAGTTCCAGTCACAAGAGCCAAAATCAGAAAAAAAGGAATTAAACCCTCATCTCCAACTCCCAAAGCTGGTATTTTACATTAAACTACTTTCTGATTGA
TCCTCCACCCTAAACAGCCCGAATCGCCCCCGAGATAACCCACGCACAAGCTCTAACACCACAAACAATGTTAACAATAACCCCCACCCTGCTGCCAAAAACATTCCGGCACCCCGAGAATAAAACATAGCAACTCCACTAAAATCTAACCGAACAGAAGACATACCCCCACC
ATCAACTGTAATCACCCCCAACTTTCAATACTCAACAAATCCTCCAACAACAACCCCAACCACTACCACCAAGACAAAACCTACCCCATACCCCACAACACGCCAATCCATTCAAGCCTCAGGAAACGGATCGGCCGCCAACGACACTGAATACACAAAAACCACCAACATCCC
ACCCAGATATACCATAAATAAAACCAAGACACAAAAGAAACACCCAAACTTAACAACCACCCACAGCCAACAACCGATGCAATAACTAAACCAACCACGCCATAATAGGGCGAAGGATTAGATGCTACCGCTAACCCACCAAAGACAAAACACAACCCTAAAAAAAGCACAAA
ATACGTCATCTTAATTCCCACTCGGCTTTTCTCCAAGGTCTATGGCCTGAAAAGCCATTGTTAAATCTTAACTATAGGAACTTCACCCAGAGAAAGAAATAGAAATTGCCTAAAGGACAAGGCCCCCCCCCCTCCCCCCCCGCAATTTAAGCCTATGCACTTCTTTGCATTACA
CTATCTTCCCCATACCTTAACTTCATGTAAGCAAGGCATTCCTATTCATGTTCTCCAGACATTCTATGTATGCTCTTCCCTTATTGTTCCCTGTAAACAAGACCCTCCCTGTCCTTTCTTCTTAGACCCTCCAACATCCATGCTCTCTGGACCTCTCTCCCTTTCCTCCAGACT
AACGACATTCCCGTGTCTTTCTTGAATGACAATCCAATAGTCCATGGTCCCAGGAATTGCCTCTCTCTTTCCTTCCCCTAAACCCATCCCCTGCTGCTCTTTACCTCTCTCTCCGCTCACCGTGTACGGCTACGCTTGGCGAGGCTCTCTGCACCGTCCCAGGCCATCCCTGCC
CCCAACCCTAACCTCCCGCTGTACCCTCAACACGCGTACTGGGTTATCTCTTGATCTGACCTCTCACGTGAAATCAGCAACCCGGCGTATGGCAGACTCTACGTTACTAGCGTCAGGCCCATTCTTTCCCCCTACACCCTCGCCCAACTTGCGCTTTTGCGCCTCTGGTTCCTA
TGTCAGGGCCATCCCCTGGTATTCTCTCCCAACTTGCTCTTCACCGATACATCTGGTTGGCTATTTATCAACTTCGTCTCTCTTAATCGTGTCACCGAAAAGTTTTCCTCTTTTGGTTCTCTTTTTTTTTCTGGGCGTCTTCACTAAACCCTTCCAGTGCGGCGGGTAAATACAC
TCTGTTGACGTGGGCACACATGCCTGGCGGCCAAACTCTCGCCCTCAAGAATCCCTGAATGAGACGGTGAAAGTATATGGGGAATCATCTTAACACTGATGCACTTTGCTTTGCATCTGGTTATGGAGTATCCACTAACCCTCTACTAGATTACTATTCGATGAATGCTTGGTA
GACATAAATTTTCATTTTTTCACTTCATCCAATTTCCTTTAATTTTAAAAACAACACTAGGGATTTTGCCCTAAATGTTTGTCACGTTTTTTTGCAAATTTTGTTCACATTCGTTCACACCTTTCGTTACGTACACCTCAACGCCAATATTTCATTAATATTCAAATTCGTTTC
GTTCGTTCGCGTTCGTTCGTTTTCGTTCGTTCGTTCGCTCACCACACCCCAACCGTACCTCCAACAACGCTGGCAATTTCCAGCTATATTCACAGACCCCCTCCACCACCATGCCCACACCGTCATCACTCGCCAATACCCAACACCACCGTCCACTTCCGGCACCGAAATTCA
CCCGTAACCCTACACCGTTACGCCACCACTTTTAGTGTTGGCGTTCACCATCCACCGTCCACTTCCGGCACCGAAATTCACCCGTAACCCTACACCGTTACGCCACCACTTTTAGTGTTGGCGTTCACCATCCACCGTCCACTTCCGGCACCGAAATCACCCGTAACCCTACAC
CGTTACGCCACCACTTTTAGTGTTGGCGTTCACCATCCACCGTCCACTTCCGGCACCGAAATTCACCCGTAACCCTACACCGTTACGCCACCACTTTTAGTGTTGGCGTTCACCATCCACCGTCCACTTCCGGCACCGAAATTCACCCGTAACCCTACACCGTTACGCCACCAC
TTTTAGTGTTGGCGTTCACCATCCACCGTCCACTTCCGGCACCGAAATCACCCGTAACCCTACACCGTTACGCCACCACTTTTAGTGTTGGCGTTCACCATCCACCGTCCACTTCCGGCACCGAAATCACCCGTAACCCTACACCGTTACGCCACCACTTTTAGTGTTGGCGTT
CACCATCCACCGTCCACTTCCGGCACCGAAATCACCCGTAACCCTACACCGTTACGCCACCACTTTTAGTGTTGGCGTTCACCATCCACCGTCCACTTCCGGCACCGAAATCACCCGTAACCCTACACCGTTACGCCACCACTTTTAGTGTTGGCGTTCACCATCCACCGTCCA
CTTCCGGCACCGAAATCACCCGTAACCCTACACCGTTACGCCACCACTTTTAGTGTTGGCGTTCACCATCCACCGTCCACTTCCGGCACCGAAATTCACCCGTAACCCTACACCGTTACGCCACCACTTTTAGTGTTGGCGTTCACCATCCACCGTCCACTTCCGGCACCGAAA
TTCACCCGTAACCCTACACCGTTACGCCACCACTTTTAGTGTTGGCGTTCACCATCCACCGTCCACTTCCGGCACCGAAATTCACCCGTAACCCTACACCGTTACGCCACCACTTTTAGTGTTGGCGTTCACCATCCACCGTCCACTTCCGGCACCGAAATTCACCCGTAACCC
TACACCGTTACGCCACCACTTTTAGTGTTGGCGTTCACCATCCACCGTCCACTTCCGGCACCGAAATCACCCGTAACCCTACACCGTTACGCCACCACTTTTAGTGTTGGCGTTCACCACCCACCGTCCACTTCCGGCACCGAAATCACCCGTAACCCTACACCGTTACGCCAC
CACTTTTAGTGTTGGCGTTCACCATCCACCGTCCACTTCCGGCACCGAAATCACCCGTAACCCTACACCGTTACGCCACCACTTTTAGTGTTGGCGTTCACCATCCACCGTCCACTTCCGGCACCGAAATTCACCCGTAACCCTACACCGTTACGCCACCACTTTTAGTGTTGG
CGTTCACCATCCACCGTCCACTTCCGGCACCGAAATCACCCGTAACCCTACACCGTTACGCCACCACTTTTAGTGTTGGCGTTCACCATCCACCGTCCACTTCCGGCACCGAAATCACCCGTAACCCTACACCGTTACGCCACCACTTTTAGTGTTGGCGTTCACCACCCACCG
TCCACTTCCGGCACCGAAATCACCCGTAACCCTACACCGTTACGCCACCACTTTTAGTGTTGGCGTTCACCATCCACCGTCCACTTCCGGCACCGAAATCACCCGTAACCCTACACCGTTACGCCACCACTTTTAGTGTTGGCGTTCACCATCCACCGTCCACTGCCCATCCCA
CCACTACCCGCAAGGATCCCACTAACAAGGCCCCCCCCCTCCCCCCCCGCAATTTAAGCCTATGCACTTCTTTGCATTACACTATCTTCCCCATACCTTAACTTCATGTAAGCAAGGCATTCCTATTCATGTTCTCCAGACATTCTATGTATGCTCTTCCCTTATTGTTCCCTG
TAAACAAGACCCTCCCTGTCCTTTCTTCTTAGACCCTCCAACATCCATGCTCTCTGGACCTCTCTCCCTTTCCTCCAGACTAACGACATTCCCGTGTCTTTCTTGAATGACAATCCAATAGTCCATGGTCCCAGGAATTGCCTCTCTCTTTCCTTCCACTAAACCCATCCCCTG
CTGCTCTTTACCTCTCTCTCCGCTCACCGTGTACGGCTACGCTTGGCGAGGCTCTCTGCACCGTCCCAGGCCATCCCTGCCCCCAACCCTAACCTCCCGCTGTACCCTCAACACGCGTACTGGGTTATCTCTTGATCTGACCTCTCACGTGAAATCAGCAACCCGGCGTATGGC
AGACTCTACGTTACTAGCGTCAGGCCCATTCTTTCCCCCTACACCCTCGCCCAACTTGCGCTTTTGCGCCTCTGGTTCCTATGTCAGGGCCATCCCCTGGTATTCTCTCCCAACTTGCTCTTCACCGATACATCTGGTTGGCTATTTATCAACTTCGTCTCTCTTAATCGTGTC
ACCGAAAAGTTTTCCTCTTTTGGTTCTCTTTTTTTTTCTGGGCGTCTTCACTAAACCCTTCCAGTGCGGCGGGTAAATACACTCTGTTGACGTGGGCACACATGCCTGGCGGCCAAACTCTCGCCCTCAAGAATCCCTGAATGAGACGGTGAAAGTATATGGGGAATCATCTTA
ACACTGATGCACTTTGCTTTGCATCTGGTTATGGAGTATCCACTAACCCTCTACTAGATTACTATTCGATGAATGCTTGGTAGACATAAATTTTCATTTTTTCACTTCATCCAATTTCCTTTAATTTTAAAAACAACACTAGGGATTTTGCCCTAAATGTTTGTCACGTTTTTT
TGCAAATTTTGTTCACATTCGTTCACACCTTTCGTTACGTACACCTCAACGCCAATATTTCATTAATATTCAAATTCGTTTCGTTCGTTCGCGTTCGTTCGTTTTCGTTCGTTCGTTCGCTCACCACACCCCAACCGTACCTCCAACAACGCTGGCAATTTCCAGCTATATTCA
CAGACCCCCTCCACCACCATGCCCACACCGTCATCACTCGCCAATACCCAACACCACCGTCCACTTCCGGCACCGAAATTCACCCGTAACCCTACACCGTTACGCCACCACTTTTAGTGTTGGCGTTCACCACCCACCGTCCACTTCCGGCACCGAAATTCACCCGTAACCCTA
CACCGTTACGCCACCACTTTTAGTGTTGGCGTTCACCATCCACCGTCCACTTCCGGCACCGAAATTCACCCGTAACCCTACACCGTTACGCCACCACTTTTAGTGTTGGCGTTCACCATCCACCGTCCACTTCCGGCACCGAAATCACCCGTAACCCTACACCGTTACGCCACC
ACTTTTAGTGTTGGCGTTCACCATCCACCGTCCACTTCCGGCACCGAAATTCACCCGTAACCCTACACCGTTACGCCACCACTTTTAGTGTTGGCGTTCACCATCCACCGTCCACTTCCGGCACCGAAATCACCCGTAACCCTACACCGTTACGCCACCACTTTTAGTGTTGGC
GTTCACCACCCACCGTCCACTTCCGGCACCGAAATCACCCGTAACCCTACACCGTTACGCCACCACTTTTAGTGTTGGCGTTCACCATCCACCGTCCACTTCCGGCACCGAAATCACCCGTAACCCTACACCGTTACGCCACCACTTTTAGTGTTGGCGTTCACCACCCACCGT
CCACTTCCGGCACCGAAATTCACCCGTAACCCTACACCGTTACGCCACCACTTTTAGTGTTGGCGTTCACCATCCACCGTCCACTTCCGGCACCGAAATTCACCCGTAACCCTACACCGTTACGCCACCACTTTTAGTGTTGGCGTTCACCATCCACCGTCCACTTCCGGCACC
GAAATTCACCCGTAACCCTACACCGTTACGCCACCACTTTTAGTGTTGGCGTTCACCATCCACCGTCCACTTCCGGCACCGAAATCACCCGTAACCCTACACCGTTACGCCACCACTTTTAGTGTTGGCGTTCACCATCCACCGTCCACTTCCGGCACCGAAATTCACCCGTAA
CCCTACACCGTTACGCCACCACTTTTAGTGTTGGCGTTCACCATCCACCGTCCACTTCCGGCACCGAAATCACCCGTAACCCTACACCGTTACGCCACCACTTTTAGTGTTGGCGTTCACCATCCACCGTCCACTTCCGGCACCGAAATTCACCCGTAACCCTACACCGTTACG
CCACCACTTTTAGTGTTGGCGTTCACCATCCACCGTCCACTTCCGGCACCGAAATCACCCGTAACCCTACACCGTTACATATCTAACGTACATACCAAACCCACAGACTTAAACCAACAACAAATCAACAACAACAAATCAACAACAACAAATCAACAACAACAAATCAACAAC
AACAAATCAACAACAACAAATCAACAACAACAAATCAACAACAACAAATCAACAACAACAAATCAACAACAACAAATCAACAACAACAAATCAACAACAACAAATCAACAACAACAAATCAACAACAACAAATCAACAACAACAAATCAACAACAACAAATCAACGA**GTCCCTG**
TAGCTTACAACAAAGCATGGCACTGAAGATGCCAAGATGGCTGCCACACAACACCCAAGGACAATAGACTTAGTCCTAACCTTACCGTTAATTTTTGCTAGGCATATACATGCAAGTATCCGCATCCCAGTGTAAATGCCCTTCGCTTCTTGCCAAGACAGAAGGAGCAGGTAT
CAGGCACACCCACAGTAGTAGCCCAAGACGCCTTGCTTGGCCACACCCCCACGGGTACTCAGCAGTAATTAACCTTAAGCAATGAGTGTAAACTTGACTTAGCCATAGCAACTCAGGGTCGGTAAATCTTGTGCCAGCCACCGCGGTCATACAAGAGGCCCAAATTAACAGCAA
ATATACGGCGTAAAGAGTGGTACACATGTTATCTCGGCAGCTATGACCGAAATGCAACTGAGCTGTCATAAGCCCAAGATGCACTTAAGGTCACCCCTCAAAATGGTCCTAGCACCCATGATTAATTAAACCCCACGAAAGCTAAGACACAAACTGGGATTAGATACCCCACTA
TGCTTAGCCCTAAATCTTGACACTTACCTCACCGAAGTGTCCGCCTGAGAACTACGAGCACAAACGCTTAAAACTCTAAGGACTTGGCGGTGCTCCAAACCCACCTAGAGGAGCCTGTTCTATAATCGATGATCCACGATACACCCGGCCTCCCCTTGCCATGGACAGCCTACA
TACCGCCGTCGCCAGCCCACCTCTACTGAAAGAACAGTAGTGAGCATAATAGCCCTAATCCCGCTAACAAGACAGGTCAAGGTATAGCCCATGGGGAGGAAAGAAATGGGCTACATTTTCTAGAATAGAAAACCAACGAAAAGGGTCTATGAAACTACCCCTAGAAGGCGGATT
TAGCAGTAAAGCAGGGCAATAATACCTTCTTTAAGTTGGCTCTAGAGCACGTACATACCGCCCGTCACCCTCCTCATAAACAACTACCCCAAATAACTAATACACCCCACCAGTCAAAGACGAGGTAAGTCGTAACAAGGTAAGTGTACCGGAAGGTGCACTTAGCACAA

Pterocles burchelli (Pterocliformes/Pteroclidae) mitogenome from ND5 to 12S rRNA (MN356340.1 - re-annotated)

GO-ND in Figure 8

[illegible]

Pterocles gutturalis (Pterocliformes/Pteroclidae) mitogenome from ND5 to 12S rRNA (CM020177.1 - annotated)

GO-NB in Figure 8

[illegible]

GAACAAAAGACTTAGTCCTAACCTTACTATTAATTTCTGCCAGACATATACATGCAAGTATCCGCGTCCCAGTGTAAATGCCCTTGATCTCTTAATAATAAGCAAAAGGAGCAGATATCAGGCACACTCACTAAGCGTAGCCCAAGACGTCTTGCTCAGCCACACCCCCACGGG
TATTCAGCAGTAATTAACATTAAGCAATAAGTGCAAACTTGACTTAGTCATGGCAATATTCAGGGTTGGTAAATCTTGTGCCAGCCACCGCGGTCACACAAGGGACCCAAATTAATTGTACTCGGCGTAAAGAGTGGTTATATGCTATCATACTGACTAAGATCAAAGTATAGC
TAAGCTGTCATAAACTTATGATATACCTAAGACCCATCTTAAAACGATCCTAGCCCTGACGACTAATTGAACCCACGAAAGCTAAGGCACAAACTGGGATTAGATACCCCACTATGCTTAGCCCTAAATCCTGATACTAATTCAACCAAAGTATCCGCCTGAGAACTACGAGCA
CAAACGCTTAAAACTCTAAGGACTTGGCGGTACCCCAAACCCACCTAGAGGAGCCTGTCCTATAATCGATAACCCACGATACACCTAACCGCTCCTTGCCAAGACAGCCTACATACCGCCGTCGCCAGCTCATCTCCACTGAGAGACCAGTAGTGAGCATAATAGCCCTCACCT
CGCTAGCAAGACAGGTCAAGGTATAGCTAATGGATGGAAGATGGGCTACATTTTCTAAACTAGAGAACTACGAAAAGAGGTGTGAAACCACTCTGGAAGGCGGATTTAGCAGTAAAACAGGATAATAATACCTGCTTTAAGCTGGCCCTGGGGTACGTACACCGCCCG
TCACCCTCCTCTCAAGCCACAGCCTCTACTCAATAAATAATATGCCTCCCTGGCTGAAGATGAGGTAAGTCGTAACAAGGTAAGTGTACCGGAAGGTGCACTTAGCACAT

Pterocles gutturalis (Pterocliformes/Pteroclidae) mitogenome from ND5 to 12S rRNA (MN356147.1 - re-annotated)

GO-NC in Figure 8

ATGGAGACCTTACTCCTCAACACCTGCATATTTCTCACACTAGCAACCATTCTCACACCAATTCTTCTGCCACTCCTCTCAAAAACATTCCAAAACTCTCCAACCATCATCACACGCACTGTTAAAACGCCTTCCTCATTAGCCTAGTACCAATAACAGTCTTCGTATACTCT
GGCACAGAAAGTATTACCTCCAGCTGAGAATGAAAATTTACTATAAACTTTAAAATCCCACTAAGCTTCAAAATAGACCAATACTCCACAATATTTTTCCCCATCGCCCTATTCGTAACATGATCCATCTTGCAATTCGCAACATGATATATGGCCTCGGAACCACACATTACA
AAATTCTTCTCTTACCTCCTGACATTCCTTATTGCAATGCTCATACTAACCATTGCCAACAATATGTTTCTTCTTTTCATTGGCTGAGAAGGCGTAGGAATTATATCATTTTTACTAATCAGCTGATGACAAGGCCGAGCAGAAGCCAATACGGCCGCACTCCAAGCCATCATC
TATAACCGAATTGGAGATATCGGCCTCATTCTAAGTATAGCATGACTTGCATCAACCCTTAACACCTGAGAAATCCAACAACCCCCCTCCCCCGGCCAAATCCCAACACTTCCACTACTCGGCCTAATCCTAGCTGCCACAGGAAAATCAGCTCAATTCGGCCTCCACCCATGA
CTACCAGCAGCCATAGAAGGCCCAACCCCAGTCTCTGCCCTACTTCACTCCAGCACTATAGTAGTAGCCGGAATTTTTCTTCTCATTCGCATACACCCCTCTTTACTGATAACCAAACAGCTCTCACACTATGCCTATGCTTAGGTGCCTTATCCACACTATTTGCCGCTTCA
TGTGCTCTAACACAAAATGATATTAAAAAAATCATCGCATTCTCAACATCCAGCCAACTAGGACTAATAATAGTTGCTATCGGTTTAAACCTTCCACAACTAGCCTTCTTCCACATTTCAACACATGCATTCTTCAAAGCTATACTATTTCTATGTTCAGGATCAATCATTCAC
AACCTCAACGGTGAACAGGACATTCGAAAAATAGGAGGCCTACAAAAAACTCTCCCAACAACCACCTCCTGCCTAACTATTGGTAATCTAGCTCTAATGGGAACCCCCTTTTTAGCGGATTTTACTCAAAAGACCTAATCATCGAAGCTTAAACACTTCCACCTAAATGCA
TGAGCACTACTCATAACACTCCTAGCAACCTCTTTTACTGCAACCTACAGCCTACGTCTAACCATGCTAGTACAAACAGGATCCACACGCGTGTCAACAATCACTCCAATAAATGAAAATAACCTAACAATCACTCGCCCTATCATCCGCCTTACCTTAGGCAGCATCATGGCC
GGCTACTTATACATCATATATTCTACCCACCAAAACACTTCTACCCTCACAACCATGCCCCTTCCAACAAAACTACAGCAGCCATTATCATTATACAATTACTAGGCTCCATCCTAGCCTCTACTAGCACTAATACCCTCCAACCCTTATCCCAACCCAACCCAAGCAAAACGCCTACTAAACTTT
TCATCCACATTAGGATATTTAACCCCCTAATACACCGCATCTACTCCACCAAACTACTAAATAACGGACAAAAGATTGCTTCACACCTAATCGACCTAACCTGATACAAAAAGATAGGACCCGAAGGACTCACCGACCTACAACTTAATAACTAAACCCTTAACTATCCTC
CACACCGGATTAATCAAGACATACCTAGGAACATTCGCTCTGTCCATCCTTATTACAATCATCCTGTACAACCATAACCTAATGGCCCCTAACCTACGCAAATCCCACCCCCTACTAAAAATAATTAACAGCTCTTTAATTGACCTCCCACCCACCAACCATATTCTGCCTG
ATGAAACTTTGGATTCCTGTTAGGCATATGCCTAACAACTCAAATCCTAACCGGTCTCCTCTTAGCTATACACTACACTGCAGACACAACCCTAGCCTTCTCATCAGTCGCCCACACATGCCGAAACGTCCAGTATGGCTGATTAATTCGTAATCTACATGCCAACGGCGCTTC
ATTCTTCTTCATCTGCATCTATCTCCACATCGGACGAGGACTCTACTATGGATCTTACCTGTATAAAGAAACCTGAAACACGGGCATTATCTTGTTACTTACCTTAATAGCAACTGCTTTCGTAGGCTACGTCCTCCCATGAGGACAAATATCCTTCTGAGGAGCCACAGTCAT
CACTAATCTCTTCTCAGCCATCCCATACATTGGCCAGACCCTTGTAGAATGAGCCTGAGGCGGGTTTTCAGTAGACAACCCTACCCTGACCCGATTCTTCGCCCTACACTTCCTCCTACCCCTTCCTAATTGCAGCTTAACACTCATCATCTCACTTCTTACATGAGTCTGG
GTCAAATAACCCACTAGGAATTTCATCCAACTGTGACAAAATCCCATTCCACCCCTACTCCTCTATTAAAGATACCCTTGGACTTGCACTCCTACTACTCCATGATAACCCTAGCCTTATTCACACCAAACCTCCTAGGAGACCCAGAAAACTTTACGCCAGCAAACCCACT
AATTACACCTCCCATATCAAACCAGAATGATATTTCTTATTTGCATATGCCATCCTACGCTCAATCCCTAACAAATTAGGGGGAGTATTAGCCCTAGCTGCATCAGTACTAATCCTATTCCTAATCCCATTCTCGCATAAATCAAAACAACGTACAATGACTTTCCGCCCACT
CTCCCAATTCCTATTCTGAACTTTAACAGCCAATCTCCTTATCCTTACATGAGTAGGAAGCCAACCAGTAGAACACCCCTTTATCATCATTGGACAAGTAGCTTCCCTCACCTACTTTAGCATTCTCCTCATCCTACTTCCAATCACTGGCACTCTGGAAAATAAAATACTCAA
CTACTAAAATACTCTAATAGTTTAACAAAAAACATTGGTCTTGTAAACCAAAGAATGAAGAATAATCCCCTTCTTAGAGTTCCAAACCACTTACAACCAAAAGCCAAACACTTACCACTAACCCTAGGCCGATATAAACCAAACTACATCCACCTAACCCAACTACCCAAATT
CTCCTCCCTTTTTTCCACTTATTAACCTAAATAACCCCTACGTCACATAACACCATCCAAACCAACCCCCTACCCATAATAACCCCCAATACCACTAAAAACGAAACCCAGAACTAAGCGGCGCGCACCTAAAACACTATATCTTTAAAAATAAGCCAACAACTTTCATCCAA
CCCGCCCCCAAATTGCTTAACCTAAAAAACCTCTCCACATACCCTAAAAAGTCACCCTATATTCCCCNTTTTTCCNCTTATTAACCTAAATAACCCCTACGTCACATAACACCATCCAAACCAACCCCCTACCCATAATAACCCCCAATACCACTAAAAACGAAACCCAGAA
CTAAGCGGCGGCGCACCTAAAACACTATATCTTAAAAATAAGCCAACAACTTTCATCCAACCCGCCCCCAAATTGCTTAACCTAAAAAACCTCTCCACATACCCTAAAAAGTCACCCTATATTCCCCNTTTTTCCNCTTATTAACCTAAATAACCCCTACGTCACATAACA
CCATCCAAACCAACCCCCTACCCATAATAACCCCCAATACCACTAAAAACGAAACCCAGAACTAAGCGGCGCGCACCTAAAACACTATATCTTTAAAAATAAGCCAACAACTTTCATCCAACCCGCCCCCAAATTGCTTAACCTAAAAAACCTCTCCACATACCCTAAAAAGT
CCACCCTATATTCCCCNTTTTTCCNCTTATTAACCTAAATAACCCCTACGTCACATAACACCATCCAAACCAACCCCCTACCCATAATAACCCCCAATACCACTAAAAACGAAACCCAGAACTAAGCGGCGCGCACCTAAAACACTATATCTTTAAAAATAAGCCAACAACT
TTCATCCAACCCGCCCCCAAATTGCTTAACCTAAAAACTCCCCCCCTACC*CCCCCGGCTCTAATGTGCCGGCTTATGTGTTAATGCGCATTATATTATAGTCCACATTATACATGCATGTAACTTAGGGAAATCCATTAAGTGAATGTACCAAAAACATACCCTGTATGCAT*
*AATATCCA*TAAC***AATGTCCATCGGGCATAC***ATATCATTCCCCCCCTTCCCCCCAGGGGAATTCCTAGTAGTTTCCAAACCAAAGGACCGGTAATAGCCACGTACTAAACCCATTGTACCGATACTTATACATACCCTACTCCTAGCATACGGAAGTGCTAGAGTACAACCCA
TGCATGGTAGCAGGACAAACTACTTCATAATTCTCTCGTAGGACCGGTGTCAGGTATTAGGTTATCTATTAATCGAGCTTCTCACGAGAAATCAGCAACCCGGTGTTTGTAAATATCTTCACGACCAGCTTCAGGACCATTCTTTCCCCCTACACCCTCGCCCATCTTGCTCTT
TTGCGCCTCTGGTTCCTCGGTCAGGAACATAACTTGGCTTCTCACCCTAACTTGCCCTTCACAGATACATCTGGTTGGGGATATTAATCAACATGACTCTTCTTAATCGTGTCACCCCCCTCTTTTTCACTTTTGGTTCTCTTTTTTTTGGGGTATCTTCAACGTGCCCCCCGG
TGCGGCGGGTGAATACAATCTCTTGACCTGAGCATACAATGCCCGTCGGCCTTTTTTCTGGCCTTCAAGAATCCCTTAATGAGACGGTTGAAGTGTATGGGGAATCATTTTACACTGATGCACTTTGTATAGCATTTGGTTATGGAGTATCCTCTATCTCTTGTCTATGTTGC
TATTTGGTGAATGCTTGTTAGACATATTTTTTCACTTTCATTTCCTCTACTTTCTTAACAAAACTAGGAGTTTTCAACTAAATTTTCCATCATCAAATTTTTTCATCTTTTATCATGAATTTTATCCATGCAAATATTCGTATTCATTGGCACTGGAGTTACATTAGTAAAACA
AACGAAACATCGTTCGTGTTTTATCGTTGTTTTATCATCATTTCATCATCATAATCATCATAAATTTCCGCTGAAAAACCAAGTAAACAAATACAAACCCATTTCACATCATCATACCTTCATCATAACAAACCCAATCTAAACTAACACCACTGAAAACCTTACTTAACTCAC
AAAATACATACCACATTTCATCATCAATCACATCTAACCCTTACAATTATCACCCTCAATACCTACTACTACCAACCACAACCTCA*TAAAACCAAACATTTACCTACTAAATGCCCTAATAACTTAAGCATTAATCCAATAAACCCAAACTT*CCACTCATTCCCCTGTAGTCAGAAAAGA
AGGACTTTAACCTCCACCGCCAACCCCAAAGCTGACGTTCTACACTAAACTATTTTCTGTTCTAAACCATACTATTCAATCTAACTGCCCGAATTGCTCCCCGAGATAATCCTGTACAAGCTCCAACACAACAAACAAAGTCAATAGTAANCCNCNCCCTGCCACTAAAAG
CATCCCCACCCCACATGAATACAACATAGCCACCCCACTAAAATCCATCTCGGACAGAAAACATACCAATACTATCAATCGTAACCAACCCAAACTTCCATCCCTCAACAGACCCACCCACAATAAACCCGACTACAAGTACTACAACAAAACCTACACTATACCCACGACACG
TCAATCCCCCCAAGCCTCCGGAAAAGGCTCGGCTGCCAAAGACACAGAGTAAACAAACACTACCAGCATTCCCCCTAAGTAAACTATAAACAACACTAACGCCACAAAAGAAAGCCCCAAACTTAATAATCACCCACAACCTACAACAGATGCCACCACCAAACCTACTACCCC
ATAATAAGGTGAAGGATTGGACGCCACTGCTAACCCCCCCAAAGCAAAACTACCCTAAAAACAGTACAAAATAAGTCATAACAATTTCTGCTTGGTCTCTCTCCAAGACATGTGGCCTGAAAAGCCACTGTTGTAAACCTCAACTACAGAAAACTCAAAATACTGAACTTATC
CTTCCTCTTAACATAAGAAATAAACATACTCCTCCTAATCCAAATACACGACGTCCATTAACCTAACACCTTAGTCCCCAAAACCTTTAACAAATCAACAATACATAAATATCCATCTCAAAACCAACCATTAAGTGTTCACTATATCATTTATATCATTTATATCATTTATAT
CATTTATATCATTTATATCATTTATATCATTTATATCATTTATATCATTTATATCATTTATATCATTTATATCATAAACGATACGACAAACGATACGACAAACGATACGACAAACGATACGACAAACGATACGACAAACGATACGACAAACGATACGATTNTAAACGATACAACAAACGGTA
CGACAAACGATACGACAAACGATACGATTAAACGATACAACAAACGGTACGACAAACGATACGATTAAACGATACAACAAACGG**TTCTTATAGCTTACAATAAAGCGTAGCACTGAAGATGCTAAAACGGCCCCTAATTACGTGCCTGAGAACA**AAAGACTTAGTCCTAACCT
TACTATTAATTTCTGCCAGACATATACATGCAAGTATCCGCGTCCCAGTGTAAATGCCCTTGATCTCTTAATAATAAGCAAAAGGAGCAGATATCAGGCACACTCACTAAGCGTAGCCCAAGACGTCTTGCTCAGCCACACCCCCACGGGTATTCAGCAGTAATTAACATTAAG
CAATAAGTGCAAACTTGACTTAGTCATGGCAATATTCAGGGTTGGTAAATCTTGTGCCAGCCACCGCGGTCACACAAGGGACCCAAATTAATTGTACTCGGCGTAAAGAGTGGTTATATGCTATCATACTGACTAAGATCAAAGTATAGCTAAGCTGTCATAAACTTATGATAT
ACCTAAGACCCATCTTAAAACGATCCTAGCCCTGACGACTAATTGAACCCACGAAAGCTAAGGCACAAACTGGGATTAGATACCCCACTATGCTTAGCCCTAAATCCTGATACTAATTCAACCAAAGTATCCGCCTGAGAACTACGAGCACAAACGCTTAAAACTCTAAGGACT
TGGCGGTACCCCAAACCCACCTAGAGGAGCCTGTCCTATAATCGATAACCCACGATACACCTAACCGCTCCTTGCCAAGACAGCCTACATACCGCCGTCGCCAGCTCATCTCCACTGAGAGACCAGTAGTGAGCATAATAGCCCTCACCTCGCTAGCAAGACAGGTCAAGGTAT
AGCTAATGGATCGGAAGAGATGGGCTACATTTTCTAAACTAGAGAACTACGAAAAGAGGTGTGAAACCATCTCTGGAAGGCGGATTTAGCAGTAAAACAGGATAATAATACCTGCTTTAAGCTGGCCCTGGGGTACGTACACCGCCCGTCACCCTCCTCTCAAGCCACAGCC
TCTACTCAATAAATAATATGCCTCCCTGGCTGAAGATGAGGTAAGTCGTAACAAGGTAAGTGTACCGGAAGGTGCACTTAGCACAT

Puffinus lherminieri (Procellariiformes/Procellariidae) mitogenome from ND5 to 12S rRNA (MH206162.1 - re-annotated)

GO-FD in Figure 8

GTGGAAACTACATTACTCCTTAACACCTCCATACTCTTAACACTAGCAATCATCCTCACTCCAACAGTACTCCCTCTTCTATCAAAAACACTCCAAAACTCCCCAACCCACATCACACGCACTATCAAGACTGCCTTCCTAACTAGTCTCATCCCAATAACCCTCTTCATACAC
TCAGGTATAGAAAGCATTACCTCTTACTGAGAATGAAAGTTCATCATAAACTTCAAAATCCCACTCAGCTTCAAAATAGACCAATACTCAATAATTTTTCCCATTGCCCTATTCGTAACATGATCCATCCTTCAATTTGCAATATGATATATAGCCTCAGAACCTCACATT
ACAAAATTCTTCACCTATCTACTAATATTCCTAATCGCCATACTAATACACTAACCATTGCCAACAACATATTCCTACTGTTCATTGGTTGAGAGGGGGTCGGAATCATATCATTCCTACTAATCGGTTGATGACAAGGACGAGCCGAAGCCAACACAGCTGCCCTTCAAGCCGTA
TTATACAACCGAATTGGAGACATTGGTCTAATCCTAAGTATAGCATGACTTGCCACAACCATAAACACTTGAGAACTCCAACAAACTCTCTCCCCCACCCAAACCCCAACCCCTCCCCTCCTTGGTCTTATCCTTGCAGCCACAGGAAAATCAGCTCAATTTGGCCTTCACCCC
TGACTGCCAGCTGCCATAGAAGGCCCTACCCCAGTCTCCGCCTTACTCCACTCCAGCACTATAGTAGTAGCCGGAATTTTCCTACTCATTCGCACCCACCCCATACTTGCTAGCAATCAAACTGCCCTCACCCTATGCCTATGCTTAGGTGCCCTATCCACCTTATTCGCCGCT
ACATGTGCTCTCACACAAAACGACATCAAAAAAATCATTGCTTTCTCCACATCTAGCCAACTAGGACTAATAATGGTCACTATCGGACTAAACCTCCCCACAACTAGCCTTCTTCCATATCTCAACACATGCCTTCTTTAAAGCTATACTATTCCTTTGTTCAGGATCAATCATC
CATAGTCTTAATGGAGAACAAGACATTCGAAAAATAGGAGGCCTACAAAAAATGCCTCCAACAACCACCTCATGTTTAACCATTGGTAACCTGGCCCTAATGGGGACCCCATTTCTAGCAGGATTCTACTCAAAAGACCTCATCATTGAAACCTAAATACCTCATCCTAAAC
ACCTGAGCACTACTGCTAACCCTCCTAGCTACCTCATTCACCGCAACCTATACCTACGTATAACCCTACTAGTCCAAACAGGATTCACCCGTACACCCTCAATCACCCCAATAAACGAAAACAACCCAACTATCATTAACCCAATTACCCGCCTCGCCCTTGGCAGCATCCTA
GCTGGTCTACTCATCACATCATCACATCATCCCTACAAAAACTCCCCAATAACTATACCTATATTAACAAAAACCGCAGCCATCCTAATCACAACCCTGGGTATTATCCTAGCCCTGGAACTCTCAAGCATAACCCACACCATTACTCAACCCAACCAAAACACCTACTTAAAC
TTTCTTCCACATTAGGTTACTTTAACCCACTAATACATCGTCTCAGCTCCTCAAGTCTACTAAATACTGGACAAAAAATCGCTTCACACCTAATCGACCTCTCCTGATATAAAAAATAGGTCCCGAAGGACTGCCGACTACAACTCATAGCAACCAAAACCTCAACCAACC
CTCCACACAGGACTAATCAAAACTTATCTAGGGTCTTTCGCCCTCTCCATCCTCATTATCCTTATCAACATACAGACCGAAACCCAATTAATGGCCCCAAACATCCGAAAATCCCATCCCCTACTAAAAATAATCAACAACTCCTTAATTGACCTACCTGCTCCCTCGAACATCT
CCGCCTGATGAAACTTTGGCTCTCTCCTAGGCATCTGCCTACTAACCCAAATTTTAACTGGCCTACTACTAGCTATACATTATACTGCTGATACAACTCTAGCCTTTTCATCCGTCGCCCATACATGCCGAAACGTACAATATGGCTGACTAATTCGAAACCTACATGCAAAC
GGCGCCTCATTCTTCTTCATCTGTATTTACCTACACATCGGACGAGGATTCTACTATGGCTCTTACCTATACAAAGAAACCTGAAACACAGGAGTCATCCTCCTCACTCTCACTCTATAGCAACTGCCTTCGTAGGATATGTCCTCCCTGAGGCCAAATATCATTCTGAGGGGCTA
CAGTCATCACCAACTTATTCTCAGCTATCCCATACATCGGCCAAACCCTCGTAGAATGAGCCTGAGGGGGATTCTCAGTAGATAACCCCACATTAACCCGATTCTTCGCCCTACATTTCCTCCTCCCTTTCATAATCGCAGGACTTACCCTAATTCACCTCACCTTCCTCCATG
AATCAGGCTCAAACAATCCCCTTGGCATCGTATCAAACTGTGACAAAATCCCATTCCACCCCTATTTCACCCTAAAGATATCCTAGGCTTTATACTCCTACTCCTTCCTCACCTAACAACCTAGCCCTATTTTCCCAAACCTACTAGGAGATCCAGAGAATTTTACCCCAGCAA
ACCCGCTAGTCACACCTCCTCATATCAAGCCAGAATGATACTTCTTATTTGCATATGCCATCCTACGCTCAATCCCTAACAAATTAGGCGGAGTACTAGCCCTAGCTGCATCAGTATTAGTCCTATTTCTACCCCCATTCCTCCACAAAGCTAAACAACGAGCAATAACCTTCC
GTCCCCTCTCCCAACTTTTATTCTGAATCCTAGTCGCTAACCTATTCATTCTTAACATGAGTAGGTAGCCAACCAGTAGAGCACCCATTCATCATCATCGGCCAACTAGCCTCCTCACCTATTTCACCATCCTTCTAATCCTCTTCCCCATTACTGGGGCCCTAGAAAATAAAA
TACTTAACTACTAAAACACTCTAATAGTTTACAAAAAACATTGGTCTTGTAAACCAAAGAATGAAGACTATCCCTTCTTAGAGTTAACCACCAAATACACTCAGAAAAAGAGGACTTAAACCTCTATCATCAACTCCCAAAGCTGATATTTTACATTAAACTATTCTCTGC
CCCATATCTACACATAACCCCTAACTGCTCGAATAGCTCCCCGAGACAACCCCGCACAAGCTCCAACACAACAAACAAAGTCAACAGAGACCTACCCCGCCACTAAAAATATCCCTATCCCGGACGAATAAAACATAGCTACACCACTAAAATCTAATCGAACTGAAAACA
TACCACTCCCATCAACCGTAACCACCCCAAACTTTCAACACTCAACAAACCCTCCAACAACAACCCCAACAACCAGCACTAAAACAAACCCACCCCATACCCCACTACACGTCAATCCCCTCAGGCTTCCGGAAAAGGATCCGCCGCTAAAGATACCGAATATACAAAACCA
CTAACATTCCCCCCAAATAAACCATAAACAACACCAAAGACACAAAAGAAACTCCTAAACTTAACAACCACCCACACCCTACAACAGATCCTACCACTAAACCAATCACCCCATAATAAGGTGAAGGATTAGATGCAACCGCTAACCCCCCCAAAACAAAGCACAAACTAGAA
AAAGTACAAAATAAGCCATAGCAATTTCCGCTTGGCTTTTCTCCAAGATCTGTGGTCTGAAAAACCACTGTTAATGAAATTTAACTACAGAAACTCCACAAATCCTCATCCCTCCCTCCTTCTTCTCCTTTTTCCCCCCCCCTCCCCCCCCCAAACTATGCTTGGCTAATAGTT
CTTGTATTTCATTTATTTTTTGACAGCCTATGTATAGATGTACATCCCTCTCTCCCCCACATCTACATCCCATTCAACTTAGGCAAACACATTCAATGCATGTATCCCGCACAACCCATTTACGCGGATTATCTCCCTCCAACCCGGTCGGAACACAAGTACACTTAAGCCC
AATAGTCCCTAATACTAAACACCATCTCTTCCCGTACTAAAACCCATTAAAACTCTCCCTTATACAGTCCAAACACTCCTAGATACGGATACGCTTACCCACACAAAGTAATGGCAACAGGACAAATCCCTTCAACCATCTCTCGACGGACCGGTAGCTATCGAGCTGGGGTTA
TTTATTAATCGTTCTTCTCACGAGAAATCAGCAACCCGGCGTAAGAAAGACTCGGTATTACTAGCGTCAGGACCATTCTTTCCCCCTACACCCCTAGCACAACTTGCGCTTTTGCGCCTCTGGTTCCTATATCAGGGCCATAACTTGGTTGTTCCTCTCTTCTTGCTCTTCACC
GATACATCTGGTTGGCTATTTATCAACATTCTCCCTCTTAATCGCGTCACCTAAATTTCTCCTCTTTTTGGTTCCTTCTTTTTTGGGGCTTCTTCACTCTGCCCTTCCAGTGGATAACGGTAATAACAATCTATAGACGTGAGCATCGCGTGCCCGGCGAACGGGTTTTTGGCT
CTCAAGAGTTGATTAATGAGACGGTTGACGTATATGGGGAATCATCTTGACACTGATGCACTTTGGTTTACATTTAGTTGTGATACCTTTGCAATTACTTATTTATGTTGCTATTTACTGAATGCTTGTATGACATAATTTTCTAAGCACACACTTCCTCTAACTTTCCAAACA
ACACCAGGAACTTTTCTGCTAAGTTACCCACGACACCAACATCACAAATTTTATCATCATTTTTACGAATCATTACCAGCAAAATTTCATTAATAAATTTTGCCATTCTCTTTCCAAAAATCACATCTAGCAAAACTGCATTAATAAATCTTATTATTCATTCCATCACAAAT
TACCACTAGCAAATCACATTAATAAATCATAAACATTCGCTCAATCATCACACTCACCTATTTTCCAACTAAACCATCCAACATAACCATTCATTATATCCTTATCTTTTCTATACGTTATTGGCACAGAAGTTACATTAATAAATTAACCATTCATTTTCTTCATCATCACA
TACCACATATTGCCTTATCACATCCTCTAACTAGGCACCTATCCCTGCACACAACCCCCAACTCCATCTATCTCACCCACCAACATCCTAACACCAAACTCTACCCTAGACTTTAAACTTATACTCGCACCTCTCCATTAAGACCACCCCTACTCCCCAAATACTAAAAAA
AATTCAAATACAAAACACTACTAAACACTCTAATAGTTTACAAAAAACATTGGTCTTGTAAACCAAAGAATGAAGACTATCCCTTCTTAGAGTTTAACCACCAAATACACTCAGAAAAAGAGGACTTAAACCTCTATCATCAACTCCCAAAGCTGATATTTTACATTAAAC
TATTCTCTGCCCCATATCTACACATAACCCCTAACTGCTCGAATAGCTCCCCGAGACAACCCCGCACAAGCTCCAACACAACAAACAAAGTCAACAGAGACCTACCCCGCCACTAAAAATATCCCTATCCCGGACGAATAAAACATAGCTACACCACTAAAATCTAATCGA
ACTGAAAACATACCACTCCCATCAACCGTAACCACCCCAAACTTTCAACACTCAACAAACCCTCCAACAACAACCCCAACAACCAGCACTAAAACAAACCCACCCCATACCCCACTACACGTCAATCCCCTCAGGCTTCCGGAAAAGGATCCGCCGCTAAAGATACCGAATAT
ACAAAACCACTAACATTCCCCCCAAATAAACCATAAACAACACCAAAGACACAAAAGAAACTCCTAAACTTAACAACCACCCACACCCTACAACAGATCCTACCACTAAACCAATCACCCCATAATAAGGTGAAGGATTAGATGCAACCGCTAACCCCCCCAAAACAAAGCAC
AAACCTAGAAAAAGTACAAAATAAGCCATAGCAATTTCCGCTTGGCTTTTCTCCAAGATCTGTGGTCTGAAAAACCACTGTTAATGAAATTTAACTACAGAAACTCCACAAATCCTCATCCCTCCCTCCTTCTTCTCCTTTTTCCCCCCCCCTCCCCCCCCCAAACTATGCTTG
GCTAATAGTTCTTGTATTTCATTTATTTTTTGACAGCCATGTATTGCACACATTAAGATCTTTACCACATCTACATTAATTCAACTTAGGCAAACACATTCAATGCATGTATCCCGCACAACCCATTTACGCGGATTATCTCCCTCCAACCCGGTCGGAACACAAGTACACTT
CTTAAGCCCAATAGTCCCTAATACTAAACACCATCTCTTCCCGTACTAAAACCCATTAAAACTCTCCCTTATACAGTCCAAACACTCCTAGATACGGATACGCTTACCCACACAAAGTAATGGCAACAGGACAAATCCCTTCAACCATCTCTCGACGGACCGGTAGCTATCGA
GCTGGGTTATTTATTAATCGTTCTTCTCACGAGAAATCAGCAACCCGGCGTAAGAAAGACTCGGTATTACTAGCGTCAGGACCATTCTTTCCCCCTACACCCCTAGCACAACTTGCGCTTTTGCGCCTCTGGTTCCTATATCAGGGCCATAACTTGGTTGTTCCTCTCTTCTTG
CTCTTCACCGATACATCTGGTTGGCTATTTATCAACATTCTCCCTCTTAATCGCGTCACCTAAATTTCTCCTCTTTTTGGTTCCTTCTTTTTTGGGGCTTCTTCACTCTGCCCTTCCAGTGGATAACGGTAATAACAATCTATAGACGTGAGCATCGCGTGCCCGGCGAACGGG
TTTTTGGCTCTCAAGAGTTGATTAATGAGACGGTTGACGTATATGGGGAATCATCTTGACACTGATGCACTTTGGTTTACATTTAGTTGTGATACCTTTGCAATTACTTATTTATGTTGCTATTTACTGAATGCTTGTATGACATAATTTTCTAAGCACACACTTCCTCTAACT
TTCCAAACAACACCAGGAACTTTTCTGCTAAGTTACCCACGACACCAACATCACAAATTTTATCATCATTTTTACGAATCATTACCAGCAAAATTTCATTAATAAATTTTGCCATTCTCTTTCCAAAAATCACATCTAGCAAAACTGCATTAATAAATCTTATTATTCATTCC
ATCACAAATTACCACTAGCAAATCACATTAATAAATCATAAACATTCGCTCAATCATCACACTCACCTATTTTCCAACTAAACCATCCAACATAACCATTCATTATATCCTTATCTTTTCTATACGTTATTGGCACAGAAGTTACATTAATAAATTTCTTCCATCATCACATC
ACATCATATCACATATTGCCTTACCACACTCACCTATTTTCCAACTAAAGCATCCAACATAACCATTCATTTTTCTTCATCATCATACATCATATTGCCTTACCACACTCACCTATTTTCCAACTAAAGCATCCAACATAACCATTCATTTTTCTTCATCATACATCATCA
ATTTTCCATCATAAAGCATCCAATATTCATTTTTCTTCATCATACATCACCCACACTCACCTATTTTCCAACTAAAGCATCCAATATAACTATTCATTTTTCTTCCATCATACATCATACATCATATTGCCTTACCACACTCACCTATTTTCCAACTAAAGCATCCAATAT
AACTATTCATTTTTCTTCATCATACATCACATCATATCACATATTGCACACACTCACCTATTTTCCAACTAAAGCATCCAATATAACTATTCGTTTTTTTTCATCATACATCATATCATATCATATTGCACACATCACCTATTTTCCTAAAGCATCCAATATAACTATTCGTTTTTTT
CTTCATCATACATCACATCATATCACATATTGCCTTACCACACTCACCTATTTTCCAACTAAAGCATCCAATATAACTATTCATTTTTCTTCCATCATACATCATCATCATACATCATATCATATTGCACACATCACCTATTTTCCAACTAAAGCATCCAATATAACTATTC
TACCACTCAAATTTTCCAACTAAAGCATCCAATATAACTATTCATTTTTCTTCCATCATACATCATATCATATCATATTGCCTTACCACACTCACCTATTTTCCAACTAAAGCATCCAATATAACTATTATTTTTCTTCCATCATCATACATCATCATATCATATCATATTTTTATCATACA
TCACATTTTTTTTCCAACTAAAGCATCCAATATAACTATCGTTTTTCTTCCATCATACATCATATCATATCATATCATATCGCATGTGTACCACATCACCTATTTTCCAACTAAAGCATCCAATATAACTATTCGTTTTTCTTCCATCATACATCACATCATATCATATCATATTGTTACCACACT
CAAATTTTCCAACTAAAGCATCCAATATAACTATTCGTTTTTTTCCATCATACATCACATCATATCATATCATATTGCACACATCACCTATTTTCCAACTAAAGCATCCAATATAACTATTCGTTTTTCTTCCATCATACATCATCATACATCATATCATATTGCACCATCACCTAT
TTTCCAACTAAAGCATCCAATATAACTATTATTTTTTCTTCCATCATACATCACATCATATCATATCATATTGTTTACCACACTCACCTATTTTCCAACTAAAGTATCCAATATAACTATTATTTTTTCTTCCATCATACATCACATCATATCATATCATATTGCACCTCACCACTAAAGCA
TCAATATATTTTTTCTTCATCATACATCACATCGCACCCACCTATTTTCCAACTAAAATCCAATATAACTATTTTTTTTCTTCCATCATACATCACATCATATCATATCATATTGTTTACCACATCACCTATTTTCCAACTAAATCATCCAATATAACTATTCTTTTTTCTTCCATCATACATC
ACATCATATCCATATTGTTTACCACACTCACCTGTTTTCCAACTAAACCATCCAATATAACTATTCGTTTTTTTCCATCATACATCACATCATGCACCACACTCACCTATTTTCCAACTAAACCATCCAATATAACTATTCGTTTTTTTTCATCATACATCACATCATATCAT
ACATACTCCTGTTTTCCAACTAAACTATCATCACCAAACCACCCACCATCAACCATCACTAATAATCATATCCAACTCCCCCCCGCCAACCCAAAAATACCCAAATAAAATAATCCCCCCTAAAGCAACAAAATAAACAACAACAAACAACAACAAACAACAACAAACAACAAACAAAC
AACAACAAACAACAAACAACAACAAACAACAAACAACAACAAACAACAACAAACAACAAACAACAACAAACAACAACAAACAACAAACAACAAACAAACAAACAAACAAACT**GTCCTTGTAGCTTAATAACAAAGCATAGCACTGAAGATGCTAAGATGGCTACCCTGCGCACCCAAGGACA**AA
AGACTTAGTCCTAACCTTACCGTTAATTTTACTAAACATATACATGCAAGTATCTGCGCCCCAGTGTAAATGCCCCTGACTTTCTTACTAAGATGACGGGAGCAGGCATCAGGCACACCCACAGCTGTAGCCCAAGACGCCTTGCCCAGCCACACCCCACGGGAACTCAGCA
GTAATTAACCTTAAGCAATAAGTGAAAACTTGACTTAGTTATAGTAATATCTAGGGTTGGTAAATCTTGTGCCAGCCACCGCGGTCATACAAGAGACCCAAATTAACTGTAATCCGGCGTAAAGAGTGGCACCACACTATCACATCAACTAGAATCAAAATGTAACTAAGCTGT
CATAAGCTCAGGATACACCTAAACCCACCCTCAAGATGATCTTAGCATCAATGATCGATTACCTCACGAAAGCTAAGGCACAAACTGGGATTAGATACCCCACTATGCTTAGCCCTAAATCTTGATACTTACCCTACTGAAGTATCCGCCTGAGAACTACGAGCACAAACGCT
TAAAACTCTAAGGACTTGGCGGTGCTCCAAACCCACCTAGAGGAGCCTGTTCTATAATCGATAACCCACGATAAACCTAACCGCTCTTTGCCAAGCAGCCTACATACCGCCGTCGCCAGCTCACCTTCCTGAAAGTATAGCAGTGAGCACAATAGCCCTCAACATCGCTAGC
AAGACAGGTCAAGGTATAGCTATAGAGCGGAAGAAATGGGCTACATTTTCTAGAGTAGAAAACTTACGAAAAGGGGTATGAAACTTCCCCTGGAAGGCGGATTTAGCAGTAAAGCGGGATAATAAATGCCCTCTTTAAGCTGGCCCTGGAGCACGTACATACCGCCCGTCAC
CCTCCTCATAAGCTATAAACCTCTAATAACTAATACACCTACCAGCCAAAGATGAGGTAAGTCGTAACAAGGTAAGTGTACCGGAAGGTGCACTTAGCACAT

Rynchops niger (Charadriiformes/Laridae) mitogenome from ND5 to 12S rRNA (MN356248.1 - re-annotated)

GO-II in Figure 8

[illegible]

Scopus umbretta (Pelecaniformes/Scopidae) mitogenome from ND5 to 12S rRNA (MN356281.1 - re-annotated)

GO-NG in Figure 8

[illegible]

Sterna hirundo (Charadriiformes/Laridae) mitogenome from ND5 to 12S rRNA (CM020500.1 - annotated)

GO-FD in Figure 8

[illegible]

CAACGACAGTGAAATTACATTAAAAACAACAAAGTTTTTTTTTGCCATCCACCTTACCTTTCTCCATGAATCTGGATCGAACAATCCACTGGGTATCCTATCAAACTGCGACAAAATCCCATTCCACCCCTACTTCTCACTAAAAGATATCCTAGGATTCACTATCATATTCC
TCCCACTAACGACCCTAGCTCTATTCTCACCAAATCTGTTAGGAGACCCAGAAAACTTTACCCCAGCAAACCCATTAGTTACACCTCCCCATATTAAACCCGAATGATATTTCCTATTCGCATACGCTATCCTACGCTCCATCCCTAACAAACTAGGGGGTGTATTAGCTTTAG
CAGCCTCCGTACTAGTACTATTCCTAGCCCCTACTCCATAAGTCCAAACAGCGTTCAATAACCTTCCGTCCCCTCTCCCAGCTCCTATTCTGAATCCTAGTCGCCAATCTCTTTATCCTGACATGAGTAGGCAGTCAACCCGTAGAACATCCATTCATCATCATCGGACAACTA
GCTTCCTTAGCCTACTTCACCATCCTACTGCTCCTATTCCCCGCTATTGGCACCCTAGAAAATAAAATACTTAATTACTAAAAACACTCTAATAGTTTATAAAAAACATTGGTCTTGTAAACCAAAGACTGAAGACTATACCCCTTCTTAGAGTTTCCCCAATCAGAAAAAGAG
GACTTAAACCTCTATCTCCAACTCCCATTGCGTGCTCCAGCCAATTTTGTACTAAACTATTCTCTGGACCCCTCCCCCTAAACCGCTCGAATTGCCCCACGAGACAACCCCCGTACTAACTCTAACACAACAAACAGAGTTAGCAACAACCCCCAACCCGCAACCAAAAACAT
CCCCACACCACACGAATAAAACATCGCCACCCCACCAAAATCTAACCGAACAGAAGACATGCCCCCACAGTCCACTGTAACAACCCCAAGATCCCAACATTCAACAACCCCACTAACAACTACCCCACACACAAGTACTAAAACAAACCCTACCCCATACCCAACAACCCGCCA
ATCCCCCCAAGCTTCCGGAAAGGGATCTGCTGCCAAAGACACAGAGTATACAAAAACCACCAGCATCCCCCCCAAATACACCATAAACAATACCAATGACACAAAAGAAACCCCTAAACTCAACAACCACCCCACACCCTGCAATAGAAGCCAACACCAAACCAACTACCCCATA
GTAGGGAGAAGGATTAGACGCAACTGCTAAACCTCCCAACACAAAGCATAACCCTAAAAAAAGCACAAAATAAGTCATAGCAGTTCCTGCTTGGCTTCTCTCCAAGACCCGCGGCCTGAAAAGCCGCTGTTGTTTAACCTCAACTACAGAAACCATAAGAGCCCCCCCCTCCC
CCCCCCCCCCTCCCCCCACGACACAGCCCGAAATATACGACCTCATGTACTGTTTTACATTAAACTACTATCCACATATATACATACAGTCCATGTCTAATAGTCATTAATATCCTAACTGACATCCCCTCCCCAAACCCATGCCCGCTTCCAGAGGACCTAACACCCATCCAA
CTCGGAATATCCGTTAACAACTCTCGTACTAATACCATTTCCTAGCTTGGCTTTACATACCTCCTTCAAGGGTACGGCAGTGCCTGCTATACCTCCCTGCATGAATTCCAGTACCTCGCACCGAGTCCCTCTCCACAGAATAAGATTCACCAGACTCTCGACGTACACAAAGCT
TCGTACCAGGTGGATTTATTAGTCGTACTCCTCACGTGAAATCAGCAACCCGGTGTTGGTAAGATCCTACGTTACTAGCTTCAGGACCATACTTTCCCCCTACACCCCTAGCCCATCTTGCTCTTTTGCGCCTCTGGTTCCTATGTCAGGGCCATAAATAGGTTAGTCCTCTCA
ACTTGTACTTCACCGATACATCTGGTCGGCTATTGATCAACATCTCACCCGTGATCGCGGCATCCGACCTTTTTGGGGCTGTTGGTTCCCTTTTTTTTTCTGGGCGTCTTCACAGGTTGCCCCTCAGAGTGCGGAGCAGGTGTATACAATCTAAGACATGTGCCCTCCCTGGTAT
GCGTACGGATCTCGCCCCCAAGGATGCCTCAATGAGACGGTTGTAGGGTTAGGGGAATCATTTTTGCACTGATGCACTTTGTTTTACATCTGGTTATGGTGTGCCCCCAAACAGCTTACTAAGTCTGCTATTTAGTGAATGCTTGCTGGACATAATTCGTCATTTTTCATTTCC
TCTAATTTTCTAAACAAAACTAGGAGGTTCTAACTGAAAAATGAACCGTATTTTCATCACACATTTTATCATCATCTTTGTTACACGTCAACGACAGTGAAATTACATTAAAAAAAGACTACACACAATCTGTATTCACATATTCGAATAACACATCACAAATTATAAAAGAAA
TCACCCTAAAAATAACACAAACAACAAACAACAAACAACAAACAACAAACAACAAACAACAAACAACAAACAACAAACAACAAACAACAAACAACAAACAACAAACAACACCTA**GTCTTTGTAGCTTATTAGACAAAGCATGGCACTGAAGATGCCAAGATGACTACCCCTACATGTCCGA**
AGACAAAAGACTTAGTCCTAACCTTACTGTTAATTTTTGCTCGACACATACATGCAAGTATCTGCGCCCCAGTGTAAATGCCCTCATGCCCCTTACCAAGGAGAGAGGAGCAGGTATCAGGCACACCCACAGCTGTAGCCCAAGACGCCTTGCTCTGCCACACCCCCACGGGTA
CTCAGCAGTAGTTAACCTTAAGCAATAAGTGTAAACTTGACTTAGTTATAGCAAACCTAGGGTTGGTAAATCTTGTGCCAGCCACCGCGGTCACACAAGAGACCCAAATTAACTGTATACGGCGTAAAGAGTGGAACTATGATGTCATAATAACTAGGATCGAAATACAGCTAA
GCCGTCATAAGCTCAAGGTGTACCTAAGACCTCCATTAAGATGATCCTAGCATTCTTGATTAATTTAACTCCACGAAAGCTAGGACACAAACTGGGATTAGATACCCCATTATGCCTAGCCCTAAATCTTGATGTTTATCATACTAAAGCATCCGCCTGAGAACTACGAGCACA
AACGCTTAAAACTCTAAGGACTTGGCGGTGCCCCAAACCCACCTAGAGGAGCCTGTTCTGTAATCGATAACCCACGATTCACCCAACCACCCCTCGCTAAGGCAGCCTACATACCGCCGTCGCCAGCTCACCTCCACTGAGAGCCAAACAGTGAGCACAATAGCTCAACTCGCT
AGCAAGACAGGTCAAGGTATAGCCTATGGGGTGGAAGAAATGGGCTACATTTTCTATAATAGAAAACCCACGAAAGGAGGCATGAAATAGCCTCCGGAAGGCGGATTTAGCAGTAAAGCGGGACAATAGAGCCCCCTTTAAGTTGGCCCTGGGGCACGTACATACCGCCCGTCA
CCCTCCTCACAAGCCACAAACCATCATAACTAATAACCCTTACGGCTGAAGATGAGGTAAGTCGTAACAAGGTAAGTGTACCGGAAGGTGTACTTAGCATAC

Thalassarche chlororhynchos (Procellariiformes/Diomedeidae) mitogenome from ND5 to 12S rRNA (MN356342.1 - re-annotated)

GO-FD in Figure 8

ATGGAGGCTGCACTACTCCTCAACACCCTCATTCTCCTAACACTAACAATTCTCATTACACCTATTCTTCTCCCATTACTATCAAAAAAATTCCAAAGCTCCCCAACCATAATCACACATGCTATCCAAACTGCACTCCTAACAAGTCTCATTCCAATAACGCTCTTCATGCAC
TCAGGCACAGAAAGCATCATCTCCCACTGAGAATGAAAATTTATCATAAACTTCAAAATCCCACTTAGCTTCAAAATGGACCTATACTCCATGATATTCCTCCCCATCGCCCTGTTCGTCACATGATCCATCCTTCAATTCGCGACATGATACATAGCCTCAGAACCACACATT
ACAAAATTCTTCTCCTACTTACTAATATTCCTAATCGCCATACTAACATTAACCATTGCTAACAACATATTCTTACTATTCATCGGCTGAGAAGGAGTTGGAATCATATCATTCTTACTAATTGGCTGATGACAAGGACGAGCTGAAGCTAACACAGCAGCACTCCAAGCCGTG
CTCTACAACCGAATCGGGGACATTGGCCTAATCCTAAGCATGGCTTGACTCGCTTCAACCATAAACACCTGAGAAATCCAACAAGCCATCTCCCCCACCCAAACCCCAACACTCCCCCTACTAGGTCTTATTCTTGCAGCCACAGGGAAATCAGCCCAATTCGGCCTACACCCA
TGATTACCCGCTGCCATAGAAGGTCCAACGCCAGTCTCTGCCCTACTTCATTCCAGCACTATGGTAGTAGCCGGAATTTTCCTACTCATCCGCACTCACCCCATACTTGCCAACAATCAAACCGCCCTCACCCTGTGCTTGTGCCTAGGTGCTCTATCCACACTATTCGCCGCT
ACCTGTGCCCTTACACAAAATGACATCAAAAAAATCATTGCCTTCTCTACATCTAGCCAACTAGGACTAATAATAGTTGCCATCGGACTAAACCTCCCACAACTAGCCTTTCTCCACATTTCAACCCATGCCTTCTTCAAAGCCATACTATTCCTCTGCTCAGGGTCAATCATC
CACAGCCTCAACGGAGAACAAGACATTCGAAAAATAGGAGGCTTACAAAAAACACTTCCAACAACCACCTCATGTTTAACCATCGGTAATCTCGCTCTAATAGGAACCCCATTTTTAGCAGGATTTTACTCAAAAGACCTCATCATTGAAAACCTAAACACATCATACCTAAAC
ACCTGAGCACTCCTCCTTACACTCCTAGCCACATCATTCACTGCAACTTACAGCCTACGCATAACCCTATTAGTCCAAACAGGATTTACCCGCATACCTGTAATCCCTCCAATAAACGAAAACAACCCAACCGTCATCAACCCAATCACCCGCCTTGCCCTAGGCAGCATCCTA
GCGGGCCTACTTATCACAACCTATATTCCCCCACAAAGACCCCTCCAATAACCATACCCACACTTACAAAAACTGCCGCTATCATCGTTACAACCCTAGGCATTATCCTAGCCCTAGAACTCTCAAACATAACCCACACCCTACTTCAACCAAAACAAAACACCTACCTAAAC
TTCTCCTCCATACTAGGCTACTTTAACCCCCTAACACACCGCCTCAGCTCCTCAAACCTACTAAACAGCGGACAAAAAATCGCCTCACACCTAATTGACTTATCCTGATACAAAAAAATAGGCCCAGAAGGACTCGCCGACCTACAACTCATAGCAACCAAAACCTCAACCACA
CTTCACACCGGACTAATCAAAACCTACCTAGGATCCTTCGCCCTATCCATCCTCATCATCCTCTCAATACATAGACCCAAAATCAATGGCCCCCAACCTCCGAAAATCTCACCCTCTACTAAAAATAATCAATAATTCCCTAATCGACCTACCCACCCCCTCAAACATCTCTGC
CTGATGAAACTTCGGATCCCTCCTAGGCATCTGCCTAATAACACAAATCCTAACCGGCCTACTACTAGCCATGCACTACACAGCAGATACAACTCTAGCCTTCTCATCCATTGCCCATACATGCCGAAACGTACAGTACGGTTGATTAATCCGAAACCTTCACGCAAACGGAGC
ATCATTTTTCTTCATCTGTATCTACCTTCACATCGGCCGTGGATTCTACTACGGCTCGTACTTGAACAAAGAAACCTGAAACACAGGAATCCTACTCCTACTCACTCTCATAGCAACTGCCTTCGTAGGATACGTCCTACCATGAGGACAAATATCATTCTGAGGAGCCACAGT
CATTACTAACCTATTCTCAGCCATCCCATATATCGGCCAGACCCTCGTAGAATGAGCCTGAGGGGGATTTTCAGTAGATAACCCCACCCTAACTCGATTCTTCGCTCTACACTTCCTCCTTCCATTCATAATCGCAGGCCTTACCCTCATCCACCTCACCTTCCTTCATGAATC
AGGATCCAACAATCCGCTAGGAATCCTATCAAACTGCGACAAAATTCCATTCCACCCCTACTTTACCCTAAAAGATATCCTAGGGTTCACACTCATATTCCTACCACTAACAGCCCTAGCCCTATTCTCACCCAACCTGCTAGGCGATCCAGAAAACTTCACTCCAGCAAACCC
ATTAGTCACTCCTCCCCACATTAAACCAGAATGGTACTTCCTATTTGCATACGCCATCCTACGCTCAATTCCCAACAAACTAGGCGGCGTACTGGCCCTAGCTGCATCCGTCCTAGTCCTATTCCTAAGCCCCCTTCTCCATAAATCCAAACAACGTACACTGACTTTCCGCCC
ACTTTCCCAACTCTTATTTTGACTCCTAGTCACTAACCTATTCATTCTGACATGAATCGGCAGCCAACCAGTAGAACACCCATTCATTATCATTGGCCAATTAGCCTCTATTACCTACTTCACCATCCTCCTAGTCCTCTTCCCCACCATCGCAGCCCTAGAAAACAAGATACT
TAACTACTAAAACCACTCTAATAGTTTACCAAAAACATTGGTCTTGTAAACCAAAGAATGAAGACTACACCTCTTCTTAGAGTTCCCAATTACCATCGACACACCTCAGAAAAAAAGGACTTAAACCTCTATCTCCAACTCCCAAAGCTGGCATTTTACATTAAACTATTCTCT
GATCTTCCCCCTAAACTGCCCGAATAGCTCCGCACGACAACCCTCGTACCAATTCCAACACAACAAACAAAGTCAACAATAACCCTCATCCTGCTACTAAAAACATCCCTACCCCATACGAATAAAACATAGCCACGCCGCTAAAATCCAACCGAACAGAAAATACCCCTCCA
CTATCAACCGTAACCACCCCCATCTTCCAACACTCTACTAAACCCCAACCTCCCACAACTGCCCCAACAACAAGCACCAAAATAAACCCCACCCCATATCCCACAACTCCCCAACTCCCTCAAGCTTCAGGATAAGGATCTGCTGCCAAAGATACAGAGTACACAAAAACTACC
AACATCCCACCCAAATACACTATAAATAGTACTAACGACACAAAAGAAACTCCTAGACTTAACAACCACCCACACCCTACAACAGATGCCAGTACCAAACCAACCACCCCATAATAAGGTGAAGGATTAGACGCAACTGCCAGCCCCCCTAAAACAAAGCATAACCCCAAAAAA
AGTACAAAATAAGCCATAGCGGTTCCCGCTTGGCTTTTCTCCAAGATCTGCGGCCTGAAAAACCGCCGTTGAACTTCAACCACGAGAACACAAAATAATTTCAAACGAACCCCTCTAGAAGACTCCCCCCCCCTCCCCCCCCAAAGCAGCTTATGTNTNAATGNGCATTACTCT
ATTTACCACATAATACATTACATTAATGTAGGAAATACATTTAATGCATGTACTACGTACATGACCATGTAGACGGGCATACCCCTTTTACCCACTCACGAACCCCCAGAGGACAAATACTTCAATAGTCCCTACCACATACCACTCAAACGGATTAAACCCATAAATTCCAAG
TTCTGTGTATATCTCTGTCCAGAATACGGCAGTGCTCGAATAACATAACCTGAATGGTANCAGAACATAACATGCCATCCCTTCTCGTAGGACCGGTAGCTGTCGGACCAGGTTATTTATTAATCGTTCTTCTCACGAGAAATCAGCAACCCGCTGCATATAAGATACTGCGTT
ACTAGCTTCAGGACCATTCTTTCCCCCTACACCCCTCGCACGACTTGCACTTTTGCGCCTCTGGTTCCTCTGTCAGGGCCATAACTTGGTTAGTCCCCTCTCCTCACTTTTTACGAAGTCATCTGGTTGGCTATATATCACCATTTAGTCCGTGATCGCGGCATTTCCTCTTC
TTAGCACTTTTGGTTCCCTTTTTTTTTGGGGCGTCTTCAGGCTGCCCTCCCAGCGCAACGGGTGCTTACAATCTAAGACCTGAGCATACAATGTGTTGCGGTCTGATCTTGGCCCTCAGGAATCCCTGAATGAGACGGTTGAAGTATATGGGGAATCATCTTGACACTGATGCA
CTTTGTTTTCCATTTGGTTATGGTGTATCCACAAACCCTTATTATGTTGTTATTTAGTGAATGCTTGTTAGACATAATTTCTTACTTTTTCTTGCTCTTACATTTCCTCTAACTTTCTAAACAACACTAGTAACTTTTCAACTAAATTTTTCGTTTCATTTCATCACAAATTTT
ATTCATGCATTTTTCACTTGTCATCAACACTGGAATTGCATTAATAAAACAACCTTTCATTTCATACCCCATCCACCCCACCTTCCTTCACGAATCACGATCTAACAATCCACTGGAAATCCTATCAAACCTACGACAAAATTTCATTCCCACCCTCTAAACCCTCTCCCCACC
ATCGCAGCCCTAGAAAACAAGATACTTAACTACTAAAACCACTCTAATAGTTTACCAAAAACATTGGTCTTGTAAACCAAAGAATGAAGACTACACCTCTTCTTAGAGTTCCCAATTACCATCGACACACCTCAGAAAAAAAGGACTTAAACCTCTATCTCCAACTCCCAAAGC
TGGCATTTTACATTAAACTATTCTCTGATCTTCCCCCTAAACTGCCCGAATAGCTCCGCACGACAACCCTCGTACCAATTCCAACACAACAAACAAAGTCAACAATAACCCTCATCCTGCTACTAAAAACATCCCTACCCCATACGAATAAAACATAGCCACGCCGCTAAAAT
CCAACCGAACAGAAAATACCCCTCCACTATCAACCGTAACCACCCCCATCTTCCAACACTCTACTAAACCCCAACCTCCCACAACTGCCCCAACAACAAGCACCAAAATAAACCCCACCCCATATCCCACAACTCCCCAACTCCCTCAAGCTTCAGGATAAGGATCTGCTGCCA
AAGATACAGAGTACACAAAAACTACCAACATCCCACCCAAATACACTATAAATAGTACTAACGACACAAAAGAAACTCCTAGACTTAACAACCACCCACACCCTACAACAGATGCCAGTACCAAACCAACCACCCCATAATAAGGTGAAGGATTAGACGCAACTGCCAGCCCCC
CTAAAACAAAGCATAACCCCAAAAAAAGTACAAAATAAGCCATAGCGGTTCCCGCTTGGCTTTTCTCCAAGATCTGCGGCCTGAAAAACCGCCGTTGAACTTCAACCACGAGAACACAAAATAATTTCAAACGAACCCCTCTAGAAGACTCCCCCCCCCTCCCCCCCCAAAGCA
GCTTATGTNTNAATGNGCATTACTCTATTTACCACATAATACATTACATTAATGTAGGAAATACATTTAATGCATGTACTACGTACATGACCATGTAGACGGGCATACCCCTTTTACCCACTCACGAACCCCCAGAGGACAAATACTTCAATAGTCCCTACCACATACCACTCA
AACGGATTAAACCCATAAATTCCAAGTTCTGTGTATATCTCTGTCCAGAATACGGCAGTGCTCGAATAACATAACCTGAATGGTANCAGAACATAACATGCCATCCCTTCTCGTAGGACCGGTAGCTGTCGGACCAGGTTATTTATTAATCGTTCTTCTCACGAGAAATCAGCA
ACCCGCTGCATATAAGATACTGCGTTACTAGCTTCAGGACCATTCTTTCCCCCTACACCCCTCGCACGACTTGCACTTTTGCGCCTCTGGTTCCTCTGTCAGGGCCATAACTTGGTTAGTCCCCTCTCCTCACTTTTTACGAAGTCATCTGGTTGGCTATATATCACCATTTA
GTCCGTGATCGCGGCATTTCCTCTTCTTAGCACTTTTGGTTCCCTTTTTTTTTGGGGCGTCTTCAGGCTGCCCTCCCAGCGCAACGGGTGCTTACAATCTAAGACCTGAGCATACAATGTGTTGCGGTCTGATCTTGGCCCTCAGGAATCCCTGAATGAGACGGTTGAAGTATA
TGGGGAATCATCTTGACACTGATGCACTTTGTTTTCCATTTGGTTATGGTGTATCCACAAACCCTTATTATGTTGTTATTTAGTGAATGCTTGTTAGACATAATTTCTTACTTTTTCTTGCTCTTACATTTCCTCTAACTTTCTAAACAACACTAGTAACTTTTCAACTAAATT
TTTCGTTTCATTTCATCACAAATTTTATTCATGCATTTTTCACTTGTCATCAACACTGGAATTGCATTAATAAAACATGACAACTCATCACTCATCACATATGTGCATAATTCACCATCAACTATTAAAGAAACCTCCCTGCAAAACAAAAACAAAAACAACAAACACAAACAA
ACAAACAAACAACAGATAAATCAAGCAACAAACAACAGATAAATCAAGCAACAAACAACAGATAAATCAAGCAACAAACAACAGATAAATCAAGCAACAAACAACAGATAAATCAAGCAACAAACAACAGATAAATCAAGCAACAAACAACAGATAAATCAAGCAACAAACAAC
AGATAAATCAAGCAACAAACAACAGATAAATCAAGCAACAAACAACAGATAAATCAAGCAACAAACAAAACACAAACAATTAAACAACAAACAACAGATAAATTAAGCAACAAACAACAGATAAATCACT**GTCTTTGTAGCTTAATGATAAAGCATGGCACTGAAGATGCCAAG**
ATGCTGCCACACGCACCCAAAGACAAAAGACTTAGTCCTAACCTTACCGTTAACTCTTGCTAAACATATACATGCAAGTATCCGCGCCCCAGTGTAAATGCCCTTGATTTCTTACCAAGATGAGGAGCGGGCATCAGGCACACCCCTGAACGTAGCCCAAGACGCCTTGCTT
AGCCACACCCCCATGGGTACTCAGCAGTAATTAACCTTAAGCAATAAGCGAAAGCTTGACTTAGTTATAGCAATACCTTAGGGTTGGTAAATCTTGTGCCAGCCACCGCGGTCACACAAGAGACCCAAGTTAACTGTGATACGGCGTAAAGAGTGGTATCATGCTATCATACCA
GCTAAGATCAAAATACAGCTGAGCTGTCATAAGCATAAGATGTACGTAAGATCACCCTCAAAACGATCTTAGCACCTACGATCAATTAGCCGCCACGAAAGCTAGGGCACAAACTGGGATTAGATACCCCACTATGCCTAGCCCTAAATCTTGATACTTACCCCACCAAAGTAT
CCGCCCGAGAACTACGAGCACAAACGCTTAAAACTCTAAGGACTTGGCGGTGTTCCAAATCCACCTAGAGGAGCCTGTTCTATAATCGATAACCCACGATGCACCCAACCGTTCCTTGCCAAAACAGCCTACATACCGCCGTCGCCAGCCCACCTCCCCTGAGAGCTCAACAGT
GGACACAATAGCCTACCCCGCTAGCAAGACAGGTCAAGGTATAGCCCATGGAGCGGAAGAAATGGGCTACATTTTCTAAAATAGAAAACTCTACGGAAGGGGGCATGAAACAACCCCTAGAAGGCGGATTTAGCAGTAAAGCGGGATAATATAAGCCTCCTTTAAACCGGCCCT
GGGACACGTACATACCGCCCGTCACCCTCCTCACAAGCTACAAGTCTCTAATAACTAATACACTCATTAGCCAAAGATGAGGTAAGTCGTAACAAGGTAAGTGTACCGGAAGGTGCGCTTAGCATA

Uria aalge (Charadriiformes/Alcidae) mitogenome from ND5 to 12S rRNA (MN356418.1 - re-annotated)

GO-FDr in Figure 8

ATGGAGACCACACTACTCCTCAACACCTTTATAATCCTAACACTAACAATCATTCTATCTCCAATCCTATTTCCTCTCCTACCCCAAAACACTAAAAACTCCCCCACCACTATCATTCGCACAGTTAAAACTGCCTTCTTAACTAGCCTAGTACCAATAATCCTATTTATGTAC
TCAGGTATAGAAAGTATCATCTCTAACTGAGAATGAAAATTTATCATAAACTTCAAAATCCCTATTAGCCTCAAAATAGATCAATACTCACTAATATTCTTTCCCATTGCATTATTCGTAACATGATCCATTCTACAATTCGCATCATGATACATAGCCTCAGAACCATACATC
ACAAAATTCTTCTATTACCTCCTAACATTTCTGATTGCCATACTGACCCTAACCATTGCCAACAACCTATTTCTTCTCTTCATTGGATGAGAAGGCGTCGGCATTATATCATTCCTACTAATCGGCTGATGACAAGGACGAGCAGAAGCCAACACAGCTGCCCTCCAAGCCGTA
CTTTACAACCGAATTGGAGATATTGGTCTTATCCTGAGCATAGCATGACTTGCCTCTACCATAAACACCTGAGAAATCCAACAAGCATTCACTCTATCCCAGACCCCAACACTCCCCCTCCTCGGCCTAATCCTAGCAGCCACAGGAAAATCAGCACAATTCGGCCTCCACCCA
TGACTGCCAGCCGCCATAGAAGGTCCAACTCCAGTCTCCGCTCTACTCCATTCCAGCACCATAGTAGTCGCCGGAATCTTCCTACTTATCCGCACCCACCCTCTACTCTCCAATAACCAAACCGCCCTCACCTTATGCTTATGTTTAGGGGCATTATCCACACTATTCGCCGCC
ACCTGTGCAATTACACAAAACGATATCAAAAAAATCATCGCCTTTTCCACATCCAGCCAACTAGGACTCATAATAGTAACCATTGGACTAAATCTCCCTCAACTTGCCTTCCTACACATTTCCACACATGCCTTCTTCAAAGCTATGTTATTCCTTTGCTCAGGATCAATCATC
CACAGCCTCAACGGAGAACAGGACATTCGAAAAATGGGTGGCTTACAAAAAATACTCCCTACAACTACTTCCTGCCTTACCATCGGCAACCTGGCCCTAATAGGAACCCCCTTCCTGGCAGGATTCTACTCAAAAGACTTAATTATCGAAAGCATAAACACCTCTTACTTAAAC
ACCTGAGCCCTTGCCATAACCCTCCTAGCCACAGCATTCACCGCAACATACACCCTGCGCATAACACTACTAGTCCAAACAGGATTCACCCGAATAACTACAATTACCCCAATAAATGAAAACGACCAAACAATCATCAACCCAATCACACGCCTAGCCTTAGGCAGCATTATA
GCCGGCCTACTCATTACGTCCTTCATCCCTCCAACAAAAACTCCTCCAATAACCATACCAATACTTACAAAAACTGCAGCCATCATCGTCACAGTCCTAGGCATTATCCTAGCACTAGAACTCTCAAACATAACCCAATCCCTAACCCAACCAAAACAAACTCACCTCATAAAC
TTCTCCTCCTCACTAGGGTACTACAACCCACTATCACATCGACTCTCATCTACAAACCTACTAAACAGCGGACAAAAAATCGCCGCCCACCTAATCGACTTATCCTGGTACAAAAAAATAGGCCCAGAAGGAATCGCCGACCTTCAACTAATAGCAACCAAAACCTCAACCACC
CTTCACTCTGGATTAATCAAAACCTACCTCGGCTCATTTGCCCTATCTATCCTCATTATTTTATCATCATATAGACCCACGACCCAATGGCCCCAAACCTCCGAAAATCCCATCCGCTCCTTAAACTCATCAACAACTCCCTAATTGACCTTCCCACCCCATCAAACATTTCTG
CCTGATGAAACTTCGGATCCTTACTAGGTATCTGCCTACTAACACAAATCCTCACAGGACTACTCCTAGCCACGCACTACACTGCAGACACAACACTAGCCTTTTCATCCGTCGCCCACACATGTCGAAACGTACAATATGGCTGACTAATTCGAAACCTCCATGCAAACGGAG
CATCCTTCTTCTTCATCTGCATTTACCTCCACATCGGACGAGGATTCTACTACGGCTCATACCTGAACAAAGAAACCTGAAACACAGGTGTCATCCTCCTACTAGCCCTAATGGCAACCGCTTTCGTAGGATATGTCCTCCCATGAGGACAAATATCATTCTGAGGGGCTACAG
TCATTACTAACCTATTCTCAGCAATCCCCTACATCGGCCAAACCCTCGTGGAATGAGCTTGAGGTGGTTTCTCAGTAGACAACCCCACACTAACCCGATTCTTCGCCCTCCATTTCCTCCTCCCATTCATAATTGCAGGCCTCGCCTTCATTCATCTTACCTTCCTCCATGAAT
CCGGATCAAACAACCCCCTAGGAATCCCATCCAACTGCGATAAAATCCCATTTCACCCTTACTTCTCACTAAAAGACATCCTAGGATTCATTATCATATTCCTTCCACTAACAACCCTAGCCCTATTCTCCCCCAACCTACTAGGCGACCCAGAGAATTTTACCCCCGCAAACC
CACTAGTTACACCTCCCCATATCAAACCAGAATGATACTTCCTATTTGCATACGCTATCCTGCGATCAATCCCCAATAAACTTGGAGGTGTACTCGCCCTAGCAGCTTCCGTACTAGTACTATTCTTGACCCCTCTACTCCACAAATCTAAGCAGCGCGCCATAACCTTTCGCC
CACTCTCACAACTCCTATTCTGAACCCTAGTCGCCAACCTCCTCATTCTAACATGAGTAGGTAGCCAACCCGTAGAACATCCATTCATCATCATTGGACAACTAGCCTCCCTTACCTACTTCACCATTCTACTCCTCCTATTCCCCATCATCGGAGCCCTAGAGAATAAAATAC
TTAACTACTAAAAACACTCTAATAGTTTATAAAAAACATTGGTCTTGTAAACCAAAGACTGAAGACTACACCCCTTCTTAGAGTTTCCACTTACCCTATCTCAGAAAAAGAGGACTTAAACCTCTATCTCCAACTCCCAAAGCTGGTATTTTGTACTAAACTATTCTCTGACCC
TCCCCCTAAACCGCTCGAATGGCCCCACGAGTTAATCCACGTACTAACTCTAATACAACAAACAAAGTCAACAACAACCCTCAACCCGCCGCCAAAAACATNCCAACGCCACAAGAATAAAACATTGCCACCCCACCAAAATCTAACCGAACAGAGAACATACCTACACTGTCA
ACAGTAACTATCCCCAAATCCCAGCACTCAAACCCACCAACAACCACCCCCGCCACAAGTACTGCAACAAAACCCATCCCATACCCAACAACCCGTCAATCCCCTCAAGCTTCAGGAAAAGGATCCGCCGCCAAGGACACAGAATAAACAAAAACTACCAACATTCCCCCTAAG
TACACCATAAACAGTACCAAGGACACAAAGGAAATACCTAAACTCAACAACCACCCGCACCCCGCAACAGAGGCCAACACCAAACCAACCACCCCATAATATGGAGAAGGATTAGATGCAACTGCCAAACCCCCTAACACAAAACATAGACCTAAAAAAAGCACAAAATAAGTC
ATAGCAGTTTCTGCTTGGCTTTTCTCCAAGACCCGCGGCCCGAAAAGCCACTGTTGTACCACTTCAACTACAGAAACTAGAAATTGTACTGGTACCCCCCCCCCCTCCCCCCCCATATAGCAAGCCAAATCGCCATGTGGGAGTATACATTAAACTACCAACCCCATAAGCACA
TACACTCCATGTTCCAAAACCATTAATATACACACAGACATAACCCTGAATTTTTCACATTTCTCCTTCAGAGGACCTCCCAGCCCAATGGATCCGAATTCCATTACAATATCCGTACTAATACCATCTCCTCTCCAGTCTTTACATACCAACTCCGACAAGATACGACAGTGC
CTGTCTACACCTTTGTAATGGGGTAAAGGCATGGCCCTCCAAAACTTCTCGAATACACAAAGCTTCGTGCCAGGTTATTTATTAATCGAACTCCTCACGTGAAATCAGCAACCCGGTGTTTGAAAGATCCTACGCTACCAGCTTCAGGACCATACTTTCCCCCTACACCCCTAG
CCCATCTTGCTCTTTTGCGCCTCTGGTTCCTATGTCAGGGCCATAACTTGGTTAGTCCTCTCAACTTGTACTTCACCGATACATCTGGTCGGCTATATATCACCATCTCACCCGTGATCGCGACATCCGACCGTTTTGGCACTTTTGGTTCCTTTTTTTTTTTTTTACGTCTTCA
ATAAACCCTTCCAGTGCNCCGAGGTACACACAATCTAAGACCTGAGCTTTCCCTGGTATTCGTACGGATTTTGGCCCTCAGGAATACCTGAATGTCAAGGTTTAACGGGTTGGGGGAATCATTTTTACACTGATGCACTTTGCTTTGCACTTGGTTATGGGATCTCCGCAAGCT
CTTATTTATGTTGTTATTTAGTGAATGCTTGTTGGGCATATTTTATTATTTTTCATTTCCTCTAACTTTTTAAACAACACTAGCAAATTTTATTCAAAAACAAACTGTGATTTTCATCACACATTTTGTCATCGTCATCACACATTTTATCATCACACTTATCATCTACAAACG
GCACTGGAATTCCATTAAAAANAGGACTAATTTAATCCGTCACATTTTATCTTACCTTCCTCCATGAATCCGGATCAAACAACCCCCTAGGAATCCCATCCAACTGCGATAAAATCCCATTTCACCCTTACTTCTCACTAAAAGACATCCTAGGATTCATTATCATATTCCTTC
CACTAACAACCCTAGCCCTATTCTCCCCCAACCTACTAGGCGACCCAGAGAATTTTACCCCCGCAAACCCACTAGTTACACCTCCCCATATCAAACCAGAATGATACTTCCTATTTGCATACGCTATCCTGCGATCAATCCCCAATAAACTTGGAGGTGTACTCGCCCTAGCAG
CTTCCGTACTAGTACTATTCTTGACCCCTCTACTCCACAAATCTAAGCAGCGCGCCATAACCTTTCGCCCACTCTCACAACTCCTATTCTGAACCCTAGTCGCCAACCTCCTCATTCTAACATGAGTAGGTAGCCAACCCGTAGAACATCCATTCATCATCATTGGACAACTAG
CCTCCCTTACCTACTTCACCATTCTACTCCTCCTATTCCCCATCATCGGAGCCCTAGAGAATAAAATACTTAACTACTAAAAACACTCTAATAGTTTATAAAAAACATTGGTCTTGTAAACCAAAGACTGAAGACTACACCCCTTCTTAGAGTTTCCACTTACCCTATCTCAGA
AAAAGAGGACTTAAACCTCTATCTCCAACTCCCAAAGCTGGTATTTTGTACTAAACTATTCTCTGACCCTCCCCCTAAACCGCTCGAATGGCCCCACGAGTTAATCCACGTACTAACTCTAATACAACAAACAAAGTCAACAACAACCCTCAACCCGCCGCCAAAAACATTCCA
ACGCCACAAGAATAAAACATTGCCACCCCACCAAAATCTAACCGAACAGAGAACATACCTACACTGTCAACAGTAACTATCCCCAAATCCCAGCACTCAAACCCACCAACAACCACCCCCGCCACAAGTACTGCAACAAAACCCATCCCATACCCAACAACCCGTCAATCCCCT
CAAGCTTCAGGAAAAGGATCCGCCGCCAAGGACACAGAATAAACAAAAACTACCAACATTCCCCCTAAGTACACCATAAACAGTACCAAGGACACAAAGGAAATACCTAAACTCAACAACCACCCGCACCCCGCAACAGAGGCCAACACCAAACCAACCACCCCATAATATGGA
GAAGGATTAGATGCAACTGCCAAACCCCCTAACACAAAACATAGACCTAAAAAAAGCACAAAATAAGTCATAGCAGTTTCTGCTTGGCTTTTCTCCAAGACCCGCGGCCCGAAAAGCCACTGTTGTACCACTTCAACTACAGAAACTAGAAATTGTACTGGTACCCCCCCCCTCC
CCCCCATGAAAAAATGCACGTTGACGTAAATATTCTCATGTCCTTCATCGCATACACTTATTATCCTCAAACAACAAACAACAAACAACAAACAACAAACAACAAACAACAAACAACAAACAACAAACAACAAACAACAAACAACAAACAACAAACAACAAACAACAAACT**GTC**
TTTGTAGCTTACCGATTAAAGCATGGCACTGAAGATGCCAAGATGGCTGCCATTCATGCACCCGAAGACAAAAGACTTAGTCCTAACCTTACAGTTAATTCTTGCTAAACATATACATGCAAGTATCTGCGCTCCAGTGTAAATGCCCTTAGTCCTTTGCTTAAGGAAAAAGGA
GCGGGTATCAGGCACACTCATAGCTGTAGCCCAAGACGCCTTGCTCAGCCACACCCCCACGGGTACTCAGCAGTAGTTAACATTAAGCAATAAGTGCAAGCTTGACTTAGTTATAGCAATTTCAGGGTTGGTAAATCTTGTGCCAGCCACCGCGGTCACACAAGAGACCCAAAT
TAACAGTATGTCGGCGTAAAGAGTGGTACTATGATATCATTGCAACTAGGATTGAAATGCAGCTAAGCTGTCATAAGCCCAAGACGCACTTAAAGTCACCTTCAAGATGATCCTAGCGCCTCTGATTAATTTAACCCCACGAAAGCTAGGGCACAAACTGGGATTAGATACCCC
ATTATGCCTAGCCCTAAATCTTGATGCTTACCACACTAAAGCATCCGCCGGAGAACTACGAGCACAAACGCTTAAAACTCTAAGGACTTGGCGGTGCCCTAAACCCACCTAGAGGAGCCTGTTCTATAATCGATAATCCACGATTTACCCAACCACCTCTCGCCAAAGCAGCCT
ACATACCGCCGTCGCCAGCCCACCTCCTCTGAGAGCCCTACAGTGGGCATAATAGCCCAACCCGCTAATAAGACAGGTCAAGGTATAGCCTATGGGGTGGAAGAAATGGGCTACATTTTCTATAATAGAAAACCCACGAAAGAGGACATGAAACAGTCCACGGAAGGCGGATTT
AGCAGTAAAGTGGGACAATAGAGCCCCCTTTAAGTCGGCCCTGGGGCACGTACATACCGCCCGTCACCCTCCTCACAAGCCACTAACTCTCATAATTAATAAACCCTATGGCTGAAGATGAGGTAAGTCGTAACAAGGTAAGTGTACCGGAAGGTGCGCTTAGCATA

Urocolius indicus (Coliiformes/Coliidae) mitogenome from ND5 to 12S rRNA (MN356373.1 - re-annotated)

GO-NH in Figure 8

GTGGAACCAGCCCTACTCATCAACACCTCTATACTCCTCACAATAACCATTATTTTAATACCCCTAATCCTTCCCCTAGCATCAACAACCATAAAAAACTCACCAACCCTAATAGTACACTCCACCAAAACAGCATTCATTACCAGCCTACTCCCCATATACACATTCATATAC
ACAAATACAGAAAGCATCACCTCCCACTGAGAATGAAAATTTATTATAAACTTCAAAATCCCACTCAGCTTCAAATTTGACCAATACTCCATAACATTCTTCCCCATCGCCCTGTTTGTGACATGATCAATCTTACAATTTGCAACATGATACATACACTCAGAACCACTAATT
ACAAAATTTTTCTCCTACCTCTCTATATTCTTGATTGCCATACTCACACTCACAGCAGCCAACAATATGTTCCTACTATTTATCGGCTGAGAGGGGGTAGGAATCATATCATTCCTACTAATCGGCTGATGATACGGACGAACAGATGCTAACACAGCTGCCCTTCAAGCCGTA
TTGTACAACCGAATTGGAGACATCGGCCTAATCCTAAGCATAGCATGACTCGCCATAACCACTAACACCTGAGAAATGCAAACTCATCCAGTCCAAGTCCAAACACTCCCCCTACTCGGATTAATCCTAGCCGCCACAGGAAAGTCAGCCCAATTTGGATTACACCCTTGGCTT
CCAGCAGCCATAGAAGGCCCAACACCAGTCTCCGCTTTACTCCACTCTAGTACAATAGTTGTAGCCGGAATTTTTCTACTAATTCGTACCCACTACATACTAGAAACTAACCACTTCGCCCTGACCCTATGTTTATGCCTAGGAGCCCTCTCCACTTTATTCGCCGCTACCTGT
GCACTTACCCAAAACGATATCAAAAAAATCATCGCCTTCTCCACCTCCAGCCAACTAGGACTAATAATAGTAACAATCGGACTAAACCTGCCCCAACTGGCCTTCCTTCACATTTCAACACACGCCTTTTTCAAAGCCATATTATTCCTCTGCTCCGGATCTATCATCCATAAC
CTCAATGGTGAGCAAGATATTCGAAAAATAGGAAACCTCCAAAAAATACTACCAACAACTACCTCCTGCCTAACTATTGGTAGCCTAGCCCTCATAGGCACACCATTCTTAGCTGGATTCTATTCAAAAGACACAATCATCGAAAACCTTAACACCTCTTACCTAAACACCTGA
GCACTACTTCTCACACTGCTAGCCACATCATTCACTGCAACCTATAGCACACGAATAGCTACACTTGTACAAACAGGACAAACCCGCATCAACACTACATTGGCAATAAACGAGAATAACCAAGCAATTACACAACCCCTCACTCGCCTTGCACTTGGAAGCATTACAGCAGGA
TTACTCATCACCTCATATATTACCCCTGCCAACACTACCCCAATAACAATACCCCCTATCACCAAAATAGCTGCAATAATCGTCACTATCCTAGGCCTCATACTAGCCCTAGAACTCTCAAATATAACACATAACCTCTCTCACCCTAAACAAACCCCACATCAAAACTTTTCC
TCCTCCCTAGGCTACTTCAACCTACTTATACACCGCCCTAACACTACCAAACTCTTAAGCAACTGTCAAAAAATTGCAACCCATCTCATTGATCTCTCCTGATACAAAAAAATAGGTCCCGAAGGTATCACTGACCTTCAACTAATCTTAACCAAAACATCAACAGCCCTACAC
ACCGGACTAATCAAAACCTACCTAACATCATTTGCACTATCAATCCTAATCATCCTGTCCACACAATAAATTAATGGCCCCAAACCTACGAAAATCCCACCCGCTACTAAAAATAATTAATAACTCCTTAATCGACCTACCTACGCCTCCCAACATTTCAGCCTGATGAAACTT
TGGATCACTCCTAGGCATCTGCCTCATAACTCAAATCCTAACAGGCCTATTACTAGCCGCCCACTACACCGCAGATACAAGCCTAGCCTTCTCATCCGTCGCTCATACCTGCCGGAACGTCCAATATGGCTGACTAATCCGCAACCTTCACGCAAACGGAGCATCTCTATTCTT
TATTTGCATCTACCTTCACATCGGACGAGGGTTCTACTACGGCTCATACCTTTACAAAGAAACCTGAAACACAGGCATCATCCTCCTCCTCACCCTAATAGCAACAGCCTTCGTAGGCTATGTCCTACCATGAGGACAAATATCCTTCTGAGGCGCTACCGTCATCACCAACTT
ATTCTCAGCCGTCCCCTACATTGGACAAACCTTAGTAGAATGAGCCTGAGGGGGATTCTCGGTAGATAACCCAACTCTAACCCGATTCTTCACCCTCCACTTCCTACTCCCCTTCATAATTGCCGGACTCACATTAATCCACCTAACCTTCCTTCACGAATCAGGCTCAAACAA
CCCCCTAGGCATCATCTCAAGCTGCGACAAAATCCCATTCCACCCCTACTTCTCTCTAAAAGACATCCTAGGATTCGCACTCATAATCTTCCCCCTAACATCTCTAGCCCTATTTTCCCCAAACCTCCTAGGAGACCCAGAAAACTTCACACCAGCAAACCCCTTAGTAACCCC
TCCCCACATTAAACCAGAGTGGTATTTCCTATTCGCATACGCCATCCTACGCTCCATCCCAAACAAATTGGGAGGAGTACTAGCCTTAGCTGCATCAGTCCTCGTACTATTCCTTTGTCCACTACTCCACAAATCCAAACAACGCACAATAACCTTCCGACCCCTATCCCAACT
ACTATTTTGAACCCTCACCGCAAACCTATTTATCCTAACATGGGTAGGCAGCCAACCAGTAGAACACCCCTTTATCATCATTGGCCAACTAGCCTCAATCACCTACTTCACAATCATCCTTATCCTCTTTCCTATCATCGGAACACTAGAAAACAAAATACTCAAATACTAAAA
TACTCTAATAGTTTATTAAAACATTGGTCTTGTAAACCAAAGAATGAAGACTTATACCTCTTCTTAGAGTTCC***CTATAATAACCAAACCAAATAAAACCTTTCCCCTAACCCATGGCCCTCCCCCTAGCAAAGGAAGGGCCATTATTGCCTTACCTTTTTCCTCCCTAACCCAT***
GGCCCTCCCCCTAGCAAAGGAAGGGTCATTATTGCCTTACCCTTCCCCCACCCCCACTCATGGTCCACAACAAAATTTCTCAATCTGGAAAGAAAAAAGACCCCATACTACCACTATTATTATCTCCCCCCCCCCCCTACCCCCCCCTATGTACTTCTGTACATTAAACCCAT
AACCCCATATCAATGCACCTCTCTAGGCGTAAATCCAAATGATTGAACTGATCCCATAACATGAAAACGGATACGAACTCAAGACAATCCATTTTTGCCGGCAGACTAACAAATCCATGCACACACGGTTTAACAATCTATATAACTTCGGACTAAACCCATAAAATGTCTCTT
TATACATCCCTAATTTCAAGCATACGGAAGTGCTTTAACACATACCATCAATGGTAACCGGACAAATCAATGTAATCTGATTTCTCGTCGGACCTATTTCTAAAGGACTGGGTTATTTATTGATCGCGCCCCTCACGTGAAATCAGCAACTCGACGTATTACGGATCCTACGTT
ACTAGCGTCAGGCCCATTCTTTCCCCCTAAACCCTCGCCCAACTTGCGCTTTTGCGCCTCTGGTTCCTATTTCAGGGCCATTCATAAGCTTGACTCCCCCCAACTTGTTATTCACAGATACATCTGGTTACTTATATCTCAACCATTTGGCCTCTTTATCGCGTCACTGGCCGG
TTCTCTACTTTTGGTTCTCTTTTTTTTCTGGGTAATTTCAACGGACCCTCCAGGCGGCTGGAGCCGGAGCATACTATCGCGTGACGTGGGCACCGGTGGACTTCGTCCAATTCCTCTCTTTCCTTCGGTTTAACTCAATGAGACGGAGTCAAGTGTCCTCGGAATCATTTTTAC
ACTGATGCACTTTGTCATGCATTTCTCATGCCTGTCTTGCTTACTTAAACTGTCAGGGGTAATTAAAGCAATGCTCGAAAGACATAAATTTTCACACTTTCAATACTCCTAACTTTCCTGCTATTTAAAAAATTTTAGCAGGAATTTTCCCAAGTCTGACACCAAAATTTTTGT
CATTTTTTGTCATTTTTTGTCATCAAATCAACACTTGTTTGCACTTGTTGTCACGGCCTGATTTCCCCTTAAACAACAAAATCGTTTCACAAAAATTTTTTCAAATCATTAACACAAATTTTACACATACATTCCTCTGACTTTTCCCATAAAATTCTTTCATCATTCACCATT
CATTTTTTTTCACCAAAATTAACTAACAACAGTAGCCAAAAACCCCGTAAACCTTCTAAGAAGGGGACCCCCCCCCCATTTGTTCAACCAACCAAACAAAAACACCCACCTAATCCCCTACCACTCTCAGAAAAAGAGGGATCAAACCTCTGCCTCCAGCTCCCAAAGCTGATG
CTCTGCATTAAACTATTTTCTGCCCCCCTAAACCGCCCGAATAGCACCACGAGATAACCCTCGAACCAACTCCAACACTACAAACAATGTTAACAATAACCCCCACCCCGCCACCAAGAACAACCCCACCCCCCACGAATAAAACATAGCTACTCCACTAAAATCTAAACGCAC
AAAAAATCCCCCACCACTATCCACAACATCAACCCCAAACCCCAAACCACCAAACCCTCCAACCAAAATACCAACAATAAGCACCAACAACAGCCCCAACCCATAACCAACTACCCGTCAATCCCCTCAAGTCTCAGGAAAAGGGTCCGCCGCCAAAGCAACAGAATATACAAA
AACCACCAACATCCCCCCTAAGTACACCATAAATAGTACCAAAGACACAAAAGACAACCCCAAACTCATCAACCACCCACACCCAACAACAGACGCCAACACCAACCCAACAACTCCATAATATGGAGAAGGATTAGATGCAACCGCCAACCCCCCTAAAACAAAACACACCCC
TAAAAAAAATATAAAATATACCATAATAGTTTCTGCTTGGCCTCTCTCCAAAACCTACGACCTGAAAAGCCGCCGTTGTACTCAATTCAACTACAGAAACATCCTACCCTAAAACCAACCCTCAACCATTTTCACAAAACCAATCACAAACCATTTATCCTGCCAAATTAACCC
CCAAGCCCTATTATAAATGACCAAATAAACAATCCCACACACTTCCCCACCTAAACTACTATACACCAACCACTTAAATCCAAACCCACTCAAACAACACACAACACAAAACTACAAAGCCACTTAACCAACCAAGACCCCATATAAGTTTACTTACAAACAACCCAACAAACG
ACAACACAAATTAGACTATTCAAGCACAAAGTTTTCCCCT**GTCTTTGTAGCTTAAACAAAGCATAACACTGAAGATGTTAAGATGTTGTTAACTACACCCAAAGAC**AAAAGACTTAGTCCTAACCTTGCAGTTAGTTCTTACCTAGTATATACATGCAAGTATCCGCGCCCCAG
TGTAAATGCCCTAACCTCTTTACTAAAGATATAGGAGCTGGTATCAGGCACACCCACCATAGTAGCCCACAACACCTTGCTTCGCCACACCCACACGGGCTTTCAGCAGTAATTGACATTAAGCAATAAGTGTAAACTTGACTTAGCTACGGTAAAACCCAGGGTCGGTAAATC
TTGTGCCAGCCACCGCGGTCATACAAGAGACCCAAATTAACTGCACCCGGCGTAAAGAGTGGTACAATGATATCATAACAACTAAGACTGAAACACAACCAAGCCGTCATAAGCTAAAGTTGAACTTAAAACCATCCTAAAAATGGTCCTAGTATATTAGATTAACTTAACACC
ACGAAAGCTAAGACACAAACTGGGATTAGATACCCCACTATGCCTAGCCATAAATCCAGATTCTCCCCTCACTAGAGAATCCGCCCGAGAACTACGAGCACAAACGCTTAAAACTCTAAGGACTTGGCGGTGCCCCAAACCCACCTAGAGGAGCCTGTTCTGTAATCGATAACC
CACGATCCACCTAACCACTTCTTGCCCAAACAGCCTACATACCGCCGTCCCCAGCTCGCCTTAACTGAAAGCACAATAGCAAGCATAACAGCCACAACCCACGCTAGTAAGACAGGTCAAGGTATAGCCTATGAAGTGGAAGAAATGGGCTACATTTTCTAACCATAGAACACT
CACGAAAAGAGGAGTGAAACCCTCCCCTAGAAGGCGGATTTAGCAGTAAAGAAAGGTAATTAAACTTCCTTTAAGACGGCCCTGGAGCACGTACATACCGCCCGTCACCCTCCTCACGCACTACCCATCAACAATACATAATACAAGTACCAACCACAAGACGAGGTAAGTCGT
AACAAGGTAAGTGTACTGGAAAGTGCACTTAGCACA

Figure S10. Sequences of mitogenomic fragments in Neoaves and Galloanserae mitogenomes amplified and sequenced for: *Alca torda* (MK263222), *Apus apus* (MW151827), *Cathartes aura* (MN629891), *Chrysolophus pictus* (MW151829), *Ciconia nigra* (MH264509), *Corythaixoides personatus* (MW082596), *Gavia arctica* (MK263210), *Gavia stellata* (MK263209), *Eurypyga helias* (MW208859), *Podiceps cristatus* (MN629890), *Podiceps grisegena* (MK263194), *Scopus umbretta* (MW151828), *Spheniscus demersus* (MH264510) and *Uria aalge* (MK263188). Genes for NADH dehydrogenase subunit 5 are marked in magenta coloring, cytochrome b in red coloring, tRNA-Thr in green coloring, tRNA-Pro in yellow coloring, NADH dehydrogenase subunit 6 in cyan coloring, tRNA-Glu in grey shading, 12S rRNA in blue fonts, **tRNA-Phe in bold fonts**, and control regions in red fonts. *Pseudogenes are in italics and underlined*.

Alca torda (Charadriiformes/Alcidae) mitogenome from CR1 to CR2 (MK263222)

GO-FD in Figure 8

GTTCCTATGTCAGGGCCATAACTTGGTTAGTCCTCTCAACTTGTACTTCACTGAGACAACCGGTCGGCTATATATCATCATCTCACCCGTGATCGCACATCCGACCGTTTTGGCACTTTTGGTTCCTTTTTTTTTTCTCTAACCTTCAATAAGCCCTTCCAGTGCACCGAGGTG
TACACAATCTATTGACGTGAGCATCTCATGGTATTCGTCCGGATCTTGGCCCTCAGGAGTACCTGAATGTCAAGGTTGAAGGATTGGGGGAATCATTTTTACACTGATGCACTTTGTCTTGCACCTGGTTATGGAGTGTCCGCAGACTCTTATTCATGTTGCTATTTAGTGAAT
GCTTGTTGGGCATAATTTTTCAATTTTCATTTCCTCTAACTTTCTAAACAACACTAGTAGATTTTACCTAAAAATTGACCATGATCAAATCACGATTTTTGTCACGTATTTTATCATCATCTTTATCACTTACAAATAGCAGTAAAGTTCCATTAATAAAACCACCAATAATCC
GTGACATTTTATCTTCATCTTTCTTCATGAATCTGGATCAAACAATCCTCTAGGAATCCTATCAAACTGTGACAAAATCCCATTCCATCCCTACTTCTCATTAAAAGATATCCTAGGATTCATTATCATATTCCTTCCACTAACAACCCTAGCCTTATTTTCACCCAACCTGTT
AGGCGACCCAGAAAACTTCACCCCCGCAAACCCATTAGTCACTCCTCCTCATATCAAACCAGAATGATACTTCCTATTTGCATACGCTATTCTACGCTCAATCCCCAACAAACTAGGAGGAGTACTAGCCCTAGCAGCCTCTGTACTAGTACTATTCCTGGCCCCTCTTCTCCA
CAAATCCAAACAACGTGCAATAACCTTCCGTCCATTCTCACAATTCCTATTCTGAACCCTAGCCGCCAACCTCTTCATTCTAACATGAGTAGGTAGCCAACCCGTGGAACACCCATTCATCATCATTGGACAACTAGCTTCCCTCACCTACTTCACCATCTTACTCCTTCTATT
CCCCATCGCTGGAGCTCTAGAAAACAAAATACTTAACTACTAAAACACTCTAATAGTTTATAAAAAACATTGGTCTTGTAAGCCAAAGATTGAAGGCTATACCCCTTCTTAGAGTTTCCATTGCCTCAGAAAAAGAGGACTTAAACCTCTATCTCCAACTCCCAAAGCTGGTGT
TTTACACTAAACTACTTTCTGGTCCCCCTCCCCCTAAACCGCTCGAATAGCTCCACGAGTTAGTCCACGTACTAACTCCAGCACAACAAACAAAGTCAGCAACAGCCCCCAACCCGCTACCAAAAACATTCCAACACCACAAGAATAAAACATTGCTACCCCACCAAAATCTA
ACCGAACAGAAAACATACCGACACTATCAACAGTAACCACCCCAAAATCCCAACATTCAACAAACCCACCAATAACCATCCCCATCATCAATACTCCAACAAAACCTGCCCCATACCCAATAACCCGTCAATCCCCCAAGCCTCAGGAAAAGGATCTGCCGCCAAAGACACAG
AATAAACAAAAACTACCAACATCCCTCCCAAGTATACTATAAACAACACCAGGGACACAAAAGAAATACCCAAACTTAACAACCACCCACACCCCGCAACAGAAGCCAGCACCAAACCTACCACCCCATAATATGGTGAGGGATTAGATGCAACTGCCAAACCCCCCAACACAA
AACATAAACCTAAGAAAAACATAAAATAAGTCATAGCAATTTCTGCTTGGCTTTTCTCCAAGACCAGTGGCCTGAAAAGCCACTGTTGTCAACTTCAACTACAGAAACTGCACGCCCCCCCCCCCCCCCCCATGCAAGTGCTCTCTACGCATGTGGGCTTGTACATTACACTACC
ATCCCCATAAATACATACAATCCATGTTCCAAAACCATTAATATACACACAGGCATAACCCTGAATTTTCCACACTTCCCCTTCAGAGGACTCTCAAGCCCAATGGGAGCAGAAATCCATTACAATATACGTACTAATACCATTTCCTACCTAGCTTATACATACCAACCCCAC
AAGATACGACAGTGCTTGGGTACCTCCTTGCCTGGTGTAGGAGCATGACCCTCCAAAATTTCTTGAGTGCGCAAAGCTCGTACCAGGTTATTTATTAATCGAGCTCCTCACGTGAAATCAGCAACCCGGTGTCTGGAAGATCCTACGCTACCAGCTTCAGGAC

Apus apus (Caprimulgiformes/Apodidae) mitogenome from CR1 to CR2 (MW151827)

GO-FD in Figure 8

CATGAACATTCTATGACATGCGTCCTGTTTTTTGGCCTTCAGGAGTAGCTGAATGAGACGGTTTGCGTATAACGGGAATCATTTTGACACTGATGCACTTGCTACCGCATTTGGTTATGGAGTATCCACTAACTACTTATATATGTTGCTTTTTAGTTCATGCTTGTTAGACAT
AATTTTCCAGCTATTTTACATTCCTCTAAATTCCCTTAATATTGCAAATCGACACCAGTAACTTTTCTACTAATCTTCCCAAACACACCATCATCATAATTTTTTCATTTTTTCAATTTTTTTCATCACACCATCACTTAACATTACCACTGAAATTACATTAATAATCTAATT
AACATTTTTCAACATTTATCATGTTAATTTTTTCATAAAAACACCATAATTTACTAAAGCAACCCTACTAGAACAAATTTATACATCATCCATCACCAAAAAACCACATCACATTTAATGACTTACCAGCAAAACACCTAATCATCATCGGCCAACTAGCCTCCCTCACCTACT
TCACTATTCTCCTAATCCTCTTCCCCCTTATCGGAGCTCTAGAAAACAAAATACTTAACTACTAAAATACTCTAATAGTTTATTAAAACATTGGTCTTGTAAGCCAAAGAATGAAGACTCCACCCCTTCTTAGAGTTACCCCAACCCAAATATCAGAAAAAAAGGACTTAAACC
TCTATCTCCAACTCCCAAAGCTGGCATTTTCATTAAACTATTCTCTGACACCCCCCTAACTGCCCGAATTGCACCACGAGAAAGTCCCCGCACAAGCTCCAATACAACAAACAGAGTCAACAGTAAACCCCACCCACCCATCAAAAACATCCCCACCCCCCAAGAATAGAACAT
AGCCACACCACCAAAATCCAACCGAACAGAAAGCATCCCCCCACTATCAACTGCTACCACCCCTCACTCCCACCCCTTAACAAAGCCACCCACCACTACCCCCAAAATCAGCACTAAGGCAAACCCAACCCCATACCCTATAACACGCCAATCTCCCCATGCCTCAGGAAAAGG
ATCAGCTGCCAACGACACAGAGTACACAAAAACTACTAACATCCCCCCCAAATACACCATAAACAAAACCAACGACACAAAAGAAAGGCCAAGACTTACCAATCACCCACATCCAACAACAGAAGCAACTACCAAACCAACCACCCCATAATATGGTGAAGGATTAGATGCAAC
TGCTAATCCCCCTAACACAAAACACACCGCTAAAAAAACAATGAAATAGGTCATAAAAGTTCCTACTTGGCTTTTCTCCAAGACCTACGGCCTGAAAAGCCGTCGTTGTTACTTCAACCATAGAAACACAGCCTACACAAACACCATTAGCCCTATGACGTATGCCCCCTACC
CCCCATAATACAGGGATGTTCCTAGAATCATTATGAGTTCTATTGGCTTTATGTCATACTAGCATTCATCTATATACCCCATTACATTAAATGATACCTAGGGCATACACCTTAATACCCGTACTAAAACCATAAGCTGTTAACCAGGGCAGGCAAAAACACTCTACGAATATG
ACACCACAGGGATTAAGAATGTAATGCTCTACCACCATAACCTAAAACCCTCGTACTAAAACCATTAGAACCCTCGGTTATGCATAAACCTATCACCCCTACGAGAGAGTATCTCAGGTACCAAAGGAATCCATGTTAGGCAGACTGAACTTGCCACTATCTCTTGAAGTGCCG
GTAGCTGTCGTATTGGGTTATTTATTGATCGTACCTCTCACGCGAGACAGCTACGCACCGTAGGTTCCTAATTACGTGACCAGCTTCAGGCCCATTTACTGCCCCTATAGCTTAATTATACACACCGGTGAGCCTTGCTCTTTTGCGCCTCGGGCTGTTTCTTCATGAGCATAG
CATGGCTCCTCCCTTTCCTTTTCTCTTCACGAGACCTTTGGTTCGTTATATTCGTTCTTCTCACCCGTAATCGCGGCATCTGTATTGCT

Cathartes aura (Cathartiformes/Cathartidae) mitogenome from ND5 to 12S rRNA (MN629891)

GO-FD in Figure 8

CGAATCGGGGACATTGGCCTCATCCTAAGCATGGCATGACTTGCCACAACCATAAACACATGAGAAATTCAACAAGCCTTCTCCCCCACCCAAACTCCAACTCTCCCTCTACTCGGCCTCATCTTAGCTGCCACAGGAAAATCAGCCCAATTTGGCCTCCACCCATGACTACCA
GCTGCCATAGAAGGCCCAACCCCAGTTTCCGCCTTACTCCACTCCAGCACAATAGTTGTTGCTGGAATTTTCCTACTCATCCGCACTCACCCCATACTCTCCAACAACCAAACTGCCCTCACCTCCTGTTTATGCTTAGGAGCTCTATCCACACTATTCGCTGCCACATGTGCA
CTCACACAAAATGACATCAAAAAAATCATTGCCTTCTCAACCTCTAGCCAACTAGGGCTAATAATAGTCACCATCGGACTAAACCTCCCACAATTAGCCTTCCTTCACATCTCAATACATGCATTCTTCAAAGCCATATTATTCCTCTGCTCAGGCTCAATCATCCACAATCTC
AACGGAGAGCAAGACATTCGAAAAATAGGGGGACTACAAAAACACTCCCAACAACAACCTCATGCCTAACCATCGGCAATCTAGCCCTAACAGGAACCCCATTCCTAGCGGGCTTCTACTCGAAAGACCTCATCATTGAAAGCCTAAATACCTCTTACCTAAACACCTGAGCA
CTACTGCTAACACTCCTAGCCACCTCATTCACTGCAACCTACAGCCTGCGTATAACCCTAATAGTCCAAACAGGATTCACCCGCACCCCAACAATCACCCCCATAAACGAAAACAATCCAACGATCATCAACCCAATCATCCGCCTTGCTCTAGGTAGCATCCTAGCTGGCCTA
TTCATCACATCATACACTATCCCCACAAAAACACCCCCCATAACCATGCCCACACTCACAAAAACTGCAGCAATCATCGCCACAATCCTAGGAGCGATCCTAGCCTTAGAGCTCTTAAACATAACACACACTCTAACCCAACCAAAACAAAACCCCCTCCTAAACTTCTCATCC
ACATTAGGATACTTCAACCCATTAACACACCGCCTTACCTCCACAAAACTTCTAAACAACGGACAAAAAATTGCCTCACACCTAATTGACCTATCTTGGTACAAAAAAGTAGGCCCTGAGGGACTCGCTGACCTACAACTCCTAGCAACCAAAACCTCAACTACCCTCCACACC
GGATTAATCAAAACCTACCTTGGATCCTTCGCCCTATCCATCCTCATTATCCTATCAACACACAGAACCAAGACTAATGGCCCCTAACATCCGAAAATCACACCCCCTACTAAAAGTAATCAACAACTCCCTGATCGACTTACCCACTCCCTCAAACATCTCTGCCTGATGAAA
CTTCGGATCCCTACTAGGCATCTGCCTAGTGACCCAAATCCTAACCGGCTTACTACTAGCCATACATTATACTGCAGACACAACACTAGCCTTTTCGTCCGTCGCTCACACATGTCGAAACGTACAATATGGCTGACTAATCCGCAACCTACATGCAAACGGAGCTTCATTCTT
CTTCATCTGTATTTACCTACATATCGGACGAGGACTCTACTATGGCTCATACCTGTACAAAGAAACATGAAACACAGGTGTCATCCTCCTACTCACCCTCATAGCAACTGCTTTCGTGGGCTACGTCCTACCATGAGGACAAATATCATTCTGGGGTGCCACAGTAATTACTAA
CCTATTCTCAGCTATCCCTTACATCGGCCAAACCCTTGTAGAATGAGCATGAGGGGGATTTTCAGTAGACAACCCCACTCTGACCCGATTCTTTGCCTTACACTTCCTCCTCCCCTTCGCAATTGCAGGCCTTACCTTAATCCACCTCACCTTCCTACACGAATCAGGTTCAAA
CAACCCTTTGGGCATCGTATCAAGCTGTGACAAAATCCCATTCCATCCCTACTTCACCCTAAAAGATGTCCTAGGATTCATGCTCATATTTCTCCCCCTAACAACCCTAGCCCTATTCTCACCTAATTTATTAGGAGACCCAGAAAACTTCACACCCGCAAACCCTCTAGTCAC
ACCACCCCATATCAAGCCAGAGTGATATTTCCTGTTCGCCTATGCCATTCTCCGCTCAATCCCCAACAAACTAGGAGGAGTACTAGCCCTAGCTGCCTCCGTACTAATCCTATTCCTAATACCTTTCCTCCATAAATCTAAACAACGTACAATAACCTTTCGCCCACTCTCCCA
ACTCCTATTCTGAACACTAGCAGCCAACCTTCTCATCCTAACATGAGTGGGCAGCCAACCAGTAGAACATCCCTTCATCATCATCGGCCAACTAGCCTCCCTCACCTACTTCACCATCCTCCTTATCCTCTTCCCTATCCTCGGAGCCCTAGAAAACAAAATACTCCATCAAAA
ATACTCTAATAGTTTATAAAAACATTGGCCTTGTAAGCCAAAGAATGAAGACTACGCCCCTTCTTAGAGTTTAAACCACACCAACCCTCCCACCAAAACACCTCAGAAAAAGAGGACTTAAACCTCTATCCCCAGCTCCCAAAGCTGGTATTTTACACTAAACTATTCTCTGAC
GTCCACCCTAAACCGCCCGAATTGCCCCACGAGACAACCCCCGCACAAGCTCTAACACAACAAACAAAGTCAACAACAACCCCCACCCAGCCACTAAAAATATTCCGGCCCCGCGCGAATAAAATATTGCTGCACCACTAAAATCCAACCGAATTGAAAACAACCCCACACTAT
CAACAGTGACTACTCCCCACTCCCAACCCCCTACAACTCCTACAACCCCCACACCCACAACAAGTACTAAAACCAGCCCCGCCCCATATCCTACTACACGCCGATCCCCCCAAGCTTCCGGAAAAGGATCGGCCGCCAAAGACACAGAGTACACAAATACCACCAACATTCCGC
CCAAATAGACCATAAATAGTACCAAAGATACAAAAGAAACCCCCAAACTCAACAACCACCCGCAACCCATGACAGATGCCAGTACTAAGCCAACCACCCCATAATATGGAGAAGGGTTGGATGCTACTGCCAGCCCCCCTAAAACAAAACATACCCCTAGAAAAAGCACAAAAT
AAGTCATCAGCAGTTTCTGCTTGGCTTTTCTCCAAGGTCTATGGCTTGAAAAGCCATCGTTGAAACTATCTCAACCACAGAAACACTGGAAATACATTAAAAAAGGCTACCCCCCCTACCCCCCCATAATAAATTAATCTACCTATGTATTACTGTGCATTAATATATTTACCC
CATATACATTACATTCATGCTAGGAAAACACATTCAATGCATGTACTATGTACATCAACATTTTACGGGCAATCATCACTCCCACCACACTCCCCCCACAAGGACAATTTAATCAATGATCTACGGACAAGCCTCCTAACTCCAAGTACTAAAACCAGTTAAATGTTAGCTTAT
ACCTACCTCAATCTCCCGAATACGACAGTGCTTGAACACATAACATTAATGGTATCCGGACAAACCATGCATCCTTTCTCGTAGTGCCTGCGTCTGAAGGACCAGGTTATCTATTAATCGTTCTTCTCACGTGAAATCAGCAACCCGCCGCATATAAGACCCAACACGACCAGC
TTCAGGACCATTCTTTCCCCCTACACCCCTAGCCCTACTTGCACTTTTGCGCCTCTGGTTCCTATATCAGGGCCATTCCTTGATTCATCCTCTCAACTTGCTCTTCACAGAAACATCTGGTAGGCTATATATTAGCATCTCACCCGTGATCGCGGCATCTGGATAGCCTTAGCA
CTTTTGGTTCCCTTTTTTTTTGGGGCGTCTTCACAGGTCACCCTTCCAGTGCGGCGGGAAATAACAATCTAAGACCTGAGCATACACTGCGTTGCGTCCTAATTTTGGCCCTCAAGAATTACTGAATGAGACGGTTTGCGTATATGGGGAATCATTTTGACACTGATGCACTTG
CTGTTACATTTGGTTATGGTATCCCCGCAAGCTCTAACTTATGTGGCTATTTAGTGAATGCTTGTTGGACATGTTTTCTTACCTTTACACTTCCTCTAACTTTTCAACCAACACTAGAGGCTTTTCGACCAAATACCTATCGTATGCTAGTCACGAATTTTATTCACACTTTTT
TTACACAACATCACCGCTGAAATTACATTAACAAAGCTAACCTTTGCCATCTTTCATATCTTCTTCTTAACATCACAAAACCTTCCGCTAAAATCTCAACTAAATTCTCCTACACAAACTCGAACCCCAAACCAGGTTCAAACAACCCTTTGGGCATCGTATCAAGCTGTGACA
AAATCCCATTCCATCCCTACTTCACCCTAAAAGATGTCCTAGGATTCATGCTCATATTTCTCCCCCTAACAACCCTAGCCCTATTCTCACCTAATTTATTAGGAGACCCAGAAAACTTCACACCCGCAAACCCTCTAGTCACACCACCCCATATCAAGCCAGAGTGATATTTCC
TGTTCGCCTATGCCATTCTCCGCTCAATCCCCAACAAACTAGGAGGAGTACTAGCCCTAGCTGCCTCCGTACTAATCCTATTCCTAATACCTTTCCTCCATAAATCTAAACAACGTACAATAACCTTTCGCCCACTCTCCCAACTCCTATTCTGAACACTAGCAGCCAACCTTC
TCATCCTAACATGAGTGGGCAGCCAACCAGTAGAACATCCCTTCATCATCATCGGCCAACTAGCCTCCCTCACCTACTTCACCATCCTCCTTATCCTCTTCCCTATCCTCGGAGCCCTAGAAAACAAAATACTCCATCAAAAATACTCTAATAGTTTATAAAAACATTGGCCTT
GTAAGCCAAAGAATGAAGACTACGCCCCTTCTTAGAGTTTAAACCACACCAACCCTCCCACCAAAACACCTCAGAAAAAGAGGACTTAAACCTCTATCCCCAGCTCCCAAAGCTGGTATTTTACACTAAACTATTCTCTGACGTCCACCCTAAACCGCCCGAATTGCCCCACGA
GACAACCCCCGCACAAGCTCTAACACAACAAACAAAGTCAACAACAACCCCCACCCAGCCACTAAAAATATTCCGGCCCCGCGCGAATAAAATATTGCTGCACCACTAAAATCCAACCGAATTGAAAACAACCCCACACTATCAACAGTGACTACTCCCCACTCCCAACCCCCT
ACAACTCCTACAACCCCCACACCCACAACAAGTACTAAAACCAGCCCCGCCCCATATCCTACTACACGCCGATCCCCCCAAGCTTCCGGAAAAGGATCGGCCGCCAAAGACACAGAGTACACAAATACCACCAACATTCCGCCCAAATAGACCATAAATAGTACCAAAGATACA
AAAGAAACCCCCAAACTCAACAACCACCCGCAACCCATGACAGATGCCAGTACTAAGCCAACCACCCCATAATATGGAGAAGGGTTGGATGCTACTGCCAGCCCCCCTAAAACAAAACATACCCCTAGAAAAAGCACAAAATAAGTCATCAGCAGTTTCTGCTTGGCTTTTCTC
CAAGGTCTATGGCTTGAAAAGCCATCGTTGAAACTATCTCAACCACAGAAACACTGGAAATACATTAAAAAAGGCTACCCCCCCTACCCCCCCATAATAATTTGCCCTACCCATGTATGGTTTTACATTAATATACCCACCCCATATACATTATATCCATGCTAGGAAAACATA
TTTAATCCATGTACTATACACATAACAATGTATGTACTATAAGCATTATAATGTATGCAGCACGTCCATCAAACTTATACCCGGATATACTTAAACCAACAGACCATTTCTCCCACAAGGACAATTTAATCAATGATCTACGGACAAGCCTCCTAACTCCAAGTACTAAAACCA
GTTAAATGTTAGCTTATACCTACCTCAATCTCCCGAATACGACAGTGCTTGAACACATAACATTAATGGTATCCGGACAAACCATGCATCCTTTCTCGTAGTGCCTGCGTCTGAAGGACCAGGTTATCTATTAATCGTTCTTCTCACGTGAAATCAGCAACCCGCCGCATATAA
GACCCAACACGACCAGCTTCAGGACCATTCTTTCCCCCTACACCCCTAGCCCTACTTGCACTTTTGCGCCTCTGGTTCCTATATCAGGGCCATTCCTTGATTCATCCTCTCAACTTGCTCTTCACAGAAACATCTGGTAGGCTATATATTAGCATCTCACCCGTGATCGCGGCA
TCTGGATAGCCTTAGCACTTTTGGTTCCCTTTTTTTTTGGGGCGTCTTCACAGGTCACCCTTCCAGTGCGGCGGGAAATAACAATCTAAGACCTGAGCATACACTGCGTTGCGTCCTAATTTTGGCCCTCAAGAATTACTGAATGAGACGGTTTGCGTATATGGGGAATCATTT
TGACACTGATGCACTTGCTGTTACATTTGGTTATGGTATCCCCGCAAGCTCTAACTTATGTGGCTATTTAGTGAATGCTTGTTGGACATGTTTTCTTACCTTTACACTTCCTCTAACTTTTCAACCAACACTAGAGGCTTTTCGACCAAATACCTATCGTATGCTAGTCACGAA
TTTTATTCACACTTTTTTTACACAACATCACCGCTGAAATTACATTAACAAAAGAAAGAACCAACATGCACGCACACACCGGCGCGCAGCGCCAAAACTTTTAAAGAATATCCTAACTAACAACACCAACACAACACAACACAACACCACCAACACCAACCACCAACCACCAACAC
AACACAACACAAAACCACAAACAACAAACAACAAACAACAAACAACAAACAACAAACAACAAACAACAAACAACAAACAACAAACAACAAACAACAAACAACAAACAACAAACAACAAACAACAAACAACAAACAACGCCAAATCGTT**GTTTTCGTAGCTTAACAAT**
AAAGCGTGGCACTGAAGATGCCAAGATGGCCTACCACATGCCCGATGACAAAAGACTTAGTCCTAACCTTACCGTTAATTCTTGCTAAACATATACATGCAAGTATCCGCGTCCCAGTGTAAATGCCCCTAATCTCTTACCAAGACAAAAGGAGCGGGTATCAGGCACACCCAC
GACTAGTAGCCCAAGACACCTTGCCCAGCCACACCCCCACGGGTACTCAGCAGTAATTAACATTAAGCAATAAGTGTAAACTTGACTTAGTTATAGCAATTATTCAGGGTTGGTAAATCTTGTGCCAGCCGCCGCGGTCATACAAGAGACCCAAATTAACCGTACACGGCGTAA
AGAGTGGGATTAGTACTATCACAGCAACTAAGATCAAAGCGCAACTAAGCCGTCATAAGCCCAAGATGCACCTAAGACCATCCTTAAGATGATCTTAGCGCCTCACGATTAATCAAACCCCACGAAAGCTTGGACACAAACTGGGATTAGATACCCCACTATGCCTGGCCCTAA
ATCTCGATGCTTAGCTCACCAAAGCATCCGCCTGAGAACTACGAGCACAAACGCTTAAAACTCTAAGGACTTGGCGGTGCCCCAAACCCACCTAGAGGAGCCTGTTCTATAATCGATAATCCACGATACACCCGACCGTTCCTTGCCAAAGCAGCCTACATACCGCCGTCGCCA
GCTCACCTTTCCTGAAAGCACAACAGTGGGCACAATAGCATTAACCTCGCTAACAAGACAGGTCAAGGTATAGCCCATGGGGCGGAAGAAATGGGCTACATTTTCTAAGTTAGAAAACTCCACGAAAAGAGGTGTGAAACCACCCCTAAAAGGCGGATTTAGTAGTAAAGTGGG
ATAATAGAGCCCCCTTTAAACTGGCCCTGGGGTACGTACATACCGCCCGTCACCCTCCTCACAAGCTCCAACTTCTTATAACTAATCCACCTATTAGCTGAAGATGAGGTAAGTCGTAACAAGGTAAGTGTACCGGAAG

Chrysolophus pictus (Galliformes/Phasianidae) mitogenome from CR1 to CR2 (MW151829)

GO-GA in Figure 8

CTATATAGTGAATGCTTGTCGGACATATTTTTACAAATTTTCACTTCCTCTATTTTCTCCACAAAACTAGGAGATTTACCACAATTTTTTCTTTGTTATTTTGTTATTTTTTTTAAAAACATTTTTAAAAAACTAAATTAAACTAAAACTACCGCATAAAAACCCTCAAACCAA
AAAAACGTTTCGTTTAGTATATATACATTGTTATATACATCACTTTTATTAGAGAAACTCCACTACCAAATCTTACTTTTTAAAAACAAAAATTACATTGGACAAAACCCTACAAGTAAACATTATTTATATTGATGATAAACAAATGACTCAATTCTCCTACATCCAACA**GCC**
TCCATAGCTTAATCTAAAGCATGGCACTGAAGATGCCAAGACGGTACCTATAATACCTGCAGGCA*AAAGACTTAGTCCTAACCTTGCTATTGATTTTTGCTAGACATATACATGCAAGTATCTGCACGCCAGTGAAAATGCCCTAACATCTTTGACAAGAAAAAGGAGCAGGTA*
TCAGGCACACCCATGAGTAGCCCAAAACACCTTGCCACAGCCACACCCCCACGGGTATTCAGCAGTAATTAACCTTAAGCAATAAGTGTAAACTTGACTTAGCCATAGCAATCCTCAGGGTTGGTAAATCTTGTGCCAGCCACCGCGGTCATACAAGAAACCCGAATCAATAGC
CAACCGGCGTAAAGAGTGGCCATATGTTATCCTCATTACCTAAGATCGAAGTGCAACTAAGCTGTCATAAGCCCAAGATCCACTTAAGGCCCCCTCCCAACCATCTTAGCCTAACGACTAATTTCACCCCACGAAAGCCAGGACACAAACTGGGATTAGATACCCCACTATGCC
TGGCCCTAAATCTAGATGCCTGCATACCCATGCATCCGCCCGAGAACTACGAGCACAAACGCTTAAAACTCTAAGGACTTGGCGGTGCCCCAAACCCACCTAGAGGAGCCTGTTCTGTAATCGATAGTCCACGATTCACCCAACCACCCCTTGCCAACACAGCCTACATACCGC
CGTCGCCAGCTCACCTAAAATGAAAGACCAACAGTGGGCCCAACAGTCATACCACTAGCAAGACAGGTCAAGGTATAGCCCATGGGGTGGAAGAAATGGGCTACATTTTCTAACATAGAATAAACGAAAAAGGGCGTGAGTTAGATGCCACAGCCAAAACCCCTAACATAAAAC
*AAACCCCAAGAAAAATCACAAAATAGGTCAT*ATATTCCCGCTTGGATAGACCCCAAGGACTACGGCTTGAAAAGCCATTGTTGTTCTCAACTACGGGAACAACAAACTTTTTTAACTCAACTCCCCTATTGAATGTACCCCCCCCTTCCCCCCCAGGGGGGGTATACTATGCAT
AATCGTGCATACATTTATATACCACATATACTATGGTACCGGTACTATATATTATAATCGTACTAAACCCATTATATGTATACGGACATTACACCACAGCCACATTTCTCCCCATGTACATATCCATGCAATGCTCCCAAACTATCCAAGCTCCCTCACCAAACAATGCTCTAC
AACTTCCAGGTCCCCATATTCATGAATGGTTACAGGACATACATGTAATACTAGGTATTTCCCCATTTGGTTATGCTCGACGTACCAGATGGATTTATTGATCGTACACCTCACGAGAGATCAGCAACCCCTGCCTGTAATGTACTCTATGACTAGCTTCAGGCCCATTCTTTC
CCCCTACACCCCTCGCCCCTCTTGCTCTTTTGCGCCTCTGGTTCCTCGGTCAGGAACATCCCATGTTTAACTCCTGAATTCCTCACTTTTCACGAAGTCATCTGTGCGTTATTCTCCCCTCTTTAGTCCGTGATCGCGGCA

Ciconia nigra (Ciconiiformes/Ciconiidae) mitogenome from CR1 to CR2 (MH264509)

GO-FD in Figure 8

CTGAATGAGACGGTTGGCGTATATGGGGAATCATCTTAACACTGATGCACTTTGGTTTCCATTTAGTTATGGCGTATCCACAGACTCTTTAAAGTGTTACTATTTAGTGAATGCTTGTTGGACATGCTATTCTACTTTTACACTTCCTCTAACTTTCTAAACAACACTAGGAAC
TTTTACTTAAATCCATTTTTATCGTTCACACATATTTCATTCGTTCTTTCATCGTTCGTTCGATCAATCTATTCAATACTAAAATTTCATTAATAAATCAAACGTTCATTCATGTGTTTGTTTCCTTTTGTTTGTTTGATCGATCAATCTATTCAATACTAAAATTTCATTAA
TAAATCAAACGTTCATTCATGTGTTTGTTTCCTTTTGTTTGTTTGATCGATCAATCTATTCAATACTAAAATTTCATTAATAAATCAAACGTTCATTCATGTGTGTTTCCTTTTGTTTGTTTGATCGATCAATCTATTCAATACTAAAATTTCATTAATAAATCAAACGTTCAT
TCATGTGTTTGTTTCCTTTTGTTTGTTTGATCGATCAATCTATTCAATACTAAAATTTCATTAATAAATCAAACGTTCATTCATGTGTTTTTCCTTTTGTTTGTTTGATCGATCAATCTATTCAATACTAAAATTTCATTAATAAATCAAACGTTCATTCATGTGTTTGTTTCC

TTTTGTTTGTTTGATCGATCAATCTATTCAATACTAAAATTTCATTAATAAATCAAACGTTCATTCATGTGTGTTTCCTTTTGTTTGTTTGATCGATCAATCTATTCAATACTAAAATTTCATTAATAAATCAAACGTTCATTCATGTGTTTGTTTCCTTTTGTTTGTTTGATC
GATCAATCTATTCAATACTAAAATTTCATTAATAAATCAAACGTTCATTCATGTGTTTTTCCTTTTGTTTGTTTGATCGATCAATCTATTCAATACTAAAATTTCATTAATAAATCAAACGTTCATTCATGTGTTTGTTTCCTTTTGTTTGTTTGATCGATCAATCTATTCAAT
ACTAAAATTTCATTAATAAATCAAACGTTCCCTTTTACACCCTCCACTTACATTCCCCTTCCTTCACGAATCCGGCTCAAACAATCCCCTGGGCATCGTCTCAAATTCCGACAAAATCCCATTTCACCCTTACTTTTCCCTAAAGGATGCCCTAGGCTTTACACTCCTGCTCCT
TCCACTAACCGCCCTCGCCCTACTCTCACCCAACCTGCTAGGCGACCCAGAAAACTTCACCCCAGCCAACCCATTAATCACACCTCCCCACATCAAGCCTGAATGATACTTCCTCTTTGCATACGCCATCCTACGCTCAATCCCCAACAAACTAGGAGGAGTACTGGCCCTCGC
CGCCTCCGTACTAATCCTATTCCTCTGCCCCCTCCTCCACAAATCCAAACAGCGCACAATGGCCTTCCGCCCCCTATCTCAACTTCTATTTTGAACCCTAACTGCCAACCTACTCATCCTCACATGAGTAGGCAGCCAACCAGTAGAGCACCCATTCATTATCATCGGCCAACT
GGCCTCCTTCACCTACTTCGCCACCCTCCTAATCCTCTTCCCTGTCGCCGGAGCCCTAGAAAACAAACTACTCAACTACTAGATACTCTAATAGTTTATAAAAAACATTGGTCTTGTAAACCAAAGAGTGAAGGCCACACCCTTCTTAGAGTTACCCCTAGCATCTCAGAGAGA
GAGGACTCAAACCTCCACCTCCAACTCCCAAAGCTGGCATTTTACATTAAACTACTCTCTGGCCCCCTAACCCCTGACTTTAAACCGCTCGAATCGTCCCACGGGACAACCCCCGCACAAGCTCCAACACAACAAGCAACGTCAACAGCAACCCCCATCCCGCCATCAAAAACA
TTCCCACCCCACACGAATAGAACACCGCCACCCCACTAAAATCTAGCCGAACAAAAGACAAACCCCCACTATCAACTGTAATTACCTCGAACTTCAAGCCCTCAACAAAACCCCCAACAACTCCCCCAACCACCAGCATCAGAATAAGCCCCATGCCATACGCCACCACCCGCC
AGTCCCCTCAAGCCTCAGGAAAGGGGTCCGCAGCCAAAGATACCGAGTATACAAAAACCACCAACATTCCCCCAAATAAACCATAAATAGTACCAAAGACACGAAGGATAACCCCAAACTCAACAACCAACCACACCCTACAACAGATGCTACCACCAAACCAACCACCCCAT
AGTAGGGCGAAGGATTAGATGCAACCGCCAATGCTCCCAAAACAAAGCAGAGCCCCCCAAAAGCACAAAGTAAGCCATCATATGTTCCCACTTGGCCTCTCTCCAAGGTCTGCGGCTTGAAAAGCCGCCGTTAAAAATGACTCTCAACTACAGGAACAGCCCAAGGACATGAC
CCTCGGTACCCCCCCTACCCCCCCACAGAGAACTGCAGTGGAATGACTATGTAATTGTGCATTAGTCTGCTTGTCCGCTTAGCATTACACTGATCTTTGGACATACATTTTAATTGGTTTAGGGCATATTATTGTAATGTTCTTAGACATAATATGTATGTAGTCAAGTGGAC
ATGATATTGTAATGCCTTAGGATTAATAGTGAAATGGGCACCGGATAAGGTTTGTCCTTAATCAGGGATTTAAAGTTGAATGAAGGTAGGAATGGGGTATGTAATGATATAGGCCTAGAGATCTAGGGGATGGTTAGAGTCATTTTCTTGAGGGAGAGGGTATTTAAAGTCTTG
GTAAGCGGTTATCCCAGAGGATTGGAATGTCATGAGGAGGGAATAGGGCTGAAAGGTTAGGACTAAACCCATGATAGGCTTGGGTCTGTACAGAAGTTGTTAGTAAGATACGGCTATTCTTGAGGGGTAGAATTTGAATGGTAGCTGGATATGAAATCTCAGTTCCTGCTCGT
AGGGCCGGTAGCTGAAGGACCAGGTTATCTATTAATCGGAGTTCTCACGTGAAATCAGCAACCCGGTGTCTGGAAGATCCGRTGTGACTAGCGTCAGGAT

Corythaixoides personatus (Musophagiformes/Musophagidae) mitogenome from CR1 to CR2 (MW082596)

GO-FD in Figure 8

CAACTTGCRCTTTTGCGCCTCTGGTTCCTCTATCAGGGCCACAACTTGGTAGTTCCTCTCTTCTTGTACTTCACAGAAACAACTGGTTGGCTATATATCACCATTCTCCCTCTTAATCGCGGCATCCGGGGGGTTTGGCACTGTTGGTTCCCTTTTTTTTTCTGGGCGTCTTCA
GGCTGCCCTTCCAAGTGCACCGGGTGAATACAATCTCTTGACGTGGGCTACACTGGTCGGCGTCCTATTTTTTGGCTCTCAGGAATCCATTAATGAGACGGTTTGCGTACGAGCCGGGCAGGCTAGTTCTGACACTGTGCACTGTGTTTTACACCTGGTAATGGTAGATCCACA
GACTTATATTATGCTGACTATTGATGAATGCTTGATGGACATGATTTTTCACTTTTACACTTCCTCTAATTTTCTTAACAACACTAGGCACTTTCAACTAAAAATTCATCGTTTCTTTTTATAATTTTTTATCGATTTTATTTTATACCTTTCATATATGCATTTTTAACATGT
CATTGCCACTGGAGTTGCATTAAAAAATTAAAACAATTTTTTCATCACTTTTTATTCCATTCACCTTCCTCCATAAATCCAAACAACGCACAATAACCTTCCGCCCCCTCTCCCAGCTACTATTCTGAACATTAGTTGCCAACCTCATCATCCTAACATGAATTGGTAGCCAAC
CAGTAGAACACCCCTTCATCATTATCGGCCAACTGGCCTCCCTCACCTACTTCACCATCATCTTAATCCTCTTCCCCCTCATCAGTGCCCTAGAAAACAAAATCCTCTACTAAATAACTCTAATAGTTTATAAAAACATTGGCCTTGTAAGCCAAAGATTGAAGACCACGCCCC
TTCTTAGAGTTTCCCAATCTCCCCAGAACCCCGCCCGCCTCAAAGAAAGAGGACTTAAACCTCTAACTCCAACTCCCAAAGCTGGCATTTTTCATTAAACTATTCTCTGATCTACCCCCCTACACAGCCCGAATGGTCCCACAAGACAACCCCCGTACAAGCTCCAGCACAACG
AACAAAGTCAACAATAATCCCCACCCCGCCACCAAAAACATCCCCACTCCTAACGAATAAAACAAAGCCACTCCACTAAAATCCAACCGAGCAGCAAACATACCCCCACTATCAACAGTACTCACTCACCACCCCCAACACTCACCAACACCCCCAACAACCCCTCCCACAACA
AGAACCAGCACAAACCCTGCACCATACCCCACAACACGTCAATCCCCCCAAGCCTCCGGATACGGATCTGCAGCCAACGACACAGAGTACACAAAAACTACTAGCATCCCCCCCAAATAGACTATAAATAGAACCAACGACACAAAAGAAACCCCTAAACTCAACAACCACCCA
CATCCCACAACCGATGCTAGCACCAAACCAACCACCCCATAATACGGAGAAGGATTAGACGCAACCGCCAAACCCCCTAAAACAAAGCATAACCCCATAAAAAACAAAAAATAAGCCATAGCAATTCCTGCTTGGCTTTTCTCCAAGACTTGTGATCTGAAAAACCACCGTTAG
TAAATTTCAACTACAGGAACAAATAGCCCTACAGACCCCCCCGGTACCCCCCCTACCCCCCCATTACTTAACGGAGGAACTCTATGTACTATGTTACATTAATCTATTTACCCCATACTATGTAATGTAATTCAGGAGAATACATTTAATGTATGTACTAAGGTCATATTATGT
TACTCGGGCATACTCCTCTTTAGTCCATCTCTACATTCAAGGACCTAATTATGTAATGACCCTGAGGAATTGCAACTATCTCCCCTCGGACTAAAACCATTCTTCATTCAGTACCGTACCTAACACAGTTCCCTTACACGGCAGTGTACTTCCACATAAATATCCATGCTCGAG
GACCAACCTTGAAATATCTCTTGACGGACCGGTTTCTGAAGGACCAGGTTATCTATTAATCGAGCTTCTCACGAGAAATCAGCAACCA

Eurypyga helias (Eurypygiformes/ Eurypygidae) mitogenome from CYTB to 12S (MW208859)

CCTATTACTAGCTGCACACTACACAGCAGACACAACACTAGCTTTCTCATCTGTCGCTCACACCTGTCGAAATGTTCAATACGGTTGACTAATTCGAAACCTCCATGCAAACGGCGCATCCCTGTTTTTCATCTGCATCTACCTACACATTGGACGAGGGCTTTACTACGGCTC
CTATCTCTATAAAGAAACCTGAAACACAGGCATCCTCCTCCTCCTCACCCTTATAGCAACTGCTTTCGTAGGATATGTCCTCCCGTGAGGACAAATATCCTTCTGAGGTGCCACAGTCATCACTAATCTATTCTCAGCCACCCCATACATTGGACAAACCCTTGTAGAATGGGC
CTGAGGAGGTTTCTCAGTAGACAACCCTACCCTAACACGATTCTTTACCCTACACTTCCTCCTTCCATTCATAATCGCTGGCCTTACACTCATCCATCTAACTCTCCTTCACGAATCTGGATCAAACAACCCCCTAGGAGTCATCTCCAACTGTGACAAAATCCCCTTCCACCC
CTACTTCACCACAAAAGATATCCTTGGATTCATACTCATACTCCTCCCCCTCATAACCCTTGCCCTATTCACGCCCAACCTCCTGGGAGACCCAGAAAATTTCACCCCAGCAAACCCACTTGTCACCCCACCCCACATTAAACCAGAATGGTACTTCCTATTTGCCTATGCCAT
CCTACGATCAATCCCCAACAAACTAGGCGGCGTCCTAGCATTAGCTGCATCCATTCTAGTCCTATTTACCGCCCCGCTCCTCCACAAAGCAAAGCAACGTACAATAACCTTCCGCCCCCTGTCACAACTCCTATTCTGAACCCTCGTTGCTAATCTATTCATCCTTACCTGAGT
TGGAGGACAACCAGTAGAACACCCATTTATCATCATCGGCCAATTAGCTTCTATTACTTACTTCACCATCCTCTTAGTCCTCTTTCCCCTCGCTGGCGCCCTAGAAAACAAAATACTCAACCATTAATCACTCTAATAGTTTAGCACAAAACATTGGTCTTGTAAGCCAAAGCC
GGAGGCCTCCCTACCCTCCTTAGAGTTGCCCATCAGAAAAAGAGGACTCAAACCTCCATCACCAACTCCCAAAGCTGGTATTTTCAACTTAAACTATTTTCTGATACACCAACCCCTAAACCGCCCGAATAGCCCCCGAGACAACCCCCGTACAAGCTCTAGCACCACAAATA
GAGTCAACAACAATCCCCACCCTGCCATCAAAAACATTCCGGCGCCCCACGAATAGAACGTAGCCACACCACTAAAATCTAACCGAGCAGAAAACAAACCCACACCATCCACAGTAACAACCCCAAAATCAACCCCACCCCCCAACCCTCCAATAACTGCCCCCACCACAAGTA
CTAAAACAAACCCCATACTATATTCCACAACCCCACGATCCCCCCAAGCCTCAGGAAATGGCTCTGCTGCTAAAGCCACAGAATACACAAAAACAACCAACATTCCACCCAAATAAACCATAAACAAAACTAATGACACAAAAGAAACCCCTAAACTCAGCAACCATCCACACC
CTGCAACAGATGCTACTACTAAGCCTATCACCCCATAATAAGGCGATGGGTTAGAAGCAACTGCTAGCGCCCCCAAAAGAAACCAACCCCCTAAAAACAGTACGAGAGAAGCCATAGCAATTCCTGCTTGGCTTCTCTCCAAGGCCTATGGTCTGAAAAACCATCGTTGATTCT
CAACTACAGGAACCTCTTACATTTAACATACATAGCAAGGGCCCCCCCTTCCCCCCCACCAGTGCTTTTAATGCAACTCGCAGGCTATGTATTAATGTACATTACATTATATTCCACATATATCATATGATATTAGTACATATTATGCATGCATCGATGACATTTTATGTATAC
GGGCATACCCCCCCCCCACAACAGTTCTCCCACAAGGACCAATTAATGAAATGATTCTAGGAACAACCCCTACACTCTTCGAACTAAACCCATAACACAATGGCTTATACATAGCAATTTCCTCCATACGGATATCCTTGACCACATAATATCAATGGTATCAGGACACAGTAA
TGCAACAACTCTTGTAGTACCGGCTTCTGAAGAACCAGGTTATCTATTAATCGAGCTCCTCACGTGAAATCAGCAACCCGCCGCATATAAGATCCTCGTTACTAGCTTCAGGACCATTCTTTCCCCCTACACCCCTAGCCCATCTTGCTCTTTTGCGCCTCTGGTTCCTTTTTC
AGGGCCATCACCTTAACGTCTCCCTTCTCCTTTCTCTTCACAGAGCCATCTGGCTGGGCTCTTGCTCACCATTCTCCCTCTTAATCGCGGCATCCCCCAGCCTAGGCGCCTTCCTTCCTTTTTTTTTGGGGCGTCTTCAATCTGCCCTTCCAGTGCATATGGGTACTATGGTTT
ATAGACGTGAGCATCTGGTTATGCGTGGCGTACTCATCTTGCCCTCAGGTCCTACTCAATGAGACGGTCTGCGGTTATTCAGTATCATTTTGGCACTGATGCACTTGCTGTTACATTTGGTTCTGGCTCTTCCATCCTACGATTAAAATGGGGTATTTAAGTGAATGCTTGTAA
GACATAAAAAATTTTATTTTATTTCATCTAACTTTCTTAACAAAACTAGGAAATTTCAACTAAATTCCTTCGTCGTCGTCAAACACGTTTTTCGATCGTATTCTTCGCATCGAAAATCGTTCGTTCCCCTTTCCAATTCCGCTAAAATTTCATTAATAACTCAAAACATATTTT
CGCGAATTTGATTTAACATAAAAAATAAATGACAAACGACAAGATTTGTCAAACGACAAAGTTTGTCAAACGACAAAGTTTGTCAAACGACAAGATTTGTCAAACGACAAAGTTTGTCAAACGACAAAGTTTGTCAAACGACAAAGTTTGTCAAACGACAAAGTTTGTCAAACG
ACAAAGTTTGTCAAACGACAAAGTTTGTCAAACGACAAAGTTTGTCAAACGACAAAGTTTGTCAAACGACAAAGTTTGTCAAACGACAAAGTTTGTCAAACGACAAAGTTTGTCAAACGACAAAGTTTGTCAAACGACAAAGTTTGTCAAACGACAAAGTTTGTCAAACGACAA
AGTTTGTCAAACGACAAAGTTTGTCAAACGACAAAGTTTGTCAAACGACAAAGTTTGTCAAACGACAAAGTTTGTCAAACGACAAAGTTTGTCAAACGACAAAGTTTGTCAAACGACAAAGTTTGTCAAACGACAAAGTTTGTCAAACGACAAAGTTTGTCAAACGACAAAGTT
TGTCAAACGACAAAGTTTGTCAAACGACAAAGTTTGTCAAACGACAAAGTTTGTCAAACGACAAAGTTTGTCAAACGACAAAGTTTGTCAAACGACAAAGTTTGTCAAACGACAAAGTTTGTCAAACGACAAAGTTTGTCAAACGACAAAGTTTGTCAAACGACAAAGTTTGTC
AAACGACAAAGTTTGTCAAACGACAAAGTTTGTCAAACGACAAAGTTTGTCAAACGACAAAGTTTGTCAAACGACAAAGTTTGTCAAACGACAAAGTTTGTCAAACGACAAAGTTTGTCAAACGACAAAGTTTGTCAAACGACAAAGTTTGTCAAACGACAAAGTTTGTCAAAC
GACAAAGTTTGTCAAACGACAAAGTTTGTCAAACGACAAAGTTTGTCAAACGACAAAGTTTGTCAAACGACAAAGTTTGTCAAACGACAAAGTTTGTCAAACGACAAAGTTTGTCAAACGACAAAGTTTGTCAAACGACAAAGTTTGTCAAACGACAAAGTTTGTCAAACGACA
AAGTTTGTCAAACGACAAAGTTTGTCAAACGACAAAGTTTGTCAAACGA**NNNNNNNNNNNNNNNNNNNNNNNNNNNNNNNNNNNNNNNNNNNNNNNNNNNNNNNNNNNNNNNNNNNNNNNNNNNNNNNNNNNNNNNNNNNNNNNNNNNNNNNNNNNNNNNNNNNNNNNNNNNNNNNNNN**
NNNNNNNNNNNNNNNNNNNNNNNNNNNNNNNNNNNNNNNNNNNNNNNNNNNNNNNNNNNNNNNNNNNNNNNNNNNNNNNNNNNNNNNNNNNNNNNNNNNNNNNNNNNNNNNNNNNNNNNNNNNNNNNNNNNNNNNNNNNNNNNN
NNNNNNNNNNNNNNNNNNNNNNNNNNNNNNNNNNNNNNNNNNNNNNNNNNNNNNNNNNNNNNNNNNNNNNNNNNNNNNNNNNNNNNNNNNNNNNNNNNNNNNNNNNNNNNNNNNNNNNNNNNNNNNNNNNNNNNNNNNNNNNNN
NNNNNNNNNNNNNNNNNNNNNNNNNNNNNNNNNNNNNNNNNNNNNNNNNNNNNNNNNNNNNNNNNNNNNNNNNNNNNNNNNNNNNNNNNNNNNNNNNNNNNNNNNNNNNNNNNNNNNNNNNNNNNNNNNNNNNNNNNNNNNNNN
NNNNNNNNNNNNNNNNNNNNNNNNNNNNNNNNNNNNNNNNNNNNNNNNNNNNNNNNNNNNNNNNNNNNNNNNNNNNNNNNNNNNNNNNNNNNNNNNNNNNNNNNNNNNNNNNNNNNNNNNNNNNNNNNNNNNNNNNNNNNNNNN
NNNNNNNNNNNNNNNNNNNNNNNNNNNNNNNNNNNNNNNNNNNNNNNNNNNNNNNNNNNNNNNNNNNNNNNNNNNNNNNNNNNNNNNNNNNNNNNNNNNNNNNNNNNNNNNNNNNNNNNNNNNNNNNNNNNNNNNNNNNNNNNN
NNNNNNNNNNNNNNNNNNNNNNNNNNNNNNNNNNNNNNNNNNNNNNNNNNNNNNNNNNNNNNNNNNNNNNNNNNNNNNNNNNNNNNNNNNNNNNNNNNNNNNNNNNNNNNNNNNNNNNNNNNNNNNNNNNNNNNNNNNNNNNNN
NNNNNNNNNNNNNNNNNNNNNNNNNNNNNNNNNNNNNNNNNNNNNNNNNNNNNNNNNNNNNNNNNNNNNNNNNNNNNNNNNNNNNNNNNNNNNNNNNNNNNNNNNNNNNNNNNNNNNNNNNNNNNNNNNNNNNNNNNNNNNNNN
NNNNNNNNNNNNNNNNNNNNNNNNNNNNNNNNNNNNNNNNNNNNNNNNNNNNNNNNNNNNNNNNNNNNNNNNNNNNNNNNNNNNNNNNNNNNNNNNNNNNNNNNNNNNNNNNNNNNNNNNNNNNNNNNNNNNNNNNNNNNNNNN
NNNNNNNNNNNNNNNNNNNNNNNNNNNNNNNNNNNNNNNNNNNNNNNNNNNNNNNNNNNNNNNNNNNNNNNNNNNNNNNNNNNNNNNNNNNNNNNNNNNNNNNNNNNNNNNNNNNNNNNNNNNNNNNNNNNNNNNNNNNNNNNN
NNNNNNNNNNNNNNNNNNNNNNNNNNNNNNNNNNNNNNNNNNNNNNNNNNNNNNNNNNNNNNNNNNNNNNNNNNNNNNNNNNNNNNNNNNNNNNNNNNNNNNNNNNNNNNNNNNNNNNNNNNNNNNNNNNNNNNNNNNNNNNNN
NNNNNNNNNNNNNNNNNNNNNNNNNNNNNNNNNNNNNNNNNNNNNNNNNNNNNNNNNNNNNNNNNNNNNNNNNNNNNNNNNNNNNNNNNNNNNNNNNNNNNNNNNNNNNNNNNNNNNNNNNNNNNNNNNNNNNNNNNNNNNNNN
NNNNNNNNNNNNNNNNNNNNNNNNNNNNNNNNNNNNNNNNNNNNNNNNNNNNNNNNNNNNNNNNNNNNNNNNNNNNNNNNNNNNNNNNNNNNNNNNNNNNNNNNNNNNNNNNNNNNNNNNNNNNNNNNNNNNNNNNNNNNNNNN
NNNNNNNNNNNNNNNNNNNNNNNNNNNNNNNNNNNNNNNNNNNNNNNNNNNNNNNNNNNNNNNNNNNNNNNNNNAAGTTTGTCAAATTTATCAAACTTGTCAAACTTGTCAAACTTGTCAAACTTGTCAAACTTGTCAAACTTGTCAAACTTGTCAAACT
TGTCAAACTTGTCAAACTTGTCAAACTTGTCAAACTTGTCAAACTTGTCAAACTGGTCAACCGACAAAATTTGTCAAACAACAAAATTTGTCAAACAACAAAATTTGTCAAACAACAAAATTTGTCAAACAACAAAATTTGTCAAACGACAAAGTTTGTCAAATTTATCAAACG
ACAAAGTTTGTCAAACGACAAAGTTTGTCAAACGACAAAGTTTGTCAAATTTATCAAACGACAAAGTTTGTCAAATTTATCAAACGACAAAGTTTGTCAAATTTATCAAACGACAAAGTTTGTCAAATTTATCAAATGACAAAGTTTGTCAAATTTATCAAATGACAAAATTTA
TCAAACTTGTCAAACTTGTCAAACTTGTCAAACTTGTCAAACTTGTCAAACTTGTCAAACTTGTCAAACTTGTCAAACTTGTCAAACTTGTCAAACTTGTCAAACTTGTCAAACGACAAAATTTGTCAAACGACAAAATTTGTCAAACGACAAAGTTTGTCAAATTTATCAAACGACAAAGTT
TGTCAAATTTATCAAACGACAAAGTTTGTCAAATTTATCAAACGACAAAGTTTGTCAAACGACATTT**GTCCTCGTAGCTTAACCACCAAAGCATGGCACTGAAGATGCCAAGATGGTCGTTGTCCAACTACCCGACGACA**AAAGACTTAGTCCTAACCTTACCGTTAATTTTTG
CTAAGTATATACATGCAAGTATCCGCGCCCCAGTGTAAATGCCCCTGTCACCTTTATCAAGATGAAGGGGGCTGGTATCAGGCACACGTTCAACCGTAGCCCAAGACGCCTTGCTCAGCCACACCCCCACGGGTACTCAGCAGTAATTAACATTAAGCAATAAGTGCAAACTTG
ACTTAGCTATAGCAGTTCCTACATAGGGCCGGTAAATCTTGTGCCAGCCACCGCGGTCACACAAGAGGCTCAAATTAACCGTCCGCGGCGTAAAGAGTGGTACAATGTTAACGCACAAACTAAGACCAAAATACAACTAAGCTGTCATAAGCCCAAGATGTACCTAAGCCTCCC
ATCAAAATGGCCTTAGCACCTTCGTTCTATTTAAACCCACTAAAGCTAGGGCCCAAACTGGGATTAGATACCCCACTATGCCTAGCCCTAAATCCAGGTGTTTACCCTACCCTAACACCCGCCAGAGAACTACGAGCACAAACGCTTAAAACTCTAAGGACTTGG

Gavia arctica (Gaviiformes/ Gaviidae) mitogenome from CYTB to 12S rRNA (MK263210)

GO-FD in Figure 8

GAGCCTCATTCTTCTTCATCTGCATCTACCTACACATCGGACGAGGACTCTACTATGGCTCATACCTATACAAAGAGACCTGAAACACAGGAGTTATCCTCCTACTTACCCTCATAGCAACTGCCTTCGTAGGCTATGTCCTACCATGAGGCCAAATATCATTCTGAGGGGCTA
CAGTCATCACTAACCTATTCTCAGCCATCCCCTATATTGGCCAAACCCTTGTAGAGTGAGCCTGAGGCGGATTTTCAGTAGACAACCCCACATTAACCCGATTCTTTGCCCTACACTTCCTCCTCCCCTTTGTAATCACAGGCCTCGCCCTAATCCACATTACCTTCCTCCACG
AATCAGGCTCGAATAACCCCCTAGGAATCGTATCCAACTGCGATAAGATTCCCTTCCACCCCTACTTCTCCCTAAAGGATACCCTAGGCTTTATCCTCATACTCACCCCACTAATAACCCTCGCCCTATTCTCACCCAATATGCTAGGAGACCCAGAGAACTTCACCCCAGCAA
ACCCACTCGTCACACCCCCTCACATCAAACCAGAGTGATACTTCCTATTTGCATACGCTATCCTACGCTCAATCCCCAACAAACTGGGGGGAGTACTAGCCCTAGCAGCCTCCGTACTAATCCTATTCCTAAGCCCCCTCCTCCACAAATCCAAACAGCGCACAATAGCCTTTC
GCCCTCTCTCTCAACTCCTATTCTGAGCCCTAATCGCCAACCTCCTTATCCTCACCTGAGTCGGTAGCCAACCCGTAGAGCACCCCTTCATCATCATCGGCCAACTAGCCTCCCTCACCTACTTCTCCATCCTCCTAATCCTCTTCCCCATTATCGGAGCCCTAGAGAATAAAA
TACTTAACCACTAAAATACTCTAATAGTTTAACAAAAACATTGGTCTTGTAAACCAAAGAGCGAAGACTCCACCTCTTCTTAGAGTTTCCTCTACCAACCGCCCCACCTCAGAAAAAGAGGGCTTAAACCTCTATCTCCAGCTCCCAAAGCTGGTATTCTACATTAAACTACTC
TCTGACCCCCCTAAACTGCTCGAAGAGCCCCACGAGATAACCCTCGAACAAGCTCCAACACAACAAACAGAGTTAGCAGCAATCCTCACCCCGCCACCAAAAACATCCCCACCCCATACGAGTAGAACATAGCCACCCCACTAAAATCCAACCGAACAGAAAACATTCCCCCAC
CATCTACAGTAACTACCCCCAACTTCCATCACTCAACAAAGCCCCCTACCACCACCCCAACAGCAAGTACCAGAGCAAACCCCGCACCGTACCCCACAACACGCCAATCCCCTCAAGCCTCGGGAAATGGATCCGCCGCCAGAGACACAGAGTACACAAAAACCACCAGTATTC
CACCCAAATACACCATAAACAGCACCAACGCCACAAATGAGACCCCCAAACTTAACAACCACCCACAACCAACCACGGACGCTAGCACCAACCCTACCACCCCGTAGTAGGGAGAAGGATTGGACGCTACTGCCAAACCCCCCAAAGTAAAACCCACGCTCAAAAAGAGTACAA
AATAAGTCACAGTATATTCCTGCTTGGCCTCTCTCCAAGGTCTACGGCTTGAAAAGCCGCCATTGACAAATCTCAACTACAGGAACAGCTAAGATTACCCCGAATATGCTGCAAGTACATTTCCCTCATTCCCCTATATAGCGGTACCCCCCCTACCCCCCCACAGTATAAACT
GCAGGACTGTGATCGATGATCGGTGTAAGCGTATGTTTACTGTGCATTACAGCTTCTGGCGTATGCCCTGTAAGCATTACATTTACTCTATCTAGAGCATGGTCTAATGTATTATGTACAAGCTATGTATGATTTAGGATATATTATGTATGATTTACAGTCATATAATGTATG
ATTCTACGGATATAATCTGTAATGTAGATTAGACATAGTATGGAATGCTGTATGGATGGGTGAATGCAATGATAGTAAGATATACAGTGTATGTATTAGGTCTAGGCTACTCTGTATGGGTGGAATCTGCCAGAGGCCAGCTCTACTTTCAGGGTTATGTTGAGTGCAATGCCT
GAGGAGTGTGGTAGATCTGTTAGCGTACTAATACCATAGGAGGGTAGATTGTACAGTCTCTGATATACCCTACGGGCAGTGCCTGCTCTCCCTAGCATGCATGGTAACGGGTCAAACTTGCATCCTCTCTCGTTGTACCGGTAGCTTCGGATTAGGTTATCTATTGATCGGTCT
TCTCACGTGAAATCAGCAACCCGGTGTCTGAAAGATACTACGTTACTAGCGTCAGGACCATTCATTCCCCCTACACCCTAGCCCAACTTGCGCTTTTGCGCCTCTGGTTCCTCGGTCAGGGTCACACCTTGGTTCACTCCTGCGAACTTGCGCGCCACAGGGGCTATTGGTTAA
TGTGTTTCATCATGTTGGCCTCGTAATCGCGGCATCTCAAGTGTTTCGGCACATATAGTATTTCTTTTTTTGGGGCTTCTTCAGGCTGCCCTTCCAGTGCGGCGGGTAAATACAATCTATAGACGTGAGCATACAATGACCGGCGGTCTGTTTTTTGGCCCTCAAGAATAACTG
GATGAGACGGTTGGCGTATATGGGGAATCATCTTTACTCTGATGCACTTTGTTTTCCATTTGGTTATGGTGTATCCACAGACTATTAAGCTATGTTGCTATTTAGTGAATGCTTGTTGGACATAATTTCTTACTTTTACACTTCCTCTAACTTTCTAAACAACACTAGGAATTT
TCAACTAAAATATCATCGATGTCATCCATCATAATTTATCGCGTTATGATGACGTGTGATGACATATTTTATTCATCATCATTTTTTATGTCATCAACGCTGGAGTTACATTAATAAATTCAATCATTATTTTCGTCACTTTTTTTCGTATCTTCCGCCCATATGCCTTTAAAA
CCAGGCTCGAATAACCCCCTAGGAATCGTATCCAACTGCGATAAGATTCCCTTCCACCCCTACTTCTCCCTAAAGGATACCCTAGGCTTTATCCTCATACTCACCCCACTAATAACCCTCGCCCTATTCTCACCCAATATGCTAGGAGACCCAGAGAACTTCACCCCAGCAAAC
CCACTCGTCACACCCCCTCACATCAAACCAGAGTGATACTTCCTATTTGCATACGCTATCCTACGCTCAATCCCCAACAAACTGGGGGGAGTACTAGCCCTAGCAGCCTCCGTACTAATCCTATTCCTAAGCCCCCTCCTCCACAAATCCAAACAGCGCACAATAGCCTTTCGC
CCTCTCTCTCAACTCCTATTCTGAGCCCTAATCGCCAACCTCCTTATCCTCACCTGAGTCGGTAGCCAACCCGTAGAGCACCCCTTCATCATCATCGGCCAACTAGCCTCCCTCACCTACTTCTCCATCCTCCTAATCCTCTTCCCCATTATCGGAGCCCTAGAGAATAAAATA
CTTAACCACTAAAATACTCTAATAGTTTAACAAAAACATTGGTCTTGTAAACCAAAGAGCGAAGACTCCACCTCTTCTTAGAGTTTCCTCTACCAACCGCCCCACCTCAGAAAAAGAGGGCTTAAACCTCTATCTCCAGCTCCCAAAGCTGGTATTCTACATTAAACTACTCTC
TGACCCCCCTAAACTGCTCGAAGAGCCCCACGAGATAACCCTCGAACAAGCTCCAACACAACAAACAGAGTTAGCAGCAATCCTCACCCCGCCACCAAAAACATCCCCACCCCATACGAGTAGAACATAGCCACCCCACTAAAATCCAACCGAACAGAAAACATTCCCCCACCA
TCTACAGTAACTACCCCCAACTTCCATCACTCAACAAAGCCCCCTACCACCACCCCAACAGCAAGTACCAGAGCAAACCCCGCACCGTACCCCACAACACGCCAATCCCCTCAAGCCTCGGGAAATGGATCCGCCGCCAGAGACACAGAGTACACAAAAACCACCAGTATTCCA
CCCAAATACACCATAAACAGCACCAACGCCACAAATGAGACCCCCAAACTTAACAACCACCCACAACCAACCACGGACGCTAGCACCAACCCTACCACCCCGTAGTAGGGAGAAGGATTGGACGCTACTGCCAAACCCCCCAAAGTAAAACCCACGCTCAAAAAGAGTACAAAA
TAAGTCACAGTATATTCCTGCTTGGCCTCTCTCCAAGGTCTACGGCTTGAAAAGCCGCCATTGACAAATCTCAACTACAGGAACAGCTAAGATTACCCCGAATATGCTGCAAGTACATTTCCCTCATTCCCCTATATAGCGGTACCCCCCCTACCCCCCCACAGTATAAACTGC
AGGACTGTGATCGATGTAAGTACAAACTCTTCTGAATTCTAGACCCTAAGCTTATGTGGGTGTGCATTGCTTGTATCCCCACAATGATGAGGGATGTACTACTTCTCTTATCCAAACATTAAGTGTATATCTAGGCCTACAAAGCTACTGCACACTTACAATGAACGGAACAGG
TTACTGAGCGTATGTAAATAATACATATAATGTATGTACTAGGTCTATATGGTTCATGTACTTTAGTCATGCTTTTGCATGTATAGGGGTTATATAGTGTATGGATTCTTGGATACTTAGATGTATGTACTGGGTTTAAGCAGTCCATGTACTCTAGCCATGTCAGTGCATGCT
TGAGGATTATACAGTGTATGTATTAGGTCTAGGCTACTCTGTATGGGTGGAATCTGCCAGAGGCCAGCTCTACTTTCAGGGTTATGTTGAGTGCAATGCCTGAGGAGTGTGGTAGATCTGTTAGCGTACTAATACCATACGGAGATAAGCTGTGCAGTCTCTGATATACCCTAC
GGGCAGTGCCTGCTCTCCCTAGCATGCATGGTAACGGGTCAAACTTGCATCCTCTCTCGTTGTACCGGTAGCTTCGGATTAGGTTATCTATTGATCGGTCTTCTCACGTGAAATCAGCAACCCGGTGTCTGAAAGATACTACGTTACTAGCGTCAGGACCATTCATTCCCCCTA
CACCCTAGCCCAACTTGCGCTTTTGCGCCTCTGGTTCCTCGGTCAGGGTCACACCTTGGTTCACTCCTGCGAACTTGCGCGCCACAGGGGCTATTGGTTAATGTGTTTCATCATGTTGGCCTCGTAATCGCGGCATCTCAAGTGTTTCGGCACATATAGTATTTCTTTTTTTGG
GGCTTCTTCAGGCTGCCCTTCCAGTGCGGCGGGTAAATACAATCTATAGACGTGAGCATACAATGACCGGCGGTCTGTTTTTTGGCCCTCAAGAATAACTGGATGAGACGGTTGGCGTATATGGGGAATCATCTTTACTCTGATGCACTTTGTTTTCCATTTGGTTATGGTGTA
TCCACAGACTATTAAGCTATGTTGCTATTTAGTGAATGCTTGTTGGACATAATTTCTTACTTTTACACTTCCTCTAACTTTCTAAACAACACTAGGAATTTTCAACTAAAATATCATCGATGTCATCCATCATAATTTATCGCGTTATGATGACGTGTGATGACATATTTTATT
CATCATCATTTTTTATGTCATCAACGCTGGAGTTACATTAATAAATTCAATCATTATTTTCGTCACTTTTTTTCGTATCTTCCGCCCATATTCTACTAAAAACAACACACAAACGACCCACAAAAATCAATCAACGTATTAACAGTCAATCAATCAACGTATTAACAGTCAATC
AATCAACGTATTAACAGTCAATCAATCAACGTATTAACAGTCAATCAATCAACGTATTAACAGTCAATCAATCAACGTATTAACAGTCAATCAATCAACGTATTAACAGTCAATCAATCAACGTATTAACAGTCAATCAATCAACGTATTAACAGTCAATCAATCAACGTATTA
ACAGTCAATCAATCAACGTATTAACAGTCAATCAATCAACGTATTAACAGTCAATCAATCAACGTATTAACAGTCAATCAATCAACGTATTAACAGTCAATCAATCAACGTATTAACAGTCAATCAATCAACGTATTAACAGTCAATCAATCAACGTATTAACAGTCAATCAAT
CGACATATAACAGTCAATCAATCAACGTATTAACAGTCAATCAATCGACATATAACAGTCAATCAACATACA**GTCCCTGTAGCTTATAGTAAAGCATGGCACTGAAGATGCCAAGACGGTTGCCTTCATCATACCCAGGGACA**AAAGACTTAGTCCTAACCTTACAGTTAATTC
TTGCTAAACATATACATGCAAGTATCCGCGCACCAGTGTAAATGCCCTTAATCTCTTGCTTGCAAGACAAAGGAGCGGGTATCAGGCACACCTATGATTGAATCGTAGCCCAAGACGCCTTGCTTAGCCACGCCCCCACGGGTATTCAGCAGTAGTTAACATTAAGCAATGAGT
GTAAACTTGACTTAGTTATAGCAGCACTTAGGGTCGGTAAATCTTGTGCCAGCCACCGCGGTCACACAAGAGGCCCAAATTAACCGTAATACGGCGTAAAGAGTGGTACCATGTTATCCCGTCAACTAAGATCAAAGTGCAACTGAGCTGTCGTAAGCCC

Gavia stellata (Gaviiformes/Gaviidae) mitogenome from CYTB to 12S rRNA (MK263209)

GO-FD in Figure 8

[illegible]

Podiceps cristatus (Podicipediformes/ Podicipedidae) mitogenome from Cytb to CR2 (MN629890)

GO-FD in Figure 8

[illegible]

Podiceps grisegena (Podicipediformes/Podicipedidae) mitogenome from ND6-1 to CR2 (MK263194)

GO-FD in Figure 8

[illegible]

Scopus umbretta (Pelecaniformes/Scopidae) mitogenome from CR1 to CR2 (MW151828)

GO-NG in Figure 8

[illegible]

AATCCTCTCTCGACGTGCCGGTAGCTGTCGGACCAGGTTATCTATTAGTCGGTCTCCTCACGTGAAATCAGCAACCCGCCGCATATAAGATACTGCGTTACTAGCTTCAGGACCATTCTTTCCCCCTAAACCCCTAGCACAACTTGCACTTTTGCGCCTCTGGTTCCTTTTTCA
GGGCCATCACCCGTTTCCTCCCCTCTACTCGCTCTTCACCGATACATCTGGTTGGCTATATATCAC

Spheniscus demersus (Sphenisciformes/Spheniscidae) mitogenome from CR1 to CR2 (MH264510)

GO-FD in Figure 8

ATACTATTGGTTGACATGAGCATCACATGGGTCACGGTCGTTCCTAGCCTTCAAGAATACCTGAATGAGACGGTTTGAGGTATTAGGGGAATCATCTTAACACTGATGCACTTTGGTTTGCATTTAGTTATGGTGTATTTACTTGCTTTTAGATATGTTGCTATTTGTTGAATG
CTTGTTAGACATAATTCCTTATTATTACACTTCCTCTAATTTCCTAAACAACACTAGGCAATTTTAACTAAACCTTAACCAAATTTTTAGATCACCAAAATCACATATCATGTCGATACACTTTACACTTCTTCTTTCTCTAAAATTTCATTAACACACGATAGCAACTCTTTG
TTGCCATCACTTACTGTATCGATACACTTTACATTTCTTCTTTCTCTAAAATTTCATTAACACATGATAGCAACTCTTTGTTGCCATCACTTACTGTATCGATACACTTTACATTTCTTCTTTCTCTAAAATTTCATTAACACATGATAGCAACTCTTTGTTGCCATCACTTAC
TGTATCGATACACTTTACATTTCTTCTTTCTCTAAAATTTCATTAACACATGATAGCAACTCTTTGTTGCCATCACTTACTGTATCGATACACTTTACATTTCTTCTTTCTCTAAAATTTCATTAACACATGATAGCAACTCTTTGTTGCCATCACTTACTGTATCGATACACT
TTACATTTCTTCTTTCTCTAAAATTTCATTAACACATGATAGCAACTCTTTGTTGCCATCACTTACTGTATCGATACACTTTACACTTCTTCTTTCTCTAAAATTTCATTAACACATGATAGCAACTCTTTGTTGCCATCACTTACTGTATCGATACACTTTACATTTCTTCTT
TCTCTAAAATTTCATTAACACACGATAGCAACTCTTTGTTGCCATCACTTACTGTATCGATACACTTTACATTTCTTCTTTCTCTAAAATTTCATTAACACACGATAGCAACTCTTTGTTGCCATCACTTACTGTATCGATACACTTTACATTTCTTCTTTCTCTAAAATTTCA
TTAACACATGATAGCAACTCTTTGTTGCCATCACTTACTGTATCGATACACTTTACATTTCTTCTTTCTCTAAAATTTCATTAACACACGATAGCAACTCTTCGCCACAAAAGATATTCTAGGATTTATACTCCTACTCCTTCCACTAACAACCTTAGCCCTATTCTCACCCAA
CCTATTAGGAGACCCAGAAAACTTCACCCCAGCAAACCCCCTAGTCACACCCCCACACATTAAACCAGAATGATACTTCCTATTTGCCTACGCCATCCTACGCTCAATCCCCAACAAACTAGGAGGAGTACTAGCCCTAGCAGCATCCGTACTAATCCTATTCCTAATACCCCT
CCTCCACAAATCTAAACAGCGCACAATAGCCTTCCGTCCACTCTCCCAACTCCTGTTCTGAACCCTAGTAGCCAACCTCATTATCCTAACGTGAATTGGCAGCCAACCAGTAGAACATCCCTTCATTGCCATCGGCCAATTAGCCTCCTTCACCTACTTCACCACCCTCCTAAT
TCTCTTTCCCCTTGCCGGAGCCCTAGAAAACAAAATACTCAATCACTAAAAATACTCTAATAGTTTATAAAAACATTGGCCTTGTAAGCCAAAGAATGAAGGCTACACCCCTTCTTAGAGTTTACCCTAACCTTCAGAGAAAGAGGACTCAAACCTCCATCCCCAACTCCCAAA
GCTGGCATTTTAAATTAAACTATTCTCTGACAACCCTCAACCCTAAACAGCACGAATTGCCCCACGAGACAGCCCCCGTACAAGCTCCAACACAACAAACAACGTTAACAACAACCCTCACCCCGCCACTAAAAACATTCCCACCCCACACGAATAAAACAAAGCTACCCCACT
AAAATCTAACCGAACAGACACCACCCCTCCACTATCAACAGTATCCACTCCAAACTTTCACCCCTCAAACCCCCCAACTCCCAACCCAACAGCAAGGACCAAAACAAGACCCACACCATATACCACAACAGGTCACTCTCCCCAACCCTCAGGAAAGGGGTCCGCGGCCAAAGC
CACAGAGTACACAAAAACCACCAACATTCCCCCCAAATACACCATAAATAAAACCAACGATACAAAAGAAACCCCCAAACTTAACAACCACCCGCACCCTACAACAGAAGCCACCACCAAACCAACCACCCCATAATAAGGCGAAGGATTAGATGCAACAGCTAATCCCCCCAA
AACAAAACACATCCCCAAAAATAACACGAGATAAGTCATAGCAGTTCCTGCTTGGCTTTTCTCCAAGACCTACGGCCTGAAAAGCCGCCGTTGCTAATTCAACCACAGGAACAGTTATAAAAGATACCCTCTACCTCCCCCCCCCTACCCCCCCATGTTATATGTTCACTTATG
TATGAAATACATTATATTGAATGTCAGAAGCTACATTCCAGTGTATGTACTATGATTGGTTTAGAATAATTGGATGGGGTATTCTTGAGAACTGCTGTGTTTCAAGGATTAATGGAGTAATACTTTCAAGGATTAATTGAGTAATGGAATAGGGATTAAGTTAATTCTCTTTTC
GTGTTAAACGGGGTTTGTGCGATGAGTTTGGGGAATTATCATGTTATGGTTGTGATGATTATCTTCTGTCCCGTTGAAGGGCTAAATCCATGATTTTGATAGTCTGTATCCTTCTAGCAGGAATGATACGGGCGTGTTTTATTGCTTGGTTTTAATGGAGAATGTGCTTATTAT
CCAATAACTTCTTGGGTGCCAGCGTCAGGGATTAGGTTATCTATTAATTTGGCTTCTCACGTGAAATCAGCAACTCGACGTATGGAAGATCCCGTGCTACTAGCGTCAGGCCCATTCTTTCCCCCTACACCCTAGCCCAACTTGCTCTTTTGCGCCTCTGGTTCCTCGGTCAGG
GCCATAACTTGAATAACTCCTTCTATCTTGCTCTTCACAGAGCCATTTGGTTGATGTGTTTCATCATTTTCGTC

Uria aalge (Charadriiformes/Alcidae) mitogenome from CR1 to CR2 (MK263188)

GO-FD in Figure 8

TAACTTGGTTAGTCCTCTCAACTTGTACTTCACCGATACATCTGGTCGGCTATATATCACCATCTCACCCGTGATCGCGACATCCGACCGTTTTGGCACTTTTGGTTCCTTTTTTTTTTTTTTTACGTCTTCAATAAACCCTTCCAGTGCACCGAGGTACACACAATCTAAGACCT
GAGCTTTCCCTGGTATTCGTACGGATTTTGGCCCTCAGGAATACCTGAATGTCAAGGTTTAACGGGTTGGGGGAATCATTTTTACACTGATGCACTTTGCTTTGCACTTGGTTATGGGATCTCCGCAAGCTCTTATTTATGTTGTTATTTAGTGAATGCTTGTTGGGCATATTT
TATTATTTTTCATTTCCTCTAACTTTTTAAACAACACTAGCAAATTTTATTCAAAAACAAACTGTGATTTTCATCACACATTTTGTCATCGTCATCACACATTTATCATCACACTTATCATCTACAAACGGCACTGGAATTCCATTAAAAAAAGGACTAATTTAATCCGTCAC
ATTTTATCTTACCTTCCTCCATGAATCCGGATCAAACAACCCCCTAGGAATCCCATCCAACTGCGATAAAATCCCATTTCACCCTTACTTCTCACTAAAAGACATCCTAGGATTCATTATCATATTCCTTCCACTAACAACCCTAGCCCTATTCTCCCCCAACCTACTAGGCGA
CCCAGAGAATTTTACCCCCGCAAACCCACTAGTTACACCTCCCCATATCAAACCAGAATGATACTTCCTATTTGCATACGCTATCCTGCGATCAATCCCCAATAAACTTGGAGGTGTACTCGCCCTAGCAGCTTCCGTACTAGTACTATTCTTGACCCCTCTACTCCACAAATC
TAAGCAGCGCGCCATAACCTTTCGCCCACTCTCACAACTCCTATTCTGAACCCTAGTCGCCAACCTCCTCATTCTAACATGAGTAGGTAGCCAACCCGTAGAACATCCATTCATCATCATTGGACAACTAGCCTCCCTTACCTACTTCACCATTCTACTCCTCCTATTCCCCAT
CATCGGAGCCCTAGAGAATAAAATACTTAACTACTAAAAACACTCTAATAGTTTATAAAAAACATTAGTCTTGTAAACCAAAGACTGAAGACTACACCCCTTCTTAGAGTTTCCACTTACCCTATCTCAGAAAAAGAGGACTTAAACCTCTATCTCCAACTCCCAAAGCTGGTA
TTTTGTACTAAACTATTCTCTGACCCTCCCCCTAAACCGCTCGAATGGCCCCACGAGTTAATCCACGTACTAACTCTAATACAACAAACAAAGTCAACAACAACCCTCAACCCGCCGCCAAAAACATTCCAACGCCACAAGAATAAAACATTGCCACCCCACCAAAATCTAACC
GAACAGAGAACATACCTACACTGTCAACAGTAACTATCCCCAAATCCCAGCACTCAAACCCACCAACAACCACCCCCGCCACAAGTACTGCAACAAAACCCATCCCATACCCAACAACCCGTCAATCCCCTCAAGCTTCAGGAAAAGGATCCGCCGCCAAGGACACAGAATAAA
CAAAAACTACCAACATTCCCCCTAAGTACACCATAAACAGTACCAAGGACACAAAGGAAATACCTAAACTCAACAACCACCCGCACCCCGCAACAGAGGCCAACACCAAACCAACCACCCCATAATATGGAGAAGGATTAGATGCAACTGCCAAACCCCCTAACACAAAACATA
GACCTAAAAAAAGCACAAAATAAGTCATAGCAGTTTCTGCTTGGCTTTTCTCCAAGACCCGCGGCCCGAAAAGCCACTGTTGTACCACTTCAACTACAGAAACTAGAAATTGTACTGGTACCCCCCCCCTCCCCCCCATATAGCAAGCCAAATCGCCATGTGGGAGTACACATT
AAACTACCAGCCCCATAAGCACATACACTCCATGTTCCAAAACCATTAATATACACACAGACATAACCCTGAATTTTCCACATTTCTCCTTCAGAGGACCTCCCAGCCCAATGGATCCGAATTCCATTACAATATCCGTACTAATACCATCTCCTCTCCAGTCTTTACATACCA
ACTCCGACAAGATACGACAGTGCCTGTCTACACCTTTGTAATGGGGTAAAGGCATGGCCCTCCAAAACTTCTCGAATACACAAAGCTTCGTGCCAGGTTATTTATTAATCGAGCTCCTCACGTGAAATCAGCAACCCGGTGTTTGAAAGATCCTACGCTACCAGCTTCAGGAC

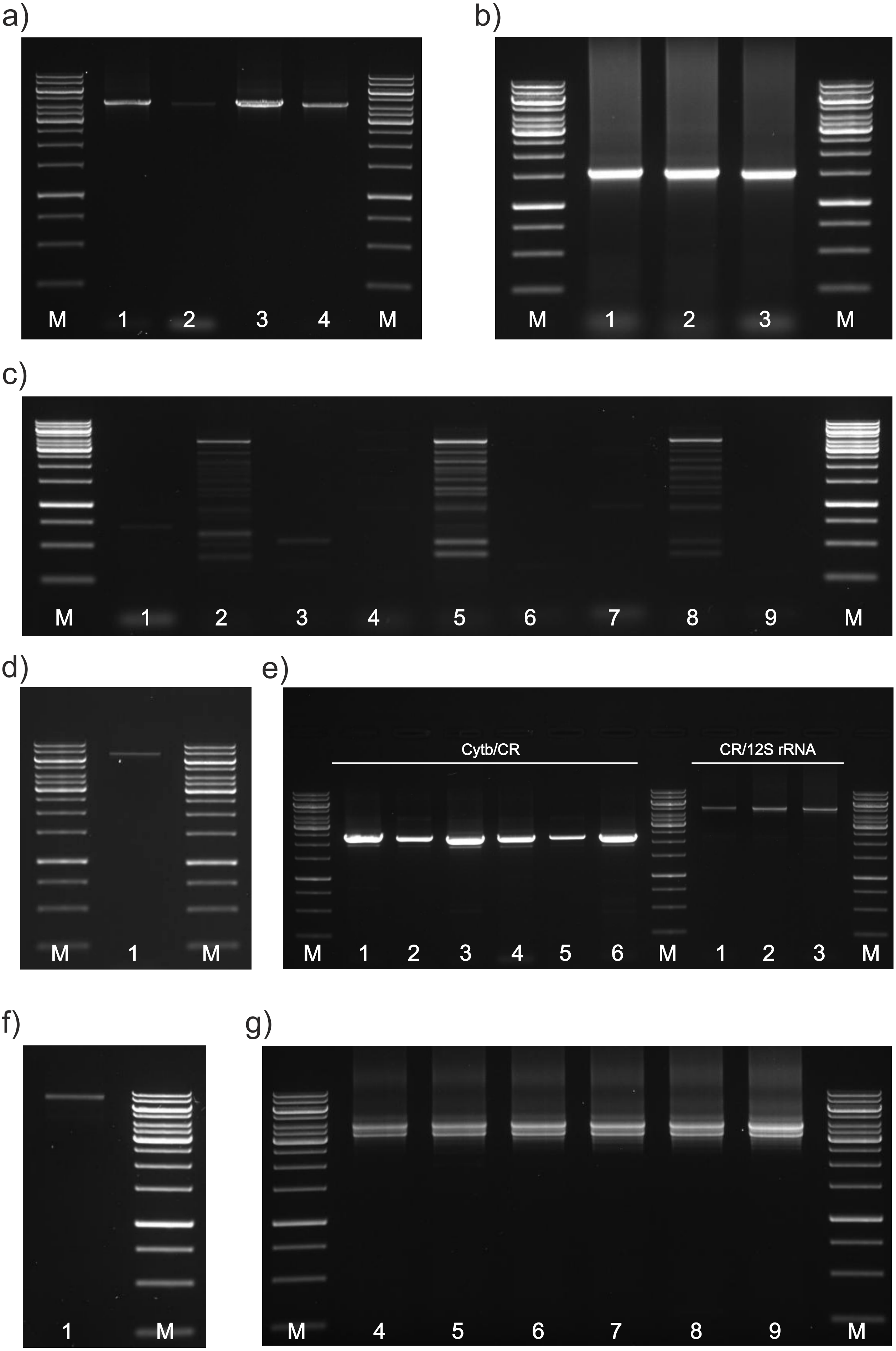

Figure S11. Representative cropped agarose gels of the following amplicons: *cytb*/12S rRNA obtained *for Rhynochetos jubatus* (a), *Eurypyga helias* (d) and *Trogon collaris* (f); CR1/CR2 obtained for *Rhynochetos jubatus* (c) and *Trogon collaris* (g); CR/12S rRNA obtained for *Rhynochetos jubatus* (b) and *Eurypyga helias* (e); *cytb*/CR obtained for *Eurypyga helias* (e). The numbering of amplicons separated in the agarose gel corresponds to the reaction numbered in Table S9. Lane M - GeneRuler 1 kb DNA Ladder (Thermo Scientific).

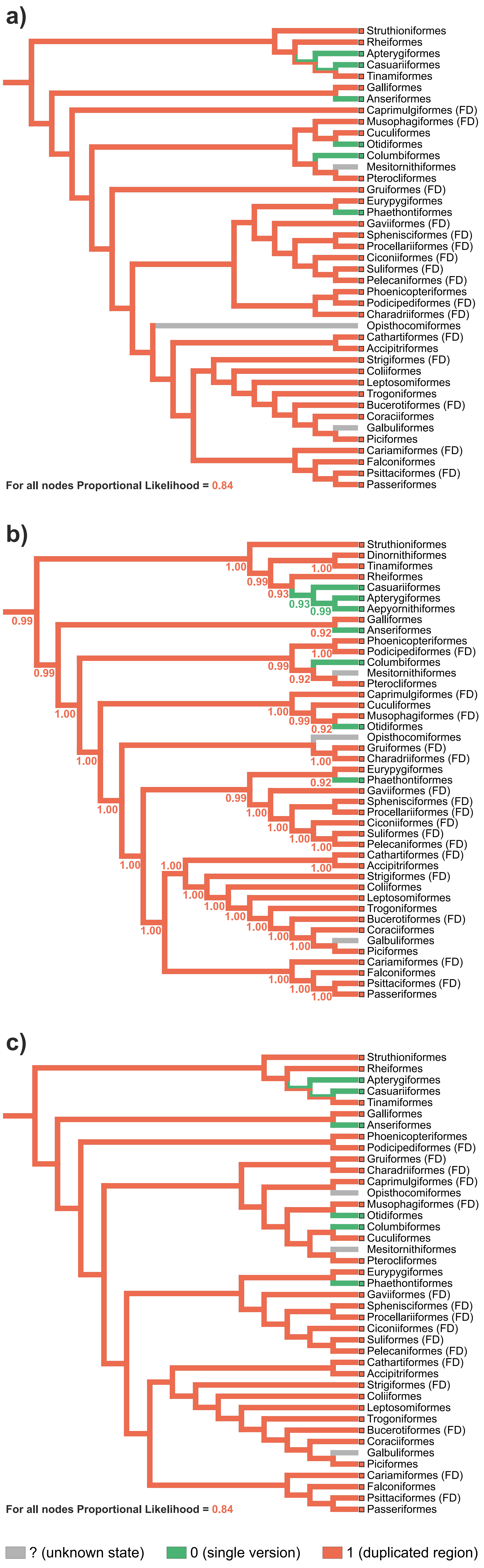

Figure S12. Reconstruction of ancestral states and mapping of mitogenomic duplications onto the Aves three tree topologies under a more liberal assumption for assignment of duplication states. The colored thick lines of the trees correspond to maximum parsimony reconstruction, whereas values at nodes mean the probability of a more likely ancestral state (proportional likelihood) provided by maximum likelihood reconstruction. The trees obtained by Prum, et al. [45] are in a, by Kimball, et al. [47] in b, whereas by Kuhl, et al. [120] in c. The duplication state was assumed for a given order if a duplication was reported in at least one species from this order. FD indicates the presence of the most fully duplicated avian region. In ML approach, AsymmMk model was applied for a and, c whereas Mk1 for b.

Figure S13. Original uncropped and unprocessed agarose gels of: CR1/CR2 amplicons obtained for *Struthio camelus* (a), which were also shown in the Fig. S1a; CR1/CR2 amplicons obtained for *Rhea pennata* (b) which were also shown in the Fig. S1b; CR1/CR2 amplicons obtained for *Rhea americana* (7 lanes to the left of DNA Ladder) (c), which were also shown in the Fig. S1c; CR1/CR2 amplicons obtained for *Crypturellus tataupa* (6 lanes to the right of the DNA Ladder) (d), which were also shown in the Fig. S1d; potential NUMTs amplified for *Dromaius novaehollandiae* (e), which were also shown in the Fig. S4a; potential NUMTs amplified for *Casuarius casuarius* (f), which were also shown in the Fig. S4b; *cytb*/*12S rRNA* amplicons obtained for *Rhynochetos jubatus* (g), which were shown in the Fig. S11a; CR/*12S rRNA* amplicons obtained for *Rhynochetos jubatus* (h), which were shown in the Fig. S11b; CR1/CR2 amplicons (potential NUMTs) obtained for *Rhynochetos jubatus* (i), which were shown in the Fig. S11c; *cytb*/*12S rRNA* amplicon obtained for *Eurypyga helias* (j), which was shown in the Fig. S11d; *cytb*/CR and CR/*12S rRNA* amplicons obtained for *Eurypyga helias* (k), which were shown in the Fig. S11e; *cytb*/*12S rRNA* amplicon obtained for *Trogon collaris* (the first lane prior the first DNA Ladder) (l), which was shown in the Fig. S11f; CR1/CR2 amplicons obtained for *Trogon collaris* (m), which were shown in the Fig. S11g.

a)

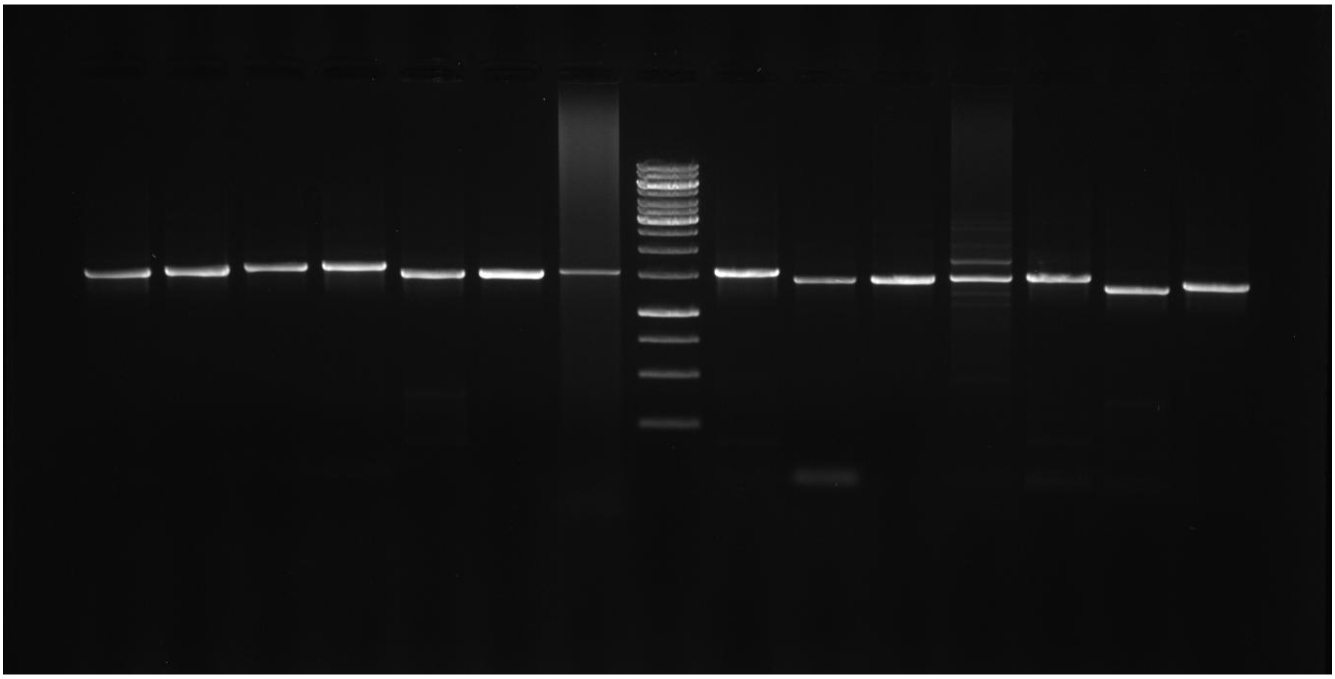

b)

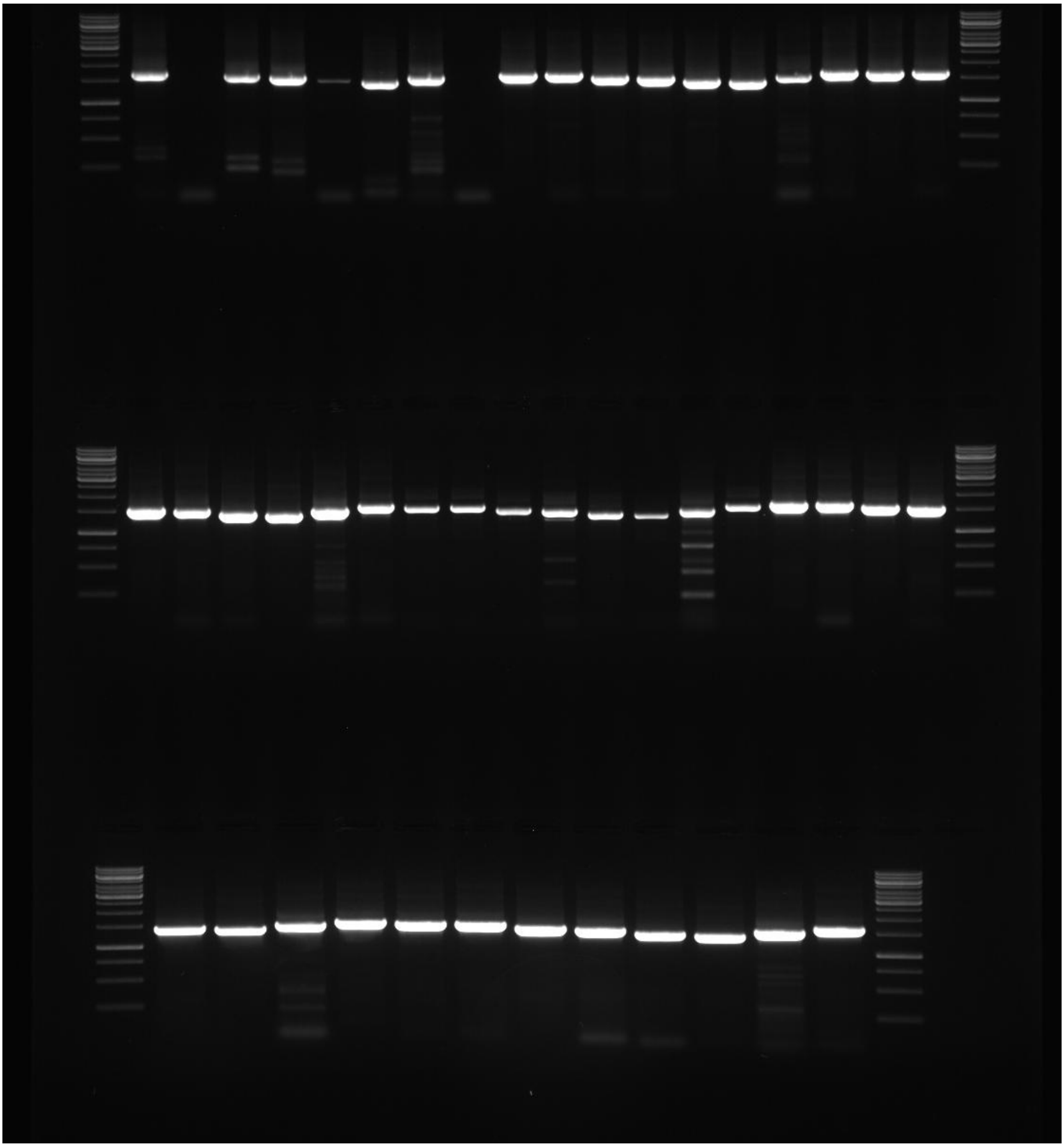

c)

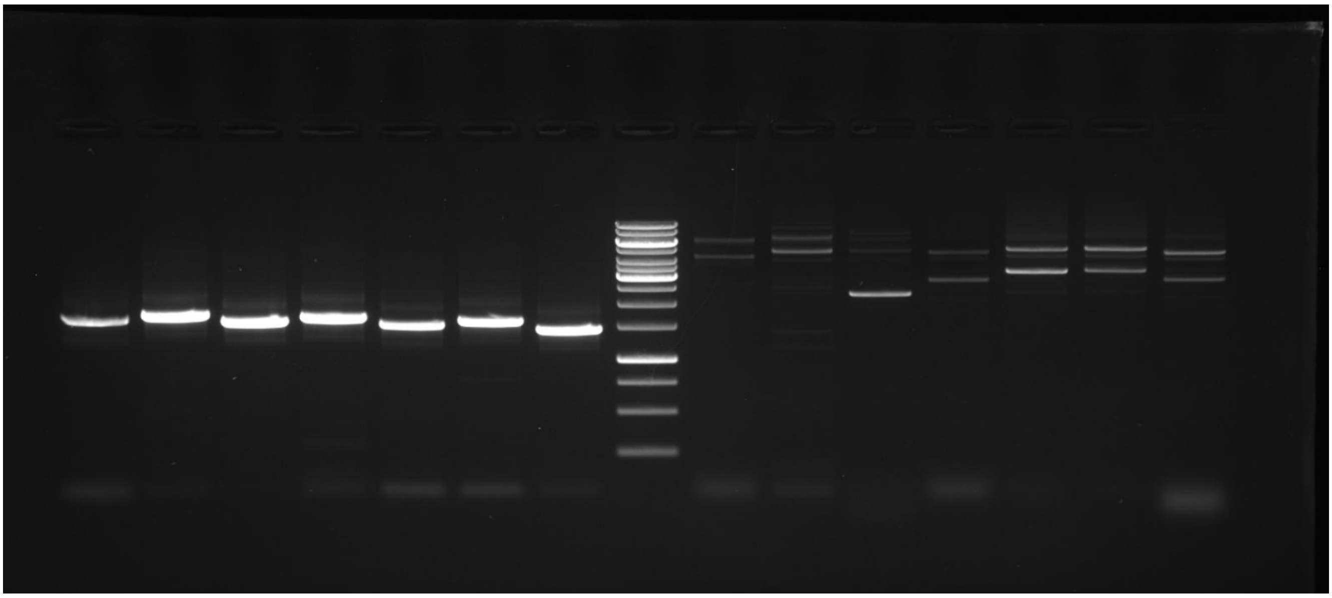

d)

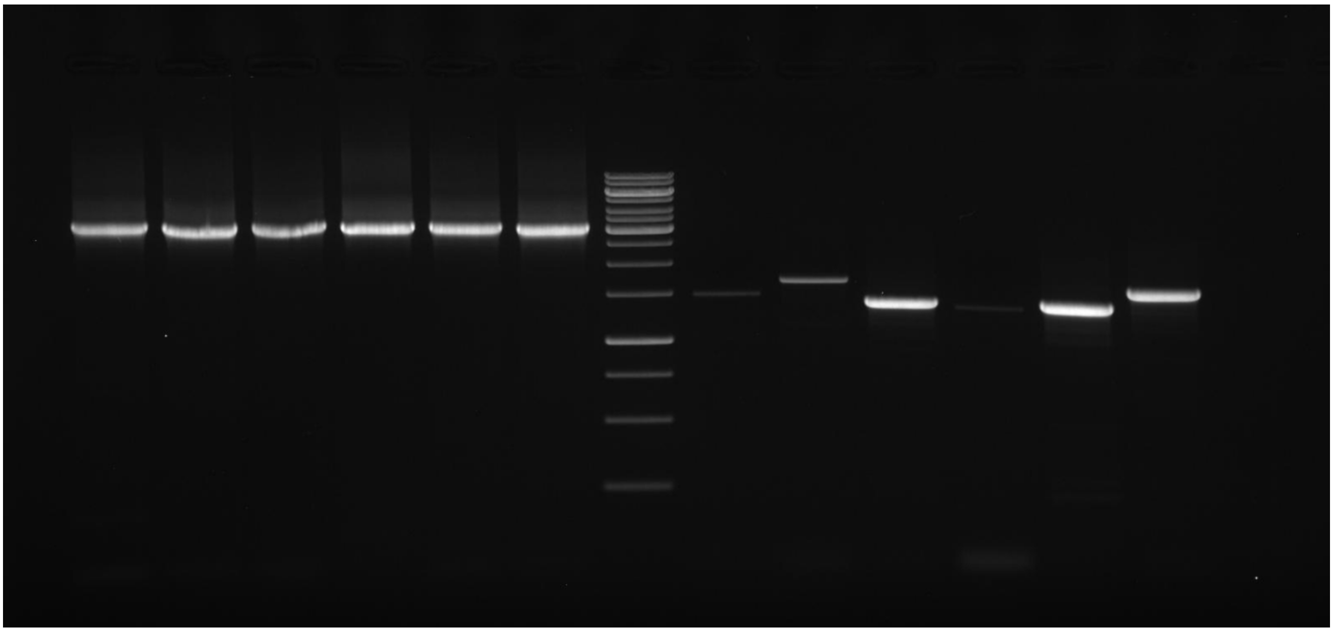

e)

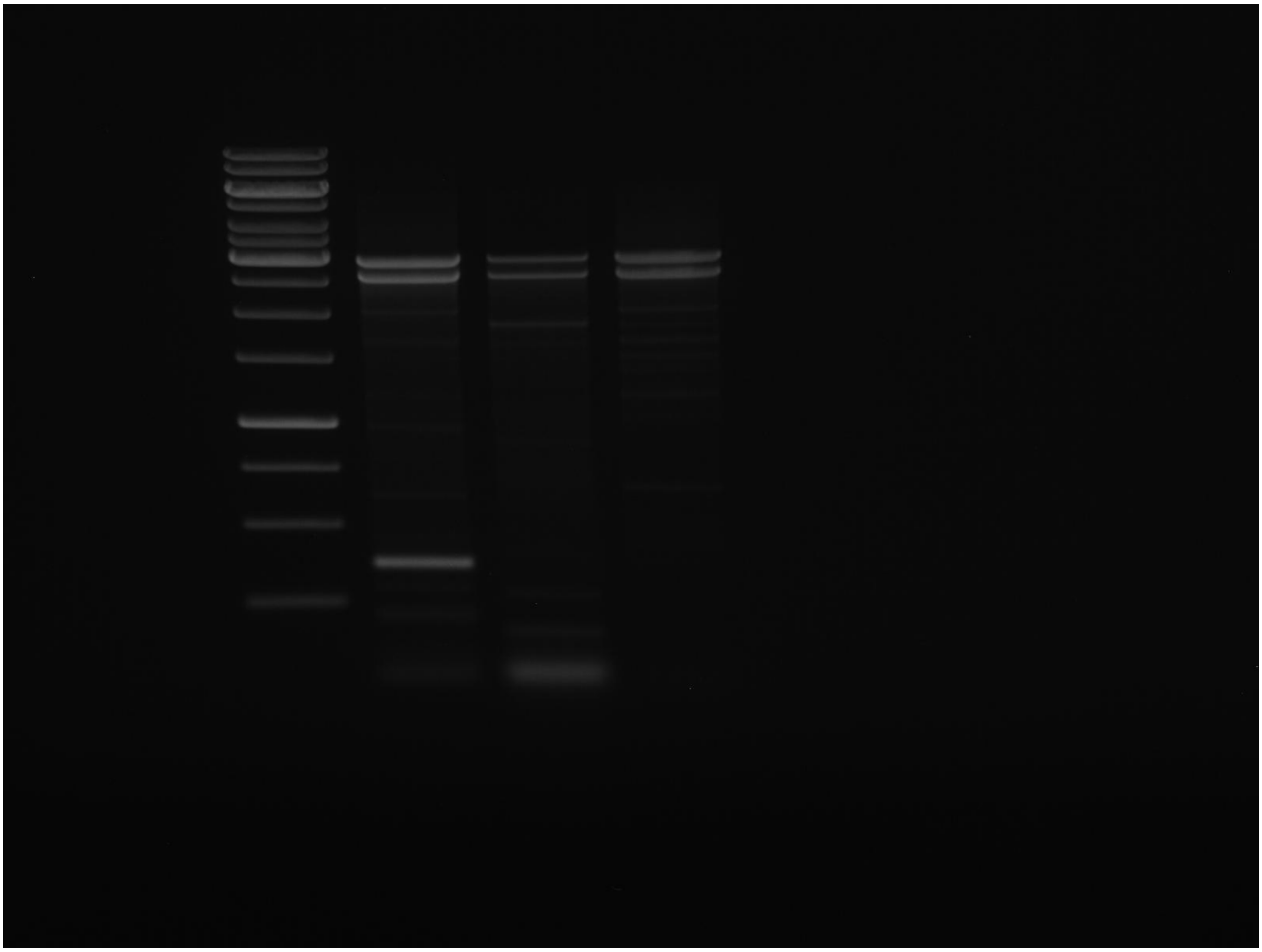

f)

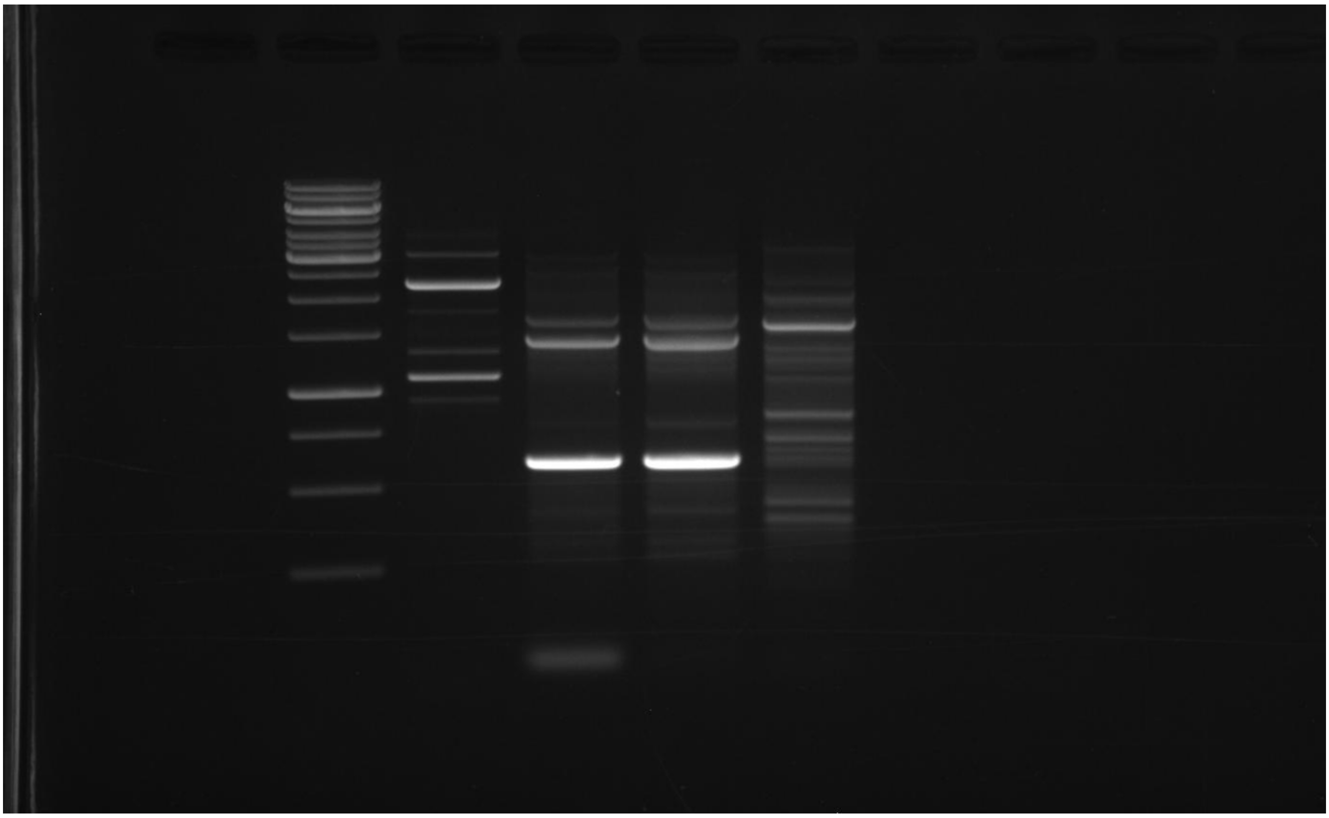

g)

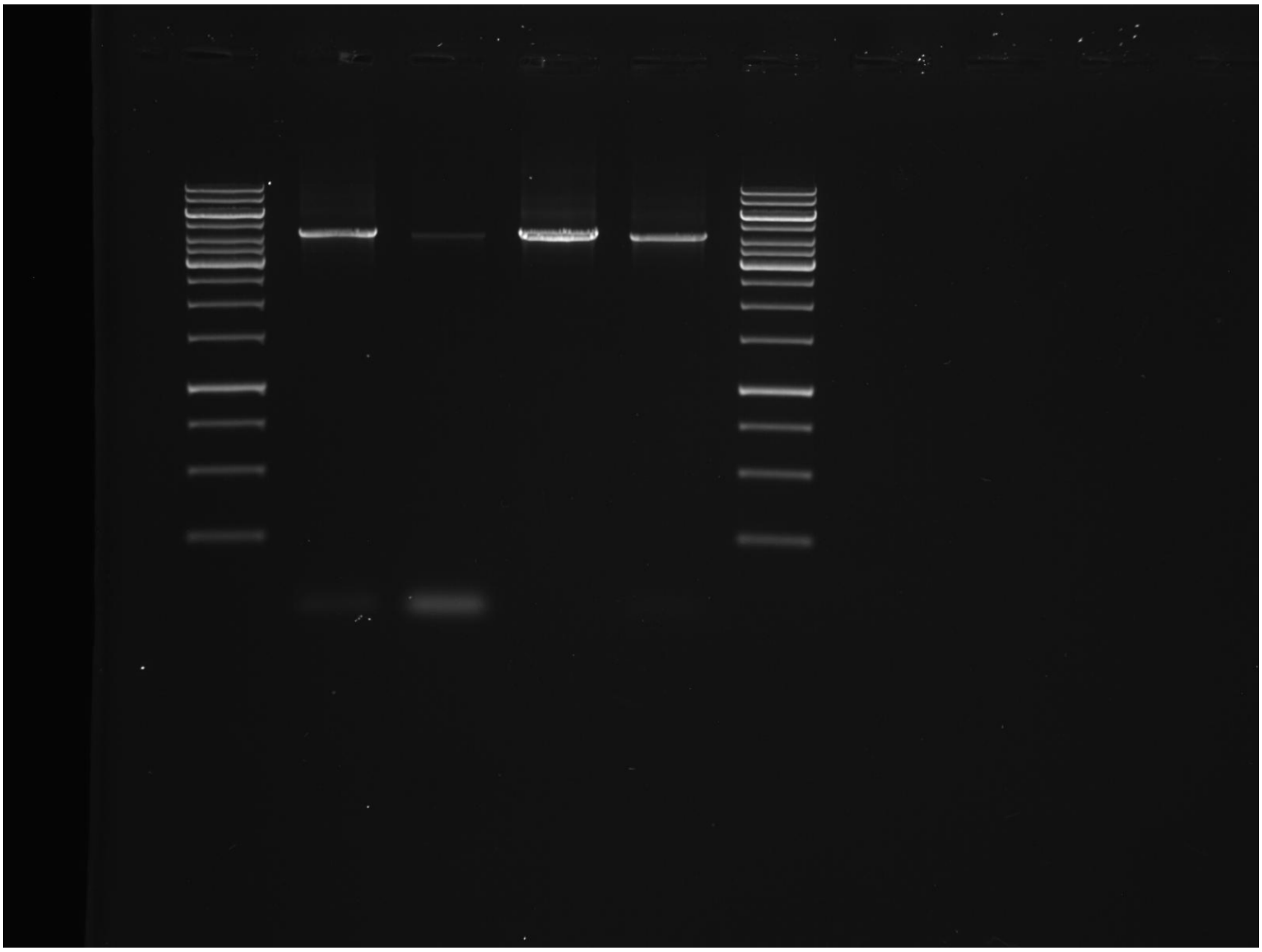

h)

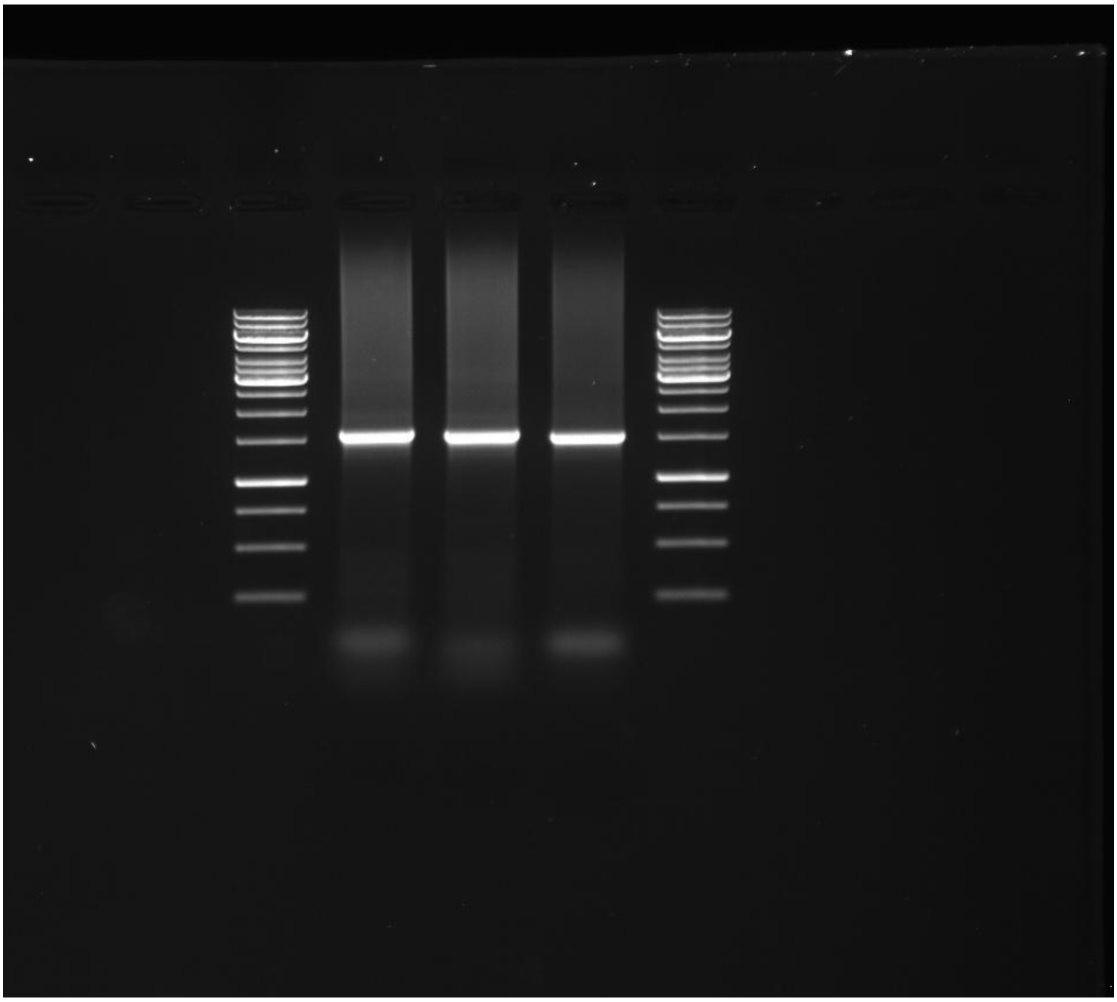

i)

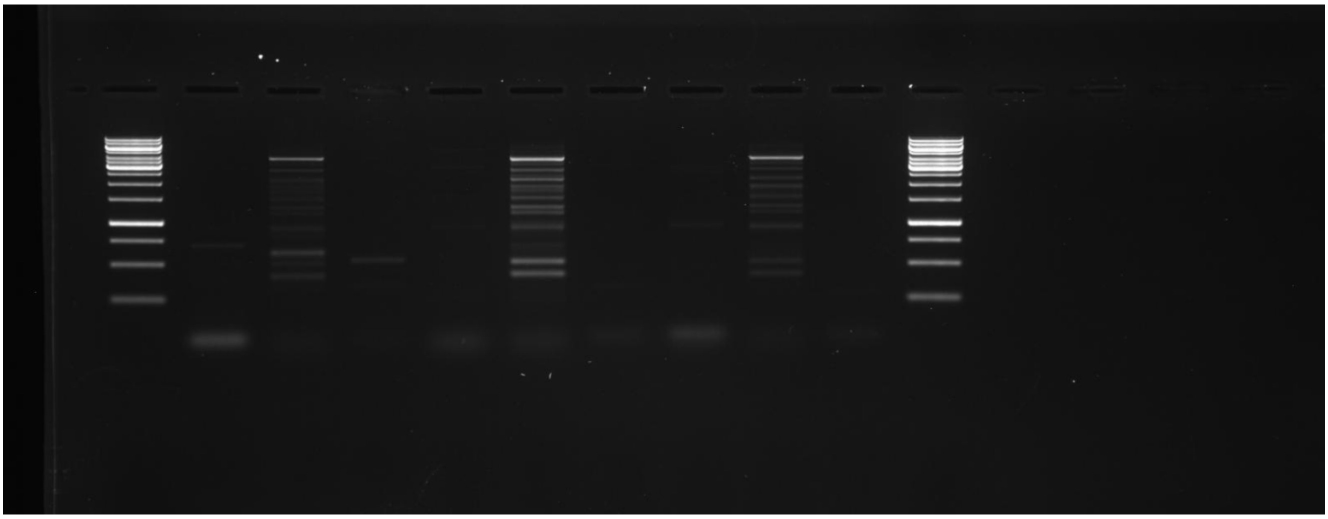

j)

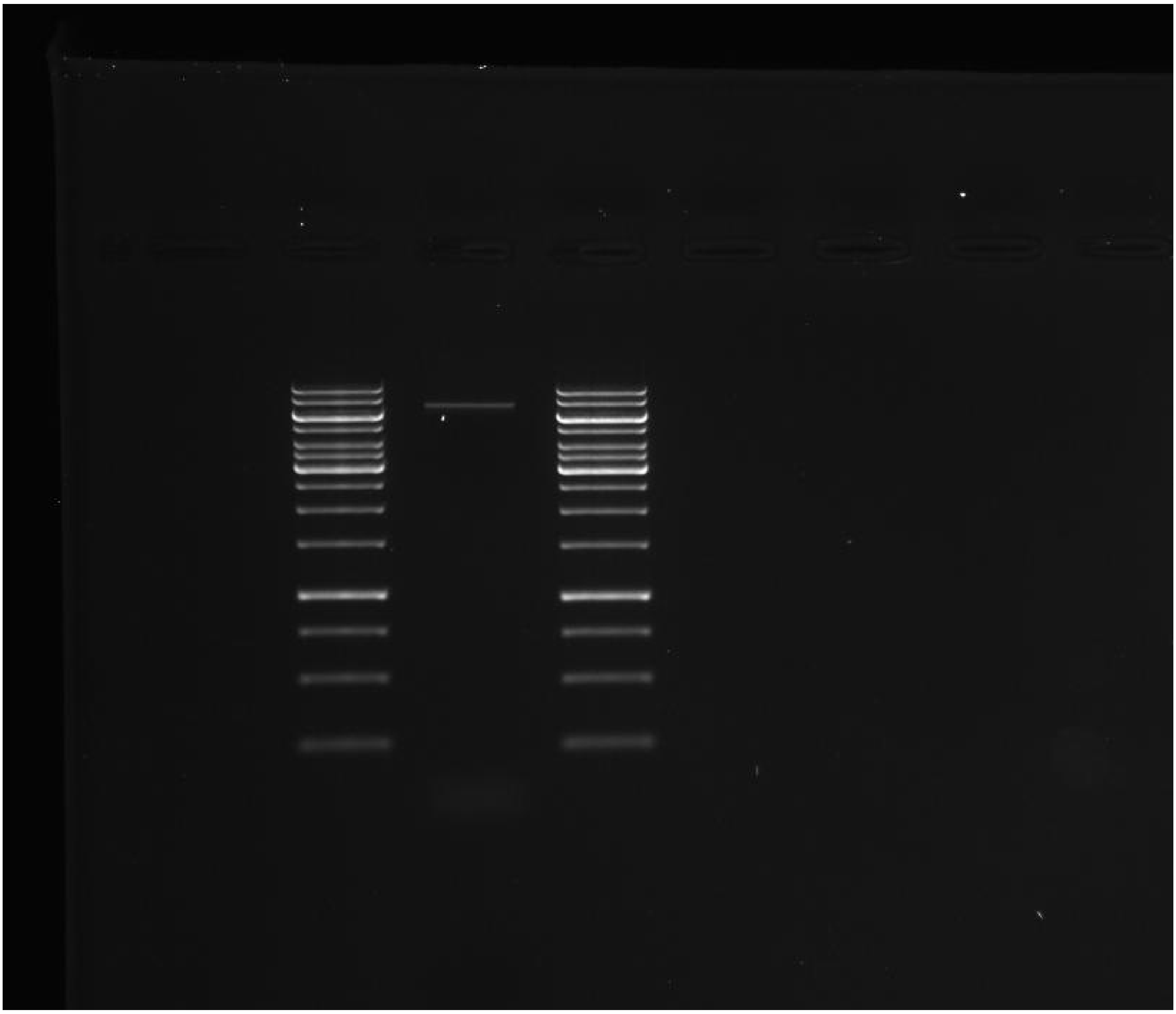

k}

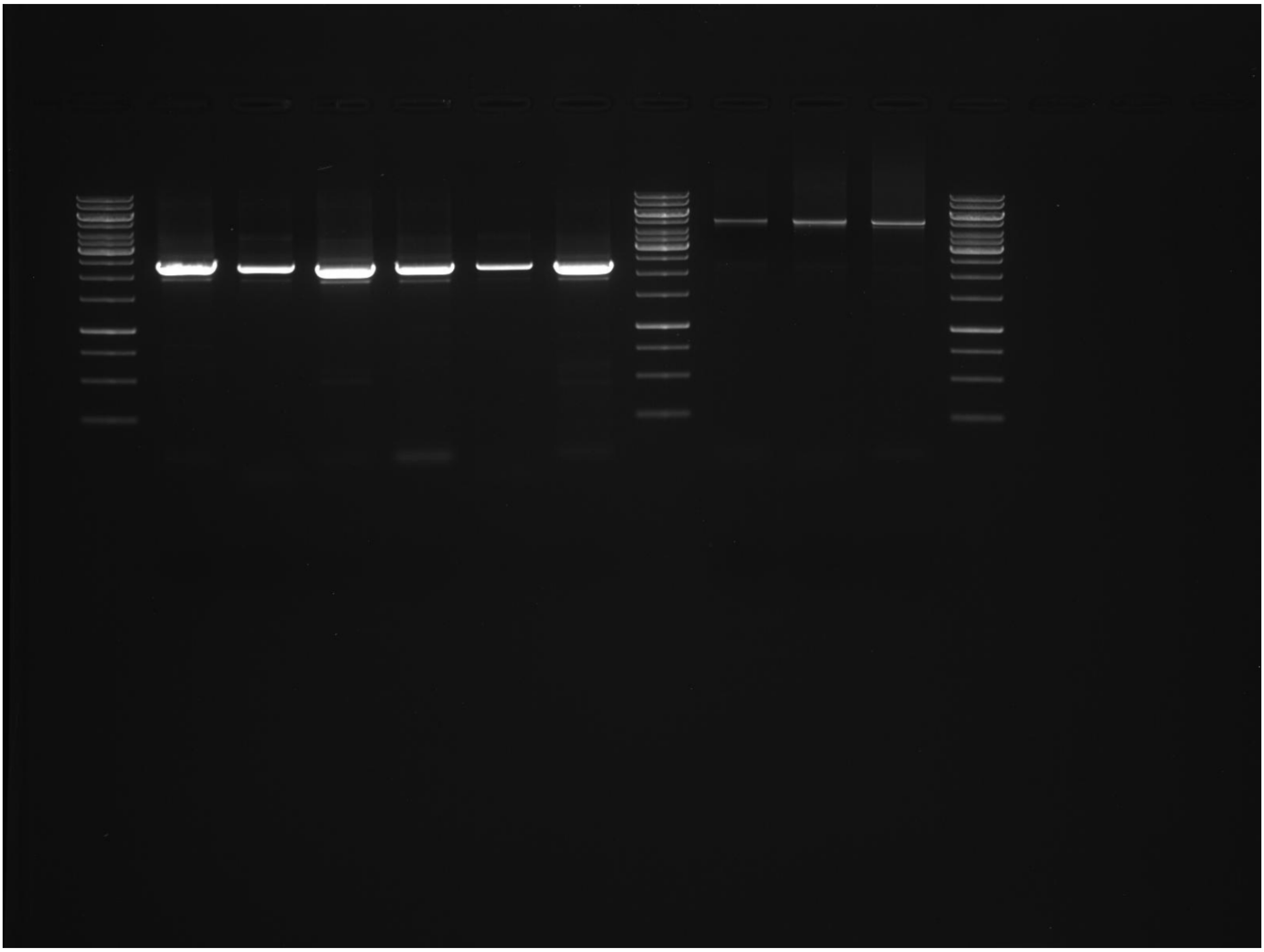

l)

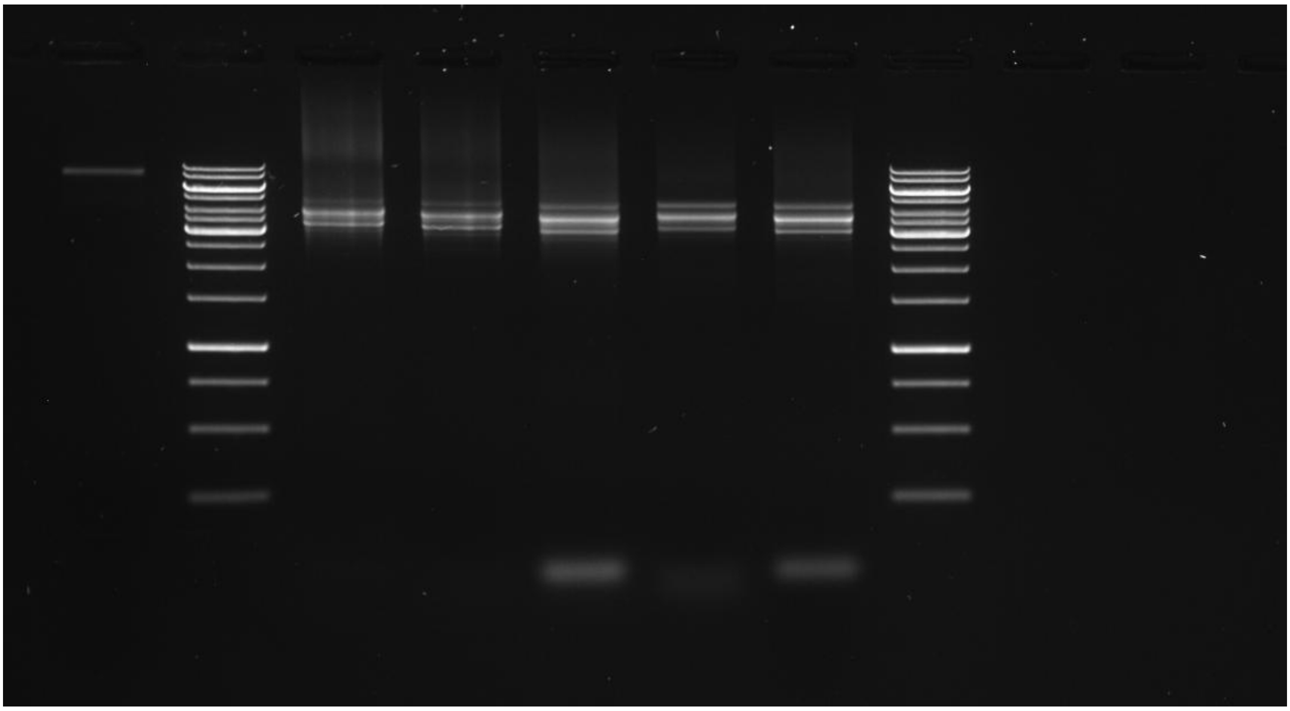

m)

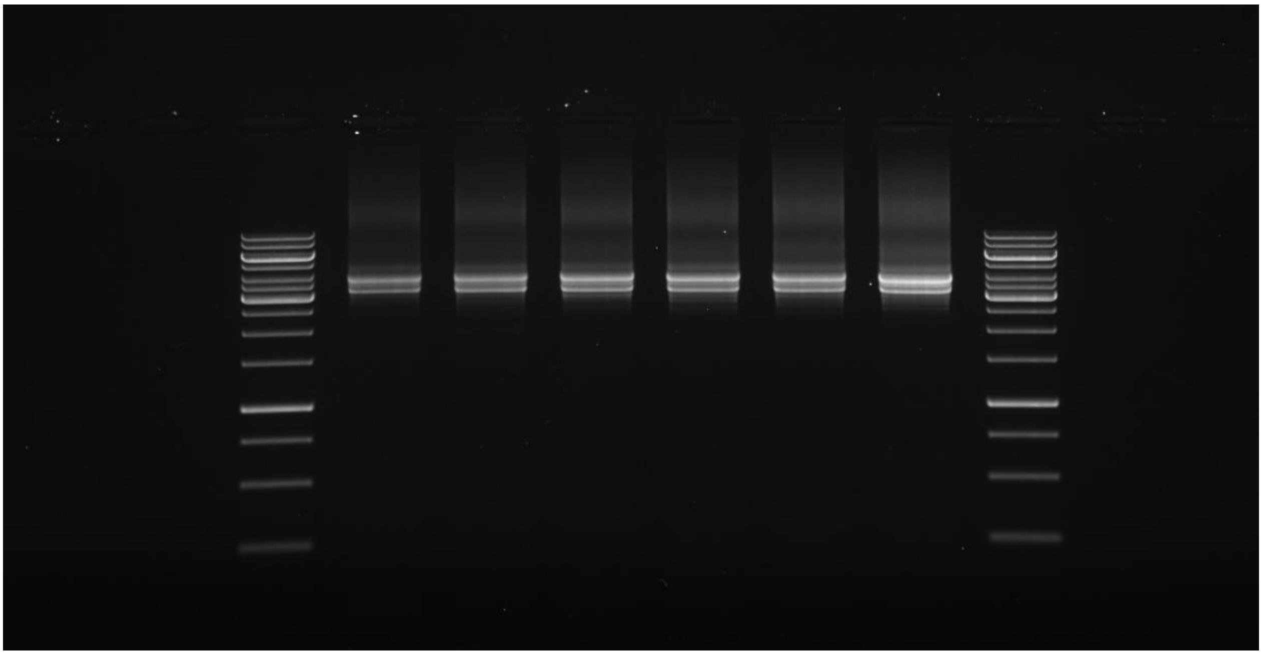

Supplement: Supplementary file 1 — Additional file 1: Fig. S1. Representative cropped agarose gels of CR1/CR2 amplicons obtained for Struthio camelus (a), Rhea pennata (b), Rhea americana (c) and Crypturellus tataupa (d). The numbering of amplicons separated in the agarose gel corresponds to the reaction numbered in Table S1 in Additional file 2. The numbers of PCR fragments, which were finally sequenced, are underlined. Lane M - GeneRuler 1 kb DNA Ladder (Thermo Scientific). Original uncropped and unprocessed gels are presented in Fig. S13a-d. Fig. S2. Sequence comparison of two copies of tRNA-Pro, ND6from, tRNA-Glu genes and control region, found in this study as duplicated, with appropriate sequences of mitogenomes previously deposited in GenBank and showing a typical avian gene order. The alignments are shown for Struthio camelus (a), Rhea americana (b), Rhea pennata (c) and Crypturellus tataupa (d). Dots indicate residues identical in the obtained copies with those in the single sequence previously published. Genes for tRNA-Pro are marked in yellow coloring, NADH dehydrogenase subunit 6 in cyan coloring, tRNA-Glu in grey shading, and control regions in red fonts. In the case of Crypturellus tataupa species only two obtained copies are compared to each other because the previously published mitogenome of these taxa is incomplete and it does not contain any of the analyzed genes or the control region. The sequences were aligned with MUSCLE [125] in MEGA [124]. Fig. S3. Sequences of mitogenomic fragments amplified and sequenced in this study for Struthio camelus (MH264503), Rhea americana (MK696563), Rhea pennata (MK696564) and Crypturellus tataupa (MK696562). Genes for tRNA-Leu are marked in bold, NADH dehydrogenase subunit 5 in magenta coloring, cytochrome b in red coloring, tRNA-Thr in green coloring, tRNA-Pro in yellow coloring, NADH dehydrogenase subunit 6 in cyan coloring, tRNA-Glu in grey shading, 12S rRNA in blue fonts, tRNA-Val in brown fonts, 16S rRNA in green fonts, tRNA-Phe are in bold [file 12864_2020_7284_MOESM1_ESM.pdf]
